# Lineage-Specific Methyltransferases Define the Methylome of the Globally Disseminated *Escherichia coli* ST131 Clone

**DOI:** 10.1128/mBio.01602-15

**Published:** 2015-11-17

**Authors:** Brian M. Forde, Minh-Duy Phan, Jayde A. Gawthorne, Melinda M. Ashcroft, Mitchell Stanton-Cook, Sohinee Sarkar, Kate M. Peters, Kok-Gan Chan, Teik Min Chong, Wai-Fong Yin, Mathew Upton, Mark A. Schembri, Scott A. Beatson

**Affiliations:** a Australian Infectious Diseases Research Centre and School of Chemistry and Molecular Biosciences, The University of Queensland, St. Lucia, Australia; b Faculty of Science, Division of Genetics and Molecular Biology, Institute of Biological Sciences, University of Malaya, Kuala Lumpur, Malaysia; c School of Biomedical and Healthcare Science, Plymouth University Peninsula Schools of Medicine and Dentistry, Plymouth, United Kingdom; UT Southwestern Medical Center Dallas

## Abstract

*Escherichia coli* sequence type 131 (ST131) is a clone of uropathogenic *E. coli* that has emerged rapidly and disseminated globally in both clinical and community settings. Members of the ST131 lineage from across the globe have been comprehensively characterized in terms of antibiotic resistance, virulence potential, and pathogenicity, but to date nothing is known about the methylome of these important human pathogens. Here we used single-molecule real-time (SMRT) PacBio sequencing to determine the methylome of *E. coli* EC958, the most-well-characterized completely sequenced ST131 strain. Our analysis of 52,081 methylated adenines in the genome of EC958 discovered three ^m6^A methylation motifs that have not been described previously. Subsequent SMRT sequencing of isogenic knockout mutants identified the two type I methyltransferases (MTases) and one type IIG MTase responsible for ^m6^A methylation of novel recognition sites. Although both type I sites were rare, the type IIG sites accounted for more than 12% of all methylated adenines in EC958. Analysis of the distribution of MTase genes across 95 ST131 genomes revealed their prevalence is highly conserved within the ST131 lineage, with most variation due to the presence or absence of mobile genetic elements on which individual MTase genes are located.

## IMPORTANCE

DNA modification plays a crucial role in bacterial regulation. Despite several examples demonstrating the role of methyltransferase (MTase) enzymes in bacterial virulence, investigation of this phenomenon on a whole-genome scale has remained elusive until now. Here we used single-molecule real-time (SMRT) sequencing to determine the first complete methylome of a strain from the multidrug-resistant *E. coli* sequence type 131 (ST131) lineage. By interrogating the methylome computationally and with further SMRT sequencing of isogenic mutants representing previously uncharacterized MTase genes, we defined the target sequences of three novel ST131-specific MTases and determined the genomic distribution of all MTase target sequences. Using a large collection of 95 previously sequenced ST131 genomes, we identified mobile genetic elements as a major factor driving diversity in DNA methylation patterns. Overall, our analysis highlights the potential for DNA methylation to dramatically influence gene regulation at the transcriptional level within a well-defined *E. coli* clone.

## INTRODUCTION

*Escherichia coli* sequence type 131 (ST131) is a clone of uropathogenic *E. coli* (UPEC) that has emerged rapidly and disseminated globally in both clinical and community settings. ST131 strains have been frequently isolated from patients with urinary tract infection (UTI) and bloodstream infection and represent a major clone of multidrug-resistant *E. coli*. Strain EC958 was originally isolated from a patient presenting with community-acquired UTI in 2005 in the United Kingdom (1) and is one of the most-well-characterized strains of ST131. EC958 has an O25b:H4 serotype (2), encodes a CTX-M-15-type extended-spectrum β-lactamase (ESBL) (3–5), is resistant to fluoroquinolones, and belongs to the *fimH*-based *fimH30* group (1), which we redefined as clade C in our recent phylogenomic analysis (6). Clinical evidence suggests that some ST131 pathogens are highly virulent (7), and the EC958 genome contains a number of genes that are associated with pathogenicity, including those coding for adhesins, autotransporter proteins, and siderophore receptors (1, 8). EC958 also expresses type 1 fimbriae, which are required for adherence and invasion of human bladder cells, as well as colonization of the mouse bladder (1). In animal models, EC958 causes acute and chronic UTI (9) and impairment of ureter contractility (10).

Using transposon-directed insertion site sequencing (TraDIS), we comprehensively defined the serum resistome of EC958 (11). As part of that study, we also identified a number of genes that were essential for EC958 growth but had no close homologs in other sequenced *E. coli* genomes. Two such genes (EC958_0008 and EC958_0009) were identified as coding for methyltransferases (MTases) that formed part of a restriction-modification (R-M) system (11). Previously, DNA adenine methylase (Dam) has been shown to regulate several UPEC virulence factors, including antigen 43 (Ag43) and P fimbriae (12, 13). However, as yet the role of MTases in any UPEC lineage has not been fully explored.

The most common DNA modification in bacteria, postreplicative, is methylation, with at least some form present in nearly all bacterial species (14). Methylation of nucleotides occurs in three ways: *N*^6^-methyladenine (^m6^A), *N*^4^-methylcytosine (^m4^C), and 5-methylcytosine (^m5^C). Genomic analysis has shown that DNA MTases are sometimes encoded within the vicinity of a restriction endonuclease (REase), suggesting that they form an R-M system. In bacteria, R-M systems are ubiquitous, extremely diverse, and largely uncharacterized (15). Functional systems are traditionally thought to be involved in the protection of the host genome from the invasion of foreign DNA such as phages, plasmids, and transposons. Methylation of specific bases may also impart additional epigenetic information that has the potential to act as a signal for genome defense, initiation of chromosome replication and repair, nucleoid segregation, regulation of gene expression, and transposition control (12). In their simplest form, R-M systems are comprised of an MTase that catalyzes the transfer of a methyl group from an *S*-adenosylmethionine (SAM) donor and its cognate REase that cleaves unmethylated DNA at internal phosphodiester bonds in the DNA backbone (16).

R-M systems are classified into four groups on the basis of subunit composition, cleavage position, sequence specificity, and cofactor requirements (17). Type I R-M systems are comprised of three subunits—the specificity (S), modification (M), and restriction (R) subunits—and are encoded by three genes, *hsdS*, *hsdM*, and *hsdR*, respectively (18). Type II R-M systems consist of two independently acting enzymes that mediate methylation and restriction, respectively. They include most commercially available restriction enzymes and are the most common of the four types (14). Type III systems consist of two subunits, the MTase and REase. The MTase subunit can function independently to hemimethylate DNA (19, 20), but the REase subunit must form a complex with the MTase for restriction activity (21). Type IV modification-dependent enzymes are related to type II REases; however, they cleave methylated DNA and require a methyl donor for successful cleavage (15). Classification of type IV R-M systems remains an evolving area of research (22).

MTases are also found independent of R-M systems, and these orphan MTases have been proposed to act as molecular vaccines, protecting the host chromosome from restriction attack (23). Dam is a well-characterized orphan MTase that methylates adenines at the *N*^6^ position of its recognition sequence, 5′-GATC-3′ (24–26). Dam can methylate both unmethylated and hemimethylated DNAs with similar efficiency (26, 27). Dam is dispensable in certain bacterial genera (e.g., *Escherichia* and *Salmonella*) (28, 29) but essential in others (e.g., *Vibrio* and *Yersinia*) (30, 31). It has been proposed that Dam is involved in the coordination of DNA replication in bacteria with more than one chromosome, such as *Vibrio* and *Yersinia*, perhaps explaining its importance in these genera (32). Dam has also been shown to influence gene expression and normal cellular processes (27) and to influence virulence in a number of pathogenic bacteria (33). Another well-characterized orphan MTase, DNA cytosine methylase (Dcm or Mec in early literature), methylates the internal cytosine residues at the *N*5 position in the sequence 5′-CCWGG-3′ (W = T or A) (24, 34). Methylation by Dcm provides partial protection of DNA against cleavage by several REases (e.g., EcoRII) (35).

The lack of high-throughput methods to efficiently detect DNA base modifications on a genome-wide scale has hindered the capacity to fully characterize the functional consequences of methylation in bacteria. Single-molecule real-time (SMRT) sequencing technology now enables the exact position of a methylated base to be examined on a genome-wide scale. The technology allows the synthesis of DNA to be monitored in real time, and methylated bases are detected by variance in the kinetic signatures of the reaction; the activity of the polymerase enzyme slows in a predictable manner that is determined by the modified base. ^m6^A and ^m4^C provide the most robust signatures, allowing their detection with high accuracy (36) due to their direct involvement in base pairing (37).

Here we defined the complete methylome of the ST131 strain EC958 using Pacific Biosciences (PacBio) SMRT sequencing. We took advantage of the kinetic signatures to determine the position of methylated bases within specific motifs. We undertook bioinformatic analysis of the entire EC958 genome to identify putative MTases and define the methylation pattern of their target sequences. MTases with equivocal methylation patterns were characterized by SMRT sequencing of isogenic knockout (KO) mutants. Finally, we investigated the distribution and diversity of MTase genes and their cognate recognition sites throughout the ST131 lineage.

## RESULTS

### Bioinformatic survey of EC958 restriction-modification systems.

**FIG 1:**
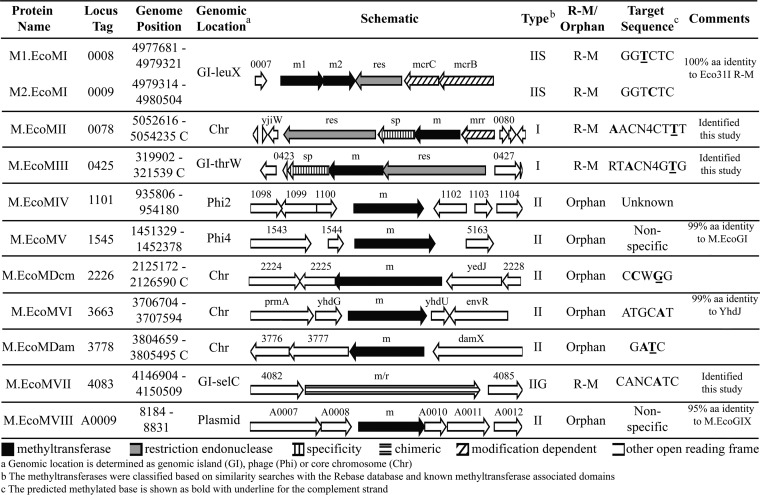
Detailed summary of R-M systems from across the EC958 genome. A schematic representation showing the structure and genomic context of EC958 R-M systems and orphan MTases is presented. Genes are shaded according to their functional classification.

A comprehensive analysis of the *E. coli* EC958 genome revealed that the strain encodes 10 putative MTases on the chromosome and one on the multidrug resistance plasmid pEC958 (Fig. 1). In addition, two type IV modification-dependent systems were identified on the EC958 chromosome (data not shown). Based on homology to other characterized MTases, we were able to predict the target sites for 4 of the 11 MTases (including Dam and Dcm). Additionally, two of the orphan MTases are homologs of MTases (M.EcoMV and M.EcoMVI) previously reported to be inactive in other strains and are similarly predicted to be inactive in EC958 (see below). The five remaining EC958 MTases represent either novel enzymes with unknown specificity or homologs of previously identified putative MTases whose specificity has not been determined. Each identified MTase and REase is detailed below, labeled according to the relevant REBASE database entry.

### (i) M1.EcoMI/M2.EcoMI (EC958_0008/EC958_0009).

M1.EcoMI and M2.EcoMI share 100% amino acid identity with the two MTases that form the previously defined Eco31I type IIS R-M system (38–40). The M1.EcoMI gene encodes the ^m6^A-MTase, and M2.EcoMI gene encodes the ^m5^C-MTase, while the R-M system is completed by the cognate REase (EC958_0010) encoded on the opposite strand. Eco31I is a short-distance cutter and cleaves DNA close to the recognition sequence. M1.EcoMI is predicted to modify the 3′ adenine residue on the bottom strand and M2.EcoMI the 5′ cytosine residue on the top strand of the recognition sequence, 5′-GGTCTC-3′ (39). The M1.EcoMI amino acid sequence contains the *N*^6^ DNA methylase Pfam domain (PF02384) that is characteristic of adenine-specific MTases in the N-terminal region of the predicted protein. The M2.EcoMI amino acid sequence contains 2 distinct regions encoding DNA methylase Pfam domains (PF00145), one each in the N- and C-terminal regions, in addition to a predicted active site residue at C-232. Both M1.EcoMI and M2.EcoMI contain a series of previously defined motifs involved in SAM binding and catalysis, namely, motifs IX, X, I, IV, V, VI, VII, and VIII (40).

### (ii) M.EcoMII (EC958_0078).

M.EcoMII represents a previously undefined *E. coli* MTase, and its gene is expected to encode the M subunit of a type I R-M system. The M.EcoMII gene is in a typical type I operon (*hsdM-hsdS-hsdR*) and contains Pfam domains that are associated with adenine MTase activity in the N-terminal domain (PF12161), and C-terminal domain (PF02384 and PF13659). The M.EcoMII gene also contains conserved catalytic domains, including those associated with SAM binding. The S subunit, which includes two target recognition domains (TRDs) (PF01420), is encoded by EC958_0077, and its recognition domain shows no homology to any previously characterized R-M system, indicating that its target sequence specificity is yet to be determined. The associated R subunit is encoded by EC958_0076 and shows 92% amino acid identity to the R subunit of StySBLI from *Salmonella enterica* serovar Blegdam.

### (iii) EcoMIII (EC958_0425).

The M.EcoMIII gene was previously undefined and predicted to encode the M subunit of a type I R-M system. Like the M.EcoMII gene, the M.EcoMIII gene is located in a typical type I R-M operon (*hsdR-hsdM-hsdS*) exhibiting a central *N*^6^ MTase Pfam domain (PF02384) associated with adenine MTases, in addition to conserved catalytic domains. The S subunit, characterized by the presence of a single TRD, is predicted to be encoded by EC958_0424. The recognition domain of EC958_0424 shows no homology to previously defined R-M systems, indicating that the recognition sequence is undefined. The R subunit is encoded by EC958_0426 and contains Pfam domains associated with restriction subunits (PF04313 and PF04851).

### (iv) M.EcoMIV (EC958_1101).

The M.EcoMIV gene is predicted to encode a type II orphan MTase carried by prophage Phi2. The amino acid sequence of M.EcoMIV is 100% identical to those of a large number (>80) of type II DNA adenine MTases whose genes have been annotated in *E. coli* genomes, including P423_04965 in *E. coli* ST131 strain JJ1886 (GenPept accession no. AGY83843). The REBASE database classifies M.EcoMIV as a type IIA MTase, which recognizes a 4- to 8-bp asymmetric sequence. As yet, no recognition site has been determined for any homologs with >65% amino acid identity to M.EcoMIV, and consequently the type IIA designation remains putative. M.EcoMIV contains a characteristic D12 class *N*^6^-adenine-specific DNA methyltransferase domain (PF02086) and the conserved catalytic motif involved in SAM binding.

### (v) M.EcoMV (EC958_1545).

M.EcoMV is encoded on prophage Phi4 and shares 99% amino acid identity with M.EcoGI, previously identified in *E. coli* O104 C227-11 (41). The recognition sequence for M.EcoGI was previously determined to be nonspecific and did not produce detectable polymerase kinetic variation (KV) signatures for SMRT sequencing under standard LB broth growth conditions (41). The high level of sequence identity to M.EcoGI suggests that M.EcoMV may also have tightly controlled expression and activity.

### (vi) M.EcoMDcm (EC958_2226).

M.EcoMDcm shares 99% amino acid identity with DNA cytosine MTase or Dcm from *E. coli* K-12 and is encoded in the sytenic position in *E. coli* EC958. Dcm is a well-characterized orphan type II MTase and recognizes the sequence 5′-CCWGG-3′, where the 2nd cytosine in the target sequence is modified on both strands. Dcm contains a DNA methylase domain (PF00145) as well as defined catalytic motifs associated with cytosine MTases.

### (vii) M.EcoMVI (EC958_3663).

M.EcoMVI shares 99% amino acid identity to the previously described orphan CcrM-like MTase YhdJ (42). Both M.EcoMVI and YhdJ contain all of the required domains for a functional MTase, including a SAM binding pocket, the conserved catalytic domain, and an *N*^6^ MTase Pfam domain (PF01555) (42). Based on this homology, M.EcoMVI is predicted to be a type II MTase and to methylate the second adenine of the sequence 5′-ATGCAT-3′ with a preference for hemimethylated sites.

### (viii) M.EcoMDam (EC958_3778).

M.EcoMDam shares 99% amino acid identity with DNA adenine MTase or Dam from *E. coli* K-12 and is encoded in the sytenic position in *E. coli* EC958. Dam is an orphan type II MTase, recognizes 5′-GATC-3′ (26), and has been very well characterized in *E. coli* and *Salmonella* (43–46). M.EcoMDam contains Dam-specific domains and catalytic motifs and is predicted to behave in exactly the same manner.

### (ix) M.EcoMVII (EC958_4083).

M.EcoMVII shares 68% amino acid identity to the type IIG R-M systems RM.StyUK11V and RM.SenTFV, and as typically observed for type IIG R-M systems, the M and R subunits are encoded as a multidomain enzyme that contains both methylation and restriction activity. M.EcoMVII contains Pfam domains associated with MTases (PF13659) and conserved catalytic domains. M.EcoMVII is predicted to hemimethylate its target sequence in a manner characteristic of the IIG family of MTases.

### (x) M.EcoMVIII (pEC958_A0009).

M.EcoMVIII is encoded on the antibiotic resistance plasmid pEC958 and shares 99% amino acid identity with the M.EcoGIX MTase in *E. coli* O104:H4 strain C227-11 (41). M.EcoGIX has been previously reported as lacking target sequence specificity and did not produce detectable KV signatures during SMRT sequencing (41).

### (xi) McrBC (EC958_0011 and EC958_0012).

The type IV modification-dependent McrBC system was identified in EC958 upstream of the Eco31I homologous R-M system (MTases 1 and -2). The same type IV system is located in a syntenic location in *E. coli* K-12. McrBC cleaves DNA containing methylcytosine on one or both strands. Its recognition sequence is 5′-R^m^C (N_40–3000_) R^m^C-3′, where the two half-sites of (G/A)^m^C can be separated by up to 3 kb; however, the optimal separation is 55 to 103 bp (47, 48). Based on sequence conservation we expect EC958_0011 and EC959_0012 to behave in a similar manner. McrBC does not restrict at Dcm sites.

### (xii) Mrr (EC958_0079).

Mrr, another type IV modification-dependent system, was also identified in EC958. Mrr is adjacent to the M.EcoMII type I system in EC958 and its gene is in a syntenic location in *E. coli* genomes that also contain the system. Mrr cleaves DNA that contains either methylcytosine or methyladenine; however, its specific target recognition sequence has not been defined. Mrr does not restrict either Dcm or Dam sites.

### EC958 MTases exhibit variable transcription levels.

**FIG 2:**
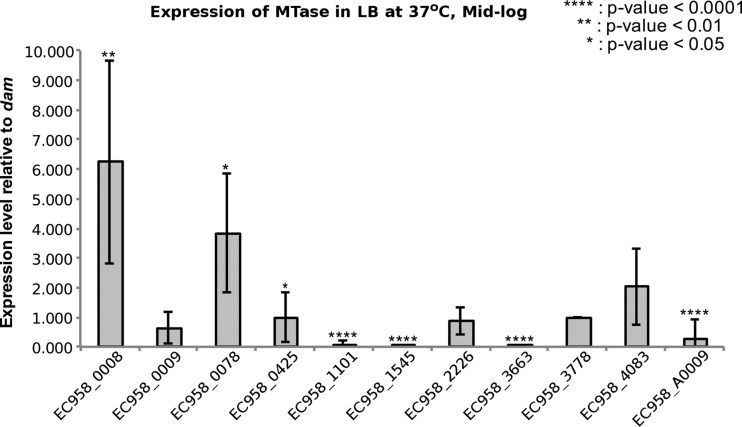
Relative expression levels of MTase genes in *E. coli* EC958. The graph shows the fold difference in expression levels of each MTase gene relative to the gene coding for M.EcoMDam (EC958_3778). MTases with expression levels similar to or higher than those of M.EcoMDam were presumed to be active in EC958. MTases with significant differences are indicated by asterisks. Measurements were performed in at least quadruplicates.

We employed quantitative reverse transcription-PCR (RT-PCR) to determine the transcription level of MTase genes in EC958 during the mid-log growth phase in LB broth at 37°C. Figure 2 shows the transcription level of each MTase gene compared to the *dam* gene (coding for M.EcoMDam). The M1.EcoMI and M.EcoMII genes were transcribed at a significantly higher level than M. EcoMDam (*P* = 0.0015 and 0.0497, respectively). In contrast, the M.EcoMIII, M.EcoMIV, M.EcoMV, M.EcoMVI, and M.EcoMVIII MTase genes were transcribed at a significantly lower level than the M.EcoMDam gene. The remaining three MTase genes, coding for M2.EcoMI, M. EcoMDcm, and M.EcoMVII, were transcribed at a similar level to the Dam MTase gene. Based on these results, we predict that in addition to Dam and Dcm, at least four other MTases were active in EC958 under the conditions tested in this study.

### Target specificity of EC958 MTases.

**FIG 3:**
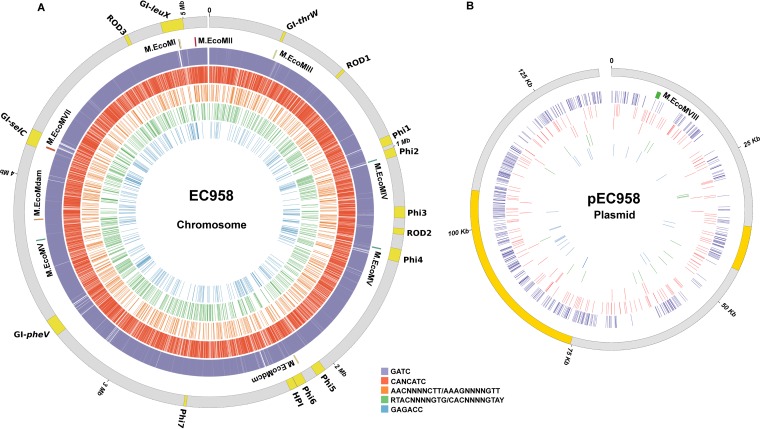
Circos plots displaying the distribution of methylated bases in the *E. coli* EC958 chromosome (A) and large plasmid pEC958 (B). The locations of MGEs on the chromosome (A) and plasmid antibiotic-resistance regions (B) are indicated on the outermost track in yellow. The relative positions of the MTases are indicated on the second outermost track. MTase expression levels are based on a scale from red to green, where red represents high expression relative to Dam and green represents low expression relative to Dam. The remaining colored tracks display the location of methylated sites for each motif. From outer to inner: GATC, purple (M.EcoMDam); CANCATC; red (RM.EcoMVII), AACN_4_CTTT, orange (RM.EcoMII); RTACN_4_GTG, green (M.EcoMIII); GAGACC, blue (RM.EcoMI). Tick marks display the genomic positions in megabases (A) and kilobases (B).

The genome-wide distribution of methylated bases in *E. coli* EC958 was determined using PacBio SMRT sequencing technology. A total of 52,081 genomic positions were found to be methylated: 50,822 on the chromosome and a further 1,259 on the large plasmid pEC958 (Fig. 3). Based on the kinetic profiles, these methylated bases were found to be predominately *N*^6^-methyladenine (^m6^A) modifications (97.19% of all modified sites). However, clustering of methylated nucleotides based on sequence context identified only five distinct recognition motifs corresponding to five MTase recognition sequences: 5′-G^m6^ATC-3′, 5′-CANC^m6^ATC-3′, 5′-GAG^m6^ACC-3′, 5′-A^m6^ACN_4_CTTT-3′, and 5′-RT^m6^ACN_4_GTG-3′. (Underlined bases indicate the detection of a methylated base on the complementary strand.) Two of the five recognition motifs matched type II MTases with known specificities: G^m6^ATC is a well-characterized methylation motif targeted by Dam, and GAG^m6^ACC is predicted to be targeted by M1.EcoMI, based on its 100% amino acid identity to the previously characterized M1.Eco31I (39). The M1.Eco31I recognition site is better known in its complementary form (5′-GGTCTC-3′), exhibiting cytosine methylation on one strand and adenine methylation on the other (39) (Fig. 1). Adenine methylation of 5′-GGTCTC-3′ was detectable by SMRT sequencing, whereas cytosine methylation (5′-GGT^m5^CTC-3′) is predicted in EC958 based on (i) the presence of an intact M2.EcoMI enzyme encoded adjacent to the gene for M1.EcoMI (locus tags EC958_0009 and EC958_0008, respectively) and (ii) an apparently full-length Eco31I restriction enzyme encoded in the same locus (Fig. 1).

The remaining three recognition motifs could not be assigned to the other identified EC958 MTases as they do not match any previously described MTase recognition sequence and as such represent novel methylation sites that may be unique to the ST131 lineage. Two of these three motifs, A^m6^ACN_4_CTTT and RT^m6^ACN_4_GTG, contain a stretch of degenerate bases that are characteristic of type I MTases (18) and are likely methylated by either of the two putative type I MTases encoded by the M.EcoMII and M.EcoMIII genes. SMRT sequencing of EC958 and bioinformatic characterization of its MTases did not identify an MTase that could recognize the CANC^m6^ATC motif.

Of the remaining six EC958 MTases whose genes are predicted in the EC958 genome, two are known to possess C5-methylcytosine (^m5^C) MTase activity (M.EcoMDcm and M2.EcoMI). Treatment of genomic DNA with the Ten-eleven translocation (Tet) family of proteins, to enhance detection of ^m5^C methylated DNA (49), was not undertaken, and consequently, ^m5^C methylated bases could not be discriminated from unmodified bases. However, as both Dcm and homologs of M2.EcoMI have previously been well characterized and are known to recognize the motifs C^m5^CWGG and GGT^m5^CTC, respectively, we predict that both are functional MTases in EC958 (Fig. 1). M.EcoMVI, is highly similar to the previously characterized type II orphan MTase YdhJ, which targets ATGC^m6^AT motifs (42). However, our transcriptional data suggest that M.EcoMVI is inactive in EC958 (Fig. 2), and consequently its target site could not be explicitly determined. There are also two prophage-encoded MTases: M.EcoMIV, which is predicted to be a Dam homolog, and M.EcoMV, which is most similar to the previously characterized M.EcoGI (41). No methylation patterns could be assigned for either M.EcoMIV or M.EcoMV; however, both are predicted to be inactive in EC958 under the conditions tested based on our RT-PCR analysis (Fig. 2). Finally, the plasmid pEC958A encodes M.EcoMVIII, a predicted type II orphan MTase highly similar to the previously characterized plasmid-encoded M.EcoGIX, which methylates adenine residues independently of sequence context (41). We predict that M.EcoMVIII has similar nonspecific methylation activity to M.EcoGIX.

### Assignment of novel methylation motifs to specific MTase genes.

To identify MTases that methylate the three novel recognition motifs defined in this study, candidate R-M systems (M.EcoMII, M.EcoMIII, and RM.EcoMVII) were disrupted by targeted gene knockout. Genomic DNA from the isogenic mutants was subjected to SMRT sequencing, and their methylome profiles were compared to that of the EC958 parent strain (see Table S5 in the supplemental material). The functional inactivation of the type I R-M systems EcoMII and EcoMIII resulted in the complete loss of methylation at AACN_4_CTTT and RTACN_4_GTG motifs, respectively (see Fig. S1A and S1B in the supplemental material). Similarly, disruption of the type IIG R-M system RM.EcoMVII resulted in the loss of CANCATC methylation (see Fig. S1C).

### The distribution of MTase-associated motifs in the genome of EC958.

In general, characterized MTase recognition motifs were found to be almost fully methylated in the genome of *E. coli* EC958 (see Table S1 in the supplemental material). On the chromosome, we found that >99% of adenines in GATC (Dam), CANCATC (M.EcoMVII), AACN_4_CTTT (M.EcoMII), and RTACN_4_GTG (M.EcoMIII) motifs and 100% of adenines in GAGACC (M1.EcoMI) motifs had characteristic kinetic profiles corresponding to ^m6^A modification. Similarly, on plasmid pEC958, four of these motifs were 100% methylated, whereas adenines in AACN_4_CTTT motifs were 95% methylated (see Table S1). Unmethylated Dam sites may be due to competition with DNA-binding proteins that block access to the GATC motif. In contrast, unmethylated sites that are recognized by an active restriction enzyme are likely to reflect limitations in SMRT base modification detection.

**FIG 4:**
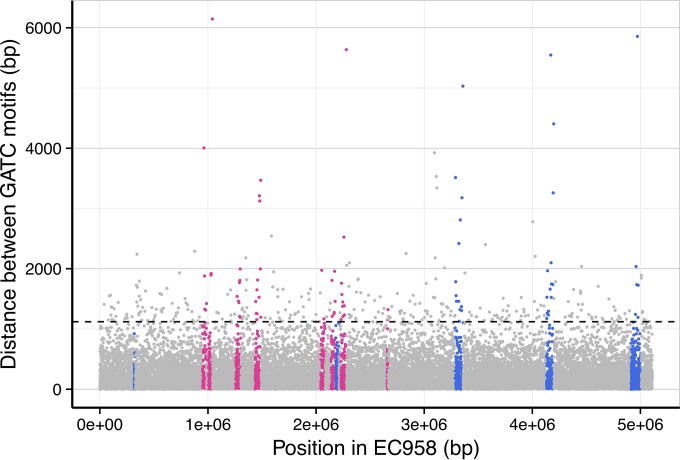
Distribution of GATC motifs in the core and accessory genome of *E. coli* EC958. The graph displays a linear representation of the EC958 chromosome showing the position of methylated GATC sites (*x* axis) and the distance between methylated GATC sites (*y* axis). Each GATC motif is represented by a single circle that has been colored based on its genomic context: genomic islands (GI-*thrW*, HPI, GI-*pheV*, GI-*selC*, and GI-*leuX*), blue; prophage (Phi1 to -7 and cryptic prophage), pink; core, gray. The dashed line denotes the boundary for outliers and is calculated as the mean distance between methylated GATC sites plus 3× the standard deviation.

The mean frequency of GATC Dam MTase sites is underrepresented in mobile genetic elements (MGEs), with significant differences between the non-MGE and MGE regions of the genome: genomic islands (GIs) GI-*pheV* (*P* < 0.0001) and GI-*selC* (*P* < 0.0001), prophages Phi1 to Phi7 (*P* ≤ 0.0001), and cryptic phage (*P* = 0.00026). The underrepresentation of GATC appears, at least in part, to be due to the relatively high frequency of GATC-free regions of ≥1 kb that are more likely to be located within MGEs compared to the rest of the chromosome (Fig. 4). Of the remaining methylated motifs in EC958, only CANCATC (M.EcoMVII) approaches Dam in terms of the number of sites in the genome (6,560 sites). However, unlike Dam there was no significant difference in the distribution of CANCATC motifs between non-MGE and MGE genomic locations. In contrast, adjusted *post hoc* testing revealed that the GAGACC (EcoMI) motifs were overrepresented in many prophage-associated regions and genomic islands in EC958 (see Table S1 in the supplemental material).

### Distribution of EC958 MTases within the ST131 lineage.

**FIG 5:**
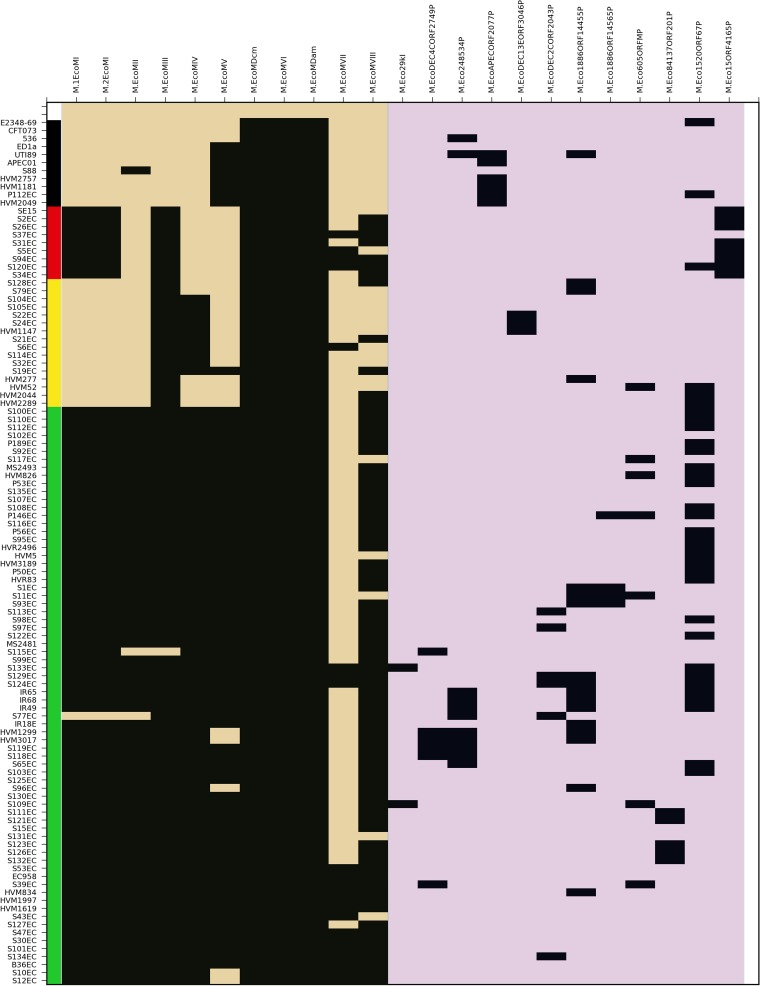
Distribution of MTases in ST131. MTases conserved in EC958 (tan) and those not encoded in EC958 (purple) are shown along the *x* axis with strain identifiers listed on the *y* axis in order of phylogenetic relatedness (6). Gene presence (black shading) is indicated by BLASTn comparison (≥95% nucleotide identity) of EC958 MTases and MTases from the REBASE database (15) to the draft assemblies of 95 ST131 strains and/or mapped reads for each ST131 strain (http://github.com/BeatsonLab-MicrobialGenomics/ST131_99/), as implemented in Seqfindr (http://github.com/mscook/seqfindr).

EC958 possesses several MTases whose genes are not found in the genomes of other completely sequenced UPEC strains (Fig. 5). The EC958 MTases show a distribution in other ST131 strains consistent with the presence or absence of MGEs on which they are encoded (Fig. 5). For example, (i) the GI-*leuX-*encoded R-M system EcoMI is completely absent in strains from clade B and the clade C strain S77, (ii) the GI-*thrW*-encoded M.EcoMIII is absent only from the clade C strain S115, (iii) the GI-*selC-*associated M.EcoMVII shows a distribution consistent with the variability of this element throughout ST131, and (iv) the Phi2- and Phi4-associated MTases M.EcoMIV and M.EcoMV, respectively, are completely absent in strains from clade A. In contrast, Dam, Dcm, and M.EcoMVI genes are present in all sequenced UPEC strains in this study (Fig. 5) and are found in syntenic locations among all *E. coli* isolates for which genome sequences are currently available (data not shown). The M.EcoMVII gene is the only EC958 MTase gene that was not found in the majority of ST131 genomes analyzed in this study.

To determine the full extent of MTase diversity throughout the ST131 lineage, we undertook a BLASTn comparison of the 95 *E. coli* ST131 genomes against the REBASE database. This enabled the identification of several additional MTase genes in the ST131 lineage that are absent from the genome of EC958 (see Tables S2 and S3 in the supplemental material). In the majority of cases, non-EC958 MTases were found in small phylogenetically linked clusters of isolates, indicating a likely ancestral acquisition of an MGE carrying the MTase gene. Acquisitions include four different type II MTases similar to M.Eco29KI, M.EcoDEC4CORF2749P, M.EcoDEC2CORF2043P, and M.Eco1886ORF14565P, respectively, and a single type I MTase similar to M.Eco84137ORF201P that were all exclusive to clade C strains; a type II orphan MTase, similar to M.Eco15ORF4165P, exclusive to strains from clade A; and a type II orphan MTase most similar to M.EcoDEC13EORF3046P, present only in several clade B strains (S22, S24, and HVM1147). The remaining five accessory MTase genes were not specific to any ST131 clade, and one gene (coding for M.Eco605ORFMP) was present in the ST131 lineage (clades B and C) but absent from all examined non-ST131 UPEC strains (Fig. 5; see Table S2).

## DISCUSSION

*E. coli* EC958 is a completely sequenced ST131 representative of the fluoroquinolone-resistant, *fimH30* clade C group. Here we have used SMRT sequencing and RT-PCR to identify the active ^m6^A MTases and methylated motifs within the genome of EC958. Subsequent SMRT sequencing of three EC958 knockout mutants allowed us to unequivocally assign three novel ^m6^A modification recognition motifs to their cognate MTases: AACN_4_CTTT, RTACN_4_GTG, and CANCATC were matched to M.EcoMII, M.EcoMIII, and M.EcoMVII, respectively.

Methylation is recognized as an important element in virulence, adaptability, and gene regulation, but bacterial methylomes have remained largely unexplored due to difficulties in obtaining epigenetic data on a whole-genome scale. Several recent studies have demonstrated the potential of SMRT sequencing to comprehensively characterize genome-wide methylome profiles across a range of bacteria. For example, Murray et al. characterized the methylomes of five Gram-negative bacteria and a single Gram-positive bacterium, which include the pathogens *Campylobacter jejuni* and *Bacillus cereus* (50). Fang et al. comprehensively characterized the methylome of the Shiga toxin-producing *E. coli* O104:H4 strain C227-11 from the 2011 German outbreak (41). Others have investigated the role of methylation in regulating the cell cycle in *Mycoplasma genitalium* and *Mycoplasma pneumoniae* (51), compared the methylomes of different *Helicobacter pylori* strains (52), or characterized the phase-variable MTase regulons of *Neisseria meningitidis* (53). This study represents the first description of the complete methylome of a strain from the globally disseminated multidrug-resistant *E. coli* ST131 lineage and indeed of any UPEC strain*.*

We identified only two EC958 MTases predicted to be capable of ^m5^C modifications, both of which have been previously characterized elsewhere (Dcm and an Eco31I homolog, encoded by the M.EcoMDcm and M2.EcoMI genes, respectively). Our analysis focused on the abundant ^m6^A modifications distributed throughout the genome as Tet treatment of DNA samples is normally required to identify ^m5^C modifications by SMRT sequencing. Previous methylome analyses have identified ^m6^A methylation as the predominant modification type in bacteria, with more than 90% of associated motifs methylated (41, 50–52). EC958 displays similarly high rates of ^m6^A modifications, with >96% of associated motifs methylated. In contrast, ^m4^C-modifying enzymes have only been fully characterized in *B. cereus* (50) and *H. pylori* (52). Consistent with other *E. coli* methylome studies (41, 54, 55), no ^m4^C MTase or ^m4^C motifs were identified in the genome of *E. coli* EC958. Interestingly, of the 436 *E. coli* genomes (162 complete and 274 draft) currently in the REBASE database (as of 13 April 2015), only one such *N*^4^-methylcytosine-modifying enzyme has been characterized in *E. coli* (M.EcoNI).

The Dam recognition site GATC is the most prevalent methylation motif throughout the *E. coli* EC958 genome. The role of Dam as a regulator of gene expression has been well established in other *E. coli* strains (33, 44–46, 56), and there is evidence that hemimethylated GATC sites play an important role in controlling transposition efficiency of mobile elements. For example, the transposition efficiency of Tn*10* is directly controlled by methylation of GATC sites (57), and hemimethylation of GATC sites in IS*10* increases transposition efficiency by enhancing binding of RNA polymerase to the transposase promoter region (57). The Tn*5* and Tn*903* transposons and the insertion element IS*3* also use hemimethylated GATC sites to control transposition (58, 59). Additionally, hemimethylated GATC sites also play an important role in Pap phase switching, and both Dam and the oxidative stress response regulator OxyR mediate on/off switching of the aggregation- and biofilm-associated protein antigen 43 (Ag43) (60, 61). A recent comparison of the methylome and expression profiles of *E. coli* O104:H4 and an *E. coli* O104:H4 mutant lacking the Shiga toxin phage-encoded functional R-M system M.EcoGIII identified 1,951 differentially expressed genes in the wild-type strain compared to the mutant (41), showing that MTases acquired as components of MGEs can have a dramatic effect on host gene expression. Interestingly, hemimethylation at CANCATC sites accounts for 12% of all ^m6^A modification in EC958 and suggests a putative regulatory role for EcoMVII, which is carried by the GI-*selC* genomic island in some clade B and clade C ST131 strains. Future work, coupling MTase knockouts with methylome and gene expression studies, should provide a clearer picture of the functional roles of all EC958 R-M systems and orphan MTases and help determine precisely how MTase-mediated DNA methylation intersects with gene expression in *E. coli* ST131.

Differences in the methylation motif distribution were found between the core and accessory genome of *E. coli* EC958. Notably, much of the difference in the distribution of GATC motifs between the core and accessory genome could be accounted for by “GATC-free” regions (≥1 kb), suggesting that there may be selective pressure against Dam methylation of certain parts of MGEs. GATC-free regions have been previously reported in a 1.6-Mbp segment of *E. coli* K-12, with distances of 2,300, 2,836, and 4,082 bp between GATC motifs observed (62). Additionally, rRNA operons have a very low occurrence of GATC motifs, which could represent a mechanism to minimize the effects of DNA replication on rRNA transcription (63). GATC-free regions greater than 1,000 bp were also identified in several *E. coli* K-12 genes, including *btuB* (1,202 bp), *hisT* (1,346 bp), *hsdS* (1,344 bp), *tyrT* (1,618 bp), and *pbpB* (1,236 bp) and regions that harbor tRNA genes, suggesting selection against GATC sites (64). In contrast, there are several well-known examples of hypermethylation of GATC sites reported. For example, *oriC* encodes a cluster of 11 Dam motifs within a 245-bp region that are involved in the initiation of chromosome replication and regulation of origin function (65). Additionally, many GATC sites are separated by less than 100 bases, with 2,700 instances occurring in the aforementioned 1.6-Mbp *E. coli* K-12 chromosome fragment (62). Of these instances, 148 genes contained abnormally high levels of GATC motifs; this includes genes associated with respiration, growth under anaerobic and aerobic conditions, and cell cycle regulation (62). Further analysis of the distribution of methylated sites in the context of the *E. coli* EC958 transcriptome and in the genomes of other *E. coli* ST131 strains should help to elucidate the reasons underlying differences in methylation motif distribution.

This study provides the first comprehensive analysis of the distribution of MTases within the ST131 lineage or indeed any UPEC clonal lineage. In general, EC958 MTases were well conserved within ST131, with variation in their distribution linked to the presence or absence of prophages, genomic islands or other MGEs. Prophage- and plasmid-encoded MTases are often promiscuous when methylating DNA, regardless of sequence context, and likely play a protective role during MGE acquisition (66). Although these enzymes are often transcriptionally silent in the host chromosome, their exogenous expression can reveal specific methylation activity (37, 41). Therefore, it is possible that MTases that are not expressed in EC958 under the conditions used in this study could be activated under specific stimuli. A number of non-EC958 MTases were also identified; however, only one of these (M.Eco1520ORF67P) was widely distributed in other ST131 strains. The sparse distribution of genes encoding MTases that are not encoded in EC958 suggests their carriage on MGEs (such as plasmids); however, further complete genome sequencing will be required to fully investigate this relationship in ST131.

R-M systems are known to inhibit the uptake of non-self DNA, restrict horizontal gene transfer, and function in maintaining species identity (67–69). The role of R-M systems in restricting intraspecies DNA exchange is less well studied (68), but recently it has been shown that R-M systems can also generate barriers to DNA exchange between members of the same species (70, 71). In *Neisseria meningitidis*, different lineages were associated with unique complements of R-M systems. Intraclade DNA exchange was found to be 2-fold and 40-fold higher than interclade DNA exchange for short (<1 kb) and long (>5 kb) DNA sequences, respectively (71). More recently, lineage-specific R-M systems and methylation patterns were described in *Burkholderia pseudomallei* (70). Transformation with reporter plasmids carrying specific restriction sites was effectively prevented in *E. coli* strains transformed with genes encoding cognate *B. pseudomallei* R-M systems (70). In both *N. meningitidis* and *B. pseudomallei*, acquisition of functioning R-M systems as components of MGEs has established significant barriers to interclade DNA exchange. In EC958, all functional MTases (excluding Dam) were components of restriction modification systems acquired as part of MGEs. The high rate of methylation of these active EC958 MTases (~100%) suggests that lineage- and clade-specific patterns of methylation could contribute to shaping the gene pool accessible to ST131.

To date, the methylome of six *E. coli* strains has been characterized: O104:H4 C227-ll, O145:H28 RM13514 and RM13516, BL21(DE3), Bal225, and DH5ɑ (41, 54, 55). These studies have shown that the R-M gene complement can vary greatly between strains, identified several novel R-M systems with previously uncharacterized specificity, and provided novel insights into the functional activity of these enzymes. Our analysis of the EC958 methylome has identified three previously uncharacterized recognition sites (CANCATC, AACN_4_CTTT, and RTACN_4_GTG) and their cognate MTase enzymes. Additionally, analysis of the distribution of EC958 MTases within the ST131 lineage highlights the importance of MGEs in the dissemination of these MTase genes, even among clonally related strains. Overall, the methylome of EC958 provides a framework for future investigation into the role of epigenetics in the evolution of the ST131 lineage.

## MATERIALS AND METHODS

### SMRT sequencing and detection of modified bases.

Genomic DNA was extracted from an overnight culture of *E. coli* EC958 and sequenced on a PacBio RSI SMRT sequencing instrument as previously described (8). Genome-wide detection of modified bases (36, 37) and identification of associated motifs were performed using the RS_Modification_and_Motif_Analysis.1 tool from the SMRT analysis package version 2.1.0. Eight SMRT cells of sequence data were mapped to the chromosome and large plasmid (pEC958) of *E. coli* EC958, achieving ~132× and 185× coverage, respectively. Interpulse durations (IPDs) were measured, and the IPD ratio for each base was determined using an *in silico* kinetic reference computational model (http://www.pacb.com/wp-content/uploads/2015/09/WP_Detecting_DNA_Base_Modifications_Using_SMRT_Sequencing.pdf). The accuracy of modification detection using this model was increased by comparing the observed IPD ratios to the expected signatures of the three bacterial modification types: ^m6^A, ^m4^C, and ^m5^C. Sequence motif cluster analysis was done using PacBio Motif finder v1 with a quality value (QV) cutoff of 30.

### Statistical analysis of methylation motif distribution.

To compare the methylation motif distributions of MGEs with the rest of the chromosome, the sequence for each strand was split into 1,000-bp segments with a 250-bp overlap using Bedtools v2.17.0 (72). We have previously defined the major MGEs of *E. coli* EC958, which include five genomic islands (GI-*thrW*, HPI, GI-*pheV*, GI-*selC*, and GI-*leuX*) and eight prophage regions (Phi1 to -7 and a cryptic prophage) (1, 8). The coordinates of each MGE were used to extract all corresponding ≥1-kb segments that did not contain GATC motifs (referred to herein as GATC-free regions). The frequency of each motif within each segment was determined using a custom Python script. Analysis of the mean distribution of individual methylation motifs per segment within these genomic regions was performed using an analysis of variance (ANOVA) and a custom R script. As these data exceeded the assumptions of an ANOVA, the analysis was adjusted for heteroscedasticity (R multcomp package [73] and sandwich package [74]). Adjusted *P* values were reported if below the α significance region (α = 0.05, two-sided test). Custom scripts used in this analysis are available on Github at http://github.com/BioMinnie/MotifDistributionStatistics.

### RT-PCR analysis.

The transcription of the 11 MTase genes found in *E. coli* EC958 was measured by quantitative RT-PCR. RNA extraction was made using RNeasy minikit (Qiagen) from bacterial cells grown in LB broth at mid-log phase (optical density of ~0.4). Synthesis of cDNA was done using SuperScript III reverse transcriptase (Invitrogen, Life Technologies). Quantitative RT-PCR was performed in at least quadruplicates using ABI SYBR green PCR master mix on the ViiA 7 real-time PCR system (Life Technologies) with a cycling program of 95°C for a 10-min initial denaturation, followed by 40 cycles of denaturation at 95°C for 15 s and annealing at 60°C for 15 s, followed by extension at 72°C for 30 s. Significant differences in expression levels were determined by one-way ANOVA followed by Dunnett’s multiple comparisons test.

### MTase diversity.

MTase genes identified in EC958 and from the REBASE database (15) were searched against the draft genomes of 95 ST131 strains (6) (BLASTn, ≥95% nucleotide identity). The presence or absence of MTase genes was visualized using Seqfindr (http://github.com/mscook/seqfindr). Assembly and mapping modes were used to eliminate false negatives by ensuring that MTase genes absent in the assembled contigs would be identified in the read data if present. SeqfindR results were verified using BLAST (75) (see Table S3 in the supplemental material).

### Construction EC958ΔMTase mutants.

EC958 mutants containing deletions in the MTase genes were constructed by λ *red*-mediated recombination as previously described (1, 76) using a three-step PCR procedure (77). In brief, for each mutant three PCR products were made, including a chloramphenicol resistance cassette from plasmid pKD3 and two 500-bp homologous regions flanking the gene of interest (see Table S4 in the supplemental material). The three products were fused by PCR and electroporated into EC958 harboring a gentamicin-resistant plasmid carrying the λ *red* recombinase gene. Mutants were then selected on LB agar supplemented with chloramphenicol (30 µg/ml) and confirmed by Sanger sequencing the ends of PCR products designed to amplify the target gene (see Table S4). Detection of modified bases was carried out as described above using PacBio RS II (2 SMRT cells per mutant, P4C2 chemistry).

### Accession numbers.

The complete sequence of the *E. coli* EC958 chromosome (5,109,767 bp) and two plasmid sequences pEC958 (135,600 bp) and pEC958B (4080 bp) have been deposited in the European Nucleotide Archive (ENA) under accession no. HG941718, HG941719, and HG941720. The raw SMRT sequence read data presented in this article were deposited in the Sequence Read Archive (SRA) under accession no. SRP058069 (EC958 wild-type strain) and SRP058075 (EC958 isogenic KO mutants [SRS931034, SRS931035, and SRS931037]). The raw data can also be retrieved from http://beatsonlab.com/pages/data.

## SUPPLEMENTAL MATERIAL

Figure S1  Representative IPD ratio plots of RM.EcoMII (A), RM.EcoMIII (B), and RM.EcoMVII (C) recognition motifs. Each plot shows a subsection of the *E. coli* EC958 genome that contains one of the aforementioned novel R-M recognition sites and a Dam site as a control. The wild-type *E. coli* EC958 IPD ratio plots (top) show that under normal conditions, the 5′-AACN_4_CTTT-3′ motif (A), 5′-RTACN_4_GTG-3′ motif (B), and 5′-CANCATC-3′ (C) are methylated. Isogenic knockout mutant IPD ratio plots (bottom) show the absence of specific methylation and that Dam methylation is unaffected. Methylated bases are indicated by the large IPD ratios, colored purple at Dam sites (G^m6^ATC), yellow at M.EcoMII recognition sites (A^m6^ACN_4_CTTT), green at M.EcoMII recognition sites (RT^m6^ACN_4_GTG), and orange at M.EcoMVII sites (CANC^m6^ATC). Download Figure S1, PDF file, 0.2 MB

Table S1  Summary of recognition motifs identified in *E. coli* EC958. Table S1, PDF file, 0.1 MB

Table S2  ST131 accessory MTases. Table S2, PDF file, 0.02 MB

Table S3  BLAST result summary for *E. coli* ST131 genomes versus REBASE protein sequences. Table S3, PDF file, 0.4 MB

Table S4  Primers used in this study. Table S4, PDF file, 0.2 MB

Table S5  Assignment of novel methylation motifs to specific MTase genes. Table S5, PDF file, 0.03 MB
