# Supplementary material for: Lineage-Specific Methyltransferases Define the Methylome of the Globally Disseminated Escherichia coli ST131 Clone
Source: mBio. 2015 Nov 17;6(6):e01602-15. doi: 10.1128/mBio.01602-15 (PMC4659465; doi:10.1128/mBio.01602-15)
Supplement: Figure S1 — Representative IPD ratio plots of RM.EcoMII (A), RM.EcoMIII (B), and RM.EcoMVII (C) recognition motifs. Each plot shows a subsection of the E. coli EC958 genome that contains one of the aforementioned novel R-M recognition sites and a Dam site as a control. The wild-type E. coli EC958 IPD ratio plots (top) show that under normal conditions, the 5′-AACN4CTTT-3′ motif (A), 5′-RTACN4GTG-3′ motif (B), and 5′-CANCATC-3′ (C) are methylated. Isogenic knockout mutant IPD ratio plots (bottom) show the absence of specific methylation and that Dam methylation is unaffected. Methylated bases are indicated by the large IPD ratios, colored purple at Dam sites (Gm6ATC), yellow at M.EcoMII recognition sites (Am6ACN4CTTT), green at M.EcoMII recognition sites (RTm6ACN4GTG), and orange at M.EcoMVII sites (CANCm6ATC). Download [file mbo005152543sf1.pdf]

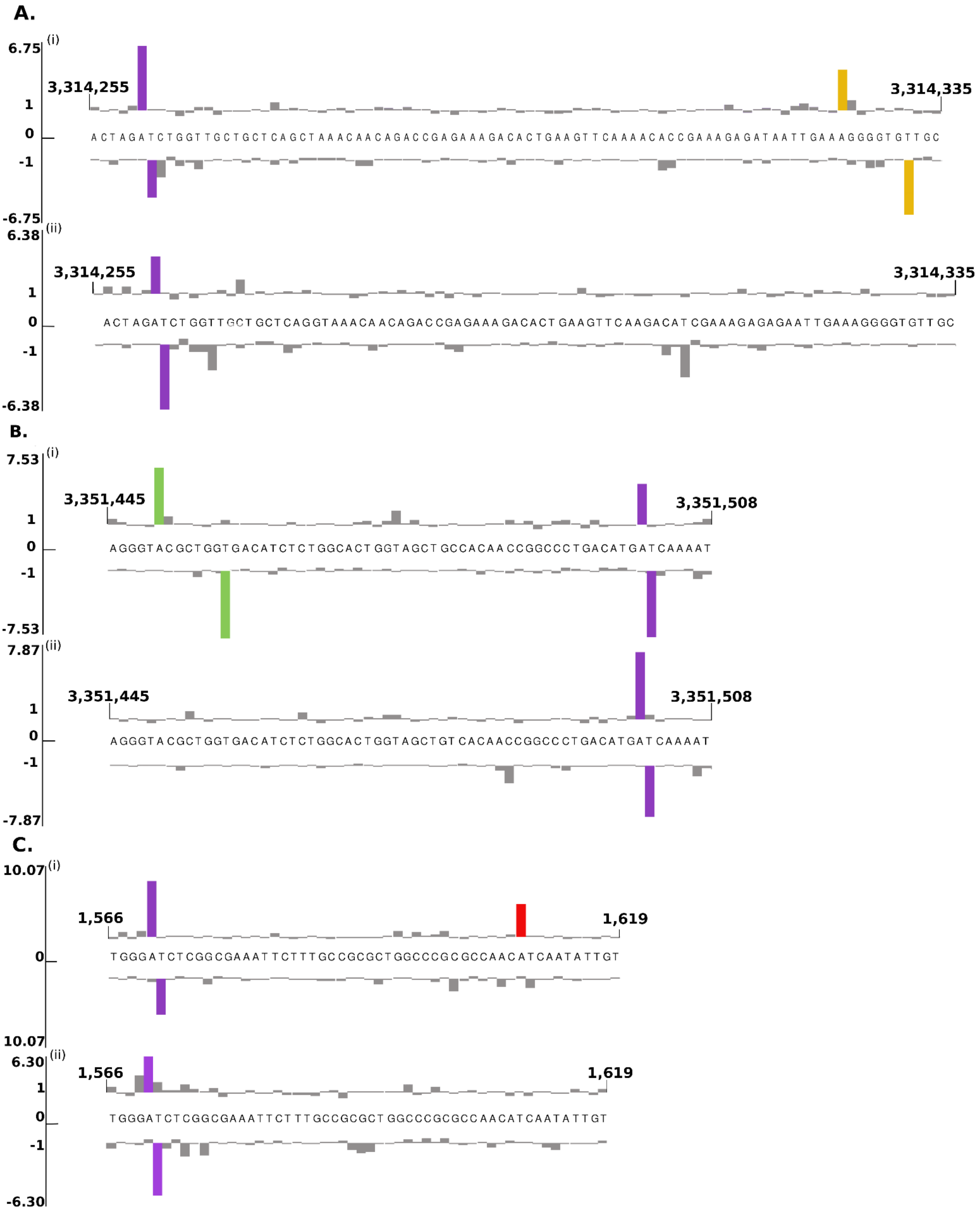

Supplementary Figure S1. Representative IPD ratio plots of RM.EcoMII (A) RM.EcoMIII (B) and RM.EcoMVII (C) recognition motifs. Each plot shows a subsection of the *E. coli* EC958 genome that contains one of the afore-mentioned novel R-M recognition sites and a Dam site as a control. The wild-type *E. coli* EC958 IPD ratio plots (top) show that under normal conditions the 5’-AACN$_4$CT$\underline{\text{T}}$T-3’ motif (A), 5’-

RTACN$_4$GTG-3’ motif (B) and 5’-CANCATC-3’ (C) are methylated. Isogenic knockout mutants IPD ratio plots (bottom) show the absence of specific methylation and that Dam methylation is unaffected. Methylated bases are indicated by the large IPD ratios, coloured purple at Dam sites (G^{m6}ATC), yellow at M.EcoMII recognition sites (A^{m6}ACN$_4$CT$\underline{T}$T), green at M.EcoMII recognition sites (RT^{m6}ACN$_4$GTG), orange at M.EcoMVII sites (CANC^{m6}ATC).
