# Supplementary material for: Lineage-Specific Methyltransferases Define the Methylome of the Globally Disseminated Escherichia coli ST131 Clone
Source: mBio. 2015 Nov 17;6(6):e01602-15. doi: 10.1128/mBio.01602-15 (PMC4659465; doi:10.1128/mBio.01602-15)
Supplement: Table S1 — Summary of recognition motifs identified in E. coli EC958. [file mbo005152543st1.pdf]

Table S1: Summary of recognition motifs identified in *E. coli* EC958

Motif String	Modification Type	Methylated (%)	Number Detected	Number in Genome	Mean Score (Qmod)	Mean IPD Ratio
Chromosome (EC958)						
GATC[1]	m6A	99.7	40820	40908	106.43927	5.6080165
CANCATC[2]	m6A	99.5	6528	6560	100.188416	5.783468
AACN_4CTTT[3]/ AAAGN_4GTT	m6A	99.8/ 99.6	846/ 844	847	99.80969/ 100.438385	6.237814/ 8.717596
RTACN_4GTG[4]/ CACN_4GTAY	m6A	99.5/ 99.5	703/ 703	706	104.12945/ 102.12233	6.7873273/ 6.5172844
GAGACC[5]	m6A	100	378	378	99.89418	5.773281
Plasmid (pEC958A)						
GATC	m6A	100	996	996	140.74197	5.6568093
CANCATC	m6A	100	164	164	133.0061	5.878233
GAGACC	m6A	100	23	23	115.608696	5.5165224
RTACN_4GTG CACN_4GTAY	m6A	100/ 100	19/ 19	19	158.3158/ 142.94737	6.87421/ 6.4668417
AAAGN_4GTT/ AACN_4CTTT	m6A	95/ 95	19/ 19	20	125.1579/ 125.10526	8.6863165/ 6.2889476

[1] GATC is under-represented in most MGEs when compared to the rest of the genome; genomic islands GI-*pheV* (P < 0.0001) and GI-*selC* (P < 0.0001), prophages Phi1-Phi7 (P ≤ 0.0001) and Cryptic Phage (P = 0.00026).

[2] No significant differences in CANCATC distribution were observed between MGEs and the rest of the genome.

[3] GAGACC is over-represented in most MGEs when compared to the rest of the genome; for example, prophage Phi4 (P = 0.002221), Cryptic Phage (P = 0.037258), and genomic islands GI-*pheV* (P = 0.000248), GI-*selC* (P = 0.001511) and GI-*leuX* (P = 0.008542). In prophage Phi1 the GAGACC motif is under-represented (P =< 0.0001).

[4] AACN_4CTTT is under-represented in the High Pathogenicity Island (P <0.0001) and GI-*leuX* (P = 0.0379).

[5] RTACN_4GTG is under-represented in Phi1 (P = <0.0001).
