# Supplementary material for: Lineage-Specific Methyltransferases Define the Methylome of the Globally Disseminated Escherichia coli ST131 Clone
Source: mBio. 2015 Nov 17;6(6):e01602-15. doi: 10.1128/mBio.01602-15 (PMC4659465; doi:10.1128/mBio.01602-15)
Supplement: Table S2 — ST131 accessory MTases. [file mbo005152543st2.pdf]

Table S2: ST131 accessory MTases

NAME	REBASE homolog	Type	Modification	Motif	ST131 specific	Clade	ST131 Strains (NT%)#
MTaseA1	M.Eco29KI	Type II	m5C	CCGCGG	Yes	C	S133(100%), S109(100%)
MTaseA2	M.EcoDEC4CORF2749P	Type II	m5C	GTCGAC	Yes	C	S115(100%), HVM1299(100%) HVM3017(100%), S119(100%),S118(100%)
MTaseA3	M.Eco248534P	Type II	m5C	GTCGAC	No	NA	, IR65 (100%),IR68(100%), IR49(100%), S77(100%),HVM1299(100%), HVM3017(100%), S119(100%),S118(100%), S65(100%)
MTaseA4	M.EcoAPECORF2077P	Type I	m6A	UD*	No	NA	NA
MTaseA5	M.EcoDEC13EORF3046 P	Type II	m5C	UD	Yes	B	S22(100%), S24(100%),HVM1147(100%)
MTaseA6	M.EcoDEC2CORF2043P	Type II	m5C	UD	Yes	C	S113(100%), S97(100%), S129(97.3%),S124(97.3%), S77(99.9%), S134(97.2%)
MTaseA7	M.Eco1886ORF14455P	Type II	UD	UD	No	NA	S128(99.1%), S79(99.1%) HVM277(99.2%), S1(100%), S11(100%), S93(100%), S129(99.9%), S124(99.9%), IR65(99.8%), IR68(99.8%), IR49(99.8%), IR18(99.1%), HVM1299(99.1%), HVM3017(99.1%), S96(99.3%), HVM834(99.8%)
MTaseA8	M.Eco1886ORF14565P	Type II	UD	UD	Yes	C	P146(100%), S1(100%), S11(100%), S93(100%)
MTaseA9	M.Eco605ORFMP	Type II	UD	UD	Yes	B,C	HVM52(99.4%), S117(97.4%), HVM826(96.5%), S115(95.6%), IR18(95.6%), HVM1299(95.6%), HVM3017(95.6%), S119(95.6%), S118(95.6%), P146(99.8%), S11(100%), S109(99.5%), S39(100%)
MTaseA10	M.Eco84137ORF201P	Type I	m6A	UD	Yes	C	S111(100%), S121(100%), S123(100%), S126(100%), S132(100%)
MTaseA11	M.Eco1520ORF67P	Type I	m6A	UD	No	NA	P112, S120(99.9%), HVM52(99.9%), HVM2044(95.3%), HVM2289(95.3%), S100(99.9%), S110(99.9%), S112(99.9%), P189(99.9%) S92(99.9%), MS2493(99.9%), HVM826(99.9%), P53(99.9%), S108(99.9%), P146(99.8%), P56(99.9%), S95(99.9%), HVR2496(99.9%), HVM5(99.9%), HVM3189(99.9%), P50(99.9%), HVR83(99.9%), S98(1005), S122(100%), S133(100%), S129(100%), S124(100%), IR65(100%), IR68(100%), IR49(100%), S65(99.9%), S103(99.9%)
MTaseA12	M.Eco15ORF4165P	Type II	m5C	UD	Yes	A	SE15, S2(100%), S26(100%), S31(100%), S5(100%), S94(100%), S120(100%), S34(100%)

*UD = undetermined

Nucleotide sequence identity to REBASE is displayed in brackets. Only matches with >=95% nucleotide sequence identity and 100% query coverage are reported.
