# Supplementary material for: Lineage-Specific Methyltransferases Define the Methylome of the Globally Disseminated Escherichia coli ST131 Clone
Source: mBio. 2015 Nov 17;6(6):e01602-15. doi: 10.1128/mBio.01602-15 (PMC4659465; doi:10.1128/mBio.01602-15)
Supplement: Table S3 — BLAST result summary for E. coli ST131 genomes versus REBASE protein sequences. [file mbo005152543st3.pdf]

Table S3: BLAST result summary for E. coli ST131 genomes versus REBASE protein sequences.

SPECIES (S)	NODE	LENGTH	MTASE (Q)	QUERY LENGTH	ALIGNMENT LENGTH	IDENTITY	Q. START	Q. STOP	S. START	S. STOP	EVALUE	SCORE
S2EC	115	34383	M1.EcoMI	1623	1623	100	1	1623	3797	5419	0	3009
S2EC	115	34383	M1.EcoMI	1191	1191	100	1	1191	5412	6602	0	2361
S2EC	21	345475	M.EcoMII	1620	19	100	1333	1351	155385	155403	0.026	38.2
S2EC	21	345475	M.EcoMII	1620	17	100	273	289	197083	197099	0.4	34.2
S2EC	21	345475	M.EcoMII	1620	20	95	1287	1306	73141	73160	1.6	32.2
S2EC	21	345475	M.EcoMII	1620	20	95	381	400	260318	260299	1.6	32.2
S2EC	21	345475	M.EcoMII	1620	15	100	1416	1430	318625	318611	6.3	30.2
S2EC	207	73347	M.EcoMIII	1638	1638	100	1	1638	38550	40187	0	3247
S2EC	207	73347	M.EcoMIII	1638	15	100	992	1006	43263	43277	6.3	30.2
S2EC	22	112141	M.EcoMIV	765	17	100	332	348	77393	77409	0.19	34.2
S2EC	22	112141	M.EcoMIV	765	15	100	630	644	20805	20791	2.9	30.2
S2EC	22	112141	M.EcoMIV	765	15	100	345	359	88443	88429	2.9	30.2
S2EC	32	521706	M.EcoMV	1050	18	100	976	993	131620	131603	0.065	36.2
S2EC	32	521706	M.EcoMV	1050	22	95.45	690	711	424395	424374	0.065	36.2
S2EC	32	521706	M.EcoMV	1050	16	100	781	796	198563	198578	1	32.2
S2EC	32	521706	M.EcoMV	1050	16	100	115	130	499630	499615	1	32.2
S2EC	32	521706	M.EcoMV	1050	15	100	685	699	393826	393812	4	30.2
S2EC	39	452171	M.EcoNwEVDcm	1419	1419	98.94	1	1419	45800	47218	0	2694
S2EC	39	452171	M.EcoNwEVDcm	1419	15	100	580	594	207303	207289	5.5	30.2
S2EC	21	345475	M.EcoMVI	891	891	100	1	891	344529	345419	0	1725
S2EC	21	345475	M.EcoMVI	891	16	100	232	247	152095	152110	0.86	32.2
S2EC	21	345475	M.EcoMVI	891	15	100	608	622	98106	98092	3.4	30.2
S2EC	14	171427	M.EcoNwEDam	837	837	100	1	837	39445	38609	0	1659
S2EC	32	521706	M.EcoMVII	3606	25	96	596	620	123505	123481	0.004	42.1
S2EC	32	521706	M.EcoMVII	3606	16	100	201	216	130320	130305	3.5	32.2
S2EC	32	521706	M.EcoMVII	3606	16	100	3500	3515	469548	469563	3.5	32.2
S2EC	35	39555	M.EcoMVIII	684	684	100	1	684	32340	33023	0	1356
S2EC	24	70188	Eco29kI	645	16	100	238	253	46021	46036	0.62	32.2
S2EC	24	70188	Eco29kI	645	18	94.44	210	227	17734	17717	9.7	28.2
S2EC	215	166980	EcoDEC4CORF2749P	1041	24	91.67	957	980	159834	159811	1	32.2
S2EC	53	100937	Eco248534P	1053	16	100	941	956	31126	31141	1	32.2
S2EC	53	100937	Eco248534P	1053	15	100	66	80	27236	27250	4	30.2

S2EC	73	46523	EcoAPECORF2077P	1590	18	100	1494	1511	13312	13295	0.099	36.2
S2EC	215	166980	EcoDEC13EORF3046P	1191	16	100	1050	1065	127300	127315	1.2	32.2
S2EC	215	166980	EcoDEC13EORF3046P	1191	15	100	156	170	99764	99750	4.6	30.2
S2EC	35	39555	Eco7A8ORF29P	684	606	98.51	79	684	32418	33023	0	1130
S2EC	32	521706	EcoDEC2CORF2043P	2019	21	95.24	1522	1542	344258	344278	0.5	34.2
S2EC	32	521706	EcoDEC2CORF2043P	2019	16	100	910	925	366387	366402	2	32.2
S2EC	32	521706	EcoDEC2CORF2043P	2019	15	100	459	473	17075	17089	7.8	30.2
S2EC	32	521706	EcoDEC2CORF2043P	2019	19	94.74	1813	1831	40356	40338	7.8	30.2
S2EC	133	19954	Eco1886ORF14455P	1053	16	100	579	594	13207	13192	1	32.2
S2EC	68	10018	Eco1886ORF14565P	900	15	100	584	598	8674	8660	3.4	30.2
S2EC	35	39555	EcoR7ACORFAP	684	684	97.37	1	684	32340	33023	0	1213
S2EC	35	39555	EcoR100ORF1P	648	606	98.51	43	648	32418	33023	0	1130
S2EC	35	39555	EcoR100ORF1P	648	18	94.44	312	329	36456	36473	9.7	28.2
S2EC	35	39555	Eco605ORFMP	684	684	93.86	1	684	32340	33023	0	1023
S2EC	39	452171	Eco84137ORF201P	1635	22	95.45	558	579	20487	20508	0.1	36.2
S2EC	39	452171	Eco84137ORF201P	1635	16	100	1361	1376	86749	86764	1.6	32.2
S2EC	39	452171	Eco84137ORF201P	1635	15	100	1056	1070	366242	366228	6.3	30.2
S2EC	39	452171	Eco84137ORF201P	1635	15	100	1096	1110	383281	383267	6.3	30.2
S2EC	39	452171	Eco84137ORF201P	1635	15	100	590	604	417187	417173	6.3	30.2
S2EC	42	56100	Eco1520ORF67P	1563	18	100	1532	1549	16454	16437	0.098	36.2
S2EC	215	166980	Eco15ORF4165P	1197	1197	100	1	1197	160952	159756	0	2278
S2EC	215	166980	Eco15ORF4165P	1197	19	94.74	72	90	12388	12406	4.6	30.2
S26EC	57	33533	M1.EcoMI	1623	1623	100	1	1623	2948	4570	0	3009
S26EC	57	33533	M1.EcoMI	1191	1191	100	1	1191	4563	5753	0	2361
S26EC	246	224740	M.EcoMII	1620	19	100	1333	1351	201055	201037	0.025	38.2
S26EC	246	224740	M.EcoMII	1620	17	100	273	289	159357	159341	0.4	34.2
S26EC	246	224740	M.EcoMII	1620	20	95	381	400	96122	96141	1.6	32.2
S26EC	246	224740	M.EcoMII	1620	15	100	1416	1430	37815	37829	6.2	30.2
S26EC	26	197012	M.EcoMIII	1638	1638	100	1	1638	162348	163985	0	3247
S26EC	26	197012	M.EcoMIII	1638	15	100	739	753	92560	92574	6.3	30.2
S26EC	26	197012	M.EcoMIII	1638	15	100	992	1006	167061	167075	6.3	30.2
S26EC	246	224740	M.EcoMIV	765	17	100	65	81	45212	45228	0.19	34.2
S26EC	246	224740	M.EcoMV	1050	18	100	628	645	118361	118378	0.065	36.2
S26EC	246	224740	M.EcoMV	1050	15	100	576	590	127058	127072	4	30.2
S26EC	246	224740	M.EcoMV	1050	15	100	165	179	222389	222403	4	30.2
S26EC	9	452081	M.EcoNwEVDcm	1419	1419	98.94	1	1419	45809	47227	0	2694

S26EC	9	452081	M.EcoNwEVDcm	1419	15	100	580	594	207312	207298	5.4	30.2
S26EC	246	224740	M.EcoMVI	891	891	100	1	891	11911	11021	0	1725
S26EC	246	224740	M.EcoMVI	891	16	100	232	247	204345	204330	0.86	32.2
S26EC	37	190078	M.EcoNwEDam	837	837	100	1	837	39453	38617	0	1659
S26EC	216	378712	M.EcoMVII	3606	25	96	596	620	255269	255293	0.004	42.1
S26EC	216	378712	M.EcoMVII	3606	16	100	201	216	248454	248469	3.5	32.2
S26EC	67	17723	M.EcoMVIII	684	684	100	1	684	10526	11209	0	1356
S26EC	27	45959	Eco29kI	645	16	100	238	253	210	195	0.62	32.2
S26EC	27	45959	Eco29kI	645	18	94.44	210	227	28497	28514	9.6	28.2
S26EC	229	72843	EcoDEC4CORF2749P	1041	16	100	643	658	27652	27667	1	32.2
S26EC	47	21896	Eco248534P	1053	16	100	262	277	5726	5741	1	32.2
S26EC	246	224740	EcoAPECORF2077P	1590	18	100	1511	1528	98197	98214	0.099	36.2
S26EC	246	224740	EcoAPECORF2077P	1590	17	100	1550	1566	45487	45503	0.39	34.2
S26EC	246	224740	EcoAPECORF2077P	1590	15	100	50	64	9330	9344	6.1	30.2
S26EC	246	224740	EcoAPECORF2077P	1590	19	94.74	1493	1511	71619	71637	6.1	30.2
S26EC	246	224740	EcoAPECORF2077P	1590	15	100	1552	1566	173916	173930	6.1	30.2
S26EC	115	167425	EcoDEC13EORF3046P	1191	16	100	8	23	7758	7773	1.2	32.2
S26EC	115	167425	EcoDEC13EORF3046P	1191	16	100	192	207	167045	167060	1.2	32.2
S26EC	67	17723	Eco7A8ORF29P	684	606	98.51	79	684	10604	11209	0	1130
S26EC	216	378712	EcoDEC2CORF2043P	2019	21	95.24	1522	1542	34515	34495	0.5	34.2
S26EC	216	378712	EcoDEC2CORF2043P	2019	19	94.74	1813	1831	338417	338435	7.7	30.2
S26EC	216	378712	EcoDEC2CORF2043P	2019	15	100	459	473	361698	361684	7.7	30.2
S26EC	37	190078	Eco1886ORF14455P	1053	16	100	579	594	178240	178255	1	32.2
S26EC	216	378712	Eco1886ORF14565P	900	15	100	565	579	175318	175304	3.4	30.2
S26EC	67	17723	EcoR7ACORFAP	684	684	97.37	1	684	10526	11209	0	1213
S26EC	67	17723	EcoR100ORF1P	648	606	98.51	43	648	10604	11209	0	1130
S26EC	67	17723	EcoR100ORF1P	648	18	94.44	312	329	14642	14659	9.7	28.2
S26EC	67	17723	Eco605ORFMP	684	684	93.86	1	684	10526	11209	0	1023
S26EC	216	378712	Eco84137ORF201P	1635	18	100	43	60	273641	273658	0.1	36.2
S26EC	216	378712	Eco84137ORF201P	1635	16	100	1212	1227	274928	274913	1.6	32.2
S26EC	216	378712	Eco84137ORF201P	1635	15	100	1080	1094	371562	371548	6.3	30.2
S26EC	53	246434	Eco1520ORF67P	1563	18	100	1501	1518	15583	15566	0.097	36.2
S26EC	53	246434	Eco1520ORF67P	1563	15	100	1239	1253	48141	48155	6	30.2
S26EC	20	96288	Eco15ORF4165P	1197	1197	100	1	1197	90250	89054	0	2278
S37EC	487	5211	M1.EcoMI	1623	1623	100	1	1623	2332	710	0	3009
S37EC	487	5211	M1.EcoMI	1191	717	100	1	717	717	1	0	1421

S37EC	131	14637	M.EcoMII	1620	19	100	1333	1351	5617	5599	0.027	38.2
S37EC	387	13971	M.EcoMIII	1638	1202	100	1	1202	12842	14043	0	2383
S37EC	17	8349	M.EcoMIV	765	17	100	332	348	6260	6276	0.2	34.2
S37EC	361	20253	M.EcoMV	1050	18	100	628	645	14713	14730	0.068	36.2
S37EC	22	58372	M.EcoNwEVDcm	1419	1419	98.94	1	1419	16289	17707	0	2694
S37EC	248	18847	M.EcoMVI	891	891	100	1	891	11925	11035	0	1725
S37EC	176	14469	M.EcoNwEDam	837	837	100	1	837	1636	2472	0	1659
S37EC	898	15800	M.EcoMVII	3606	3606	100	1	3606	9744	6139	0	6988
S37EC	867	5270	M.EcoMVIII	684	684	100	1	684	3983	3300	0	1356
S37EC	165	22712	Eco29kI	645	16	100	238	253	13009	13024	0.65	32.2
S37EC	539	2720	EcoDEC4CORF2749P	1041	16	100	643	658	352	367	1.1	32.2
S37EC	1	24004	Eco248534P	1053	16	100	941	956	15651	15666	1.1	32.2
S37EC	1	24004	Eco248534P	1053	15	100	66	80	11761	11775	4.2	30.2
S37EC	296	15780	EcoAPECORF2077P	1590	18	100	1260	1277	3882	3865	0.1	36.2
S37EC	844	704	EcoDEC13EORF3046P	1191	16	100	192	207	463	448	1.2	32.2
S37EC	867	5270	Eco7A8ORF29P	684	606	98.51	79	684	3905	3300	0	1130
S37EC	728	1146	EcoDEC2CORF2043P	2019	21	95.24	1522	1542	1163	1183	0.52	34.2
S37EC	514	3072	Eco1886ORF14455P	1053	16	100	958	973	1267	1282	1.1	32.2
S37EC	543	11669	Eco1886ORF14565P	900	15	100	74	88	9203	9189	3.6	30.2
S37EC	867	5270	EcoR7ACORFAP	684	684	97.37	1	684	3983	3300	0	1213
S37EC	867	5270	EcoR100ORF1P	648	606	98.51	43	648	3905	3300	0	1130
S37EC	867	5270	Eco605ORFMP	684	684	93.86	1	684	3983	3300	0	1023
S37EC	322	8776	Eco84137ORF201P	1635	22	95.45	558	579	5055	5076	0.11	36.2
S37EC	530	11268	Eco1520ORF67P	1563	18	100	1532	1549	7933	7950	0.1	36.2
S37EC	389	6691	Eco15ORF4165P	1197	1015	100	1	1015	5759	6773	0	1917
S31EC	32	34403	M1.EcoMI	1623	1623	100	1	1623	3817	5439	0	3009
S31EC	32	34403	M1.EcoMI	1191	1191	100	1	1191	5432	6622	0	2361
S31EC	17	91811	M.EcoMII	1620	19	100	1333	1351	18209	18227	0.026	38.2
S31EC	17	91811	M.EcoMII	1620	17	100	273	289	59907	59923	0.41	34.2
S31EC	23	73047	M.EcoMIII	1638	1638	100	1	1638	23602	21965	0	3247
S31EC	23	73047	M.EcoMIII	1638	15	100	992	1006	18889	18875	6.4	30.2
S31EC	81	29832	M.EcoMIV	765	17	100	65	81	19262	19246	0.19	34.2
S31EC	207	35718	M.EcoMV	1050	18	100	976	993	35442	35425	0.066	36.2
S31EC	99	13113	M.EcoNwEVDcm	1419	1419	98.94	1	1419	1652	3070	0	2694
S31EC	69	34567	M.EcoMVI	891	891	100	1	891	11923	11033	0	1725
S31EC	19	44807	M.EcoNwEDam	837	837	100	1	837	14972	15808	0	1659

S31EC	207	35718	M.EcoMVII	3606	25	96	596	620	27327	27303	0.004	42.1
S31EC	207	35718	M.EcoMVII	3606	16	100	201	216	34142	34127	3.6	32.2
S31EC	41	3958	M.EcoMVIII	684	684	100	1	684	2205	1522	0	1356
S31EC	13	50381	Eco29kI	645	16	100	238	253	31807	31822	0.63	32.2
S31EC	13	50381	Eco29kI	645	18	94.44	210	227	3520	3503	9.9	28.2
S31EC	88	29317	EcoDEC4CORF2749P	1041	24	91.67	957	980	7232	7255	1	32.2
S31EC	186	7489	Eco248534P	1053	16	100	262	277	5756	5771	1	32.2
S31EC	115	29130	EcoAPECORF2077P	1590	18	100	1511	1528	6470	6487	0.1	36.2
S31EC	218	65398	EcoDEC13EORF3046P	1191	16	100	8	23	54312	54327	1.2	32.2
S31EC	41	3958	Eco7A8ORF29P	684	606	98.51	79	684	2127	1522	0	1130
S31EC	142	14085	EcoDEC2CORF2043P	2019	21	95.24	1522	1542	13344	13364	0.51	34.2
S31EC	288	5218	Eco1886ORF14455P	1053	16	100	124	139	1580	1565	1	32.2
S31EC	178	31177	Eco1886ORF14565P	900	15	100	50	64	24604	24590	3.5	30.2
S31EC	41	3958	EcoR7ACORFAP	684	684	97.37	1	684	2205	1522	0	1213
S31EC	41	3958	EcoR100ORF1P	648	606	98.51	43	648	2127	1522	0	1130
S31EC	41	3958	Eco605ORFMP	684	684	93.86	1	684	2205	1522	0	1023
S31EC	251	3649	Eco84137ORF201P	1635	22	95.45	558	579	1061	1040	0.1	36.2
S31EC	450	41096	Eco1520ORF67P	1563	18	100	1532	1549	11488	11505	0.099	36.2
S31EC	88	29317	Eco15ORF4165P	1197	1197	100	1	1197	6114	7310	0	2278
S5EC	17	37392	M1.EcoMI	1623	1623	100	1	1623	6807	8429	0	3009
S5EC	17	37392	M1.EcoMI	1191	1191	100	1	1191	8422	9612	0	2361
S5EC	133	365769	M.EcoMII	1620	19	100	1333	1351	201061	201043	0.026	38.2
S5EC	133	365769	M.EcoMII	1620	17	100	273	289	159363	159347	0.41	34.2
S5EC	133	365769	M.EcoMII	1620	20	95	381	400	96128	96147	1.6	32.2
S5EC	133	365769	M.EcoMII	1620	20	95	1287	1306	283538	283519	1.6	32.2
S5EC	133	365769	M.EcoMII	1620	15	100	1416	1430	37821	37835	6.4	30.2
S5EC	14	269787	M.EcoMIII	1638	1638	100	1	1638	235123	236760	0	3247
S5EC	14	269787	M.EcoMIII	1638	16	100	1336	1351	32091	32076	1.6	32.2
S5EC	14	269787	M.EcoMIII	1638	15	100	1509	1523	64901	64887	6.5	30.2
S5EC	14	269787	M.EcoMIII	1638	15	100	739	753	165335	165349	6.5	30.2
S5EC	14	269787	M.EcoMIII	1638	15	100	992	1006	239836	239850	6.5	30.2
S5EC	133	365769	M.EcoMIV	765	17	100	65	81	45218	45234	0.19	34.2
S5EC	133	365769	M.EcoMV	1050	18	100	628	645	118367	118384	0.067	36.2
S5EC	133	365769	M.EcoMV	1050	16	100	148	163	262546	262561	1	32.2
S5EC	133	365769	M.EcoMV	1050	15	100	576	590	127064	127078	4.1	30.2
S5EC	133	365769	M.EcoMV	1050	15	100	165	179	222395	222409	4.1	30.2

S5EC	133	365769	M.EcoMV	1050	15	100	671	685	332385	332399	4.1	30.2
S5EC	10	461045	M.EcoNwEVDcm	1419	1419	98.94	1	1419	54763	56181	0	2694
S5EC	10	461045	M.EcoNwEVDcm	1419	15	100	580	594	216266	216252	5.6	30.2
S5EC	133	365769	M.EcoMVI	891	891	100	1	891	11917	11027	0	1725
S5EC	133	365769	M.EcoMVI	891	16	100	232	247	204351	204336	0.89	32.2
S5EC	123	191392	M.EcoNwEDam	837	837	100	1	837	152008	152844	0	1659
S5EC	23	15476	M.EcoMVII	3606	3606	100	1	3606	9420	5815	0	6988
S5EC	84	55512	M.EcoMVIII	684	684	89.91	1	684	1360	2043	0	809
S5EC	63	105229	Eco29kI	645	16	100	238	253	59269	59254	0.64	32.2
S5EC	63	105229	Eco29kI	645	18	94.44	210	227	87556	87573	10	28.2
S5EC	79	162466	EcoDEC4CORF2749P	1041	24	91.67	957	980	7231	7254	1	32.2
S5EC	28	17434	Eco248534P	1053	16	100	262	277	5750	5765	1.1	32.2
S5EC	133	365769	EcoAPECORF2077P	1590	18	100	1511	1528	98203	98220	0.1	36.2
S5EC	133	365769	EcoAPECORF2077P	1590	17	100	1550	1566	45493	45509	0.4	34.2
S5EC	133	365769	EcoAPECORF2077P	1590	17	100	2	18	226900	226884	0.4	34.2
S5EC	133	365769	EcoAPECORF2077P	1590	16	100	690	705	333419	333434	1.6	32.2
S5EC	133	365769	EcoAPECORF2077P	1590	15	100	50	64	9336	9350	6.3	30.2
S5EC	133	365769	EcoAPECORF2077P	1590	19	94.74	1493	1511	71625	71643	6.3	30.2
S5EC	133	365769	EcoAPECORF2077P	1590	15	100	1552	1566	173922	173936	6.3	30.2
S5EC	133	365769	EcoAPECORF2077P	1590	15	100	811	825	290684	290698	6.3	30.2
S5EC	79	162466	EcoDEC13EORF3046P	1191	16	100	1050	1065	39765	39750	1.2	32.2
S5EC	79	162466	EcoDEC13EORF3046P	1191	15	100	156	170	67301	67315	4.7	30.2
S5EC	84	55512	Eco7A8ORF29P	684	621	88.89	64	684	1423	2043	0	684
S5EC	40	500290	EcoDEC2CORF2043P	2019	21	95.24	1522	1542	133858	133838	0.51	34.2
S5EC	40	500290	EcoDEC2CORF2043P	2019	15	100	1943	1957	91624	91638	8	30.2
S5EC	40	500290	EcoDEC2CORF2043P	2019	19	94.74	1813	1831	437763	437781	8	30.2
S5EC	40	500290	EcoDEC2CORF2043P	2019	15	100	459	473	461044	461030	8	30.2
S5EC	133	365769	Eco1886ORF14455P	1053	16	100	958	973	266073	266088	1.1	32.2
S5EC	176	84061	Eco1886ORF14565P	900	15	100	289	303	49887	49901	3.5	30.2
S5EC	176	84061	Eco1886ORF14565P	900	15	100	273	287	69073	69059	3.5	30.2
S5EC	84	55512	EcoR7ACORFAP	684	684	89.47	1	684	1360	2043	0	785
S5EC	84	55512	EcoR7ACORFAP	684	15	100	182	196	37334	37348	2.7	30.2
S5EC	84	55512	EcoR100ORF1P	648	621	88.89	28	648	1423	2043	0	684
S5EC	84	55512	Eco605ORFMP	684	684	89.62	1	684	1360	2043	0	793
S5EC	160	12738	Eco84137ORF201P	1635	22	95.45	558	579	5468	5489	0.11	36.2
S5EC	266	180805	Eco1520ORF67P	1563	18	100	1532	1549	141047	141030	0.1	36.2

S5EC	79	162466	Eco15ORF4165P	1197	1197	100	1	1197	6113	7309	0	2278
S5EC	79	162466	Eco15ORF4165P	1197	19	94.74	72	90	154677	154659	4.7	30.2
S94EC	139	34532	M1.EcoMI	1623	1623	100	1	1623	30649	29027	0	3009
S94EC	139	34532	M1.EcoMI	1191	1191	100	1	1191	29034	27844	0	2361
S94EC	219	239539	M.EcoMII	1620	19	100	1333	1351	165940	165958	0.026	38.2
S94EC	219	239539	M.EcoMII	1620	17	100	273	289	207638	207654	0.41	34.2
S94EC	219	239539	M.EcoMII	1620	20	95	1287	1306	83463	83482	1.6	32.2
S94EC	256	269774	M.EcoMIII	1638	1638	100	1	1638	235110	236747	0	3247
S94EC	256	269774	M.EcoMIII	1638	16	100	1336	1351	32079	32064	1.6	32.2
S94EC	256	269774	M.EcoMIII	1638	15	100	1509	1523	64889	64875	6.5	30.2
S94EC	256	269774	M.EcoMIII	1638	15	100	739	753	165322	165336	6.5	30.2
S94EC	256	269774	M.EcoMIII	1638	15	100	992	1006	239823	239837	6.5	30.2
S94EC	39	158829	M.EcoMIV	765	17	100	332	348	123852	123868	0.19	34.2
S94EC	39	158829	M.EcoMIV	765	15	100	630	644	67264	67250	3	30.2
S94EC	39	158829	M.EcoMIV	765	15	100	345	359	134902	134888	3	30.2
S94EC	162	500364	M.EcoMV	1050	18	100	976	993	368805	368822	0.067	36.2
S94EC	162	500364	M.EcoMV	1050	16	100	190	205	69147	69162	1.1	32.2
S94EC	162	500364	M.EcoMV	1050	16	100	781	796	301862	301847	1.1	32.2
S94EC	162	500364	M.EcoMV	1050	15	100	534	548	29457	29471	4.2	30.2
S94EC	162	500364	M.EcoMV	1050	15	100	784	798	74839	74853	4.2	30.2
S94EC	31	486812	M.EcoNwEVDcm	1419	1419	98.94	1	1419	45802	47220	0	2694
S94EC	31	486812	M.EcoNwEVDcm	1419	15	100	580	594	207305	207291	5.6	30.2
S94EC	31	486812	M.EcoNwEVDcm	1419	15	100	756	770	295913	295899	5.6	30.2
S94EC	24	91668	M.EcoMVI	891	891	100	1	891	11905	11015	0	1725
S94EC	33	191380	M.EcoNwEDam	837	837	100	1	837	39447	38611	0	1659
S94EC	145	15464	M.EcoMVII	3606	3606	100	1	3606	6119	9724	0	6988
S94EC	15	48400	M.EcoMVIII	684	684	99.71	1	684	46457	45774	0	1340
S94EC	153	105672	Eco29kI	645	16	100	238	253	46023	46038	0.64	32.2
S94EC	153	105672	Eco29kI	645	18	94.44	210	227	17736	17719	10	28.2
S94EC	256	269774	EcoDEC4CORF2749P	1041	16	100	643	658	45254	45239	1	32.2
S94EC	55	17280	Eco248534P	1053	16	100	262	277	11548	11533	1.1	32.2
S94EC	162	500364	EcoAPECORF2077P	1590	18	100	1260	1277	27178	27195	0.1	36.2
S94EC	162	500364	EcoAPECORF2077P	1590	17	100	99	115	6188	6172	0.4	34.2
S94EC	162	500364	EcoAPECORF2077P	1590	17	100	1	17	31540	31524	0.4	34.2
S94EC	162	500364	EcoAPECORF2077P	1590	17	100	930	946	361914	361930	0.4	34.2
S94EC	162	500364	EcoAPECORF2077P	1590	15	100	669	683	72023	72009	6.3	30.2

S94EC	162	500364	EcoAPECORF2077P	1590	15	100	1401	1415	191325	191311	6.3	30.2
S94EC	162	500364	EcoAPECORF2077P	1590	15	100	1294	1308	208731	208745	6.3	30.2
S94EC	162	500364	EcoAPECORF2077P	1590	15	100	1436	1450	275697	275711	6.3	30.2
S94EC	61	176020	EcoDEC13EORF3046P	1191	16	100	1050	1065	136480	136495	1.2	32.2
S94EC	61	176020	EcoDEC13EORF3046P	1191	15	100	156	170	108944	108930	4.7	30.2
S94EC	15	48400	Eco7A8ORF29P	684	606	98.51	79	684	46379	45774	0	1130
S94EC	162	500364	EcoDEC2CORF2043P	2019	21	95.24	1522	1542	156166	156146	0.52	34.2
S94EC	162	500364	EcoDEC2CORF2043P	2019	16	100	124	139	20017	20032	2	32.2
S94EC	162	500364	EcoDEC2CORF2043P	2019	15	100	1943	1957	113932	113946	8	30.2
S94EC	162	500364	EcoDEC2CORF2043P	2019	19	94.74	1813	1831	460069	460087	8	30.2
S94EC	162	500364	EcoDEC2CORF2043P	2019	15	100	459	473	483350	483336	8	30.2
S94EC	219	239539	Eco1886ORF14455P	1053	16	100	958	973	100928	100913	1.1	32.2
S94EC	225	231198	Eco1886ORF14565P	900	15	100	289	303	41339	41353	3.6	30.2
S94EC	225	231198	Eco1886ORF14565P	900	15	100	273	287	60523	60509	3.6	30.2
S94EC	15	48400	EcoR7ACORFAP	684	684	97.37	1	684	46457	45774	0	1213
S94EC	15	48400	EcoR100ORF1P	648	606	98.51	43	648	46379	45774	0	1130
S94EC	15	48400	Eco605ORFMP	684	684	93.86	1	684	46457	45774	0	1023
S94EC	162	500364	Eco84137ORF201P	1635	18	100	43	60	395293	395310	0.11	36.2
S94EC	162	500364	Eco84137ORF201P	1635	16	100	1212	1227	396580	396565	1.6	32.2
S94EC	162	500364	Eco84137ORF201P	1635	15	100	49	63	13653	13667	6.5	30.2
S94EC	162	500364	Eco84137ORF201P	1635	15	100	1080	1094	493214	493200	6.5	30.2
S94EC	54	234870	Eco1520ORF67P	1563	18	100	1501	1518	15577	15560	0.1	36.2
S94EC	54	234870	Eco1520ORF67P	1563	15	100	1239	1253	48135	48149	6.2	30.2
S94EC	61	176020	Eco15ORF4165P	1197	1197	100	1	1197	170132	168936	0	2278
S94EC	61	176020	Eco15ORF4165P	1197	19	94.74	72	90	21567	21585	4.7	30.2
S120EC	27	34234	M1.EcoMI	1623	1623	100	1	1623	3648	5270	0	3009
S120EC	27	34234	M1.EcoMI	1191	1191	100	1	1191	5263	6453	0	2361
S120EC	231	248087	M.EcoMII	1620	19	100	1333	1351	70996	70978	0.026	38.2
S120EC	231	248087	M.EcoMII	1620	17	100	273	289	29298	29282	0.41	34.2
S120EC	231	248087	M.EcoMII	1620	20	95	1287	1306	153472	153453	1.6	32.2
S120EC	284	268677	M.EcoMIII	1638	1638	100	1	1638	34727	33090	0	3247
S120EC	284	268677	M.EcoMIII	1638	16	100	1336	1351	236661	236676	1.6	32.2
S120EC	284	268677	M.EcoMIII	1638	15	100	992	1006	30014	30000	6.5	30.2
S120EC	284	268677	M.EcoMIII	1638	15	100	739	753	104515	104501	6.5	30.2
S120EC	284	268677	M.EcoMIII	1638	15	100	1509	1523	203851	203865	6.5	30.2
S120EC	95	158829	M.EcoMIV	765	17	100	332	348	123852	123868	0.19	34.2

S120EC	95	158829	M.EcoMIV	765	15	100	630	644	67264	67250	3	30.2
S120EC	95	158829	M.EcoMIV	765	15	100	345	359	134902	134888	3	30.2
S120EC	217	439761	M.EcoMV	1050	22	95.45	690	711	369060	369039	0.067	36.2
S120EC	217	439761	M.EcoMV	1050	16	100	190	205	228610	228595	1	32.2
S120EC	217	439761	M.EcoMV	1050	15	100	784	798	222918	222904	4.1	30.2
S120EC	217	439761	M.EcoMV	1050	15	100	534	548	268300	268286	4.1	30.2
S120EC	217	439761	M.EcoMV	1050	15	100	685	699	338491	338477	4.1	30.2
S120EC	147	415522	M.EcoNwEVDcm	1419	1419	98.94	1	1419	25573	26991	0	2694
S120EC	147	415522	M.EcoNwEVDcm	1419	15	100	580	594	187076	187062	5.6	30.2
S120EC	18	91678	M.EcoMVI	891	891	100	1	891	11905	11015	0	1725
S120EC	264	191380	M.EcoNwEDam	837	837	100	1	837	39447	38611	0	1659
S120EC	49	15692	M.EcoMVII	3606	3606	99.92	1	3606	9636	6031	0	6964
S120EC	1	5716	M.EcoMVIII	684	684	100	1	684	1816	2499	0	1356
S120EC	25	105128	Eco29kI	645	16	100	238	253	46023	46038	0.64	32.2
S120EC	25	105128	Eco29kI	645	18	94.44	210	227	17736	17719	9.9	28.2
S120EC	284	268677	EcoDEC4CORF2749P	1041	16	100	643	658	223486	223501	1	32.2
S120EC	32	8108	Eco248534P	1053	16	100	262	277	5541	5526	1	32.2
S120EC	217	439761	EcoAPECORF2077P	1590	18	100	1260	1277	270579	270562	0.1	36.2
S120EC	217	439761	EcoAPECORF2077P	1590	17	100	1	17	266217	266233	0.4	34.2
S120EC	217	439761	EcoAPECORF2077P	1590	17	100	99	115	291569	291585	0.4	34.2
S120EC	217	439761	EcoAPECORF2077P	1590	15	100	1436	1450	22060	22046	6.3	30.2
S120EC	217	439761	EcoAPECORF2077P	1590	15	100	1294	1308	89026	89012	6.3	30.2
S120EC	217	439761	EcoAPECORF2077P	1590	15	100	1401	1415	106432	106446	6.3	30.2
S120EC	217	439761	EcoAPECORF2077P	1590	15	100	669	683	225734	225748	6.3	30.2
S120EC	217	439761	EcoAPECORF2077P	1590	15	100	1025	1039	315634	315648	6.3	30.2
S120EC	217	439761	EcoAPECORF2077P	1590	15	100	65	79	353687	353673	6.3	30.2
S120EC	217	439761	EcoAPECORF2077P	1590	19	94.74	1523	1541	436797	436815	6.3	30.2
S120EC	147	415522	EcoDEC13EORF3046P	1191	16	100	920	935	111002	110987	1.2	32.2
S120EC	147	415522	EcoDEC13EORF3046P	1191	15	100	706	720	370533	370519	4.7	30.2
S120EC	1	5716	Eco7A8ORF29P	684	606	98.51	79	684	1894	2499	0	1130
S120EC	217	439761	EcoDEC2CORF2043P	2019	21	95.24	1522	1542	141591	141611	0.51	34.2
S120EC	217	439761	EcoDEC2CORF2043P	2019	16	100	124	139	277740	277725	2	32.2
S120EC	217	439761	EcoDEC2CORF2043P	2019	16	100	910	925	311052	311067	2	32.2
S120EC	217	439761	EcoDEC2CORF2043P	2019	15	100	1943	1957	183825	183811	8	30.2
S120EC	264	191380	Eco1886ORF14455P	1053	16	100	579	594	178234	178249	1	32.2
S120EC	264	191380	Eco1886ORF14565P	900	15	100	30	44	6810	6796	3.5	30.2

S120EC	1	5716	EcoR7ACORFAP	684	684	97.37	1	684	1816	2499	0	1213
S120EC	1	5716	EcoR100ORF1P	648	606	98.51	43	648	1894	2499	0	1130
S120EC	1	5716	Eco605ORFMP	684	684	93.86	1	684	1816	2499	0	1023
S120EC	92	7572	Eco84137ORF201P	1635	27	92.59	811	837	5526	5500	0.027	38.2
S120EC	92	7572	Eco84137ORF201P	1635	20	95	550	569	5766	5747	1.6	32.2
S120EC	92	7572	Eco1520ORF67P	1563	1563	99.94	1	1563	6300	4738	0	3049
S120EC	11	144188	Eco15ORF4165P	1197	1197	100	1	1197	138150	136954	0	2278
S34EC	250	10292	M1.EcoMI	1623	1623	100	1	1623	6556	4934	0	3009
S34EC	250	10292	M1.EcoMI	1191	1191	100	1	1191	4941	3751	0	2361
S34EC	128	83909	M.EcoMII	1620	20	100	853	872	44966	44985	0.007	40.1
S34EC	114	268099	M.EcoMIII	1638	1638	100	1	1638	32940	31303	0	3247
S34EC	114	268099	M.EcoMIII	1638	16	100	1336	1351	236029	236044	1.7	32.2
S34EC	114	268099	M.EcoMIII	1638	15	100	992	1006	28227	28213	6.7	30.2
S34EC	114	268099	M.EcoMIII	1638	15	100	739	753	102728	102714	6.7	30.2
S34EC	114	268099	M.EcoMIII	1638	15	100	1509	1523	203219	203233	6.7	30.2
S34EC	110	41929	M.EcoMIV	765	572	94.41	1	572	18877	18306	0	880
S34EC	91	335969	M.EcoMV	1050	18	100	976	993	153866	153849	0.07	36.2
S34EC	91	335969	M.EcoMV	1050	16	100	781	796	220809	220824	1.1	32.2
S34EC	20	47980	M.EcoNwEVDcm	1419	1419	98.94	1	1419	2240	822	0	2694
S34EC	31	91666	M.EcoMVI	891	891	100	1	891	11917	11027	0	1725
S34EC	38	171415	M.EcoNwEDam	837	837	100	1	837	39459	38623	0	1659
S34EC	91	335969	M.EcoMVII	3606	25	96	596	620	145751	145727	0.004	42.1
S34EC	91	335969	M.EcoMVII	3606	16	100	201	216	152566	152551	3.8	32.2
S34EC	51	23729	M.EcoMVIII	684	684	100	1	684	19829	20512	0	1356
S34EC	9	63422	Eco29kI	645	16	100	238	253	17462	17447	0.66	32.2
S34EC	114	268099	EcoDEC4CORF2749P	1041	16	100	643	658	222854	222869	1.1	32.2
S34EC	7	216858	Eco248534P	1053	16	100	941	956	31138	31153	1.1	32.2
S34EC	7	216858	Eco248534P	1053	15	100	66	80	27248	27262	4.3	30.2
S34EC	7	216858	Eco248534P	1053	15	100	972	986	156995	156981	4.3	30.2
S34EC	70	109274	EcoAPECORF2077P	1590	18	100	608	625	58203	58186	0.11	36.2
S34EC	70	109274	EcoAPECORF2077P	1590	17	100	1034	1050	64710	64694	0.42	34.2
S34EC	70	109274	EcoAPECORF2077P	1590	15	100	1543	1557	78604	78590	6.5	30.2
S34EC	70	109274	EcoAPECORF2077P	1590	15	100	668	682	84587	84573	6.5	30.2
S34EC	268	87094	EcoDEC13EORF3046P	1191	16	100	920	935	83160	83145	1.2	32.2
S34EC	51	23729	Eco7A8ORF29P	684	606	98.51	79	684	19907	20512	0	1130
S34EC	19	65162	EcoDEC2CORF2043P	2019	21	95.24	1522	1542	30536	30556	0.53	34.2

S34EC	268	87094	Eco1886ORF14455P	1053	16	100	883	898	39835	39850	1.1	32.2
S34EC	182	243688	Eco1886ORF14565P	900	15	100	831	845	50238	50252	3.7	30.2
S34EC	51	23729	EcoR7ACORFAP	684	684	97.37	1	684	19829	20512	0	1213
S34EC	51	23729	EcoR100ORF1P	648	606	98.51	43	648	19907	20512	0	1130
S34EC	51	23729	Eco605ORFMP	684	684	93.86	1	684	19829	20512	0	1023
S34EC	91	335969	Eco84137ORF201P	1635	18	100	43	60	127378	127361	0.11	36.2
S34EC	91	335969	Eco84137ORF201P	1635	16	100	1212	1227	126091	126106	1.7	32.2
S34EC	91	335969	Eco84137ORF201P	1635	15	100	1080	1094	29457	29471	6.7	30.2
S34EC	182	243688	Eco1520ORF67P	1563	18	100	1501	1518	12837	12820	0.1	36.2
S34EC	182	243688	Eco1520ORF67P	1563	15	100	1239	1253	45395	45409	6.4	30.2
S34EC	6	107222	Eco15ORF4165P	1197	1197	100	1	1197	6118	7314	0	2278
S128EC	59	205509	M1.EcoMI	1623	16	100	777	792	63726	63741	1.6	32.2
S128EC	59	205509	M1.EcoMI	1623	15	100	1603	1617	17455	17469	6.4	30.2
S128EC	57	67591	M1.EcoMI	1191	17	100	807	823	12645	12629	0.3	34.2
S128EC	59	205509	M.EcoMII	1620	30	93.33	501	530	155953	155982	4e-04	44.1
S128EC	59	205509	M.EcoMII	1620	27	92.59	1022	1048	156492	156518	0.026	38.2
S128EC	59	205509	M.EcoMII	1620	47	85.11	1450	1496	156869	156915	0.026	38.2
S128EC	59	205509	M.EcoMII	1620	15	100	1016	1030	75687	75701	6.4	30.2
S128EC	59	205509	M.EcoMII	1620	15	100	1224	1238	156688	156702	6.4	30.2
S128EC	59	205509	M.EcoMII	1620	15	100	74	88	170176	170190	6.4	30.2
S128EC	56	113591	M.EcoMIII	1638	1638	100	1	1638	40656	42293	0	3247
S128EC	56	113591	M.EcoMIII	1638	15	100	992	1006	45369	45383	6.5	30.2
S128EC	117	183457	M.EcoMIV	765	17	100	332	348	113014	113030	0.19	34.2
S128EC	117	183457	M.EcoMIV	765	15	100	630	644	56426	56412	3	30.2
S128EC	117	183457	M.EcoMIV	765	15	100	345	359	123949	123935	3	30.2
S128EC	141	83117	M.EcoMV	1050	515	83.11	86	600	20982	20468	8e-91	331
S128EC	141	83117	M.EcoMV	1050	104	80.77	817	920	20251	20148	2e-05	48.1
S128EC	97	38974	M.EcoNwEVDcm	1419	1419	100	1	1419	30199	28781	0	2813
S128EC	3	91638	M.EcoMVI	891	891	99.89	1	891	79791	80681	0	1717
S128EC	45	115266	M.EcoNwEDam	837	837	100	1	837	39463	38627	0	1659
S128EC	4	92121	M.EcoMVII	3606	25	96	596	620	40925	40901	0.004	42.1
S128EC	4	92121	M.EcoMVII	3606	16	100	201	216	47740	47725	3.6	32.2
S128EC	1	21878	M.EcoMVIII	684	684	97.66	1	684	19569	20252	0	1229
S128EC	203	215278	Eco29kI	645	16	100	238	253	198705	198690	0.64	32.2
S128EC	85	7987	EcoDEC4CORF2749P	1041	16	100	643	658	5875	5860	1	32.2
S128EC	34	87528	Eco248534P	1053	16	100	941	956	52020	52035	1.1	32.2

S128EC	34	87528	Eco248534P	1053	15	100	66	80	48130	48144	4.2	30.2
S128EC	117	183457	EcoAPECORF2077P	1590	18	100	1494	1511	2476	2459	0.1	36.2
S128EC	117	183457	EcoAPECORF2077P	1590	16	100	827	842	88430	88415	1.6	32.2
S128EC	117	183457	EcoAPECORF2077P	1590	15	100	414	428	146519	146533	6.3	30.2
S128EC	148	752	EcoDEC13EORF3046P	1191	16	100	920	935	800	815	1.2	32.2
S128EC	1	21878	Eco7A8ORF29P	684	621	98.71	64	684	19632	20252	0	1168
S128EC	117	183457	EcoDEC2CORF2043P	2019	17	100	1640	1656	177387	177371	0.51	34.2
S128EC	117	183457	EcoDEC2CORF2043P	2019	15	100	651	665	137409	137395	8	30.2
S128EC	141	83117	Eco1886ORF14455P	1053	1053	99.15	1	1053	21067	20015	0	1974
S128EC	141	83117	Eco1886ORF14455P	1053	19	94.74	24	42	21022	21040	4.2	30.2
S128EC	195	81815	Eco1886ORF14565P	900	15	100	273	287	22782	22796	3.5	30.2
S128EC	195	81815	Eco1886ORF14565P	900	15	100	289	303	41968	41954	3.5	30.2
S128EC	1	21878	EcoR7ACORFAP	684	684	99.71	1	684	19569	20252	0	1340
S128EC	1	21878	EcoR100ORF1P	648	621	98.71	28	648	19632	20252	0	1168
S128EC	1	21878	Eco605ORFMP	684	684	94.74	1	684	19569	20252	0	1070
S128EC	99	23710	Eco84137ORF201P	1635	22	95.45	558	579	16673	16652	0.11	36.2
S128EC	55	43157	Eco1520ORF67P	1563	18	100	1532	1549	39828	39845	0.1	36.2
S128EC	9	85565	Eco15ORF4165P	1197	17	100	486	502	34629	34645	0.3	34.2
S79EC	75	6886	M1.EcoMI	1623	22	95.45	362	383	1356	1335	0.11	36.2
S79EC	221	14372	M1.EcoMI	1191	17	100	336	352	12456	12472	0.32	34.2
S79EC	25	196750	M.EcoMII	1620	30	93.33	501	530	78245	78274	4e-04	44.1
S79EC	25	196750	M.EcoMII	1620	27	92.59	1022	1048	78784	78810	0.028	38.2
S79EC	25	196750	M.EcoMII	1620	47	85.11	1450	1496	79161	79207	0.028	38.2
S79EC	25	196750	M.EcoMII	1620	15	100	1224	1238	78980	78994	6.7	30.2
S79EC	25	196750	M.EcoMII	1620	15	100	74	88	92468	92482	6.7	30.2
S79EC	18	103049	M.EcoMIII	1638	1638	100	1	1638	72877	71240	0	3247
S79EC	18	103049	M.EcoMIII	1638	15	100	992	1006	68164	68150	6.8	30.2
S79EC	30	399446	M.EcoMIV	765	17	100	332	348	67361	67345	0.2	34.2
S79EC	30	399446	M.EcoMIV	765	15	100	345	359	56426	56440	3.1	30.2
S79EC	30	399446	M.EcoMIV	765	15	100	630	644	123949	123963	3.1	30.2
S79EC	79	85015	M.EcoMV	1050	515	83.11	86	600	63614	64128	9e-91	331
S79EC	79	85015	M.EcoMV	1050	104	80.77	817	920	64345	64448	2e-05	48.1
S79EC	90	38974	M.EcoNwEVDcm	1419	1419	100	1	1419	30199	28781	0	2813
S79EC	20	91674	M.EcoMVI	891	891	100	1	891	11922	11032	0	1725
S79EC	24	54310	M.EcoNwEDam	837	837	100	1	837	14926	15762	0	1659
S79EC	43	244197	M.EcoMVII	3606	25	96	596	620	235565	235589	0.004	42.1

S79EC	43	244197	M.EcoMVII	3606	16	100	201	216	228861	228876	3.8	32.2
S79EC	19	7263	M.EcoMVIII	684	684	92.84	1	684	3937	3254	0	967
S79EC	74	97395	Eco29kI	645	16	100	375	390	11466	11481	0.67	32.2
S79EC	74	97395	Eco29kI	645	15	100	494	508	87659	87645	2.6	30.2
S79EC	43	244197	EcoDEC4CORF2749P	1041	16	100	654	669	194270	194255	1.1	32.2
S79EC	2	282902	Eco248534P	1053	16	100	941	956	237967	237952	1.1	32.2
S79EC	2	282902	Eco248534P	1053	15	100	972	986	112134	112148	4.4	30.2
S79EC	2	282902	Eco248534P	1053	15	100	66	80	241857	241843	4.4	30.2
S79EC	168	81331	EcoAPECORF2077P	1590	18	100	608	625	51110	51127	0.11	36.2
S79EC	168	81331	EcoAPECORF2077P	1590	17	100	1034	1050	44603	44619	0.42	34.2
S79EC	168	81331	EcoAPECORF2077P	1590	15	100	668	682	24762	24776	6.6	30.2
S79EC	168	81331	EcoAPECORF2077P	1590	15	100	1543	1557	30745	30759	6.6	30.2
S79EC	231	756	EcoDEC13EORF3046P	1191	16	100	920	935	31	16	1.2	32.2
S79EC	19	7263	Eco7A8ORF29P	684	621	91.79	64	684	3874	3254	0	827
S79EC	32	65698	EcoDEC2CORF2043P	2019	17	100	153	169	58803	58787	0.54	34.2
S79EC	79	85015	Eco1886ORF14455P	1053	1053	99.15	1	1053	63529	64581	0	1974
S79EC	78	38621	Eco1886ORF14565P	900	16	100	186	201	23858	23843	0.94	32.2
S79EC	19	7263	EcoR7ACORFAP	684	684	91.67	1	684	3937	3254	0	904
S79EC	19	7263	EcoR100ORF1P	648	621	91.79	28	648	3874	3254	0	827
S79EC	19	7263	Eco605ORFMP	684	684	91.67	1	684	3937	3254	0	904
S79EC	43	244197	Eco84137ORF201P	1635	18	100	43	60	9742	9759	0.11	36.2
S79EC	43	244197	Eco84137ORF201P	1635	16	100	1212	1227	11029	11014	1.7	32.2
S79EC	59	141955	Eco1520ORF67P	1563	18	100	1501	1518	7344	7327	0.11	36.2
S79EC	59	141955	Eco1520ORF67P	1563	15	100	1239	1253	39902	39916	6.5	30.2
S79EC	51	19196	Eco15ORF4165P	1197	16	100	1102	1117	15831	15816	1.3	32.2
S104EC	6	105528	M1.EcoMI	1623	17	100	1595	1611	75141	75125	0.42	34.2
S104EC	172	310077	M1.EcoMI	1191	15	100	608	622	125537	125523	4.8	30.2
S104EC	172	310077	M1.EcoMI	1191	19	94.74	949	967	261060	261078	4.8	30.2
S104EC	3	157542	M.EcoMII	1620	30	93.33	501	530	10131	10102	4e-04	44.1
S104EC	3	157542	M.EcoMII	1620	47	85.11	1450	1496	9215	9169	0.027	38.2
S104EC	3	157542	M.EcoMII	1620	27	92.59	1022	1048	9592	9566	0.027	38.2
S104EC	3	157542	M.EcoMII	1620	15	100	1224	1238	9396	9382	6.5	30.2
S104EC	3	157542	M.EcoMII	1620	15	100	1016	1030	90397	90383	6.5	30.2
S104EC	172	310077	M.EcoMIII	1638	1638	100	1	1638	237142	238779	0	3247
S104EC	172	310077	M.EcoMIII	1638	16	100	1336	1351	34085	34070	1.7	32.2
S104EC	172	310077	M.EcoMIII	1638	15	100	1509	1523	66920	66906	6.6	30.2

S104EC	172	310077	M.EcoMIII	1638	15	100	739	753	167354	167368	6.6	30.2
S104EC	172	310077	M.EcoMIII	1638	15	100	992	1006	241855	241869	6.6	30.2
S104EC	6	105528	M.EcoMIV	765	765	98.04	1	765	81667	80903	0	1398
S104EC	106	138447	M.EcoMV	1050	22	95.45	690	711	78480	78459	0.068	36.2
S104EC	106	138447	M.EcoMV	1050	15	100	685	699	47911	47897	4.2	30.2
S104EC	61	39008	M.EcoNwEVDcm	1419	1419	100	1	1419	30234	28816	0	2813
S104EC	26	91669	M.EcoMVI	891	891	100	1	891	79822	80712	0	1725
S104EC	7	171391	M.EcoNwEDam	837	837	100	1	837	39459	38623	0	1659
S104EC	10	439682	M.EcoMVII	3606	25	96	596	620	337966	337990	0.004	42.1
S104EC	10	439682	M.EcoMVII	3606	16	100	201	216	331262	331277	3.7	32.2
S104EC	24	86713	M.EcoMVIII	684	684	89.91	1	684	24918	25601	0	809
S104EC	52	97407	Eco29kI	645	16	100	375	390	11478	11493	0.65	32.2
S104EC	52	97407	Eco29kI	645	15	100	494	508	87671	87657	2.6	30.2
S104EC	172	310077	EcoDEC4CORF2749P	1041	16	100	643	658	47260	47245	1.1	32.2
S104EC	24	86713	Eco248534P	1053	16	100	262	277	5807	5822	1.1	32.2
S104EC	72	100364	EcoAPECORF2077P	1590	18	100	608	625	58213	58196	0.1	36.2
S104EC	72	100364	EcoAPECORF2077P	1590	17	100	1034	1050	64720	64704	0.41	34.2
S104EC	72	100364	EcoAPECORF2077P	1590	15	100	1543	1557	78572	78558	6.4	30.2
S104EC	72	100364	EcoAPECORF2077P	1590	15	100	668	682	84555	84541	6.4	30.2
S104EC	102	74555	EcoDEC13EORF3046P	1191	16	100	920	935	74586	74571	1.2	32.2
S104EC	24	86713	Eco7A8ORF29P	684	621	88.89	64	684	24981	25601	0	684
S104EC	10	439682	EcoDEC2CORF2043P	2019	21	95.24	1522	1542	117446	117426	0.52	34.2
S104EC	10	439682	EcoDEC2CORF2043P	2019	15	100	1943	1957	35125	35139	8.2	30.2
S104EC	10	439682	EcoDEC2CORF2043P	2019	19	94.74	1813	1831	421147	421165	8.2	30.2
S104EC	106	138447	Eco1886ORF14455P	1053	16	100	284	299	82904	82889	1.1	32.2
S104EC	102	74555	Eco1886ORF14565P	900	15	100	50	64	13015	13029	3.6	30.2
S104EC	24	86713	EcoR7ACORFAP	684	684	89.47	1	684	24918	25601	0	785
S104EC	24	86713	EcoR7ACORFAP	684	15	100	182	196	60886	60900	2.7	30.2
S104EC	24	86713	EcoR100ORF1P	648	621	88.89	28	648	24981	25601	0	684
S104EC	24	86713	Eco605ORFMP	684	684	89.62	1	684	24918	25601	0	793
S104EC	102	74555	Eco84137ORF201P	1635	22	95.45	558	579	57961	57982	0.11	36.2
S104EC	100	180794	Eco1520ORF67P	1563	18	100	1532	1549	141044	141027	0.1	36.2
S104EC	106	138447	Eco15ORF4165P	1197	16	100	280	295	78830	78845	1.2	32.2
S105EC	22	55343	M1.EcoMI	1623	17	100	1595	1611	40739	40723	0.42	34.2
S105EC	144	151332	M1.EcoMI	1191	15	100	1112	1126	41377	41363	4.8	30.2
S105EC	144	151332	M1.EcoMI	1191	15	100	1116	1130	66557	66571	4.8	30.2

S105EC	1	511704	M.EcoMII	1620	30	93.33	501	530	131846	131817	4e-04	44.1
S105EC	1	511704	M.EcoMII	1620	47	85.11	1450	1496	130930	130884	0.027	38.2
S105EC	1	511704	M.EcoMII	1620	27	92.59	1022	1048	131307	131281	0.027	38.2
S105EC	1	511704	M.EcoMII	1620	16	100	748	763	352128	352113	1.6	32.2
S105EC	1	511704	M.EcoMII	1620	15	100	74	88	117623	117609	6.5	30.2
S105EC	1	511704	M.EcoMII	1620	15	100	1224	1238	131111	131097	6.5	30.2
S105EC	1	511704	M.EcoMII	1620	15	100	1016	1030	212112	212098	6.5	30.2
S105EC	1	511704	M.EcoMII	1620	15	100	1592	1606	363685	363671	6.5	30.2
S105EC	6	79673	M.EcoMIII	1638	1638	100	1	1638	42341	43978	0	3247
S105EC	6	79673	M.EcoMIII	1638	15	100	992	1006	47054	47068	6.6	30.2
S105EC	22	55343	M.EcoMIV	765	765	98.04	1	765	47265	46501	0	1398
S105EC	78	109425	M.EcoMV	1050	22	95.45	690	711	78490	78469	0.068	36.2
S105EC	78	109425	M.EcoMV	1050	15	100	685	699	47921	47907	4.2	30.2
S105EC	49	39001	M.EcoNwEVDcm	1419	1419	100	1	1419	30227	28809	0	2813
S105EC	151	91752	M.EcoMVI	891	891	100	1	891	79790	80680	0	1725
S105EC	24	191365	M.EcoNwEDam	837	837	100	1	837	151981	152817	0	1659
S105EC	41	263784	M.EcoMVII	3606	25	96	596	620	140048	140072	0.004	42.1
S105EC	41	263784	M.EcoMVII	3606	16	100	201	216	133232	133247	3.7	32.2
S105EC	10	62406	M.EcoMVIII	684	684	89.91	1	684	599	1282	0	809
S105EC	132	203080	Eco29kI	645	16	100	238	253	156980	156965	0.64	32.2
S105EC	41	263784	EcoDEC4CORF2749P	1041	16	100	654	669	98641	98626	1	32.2
S105EC	33	19092	Eco248534P	1053	16	100	262	277	16525	16510	1.1	32.2
S105EC	128	180365	EcoAPECORF2077P	1590	18	100	1494	1511	34849	34832	0.1	36.2
S105EC	128	180365	EcoAPECORF2077P	1590	16	100	827	842	120802	120787	1.6	32.2
S105EC	128	180365	EcoAPECORF2077P	1590	15	100	414	428	175107	175121	6.4	30.2
S105EC	121	80653	EcoDEC13EORF3046P	1191	16	100	920	935	43	58	1.2	32.2
S105EC	10	62406	Eco7A8ORF29P	684	621	88.89	64	684	662	1282	0	684
S105EC	38	30199	EcoDEC2CORF2043P	2019	21	95.24	1522	1542	12691	12671	0.52	34.2
S105EC	132	203080	Eco1886ORF14455P	1053	16	100	803	818	7718	7733	1.1	32.2
S105EC	121	80653	Eco1886ORF14565P	900	15	100	884	898	17144	17130	3.6	30.2
S105EC	10	62406	EcoR7ACORFAP	684	684	89.47	1	684	599	1282	0	785
S105EC	10	62406	EcoR7ACORFAP	684	15	100	182	196	36579	36593	2.7	30.2
S105EC	10	62406	EcoR100ORF1P	648	621	88.89	28	648	662	1282	0	684
S105EC	10	62406	Eco605ORFMP	684	684	89.62	1	684	599	1282	0	793
S105EC	41	263784	Eco84137ORF201P	1635	18	100	43	60	158421	158438	0.11	36.2
S105EC	41	263784	Eco84137ORF201P	1635	16	100	1212	1227	159708	159693	1.7	32.2

S105EC	41	263784	Eco84137ORF201P	1635	15	100	1080	1094	256364	256350	6.5	30.2
S105EC	144	151332	Eco1520ORF67P	1563	18	100	1532	1549	10363	10380	0.1	36.2
S105EC	78	109425	Eco15ORF4165P	1197	16	100	280	295	78840	78855	1.2	32.2
S22EC	82	20716	M1.EcoMI	1623	17	100	1595	1611	16159	16143	0.42	34.2
S22EC	272	4250	M1.EcoMI	1191	15	100	1112	1126	1202	1188	4.8	30.2
S22EC	40	55982	M.EcoMII	1620	30	93.33	501	530	33716	33687	4e-04	44.1
S22EC	40	55982	M.EcoMII	1620	47	85.11	1450	1496	32800	32754	0.027	38.2
S22EC	40	55982	M.EcoMII	1620	27	92.59	1022	1048	33177	33151	0.027	38.2
S22EC	40	55982	M.EcoMII	1620	15	100	74	88	19493	19479	6.6	30.2
S22EC	40	55982	M.EcoMII	1620	15	100	1224	1238	32981	32967	6.6	30.2
S22EC	137	43498	M.EcoMIII	1638	1638	100	1	1638	1669	32	0	3247
S22EC	67	16662	M.EcoMIV	765	765	98.04	1	765	15583	16347	0	1398
S22EC	188	29928	M.EcoMV	1050	22	95.45	690	711	13115	13136	0.069	36.2
S22EC	139	12393	M.EcoNwEVDcm	1419	1419	100	1	1419	8857	10275	0	2813
S22EC	83	39538	M.EcoMVI	891	891	100	1	891	9720	8830	0	1725
S22EC	65	41438	M.EcoNwEDam	837	837	100	1	837	11675	10839	0	1659
S22EC	57	42836	M.EcoMVII	3606	25	96	596	620	36966	36990	0.004	42.1
S22EC	57	42836	M.EcoMVII	3606	16	100	201	216	30150	30165	3.7	32.2
S22EC	154	37629	M.EcoMVIII	684	684	89.91	1	684	24926	25609	0	809
S22EC	107	17804	Eco29kI	645	16	100	375	390	6374	6389	0.65	32.2
S22EC	327	4672	EcoDEC4CORF2749P	1041	16	100	654	669	302	287	1.1	32.2
S22EC	154	37629	Eco248534P	1053	16	100	262	277	5815	5830	1.1	32.2
S22EC	215	37189	EcoAPECORF2077P	1590	18	100	1494	1511	2028	2045	0.1	36.2
S22EC	12	42147	EcoDEC13EORF3046P	1191	1191	100	1	1191	19113	17923	0	2278
S22EC	12	42147	EcoDEC13EORF3046P	1191	15	100	148	162	1178	1192	4.8	30.2
S22EC	154	37629	Eco7A8ORF29P	684	621	88.89	64	684	24989	25609	0	684
S22EC	148	17584	EcoDEC2CORF2043P	2019	21	95.24	1522	1542	115	95	0.53	34.2
S22EC	278	8166	Eco1886ORF14455P	1053	16	100	958	973	5621	5606	1.1	32.2
S22EC	344	14545	Eco1886ORF14565P	900	15	100	50	64	1605	1591	3.6	30.2
S22EC	154	37629	EcoR7ACORFAP	684	684	89.47	1	684	24926	25609	0	785
S22EC	154	37629	EcoR100ORF1P	648	621	88.89	28	648	24989	25609	0	684
S22EC	154	37629	Eco605ORFMP	684	684	89.62	1	684	24926	25609	0	793
S22EC	212	5923	Eco84137ORF201P	1635	22	95.45	558	579	2099	2120	0.11	36.2
S22EC	171	21317	Eco1520ORF67P	1563	18	100	1532	1549	10364	10347	0.1	36.2
S22EC	188	29928	Eco15ORF4165P	1197	16	100	280	295	12765	12750	1.2	32.2
S24EC	36	151303	M1.EcoMI	1623	17	100	1595	1611	120916	120900	0.42	34.2

S24EC	110	112295	M1.EcoMI	1191	15	100	807	821	55887	55873	4.8	30.2
S24EC	18	502489	M.EcoMII	1620	30	93.33	501	530	330769	330798	4e-04	44.1
S24EC	18	502489	M.EcoMII	1620	27	92.59	1022	1048	331308	331334	0.027	38.2
S24EC	18	502489	M.EcoMII	1620	47	85.11	1450	1496	331685	331731	0.027	38.2
S24EC	18	502489	M.EcoMII	1620	16	100	748	763	110487	110502	1.7	32.2
S24EC	18	502489	M.EcoMII	1620	15	100	1592	1606	98930	98944	6.5	30.2
S24EC	18	502489	M.EcoMII	1620	15	100	1016	1030	250503	250517	6.5	30.2
S24EC	18	502489	M.EcoMII	1620	15	100	1224	1238	331504	331518	6.5	30.2
S24EC	18	502489	M.EcoMII	1620	15	100	74	88	344992	345006	6.5	30.2
S24EC	90	339419	M.EcoMIII	1638	1638	100	1	1638	73010	71373	0	3247
S24EC	90	339419	M.EcoMIII	1638	16	100	1336	1351	276060	276075	1.7	32.2
S24EC	90	339419	M.EcoMIII	1638	15	100	992	1006	68297	68283	6.6	30.2
S24EC	90	339419	M.EcoMIII	1638	15	100	739	753	142803	142789	6.6	30.2
S24EC	90	339419	M.EcoMIII	1638	15	100	1509	1523	243237	243251	6.6	30.2
S24EC	36	151303	M.EcoMIV	765	765	98.04	1	765	127442	126678	0	1398
S24EC	137	291361	M.EcoMV	1050	18	100	976	993	242517	242534	0.068	36.2
S24EC	137	291361	M.EcoMV	1050	16	100	781	796	175574	175559	1.1	32.2
S24EC	70	11053	M.EcoNwEVDcm	1419	1419	100	1	1419	8849	10267	0	2813
S24EC	13	91639	M.EcoMVI	891	891	100	1	891	11922	11032	0	1725
S24EC	31	166677	M.EcoNwEDam	837	837	100	1	837	39365	38529	0	1659
S24EC	137	291361	M.EcoMVII	3606	25	96	596	620	250633	250657	0.004	42.1
S24EC	137	291361	M.EcoMVII	3606	16	100	405	420	15751	15736	3.7	32.2
S24EC	137	291361	M.EcoMVII	3606	16	100	201	216	243817	243832	3.7	32.2
S24EC	4	32556	M.EcoMVIII	684	684	89.91	1	684	24918	25601	0	809
S24EC	58	97411	Eco29kI	645	16	100	375	390	11482	11497	0.65	32.2
S24EC	58	97411	Eco29kI	645	15	100	494	508	87675	87661	2.6	30.2
S24EC	137	291361	EcoDEC4CORF2749P	1041	16	100	654	669	209226	209211	1.1	32.2
S24EC	4	32556	Eco248534P	1053	16	100	262	277	5807	5822	1.1	32.2
S24EC	133	109362	EcoAPECORF2077P	1590	18	100	608	625	51224	51241	0.1	36.2
S24EC	133	109362	EcoAPECORF2077P	1590	17	100	1034	1050	44717	44733	0.41	34.2
S24EC	133	109362	EcoAPECORF2077P	1590	15	100	668	682	24882	24896	6.4	30.2
S24EC	133	109362	EcoAPECORF2077P	1590	15	100	1543	1557	30865	30879	6.4	30.2
S24EC	10	218440	EcoDEC13EORF3046P	1191	1191	100	1	1191	191876	190686	0	2278
S24EC	10	218440	EcoDEC13EORF3046P	1191	15	100	148	162	173941	173955	4.8	30.2
S24EC	4	32556	Eco7A8ORF29P	684	621	88.89	64	684	24981	25601	0	684
S24EC	137	291361	EcoDEC2CORF2043P	2019	21	95.24	1522	1542	29881	29861	0.52	34.2

S24EC	64	19472	Eco1886ORF14455P	1053	16	100	579	594	6813	6828	1.1	32.2
S24EC	137	291361	Eco1886ORF14565P	900	15	100	565	579	170681	170667	3.6	30.2
S24EC	4	32556	EcoR7ACORFAP	684	684	89.47	1	684	24918	25601	0	785
S24EC	4	32556	EcoR100ORF1P	648	621	88.89	28	648	24981	25601	0	684
S24EC	4	32556	Eco605ORFMP	684	684	89.62	1	684	24918	25601	0	793
S24EC	137	291361	Eco84137ORF201P	1635	18	100	43	60	269006	269023	0.11	36.2
S24EC	137	291361	Eco84137ORF201P	1635	16	100	1212	1227	270293	270278	1.7	32.2
S24EC	137	291361	Eco84137ORF201P	1635	15	100	1218	1232	21145	21159	6.6	30.2
S24EC	63	165828	Eco1520ORF67P	1563	18	100	1501	1518	118527	118544	0.1	36.2
S24EC	63	165828	Eco1520ORF67P	1563	15	100	1239	1253	85969	85955	6.3	30.2
S24EC	21	95664	Eco15ORF4165P	1197	16	100	280	295	36047	36062	1.2	32.2
HVM1147	1	580395	M1.EcoMI	1623	17	100	1595	1611	216582	216566	0.42	34.2
HVM1147	1	580395	M1.EcoMI	1623	15	100	386	400	43450	43464	6.5	30.2
HVM1147	1	580395	M1.EcoMI	1623	15	100	117	131	282799	282785	6.5	30.2
HVM1147	1	580395	M1.EcoMI	1623	15	100	61	75	295538	295552	6.5	30.2
HVM1147	186	218607	M1.EcoMI	1191	19	94.74	1150	1168	30531	30549	4.8	30.2
HVM1147	186	218607	M1.EcoMI	1191	15	100	1112	1126	98512	98498	4.8	30.2
HVM1147	186	218607	M1.EcoMI	1191	15	100	1116	1130	123692	123706	4.8	30.2
HVM1147	22	363997	M.EcoMII	1620	30	93.33	501	530	27216	27245	4e-04	44.1
HVM1147	22	363997	M.EcoMII	1620	27	92.59	1022	1048	27755	27781	0.027	38.2
HVM1147	22	363997	M.EcoMII	1620	47	85.11	1450	1496	28132	28178	0.027	38.2
HVM1147	22	363997	M.EcoMII	1620	15	100	1224	1238	27951	27965	6.5	30.2
HVM1147	22	363997	M.EcoMII	1620	15	100	74	88	41439	41453	6.5	30.2
HVM1147	22	363997	M.EcoMII	1620	15	100	746	760	237254	237240	6.5	30.2
HVM1147	15	339395	M.EcoMIII	1638	1638	100	1	1638	72874	71237	0	3247
HVM1147	15	339395	M.EcoMIII	1638	16	100	1336	1351	275936	275951	1.7	32.2
HVM1147	15	339395	M.EcoMIII	1638	15	100	992	1006	68161	68147	6.6	30.2
HVM1147	15	339395	M.EcoMIII	1638	15	100	739	753	142667	142653	6.6	30.2
HVM1147	15	339395	M.EcoMIII	1638	15	100	1509	1523	243101	243115	6.6	30.2
HVM1147	1	580395	M.EcoMIV	765	765	98.04	1	765	223108	222344	0	1398
HVM1147	182	127498	M.EcoMV	1050	18	100	628	645	9086	9069	0.068	36.2
HVM1147	182	127498	M.EcoMV	1050	15	100	576	590	389	375	4.2	30.2
HVM1147	59	38970	M.EcoNwEVDcm	1419	1419	100	1	1419	8847	10265	0	2813
HVM1147	182	127498	M.EcoMVI	891	891	100	1	891	115536	116426	0	1725
HVM1147	9	190786	M.EcoNwEDam	837	837	100	1	837	39363	38527	0	1659
HVM1147	1	580395	M.EcoMVII	3606	25	96	596	620	567373	567397	0.004	42.1

HVM1147	1	580395	M.EcoMVII	3606	16	100	1317	1332	22300	22285	3.7	32.2
HVM1147	1	580395	M.EcoMVII	3606	16	100	405	420	332489	332474	3.7	32.2
HVM1147	1	580395	M.EcoMVII	3606	16	100	201	216	560557	560572	3.7	32.2
HVM1147	7	86723	M.EcoMVIII	684	684	89.91	1	684	61880	61197	0	809
HVM1147	34	97406	Eco29kI	645	16	100	375	390	86002	85987	0.65	32.2
HVM1147	34	97406	Eco29kI	645	15	100	494	508	9809	9823	2.6	30.2
HVM1147	15	339395	EcoDEC4CORF2749P	1041	16	100	643	658	262761	262776	1.1	32.2
HVM1147	7	86723	Eco248534P	1053	16	100	262	277	80991	80976	1.1	32.2
HVM1147	182	127498	EcoAPECORF2077P	1590	18	100	1511	1528	29250	29233	0.1	36.2
HVM1147	182	127498	EcoAPECORF2077P	1590	17	100	1550	1566	81960	81944	0.41	34.2
HVM1147	182	127498	EcoAPECORF2077P	1590	19	94.74	1493	1511	55828	55810	6.4	30.2
HVM1147	182	127498	EcoAPECORF2077P	1590	15	100	50	64	118117	118103	6.4	30.2
HVM1147	186	218607	EcoDEC13EORF3046P	1191	1191	100	1	1191	26637	27827	0	2278
HVM1147	186	218607	EcoDEC13EORF3046P	1191	15	100	148	162	44572	44558	4.8	30.2
HVM1147	7	86723	Eco7A8ORF29P	684	621	88.89	64	684	61817	61197	0	684
HVM1147	3	263196	EcoDEC2CORF2043P	2019	21	95.24	1522	1542	35284	35264	0.52	34.2
HVM1147	3	263196	EcoDEC2CORF2043P	2019	16	100	703	718	144670	144685	2.1	32.2
HVM1147	3	263196	EcoDEC2CORF2043P	2019	15	100	1262	1276	85229	85243	8.2	30.2
HVM1147	158	33071	Eco1886ORF14455P	1053	16	100	124	139	3759	3774	1.1	32.2
HVM1147	38	256542	Eco1886ORF14565P	900	15	100	831	845	195020	195006	3.6	30.2
HVM1147	7	86723	EcoR7ACORFAP	684	684	89.47	1	684	61880	61197	0	785
HVM1147	7	86723	EcoR7ACORFAP	684	15	100	182	196	25900	25886	2.7	30.2
HVM1147	7	86723	EcoR100ORF1P	648	621	88.89	28	648	61817	61197	0	684
HVM1147	7	86723	Eco605ORFMP	684	684	89.62	1	684	61880	61197	0	793
HVM1147	29	19688	Eco84137ORF201P	1635	22	95.45	558	579	16667	16646	0.11	36.2
HVM1147	186	218607	Eco1520ORF67P	1563	18	100	1532	1549	67498	67515	0.1	36.2
HVM1147	1	580395	Eco15ORF4165P	1197	16	100	280	295	59690	59675	1.2	32.2
HVM1147	1	580395	Eco15ORF4165P	1197	15	100	72	86	421943	421957	4.8	30.2
S21EC	84	216724	M1.EcoMI	1623	17	100	1595	1611	216568	216552	0.42	34.2
S21EC	84	216724	M1.EcoMI	1623	15	100	386	400	43436	43450	6.6	30.2
S21EC	348	359763	M1.EcoMI	1191	19	94.74	93	111	128242	128224	4.8	30.2
S21EC	180	298926	M.EcoMII	1620	30	93.33	501	530	196616	196645	4e-04	44.1
S21EC	180	298926	M.EcoMII	1620	27	92.59	1022	1048	197155	197181	0.027	38.2
S21EC	180	298926	M.EcoMII	1620	47	85.11	1450	1496	197532	197578	0.027	38.2
S21EC	180	298926	M.EcoMII	1620	15	100	1016	1030	116350	116364	6.6	30.2
S21EC	180	298926	M.EcoMII	1620	15	100	1224	1238	197351	197365	6.6	30.2

S21EC	180	298926	M.EcoMII	1620	15	100	74	88	210839	210853	6.6	30.2
S21EC	4	309356	M.EcoMIII	1638	1638	100	1	1638	236554	238191	0	3247
S21EC	4	309356	M.EcoMIII	1638	16	100	1336	1351	33491	33476	1.7	32.2
S21EC	4	309356	M.EcoMIII	1638	15	100	1509	1523	66326	66312	6.7	30.2
S21EC	4	309356	M.EcoMIII	1638	15	100	739	753	166760	166774	6.7	30.2
S21EC	4	309356	M.EcoMIII	1638	15	100	992	1006	241267	241281	6.7	30.2
S21EC	405	2699	M.EcoMIV	765	765	98.04	1	765	2535	1771	0	1398
S21EC	212	435574	M.EcoMV	1050	18	100	976	993	99645	99628	0.069	36.2
S21EC	212	435574	M.EcoMV	1050	16	100	781	796	166588	166603	1.1	32.2
S21EC	102	136424	M.EcoNwEVDcm	1419	1419	100	1	1419	8833	10251	0	2813
S21EC	11	91682	M.EcoMVI	891	891	100	1	891	11906	11016	0	1725
S21EC	66	190866	M.EcoNwEDam	837	837	100	1	837	151482	152318	0	1659
S21EC	212	435574	M.EcoMVII	3606	25	96	596	620	91529	91505	0.004	42.1
S21EC	212	435574	M.EcoMVII	3606	16	100	201	216	98345	98330	3.7	32.2
S21EC	212	435574	M.EcoMVII	3606	16	100	405	420	326411	326426	3.7	32.2
S21EC	50	9471	M.EcoMVIII	684	606	98.51	79	684	3882	3277	0	1130
S21EC	102	136424	Eco29kI	645	16	100	375	390	50495	50510	0.65	32.2
S21EC	102	136424	Eco29kI	645	15	100	494	508	126688	126674	2.6	30.2
S21EC	212	435574	EcoDEC4CORF2749P	1041	16	100	654	669	132936	132951	1.1	32.2
S21EC	348	359763	Eco248534P	1053	16	100	941	956	78301	78286	1.1	32.2
S21EC	348	359763	Eco248534P	1053	15	100	66	80	82191	82177	4.3	30.2
S21EC	166	316456	EcoAPECORF2077P	1590	18	100	1494	1511	170940	170923	0.1	36.2
S21EC	166	316456	EcoAPECORF2077P	1590	16	100	1397	1412	4489	4474	1.6	32.2
S21EC	166	316456	EcoAPECORF2077P	1590	16	100	827	842	256893	256878	1.6	32.2
S21EC	166	316456	EcoAPECORF2077P	1590	15	100	55	69	19023	19009	6.5	30.2
S21EC	166	316456	EcoAPECORF2077P	1590	15	100	1473	1487	91423	91409	6.5	30.2
S21EC	166	316456	EcoAPECORF2077P	1590	15	100	514	528	129072	129086	6.5	30.2
S21EC	166	316456	EcoAPECORF2077P	1590	15	100	414	428	311198	311212	6.5	30.2
S21EC	180	298926	EcoDEC13EORF3046P	1191	16	100	1050	1065	223972	223957	1.2	32.2
S21EC	180	298926	EcoDEC13EORF3046P	1191	15	100	156	170	251508	251522	4.8	30.2
S21EC	50	9471	Eco7A8ORF29P	684	684	100	1	684	3960	3277	0	1356
S21EC	212	435574	EcoDEC2CORF2043P	2019	21	95.24	1522	1542	312281	312301	0.53	34.2
S21EC	212	435574	EcoDEC2CORF2043P	2019	19	94.74	1813	1831	8348	8330	8.2	30.2
S21EC	212	435574	EcoDEC2CORF2043P	2019	15	100	1943	1957	402710	402696	8.2	30.2
S21EC	102	136424	Eco1886ORF14455P	1053	16	100	883	898	93130	93145	1.1	32.2
S21EC	348	359763	Eco1886ORF14565P	900	15	100	176	190	121385	121371	3.6	30.2

S21EC	348	359763	Eco1886ORF14565P	900	15	100	289	303	170166	170180	3.6	30.2
S21EC	348	359763	Eco1886ORF14565P	900	15	100	273	287	189162	189148	3.6	30.2
S21EC	50	9471	EcoR7ACORFAP	684	621	98.55	64	684	3897	3277	0	1160
S21EC	50	9471	EcoR100ORF1P	648	648	100	1	648	3924	3277	0	1285
S21EC	50	9471	Eco605ORFMP	684	621	95.17	64	684	3897	3277	0	993
S21EC	212	435574	Eco84137ORF201P	1635	18	100	43	60	73156	73139	0.11	36.2
S21EC	212	435574	Eco84137ORF201P	1635	16	100	1212	1227	71869	71884	1.7	32.2
S21EC	212	435574	Eco84137ORF201P	1635	15	100	1218	1232	321017	321003	6.7	30.2
S21EC	258	56201	Eco1520ORF67P	1563	18	100	1532	1549	16452	16435	0.1	36.2
S21EC	84	216724	Eco15ORF4165P	1197	16	100	280	295	59676	59661	1.2	32.2
S6EC	8	134267	M1.EcoMI	1623	17	100	1595	1611	103880	103864	0.43	34.2
S6EC	227	168437	M1.EcoMI	1191	15	100	1112	1126	70835	70821	4.9	30.2
S6EC	227	168437	M1.EcoMI	1191	15	100	1116	1130	96015	96029	4.9	30.2
S6EC	14	143112	M.EcoMII	1620	30	93.33	501	530	26234	26263	4e-04	44.1
S6EC	14	143112	M.EcoMII	1620	27	92.59	1022	1048	26773	26799	0.027	38.2
S6EC	14	143112	M.EcoMII	1620	47	85.11	1450	1496	27150	27196	0.027	38.2
S6EC	14	143112	M.EcoMII	1620	15	100	1224	1238	26969	26983	6.7	30.2
S6EC	14	143112	M.EcoMII	1620	15	100	74	88	40457	40471	6.7	30.2
S6EC	5	316210	M.EcoMIII	1638	1638	100	1	1638	49789	48152	0	3247
S6EC	5	316210	M.EcoMIII	1638	16	100	1336	1351	252851	252866	1.7	32.2
S6EC	5	316210	M.EcoMIII	1638	15	100	992	1006	45076	45062	6.8	30.2
S6EC	5	316210	M.EcoMIII	1638	15	100	739	753	119582	119568	6.8	30.2
S6EC	5	316210	M.EcoMIII	1638	15	100	1509	1523	220016	220030	6.8	30.2
S6EC	8	134267	M.EcoMIV	765	765	98.04	1	765	110406	109642	0	1398
S6EC	8	134267	M.EcoMIV	765	15	100	258	272	11439	11425	3.1	30.2
S6EC	268	135229	M.EcoMV	1050	22	95.45	690	711	60040	60061	0.07	36.2
S6EC	268	135229	M.EcoMV	1050	15	100	685	699	90609	90623	4.3	30.2
S6EC	17	38973	M.EcoNwEVDcm	1419	1419	99.93	1	1419	30199	28781	0	2805
S6EC	252	91785	M.EcoMVI	891	891	99.89	1	891	12035	11145	0	1717
S6EC	24	171420	M.EcoNwEDam	837	837	100	1	837	39457	38621	0	1659
S6EC	83	22078	M.EcoMVII	3606	3606	99.97	1	3606	12505	16110	0	6980
S6EC	27	16315	M.EcoMVIII	684	684	89.33	1	684	12170	12853	0	777
S6EC	238	115057	Eco29kI	645	16	100	238	253	68957	68942	0.66	32.2
S6EC	234	350559	EcoDEC4CORF2749P	1041	16	100	654	669	312184	312169	1.1	32.2
S6EC	27	16315	Eco248534P	1053	16	100	262	277	2218	2233	1.1	32.2
S6EC	192	109252	EcoAPECORF2077P	1590	18	100	608	625	58211	58194	0.11	36.2

S6EC	192	109252	EcoAPECORF2077P	1590	17	100	1034	1050	64718	64702	0.42	34.2
S6EC	192	109252	EcoAPECORF2077P	1590	15	100	1543	1557	78582	78568	6.6	30.2
S6EC	192	109252	EcoAPECORF2077P	1590	15	100	668	682	84565	84551	6.6	30.2
S6EC	167	2730	EcoDEC13EORF3046P	1191	16	100	920	935	42	57	1.2	32.2
S6EC	27	16315	Eco7A8ORF29P	684	609	88.83	76	684	12245	12853	0	668
S6EC	234	350559	EcoDEC2CORF2043P	2019	21	95.24	1522	1542	132961	132941	0.53	34.2
S6EC	234	350559	EcoDEC2CORF2043P	2019	15	100	1943	1957	36146	36160	8.3	30.2
S6EC	268	135229	Eco1886ORF14455P	1053	16	100	284	299	55616	55631	1.1	32.2
S6EC	234	350559	Eco1886ORF14565P	900	15	100	565	579	273639	273625	3.7	30.2
S6EC	27	16315	EcoR7ACORFAP	684	684	89.33	1	684	12170	12853	0	777
S6EC	27	16315	EcoR100ORF1P	648	609	88.83	40	648	12245	12853	0	668
S6EC	27	16315	Eco605ORFMP	684	684	89.47	1	684	12170	12853	0	785
S6EC	67	117673	Eco84137ORF201P	1635	18	100	43	60	21292	21309	0.11	36.2
S6EC	67	117673	Eco84137ORF201P	1635	16	100	1212	1227	22579	22564	1.7	32.2
S6EC	227	168437	Eco1520ORF67P	1563	18	100	1532	1549	39821	39838	0.1	36.2
S6EC	268	135229	Eco15ORF4165P	1197	16	100	280	295	59690	59675	1.2	32.2
S114EC	70	213053	M1.EcoMI	1623	17	100	1595	1611	62122	62138	0.41	34.2
S114EC	138	57285	M1.EcoMI	1191	15	100	1102	1116	33674	33688	4.7	30.2
S114EC	31	79242	M.EcoMII	1620	30	93.33	501	530	28242	28271	4e-04	44.1
S114EC	31	79242	M.EcoMII	1620	27	92.59	1022	1048	28781	28807	0.026	38.2
S114EC	31	79242	M.EcoMII	1620	47	85.11	1450	1496	29158	29204	0.026	38.2
S114EC	31	79242	M.EcoMII	1620	15	100	1224	1238	28977	28991	6.4	30.2
S114EC	31	79242	M.EcoMII	1620	15	100	74	88	42465	42479	6.4	30.2
S114EC	26	101757	M.EcoMIII	1638	1202	100	1	1202	100623	101824	0	2383
S114EC	26	101757	M.EcoMIII	1638	15	100	739	753	30830	30844	6.5	30.2
S114EC	70	213053	M.EcoMIV	765	765	98.04	1	765	55596	56360	0	1398
S114EC	40	108879	M.EcoMV	1050	18	100	976	993	55987	56004	0.067	36.2
S114EC	79	38972	M.EcoNwEVDcm	1419	1419	100	1	1419	30198	28780	0	2813
S114EC	169	91757	M.EcoMVI	891	891	100	1	891	12047	11157	0	1725
S114EC	20	98772	M.EcoNwEDam	837	837	100	1	837	39461	38625	0	1659
S114EC	40	108879	M.EcoMVII	3606	25	96	596	620	64103	64127	0.004	42.1
S114EC	40	108879	M.EcoMVII	3606	16	100	201	216	57287	57302	3.7	32.2
S114EC	55	25648	M.EcoMVIII	684	684	89.91	1	684	21755	22438	0	809
S114EC	72	93548	Eco29kI	645	16	100	375	390	7619	7634	0.64	32.2
S114EC	72	93548	Eco29kI	645	15	100	494	508	83812	83798	2.5	30.2
S114EC	40	108879	EcoDEC4CORF2749P	1041	16	100	654	669	22696	22681	1	32.2

S114EC	55	25648	Eco248534P	1053	16	100	262	277	2644	2659	1.1	32.2
S114EC	183	109549	EcoAPECORF2077P	1590	18	100	608	625	58215	58198	0.1	36.2
S114EC	183	109549	EcoAPECORF2077P	1590	17	100	1034	1050	64722	64706	0.41	34.2
S114EC	183	109549	EcoAPECORF2077P	1590	15	100	1543	1557	78580	78566	6.3	30.2
S114EC	183	109549	EcoAPECORF2077P	1590	15	100	668	682	84563	84549	6.3	30.2
S114EC	138	57285	EcoDEC13EORF3046P	1191	16	100	920	935	46	61	1.2	32.2
S114EC	55	25648	Eco7A8ORF29P	684	621	88.89	64	684	21818	22438	0	684
S114EC	19	176434	EcoDEC2CORF2043P	2019	21	95.24	1522	1542	106490	106470	0.52	34.2
S114EC	19	176434	EcoDEC2CORF2043P	2019	15	100	1262	1276	156435	156449	8.1	30.2
S114EC	113	167953	Eco1886ORF14455P	1053	16	100	803	818	120630	120615	1.1	32.2
S114EC	82	10078	Eco1886ORF14565P	900	15	100	50	64	5740	5754	3.6	30.2
S114EC	55	25648	EcoR7ACORFAP	684	684	89.47	1	684	21755	22438	0	785
S114EC	55	25648	EcoR100ORF1P	648	621	88.89	28	648	21818	22438	0	684
S114EC	55	25648	Eco605ORFMP	684	684	89.62	1	684	21755	22438	0	793
S114EC	138	57285	Eco84137ORF201P	1635	22	95.45	558	579	16671	16650	0.11	36.2
S114EC	52	162605	Eco1520ORF67P	1563	18	100	1532	1549	151092	151075	0.1	36.2
S114EC	30	146878	Eco15ORF4165P	1197	16	100	280	295	140995	140980	1.2	32.2
S32EC	146	72004	M1.EcoMI	1623	17	100	1595	1611	38263	38279	0.42	34.2
S32EC	200	28704	M1.EcoMI	1191	15	100	1112	1126	20654	20640	4.8	30.2
S32EC	14	233292	M.EcoMII	1620	30	93.33	501	530	140415	140444	4e-04	44.1
S32EC	14	233292	M.EcoMII	1620	27	92.59	1022	1048	140954	140980	0.027	38.2
S32EC	14	233292	M.EcoMII	1620	47	85.11	1450	1496	141331	141377	0.027	38.2
S32EC	14	233292	M.EcoMII	1620	15	100	1016	1030	60149	60163	6.5	30.2
S32EC	14	233292	M.EcoMII	1620	15	100	1224	1238	141150	141164	6.5	30.2
S32EC	14	233292	M.EcoMII	1620	15	100	74	88	154638	154652	6.5	30.2
S32EC	111	16217	M.EcoMIII	1638	1638	100	1	1638	3433	1796	0	3247
S32EC	146	72004	M.EcoMIV	765	765	98.04	1	765	31737	32501	0	1398
S32EC	84	34659	M.EcoMV	1050	18	100	628	645	26635	26652	0.068	36.2
S32EC	34	12360	M.EcoNwEVDcm	1419	1419	100	1	1419	8853	10271	0	2813
S32EC	224	39829	M.EcoMVI	891	891	100	1	891	12041	11151	0	1725
S32EC	82	61224	M.EcoNwEDam	837	837	100	1	837	21840	22676	0	1659
S32EC	26	55824	M.EcoMVII	3606	25	96	596	620	7731	7707	0.004	42.1
S32EC	26	55824	M.EcoMVII	3606	16	100	201	216	14547	14532	3.7	32.2
S32EC	115	13419	M.EcoMVIII	684	684	89.91	1	684	813	1496	0	809
S32EC	214	97893	Eco29kI	645	16	100	238	253	51793	51778	0.65	32.2
S32EC	83	32998	EcoDEC4CORF2749P	1041	16	100	643	658	10851	10866	1.1	32.2

S32EC	95	7477	Eco248534P	1053	16	100	262	277	1745	1730	1.1	32.2
S32EC	84	34659	EcoAPECORF2077P	1590	18	100	1511	1528	6471	6488	0.1	36.2
S32EC	165	1354	EcoDEC13EORF3046P	1191	16	100	920	935	30	15	1.2	32.2
S32EC	115	13419	Eco7A8ORF29P	684	621	88.89	64	684	876	1496	0	684
S32EC	68	57943	EcoDEC2CORF2043P	2019	21	95.24	1522	1542	22295	22315	0.52	34.2
S32EC	79	20753	Eco1886ORF14455P	1053	16	100	958	973	2608	2623	1.1	32.2
S32EC	221	63124	Eco1886ORF14565P	900	15	100	273	287	4162	4176	3.6	30.2
S32EC	221	63124	Eco1886ORF14565P	900	15	100	289	303	23279	23265	3.6	30.2
S32EC	115	13419	EcoR7ACORFAP	684	684	89.47	1	684	813	1496	0	785
S32EC	115	13419	EcoR100ORF1P	648	621	88.89	28	648	876	1496	0	684
S32EC	115	13419	Eco605ORFMP	684	684	89.62	1	684	813	1496	0	793
S32EC	59	22066	Eco84137ORF201P	1635	22	95.45	558	579	5472	5493	0.11	36.2
S32EC	81	21380	Eco1520ORF67P	1563	18	100	1532	1549	11023	11040	0.1	36.2
S32EC	28	54660	Eco15ORF4165P	1197	16	100	280	295	8801	8786	1.2	32.2
S19EC	30	208187	M1.EcoMI	1623	17	100	1595	1611	177800	177784	0.43	34.2
S19EC	30	208187	M1.EcoMI	1623	15	100	386	400	4668	4682	6.8	30.2
S19EC	150	112391	M1.EcoMI	1191	15	100	807	821	55885	55871	5	30.2
S19EC	40	137949	M.EcoMII	1620	30	93.33	501	530	54483	54512	4e-04	44.1
S19EC	40	137949	M.EcoMII	1620	27	92.59	1022	1048	55022	55048	0.028	38.2
S19EC	40	137949	M.EcoMII	1620	47	85.11	1450	1496	55399	55445	0.028	38.2
S19EC	40	137949	M.EcoMII	1620	15	100	1224	1238	55218	55232	6.8	30.2
S19EC	40	137949	M.EcoMII	1620	15	100	74	88	68706	68720	6.8	30.2
S19EC	29	90610	M.EcoMIII	1638	1638	100	1	1638	72875	71238	0	3247
S19EC	29	90610	M.EcoMIII	1638	15	100	992	1006	68162	68148	6.8	30.2
S19EC	30	208187	M.EcoMIV	765	765	98.04	1	765	184326	183562	0	1398
S19EC	66	15903	M.EcoMV	1050	1036	95.85	6	1041	890	1925	0	1687
S19EC	43	13451	M.EcoNwEVDcm	1419	1419	100	1	1419	4677	3259	0	2813
S19EC	18	91643	M.EcoMVI	891	891	100	1	891	79796	80686	0	1725
S19EC	2	191365	M.EcoNwEDam	837	837	100	1	837	151981	152817	0	1659
S19EC	15	117560	M.EcoMVII	3606	25	96	596	620	114826	114802	0.004	42.1
S19EC	68	2686	M.EcoMVIII	684	684	100	1	684	741	58	0	1356
S19EC	65	30854	Eco29kI	645	16	100	375	390	19444	19429	0.67	32.2
S19EC	124	61304	EcoDEC4CORF2749P	1041	16	100	84	99	53005	52990	1.1	32.2
S19EC	246	17800	Eco248534P	1053	16	100	262	277	5805	5820	1.1	32.2
S19EC	96	6275	EcoAPECORF2077P	1590	18	100	1431	1448	4005	3988	0.11	36.2
S19EC	134	77705	EcoDEC13EORF3046P	1191	16	100	920	935	77735	77720	1.3	32.2

S19EC	68	2686	Eco7A8ORF29P	684	606	98.51	79	684	663	58	0	1130
S19EC	28	64164	EcoDEC2CORF2043P	2019	21	95.24	1522	1542	41963	41943	0.54	34.2
S19EC	66	15903	Eco1886ORF14455P	1053	641	81.59	7	647	891	1531	6e-92	335
S19EC	66	15903	Eco1886ORF14455P	1053	173	82.08	805	977	1689	1861	1e-12	71.9
S19EC	134	77705	Eco1886ORF14565P	900	15	100	884	898	60634	60648	3.7	30.2
S19EC	68	2686	EcoR7ACORFAP	684	684	97.37	1	684	741	58	0	1213
S19EC	68	2686	EcoR100ORF1P	648	606	98.51	43	648	663	58	0	1130
S19EC	68	2686	Eco605ORFMP	684	684	93.86	1	684	741	58	0	1023
S19EC	74	27025	Eco84137ORF201P	1635	22	95.45	558	579	10431	10452	0.11	36.2
S19EC	53	181931	Eco1520ORF67P	1563	18	100	1532	1549	171024	171007	0.11	36.2
S19EC	30	208187	Eco15ORF4165P	1197	16	100	280	295	20908	20893	1.3	32.2
HVM277	67	183966	M1.EcoMI	1623	18	100	364	381	131631	131648	0.11	36.2
HVM277	158	14260	M1.EcoMI	1191	16	100	1153	1168	9438	9423	1.2	32.2
HVM277	18	109471	M.EcoMII	1620	19	100	1333	1351	41227	41209	0.027	38.2
HVM277	200	41957	M.EcoMIII	1638	1638	100	1	1638	29045	30682	0	3247
HVM277	42	138729	M.EcoMIV	765	17	100	332	348	60286	60270	0.19	34.2
HVM277	42	138729	M.EcoMIV	765	15	100	345	359	49124	49138	3	30.2
HVM277	42	138729	M.EcoMIV	765	15	100	630	644	116874	116888	3	30.2
HVM277	269	29852	M.EcoMV	1050	515	83.3	86	600	11185	11699	3e-93	339
HVM277	269	29852	M.EcoMV	1050	104	80.77	817	920	11916	12019	2e-05	48.1
HVM277	28	27751	M.EcoNwEVDcm	1419	1419	100	1	1419	8855	10273	0	2813
HVM277	32	79613	M.EcoMVI	891	891	100	1	891	67766	68656	0	1725
HVM277	9	65023	M.EcoNwEDam	837	837	100	1	837	25639	26475	0	1659
HVM277	25	98668	M.EcoMVII	3606	25	96	596	620	4533	4509	0.004	42.1
HVM277	25	98668	M.EcoMVII	3606	16	100	201	216	11349	11334	3.7	32.2
HVM277	125	10536	M.EcoMVIII	684	16	100	410	425	3810	3795	0.68	32.2
HVM277	178	3937	Eco29kI	645	16	100	375	390	3182	3197	0.64	32.2
HVM277	25	98668	EcoDEC4CORF2749P	1041	16	100	654	669	45940	45955	1	32.2
HVM277	64	41687	Eco248534P	1053	16	100	941	956	14016	14001	1.1	32.2
HVM277	64	41687	Eco248534P	1053	15	100	66	80	17906	17892	4.2	30.2
HVM277	356	33553	EcoAPECORF2077P	1590	19	100	312	330	29748	29766	0.026	38.2
HVM277	356	33553	EcoAPECORF2077P	1590	15	100	724	738	25893	25879	6.4	30.2
HVM277	275	749	EcoDEC13EORF3046P	1191	16	100	920	935	800	815	1.2	32.2
HVM277	70	25436	Eco7A8ORF29P	684	20	95	213	232	7604	7623	0.68	32.2
HVM277	139	16032	EcoDEC2CORF2043P	2019	21	95.24	1522	1542	2412	2392	0.52	34.2
HVM277	269	29852	Eco1886ORF14455P	1053	1053	99.24	1	1053	11100	12152	0	1982

HVM277	225	7842	Eco1886ORF14565P	900	15	100	50	64	5851	5865	3.6	30.2
HVM277	1	99771	EcoR7ACORFAP	684	21	95.24	181	201	14533	14553	0.17	34.2
HVM277	70	25436	EcoR100ORF1P	648	20	95	177	196	7604	7623	0.65	32.2
HVM277	63	81152	Eco605ORFMP	684	16	100	265	280	45640	45625	0.68	32.2
HVM277	321	90202	Eco84137ORF201P	1635	18	100	320	337	46825	46842	0.11	36.2
HVM277	174	5673	Eco1520ORF67P	1563	18	100	1532	1549	2326	2343	0.1	36.2
HVM277	159	16814	Eco15ORF4165P	1197	16	100	280	295	10135	10120	1.2	32.2
HVM52	75	129723	M1.EcoMI	1623	18	100	364	381	77411	77428	0.11	36.2
HVM52	269	11441	M1.EcoMI	1191	16	100	1153	1168	10413	10398	1.3	32.2
HVM52	14	80118	M.EcoMII	1620	19	100	1333	1351	73708	73690	0.028	38.2
HVM52	14	80118	M.EcoMII	1620	17	100	273	289	32010	31994	0.43	34.2
HVM52	22	14762	M.EcoMIII	1638	1638	100	1	1638	2058	3695	0	3247
HVM52	88	101224	M.EcoMIV	765	17	100	332	348	31021	31037	0.2	34.2
HVM52	88	101224	M.EcoMIV	765	15	100	345	359	42183	42169	3.2	30.2
HVM52	594	291	M.EcoMV	1050	322	86.34	86	407	324	3	3e-78	289
HVM52	126	9651	M.EcoNwEVDcm	1419	1419	100	1	1419	7404	8822	0	2813
HVM52	13	88066	M.EcoMVI	891	891	100	1	891	76219	77109	0	1725
HVM52	17	161692	M.EcoNwEDam	837	837	100	1	837	132031	132867	0	1659
HVM52	78	11155	M.EcoMVII	3606	25	96	596	620	10687	10711	0.004	42.1
HVM52	78	11155	M.EcoMVII	3606	16	100	201	216	3871	3886	3.8	32.2
HVM52	177	3814	M.EcoMVIII	684	680	94.26	5	684	3896	3217	0	1039
HVM52	235	7134	Eco29kI	645	16	100	375	390	6316	6331	0.67	32.2
HVM52	29	9398	EcoDEC4CORF2749P	1041	16	100	643	658	4796	4811	1.1	32.2
HVM52	143	18352	Eco248534P	1053	16	100	941	956	10896	10881	1.1	32.2
HVM52	143	18352	Eco248534P	1053	15	100	66	80	14786	14772	4.4	30.2
HVM52	540	25330	EcoAPECORF2077P	1590	19	100	312	330	22045	22063	0.027	38.2
HVM52	540	25330	EcoAPECORF2077P	1590	15	100	724	738	18190	18176	6.6	30.2
HVM52	451	747	EcoDEC13EORF3046P	1191	16	100	920	935	30	15	1.3	32.2
HVM52	177	3814	Eco7A8ORF29P	684	621	95.65	64	684	3837	3217	0	1017
HVM52	167	27764	EcoDEC2CORF2043P	2019	21	95.24	1522	1542	17552	17572	0.54	34.2
HVM52	763	15634	Eco1886ORF14455P	1053	726	99.45	328	1053	1	726	0	1366
HVM52	254	6154	Eco1886ORF14565P	900	15	100	831	845	5954	5968	3.7	30.2
HVM52	177	3814	EcoR7ACORFAP	684	680	94.85	5	684	3896	3217	0	1070
HVM52	177	3814	EcoR100ORF1P	648	621	95.65	28	648	3837	3217	0	1017
HVM52	177	3814	Eco605ORFMP	684	680	99.41	5	684	3896	3217	0	1316
HVM52	209	10271	Eco84137ORF201P	1635	27	92.59	811	837	5674	5648	0.028	38.2

HVM52	209	10271	Eco84137ORF201P	1635	20	95	550	569	5914	5895	1.7	32.2
HVM52	209	10271	Eco1520ORF67P	1563	1563	99.94	1	1563	6448	4886	0	3049
HVM52	480	4279	Eco15ORF4165P	1197	16	100	280	295	4227	4242	1.3	32.2
HVM2044	35	10680	M1.EcoMI	1623	18	100	364	381	9781	9764	0.11	36.2
HVM2044	207	35721	M1.EcoMI	1191	16	100	1153	1168	30140	30125	1.2	32.2
HVM2044	47	171268	M.EcoMII	1620	19	100	1333	1351	2761	2743	0.027	38.2
HVM2044	47	171268	M.EcoMII	1620	20	95	1287	1306	85005	84986	1.7	32.2
HVM2044	154	102608	M.EcoMIII	1638	1638	100	1	1638	89696	91333	0	3247
HVM2044	154	102608	M.EcoMIII	1638	15	100	739	753	19903	19917	6.7	30.2
HVM2044	116	190127	M.EcoMIV	765	17	100	332	348	151238	151254	0.2	34.2
HVM2044	116	190127	M.EcoMIV	765	15	100	630	644	94650	94636	3.1	30.2
HVM2044	116	190127	M.EcoMIV	765	15	100	345	359	162400	162386	3.1	30.2
HVM2044	180	299	M.EcoMV	1050	322	86.34	86	407	324	3	3e-78	289
HVM2044	63	38975	M.EcoNwEVDcm	1419	1419	100	1	1419	30201	28783	0	2813
HVM2044	2	91667	M.EcoMVI	891	891	100	1	891	79820	80710	0	1725
HVM2044	19	171397	M.EcoNwEDam	837	837	100	1	837	132013	132849	0	1659
HVM2044	66	267189	M.EcoMVII	3606	25	96	596	620	150444	150468	0.004	42.1
HVM2044	66	267189	M.EcoMVII	3606	16	100	201	216	143628	143643	3.8	32.2
HVM2044	38	14532	M.EcoMVIII	684	684	97.37	1	684	3887	3204	0	1213
HVM2044	76	97410	Eco29kI	645	16	100	375	390	86004	85989	0.66	32.2
HVM2044	76	97410	Eco29kI	645	15	100	494	508	9811	9825	2.6	30.2
HVM2044	66	267189	EcoDEC4CORF2749P	1041	16	100	654	669	109037	109022	1.1	32.2
HVM2044	1	361864	Eco248534P	1053	16	100	941	956	315265	315250	1.1	32.2
HVM2044	1	361864	Eco248534P	1053	15	100	972	986	189432	189446	4.3	30.2
HVM2044	1	361864	Eco248534P	1053	15	100	66	80	319155	319141	4.3	30.2
HVM2044	98	7274	EcoAPECORF2077P	1590	19	100	312	330	3469	3487	0.027	38.2
HVM2044	89	755	EcoDEC13EORF3046P	1191	16	100	920	935	30	15	1.2	32.2
HVM2044	38	14532	Eco7A8ORF29P	684	621	98.55	64	684	3824	3204	0	1160
HVM2044	192	420937	EcoDEC2CORF2043P	2019	21	95.24	1522	1542	70375	70395	0.53	34.2
HVM2044	192	420937	EcoDEC2CORF2043P	2019	16	100	910	925	92504	92519	2.1	32.2
HVM2044	192	420937	EcoDEC2CORF2043P	2019	16	100	1114	1129	384713	384728	2.1	32.2
HVM2044	192	420937	EcoDEC2CORF2043P	2019	16	100	1428	1443	400709	400724	2.1	32.2
HVM2044	219	388	Eco1886ORF14455P	1053	461	99.35	445	905	2	462	0	890
HVM2044	192	420937	Eco1886ORF14565P	900	15	100	831	845	227486	227500	3.7	30.2
HVM2044	38	14532	EcoR7ACORFAP	684	684	100	1	684	3887	3204	0	1356
HVM2044	38	14532	EcoR100ORF1P	648	621	98.55	28	648	3824	3204	0	1160

HVM2044	38	14532	Eco605ORFMP	684	684	94.44	1	684	3887	3204	0	1055
HVM2044	43	14550	Eco84137ORF201P	1635	23	95.65	547	569	8934	8956	0.027	38.2
HVM2044	43	14550	Eco84137ORF201P	1635	27	92.59	811	837	9177	9203	0.027	38.2
HVM2044	43	14550	Eco1520ORF67P	1563	1560	95.32	4	1563	8406	9965	0	2472
HVM2044	192	420937	Eco15ORF4165P	1197	16	100	280	295	150854	150869	1.2	32.2
HVM2289	54	10682	M1.EcoMI	1623	18	100	364	381	9781	9764	0.11	36.2
HVM2289	67	29057	M1.EcoMI	1191	16	100	1153	1168	5654	5669	1.2	32.2
HVM2289	58	171266	M.EcoMII	1620	19	100	1333	1351	2759	2741	0.027	38.2
HVM2289	58	171266	M.EcoMII	1620	20	95	1287	1306	85003	84984	1.7	32.2
HVM2289	222	102606	M.EcoMIII	1638	1638	100	1	1638	89694	91331	0	3247
HVM2289	222	102606	M.EcoMIII	1638	15	100	739	753	19901	19915	6.7	30.2
HVM2289	32	191498	M.EcoMIV	765	17	100	332	348	151236	151252	0.2	34.2
HVM2289	32	191498	M.EcoMIV	765	15	100	630	644	94648	94634	3.1	30.2
HVM2289	32	191498	M.EcoMIV	765	15	100	345	359	162398	162384	3.1	30.2
HVM2289	249	301	M.EcoMV	1050	322	86.34	86	407	324	3	3e-78	289
HVM2289	178	32646	M.EcoNwEVDcm	1419	1419	100	1	1419	23872	22454	0	2813
HVM2289	8	91645	M.EcoMVI	891	891	100	1	891	79798	80688	0	1725
HVM2289	9	191364	M.EcoNwEDam	837	837	100	1	837	151980	152816	0	1659
HVM2289	86	343027	M.EcoMVII	3606	25	96	596	620	218946	218970	0.004	42.1
HVM2289	86	343027	M.EcoMVII	3606	16	100	201	216	212130	212145	3.8	32.2
HVM2289	56	5799	M.EcoMVIII	684	684	97.37	1	684	2424	1741	0	1213
HVM2289	41	97417	Eco29kI	645	16	100	375	390	86002	85987	0.66	32.2
HVM2289	41	97417	Eco29kI	645	15	100	494	508	9809	9823	2.6	30.2
HVM2289	86	343027	EcoDEC4CORF2749P	1041	16	100	654	669	177539	177524	1.1	32.2
HVM2289	4	519848	Eco248534P	1053	16	100	941	956	202229	202214	1.1	32.2
HVM2289	4	519848	Eco248534P	1053	15	100	972	986	76396	76410	4.3	30.2
HVM2289	4	519848	Eco248534P	1053	15	100	66	80	206119	206105	4.3	30.2
HVM2289	57	6752	EcoAPECORF2077P	1590	19	100	312	330	3358	3340	0.027	38.2
HVM2289	175	106567	EcoDEC13EORF3046P	1191	16	100	192	207	106187	106202	1.2	32.2
HVM2289	56	5799	Eco7A8ORF29P	684	621	98.55	64	684	2361	1741	0	1160
HVM2289	19	159897	EcoDEC2CORF2043P	2019	21	95.24	1522	1542	158088	158068	0.53	34.2
HVM2289	19	159897	EcoDEC2CORF2043P	2019	16	100	124	139	21948	21963	2.1	32.2
HVM2289	19	159897	EcoDEC2CORF2043P	2019	15	100	1943	1957	115854	115868	8.3	30.2
HVM2289	206	390	Eco1886ORF14455P	1053	461	99.35	445	905	461	1	0	890
HVM2289	86	343027	Eco1886ORF14565P	900	15	100	565	579	138994	138980	3.7	30.2
HVM2289	56	5799	EcoR7ACORFAP	684	684	100	1	684	2424	1741	0	1356

HVM2289	56	5799	EcoR100ORF1P	648	621	98.55	28	648	2361	1741	0	1160
HVM2289	56	5799	Eco605ORFMP	684	684	94.44	1	684	2424	1741	0	1055
HVM2289	51	11209	Eco84137ORF201P	1635	27	92.59	811	837	5446	5420	0.027	38.2
HVM2289	51	11209	Eco84137ORF201P	1635	23	95.65	547	569	5689	5667	0.027	38.2
HVM2289	51	11209	Eco1520ORF67P	1563	1560	95.32	4	1563	6217	4658	0	2472
HVM2289	42	109768	Eco15ORF4165P	1197	16	100	280	295	63893	63878	1.2	32.2
S100EC	262	12846	M1.EcoMI	1623	1623	100	1	1623	9521	7899	0	3009
S100EC	262	12846	M1.EcoMI	1191	1191	100	1	1191	7906	6716	0	2361
S100EC	110	74046	M.EcoMII	1620	1581	100	40	1620	28446	30026	0	3092
S100EC	102	330384	M.EcoMIII	1638	1638	100	1	1638	257449	259086	0	3247
S100EC	102	330384	M.EcoMIII	1638	16	100	1336	1351	54399	54384	1.7	32.2
S100EC	102	330384	M.EcoMIII	1638	15	100	1509	1523	87222	87208	6.6	30.2
S100EC	102	330384	M.EcoMIII	1638	15	100	739	753	187656	187670	6.6	30.2
S100EC	102	330384	M.EcoMIII	1638	15	100	992	1006	262162	262176	6.6	30.2
S100EC	74	4737	M.EcoMIV	765	765	100	1	765	1558	2322	0	1516
S100EC	22	5774	M.EcoMV	1050	1050	100	1	1050	3981	2932	0	2040
S100EC	105	137773	M.EcoNwEVDcm	1419	1419	100	1	1419	30200	28782	0	2813
S100EC	155	358403	M.EcoMVI	891	891	100	1	891	346556	347446	0	1725
S100EC	155	358403	M.EcoMVI	891	16	100	232	247	154122	154137	0.91	32.2
S100EC	56	190860	M.EcoNwEDam	837	837	100	1	837	39437	38601	0	1659
S100EC	58	371354	M.EcoMVII	3606	25	96	596	620	114815	114791	0.004	42.1
S100EC	58	371354	M.EcoMVII	3606	16	100	201	216	121631	121616	3.7	32.2
S100EC	1	5706	M.EcoMVIII	684	684	100	1	684	3953	3270	0	1356
S100EC	255	199156	Eco29kI	645	16	100	238	253	46013	46028	0.65	32.2
S100EC	102	330384	EcoDEC4CORF2749P	1041	16	100	643	658	67574	67559	1.1	32.2
S100EC	92	11190	Eco248534P	1053	16	100	262	277	5531	5516	1.1	32.2
S100EC	155	358403	EcoAPECORF2077P	1590	18	100	1511	1528	260270	260253	0.1	36.2
S100EC	155	358403	EcoAPECORF2077P	1590	17	100	2	18	131573	131589	0.41	34.2
S100EC	155	358403	EcoAPECORF2077P	1590	17	100	1550	1566	312980	312964	0.41	34.2
S100EC	155	358403	EcoAPECORF2077P	1590	16	100	690	705	25406	25391	1.6	32.2
S100EC	155	358403	EcoAPECORF2077P	1590	15	100	811	825	68019	68005	6.4	30.2
S100EC	155	358403	EcoAPECORF2077P	1590	15	100	1552	1566	184551	184537	6.4	30.2
S100EC	155	358403	EcoAPECORF2077P	1590	19	94.74	1493	1511	286848	286830	6.4	30.2
S100EC	155	358403	EcoAPECORF2077P	1590	15	100	50	64	349137	349123	6.4	30.2
S100EC	105	137773	EcoDEC13EORF3046P	1191	16	100	920	935	40378	40393	1.2	32.2
S100EC	1	5706	Eco7A8ORF29P	684	606	98.51	79	684	3875	3270	0	1130

S100EC	58	371354	EcoDEC2CORF2043P	2019	21	95.24	1522	1542	335571	335591	0.52	34.2
S100EC	58	371354	EcoDEC2CORF2043P	2019	15	100	459	473	8353	8367	8.2	30.2
S100EC	58	371354	EcoDEC2CORF2043P	2019	19	94.74	1813	1831	31634	31616	8.2	30.2
S100EC	22	5774	Eco1886ORF14455P	1053	594	82.32	7	600	3975	3382	6e-95	345
S100EC	22	5774	Eco1886ORF14455P	1053	104	81.73	817	920	3165	3062	7e-08	56
S100EC	245	64479	Eco1886ORF14565P	900	15	100	289	303	39937	39951	3.6	30.2
S100EC	245	64479	Eco1886ORF14565P	900	15	100	273	287	59123	59109	3.6	30.2
S100EC	1	5706	EcoR7ACORFAP	684	684	97.37	1	684	3953	3270	0	1213
S100EC	1	5706	EcoR100ORF1P	648	606	98.51	43	648	3875	3270	0	1130
S100EC	1	5706	Eco605ORFMP	684	684	93.86	1	684	3953	3270	0	1023
S100EC	36	7561	Eco84137ORF201P	1635	27	92.59	811	837	5515	5489	0.027	38.2
S100EC	36	7561	Eco84137ORF201P	1635	20	95	550	569	5755	5736	1.7	32.2
S100EC	36	7561	Eco1520ORF67P	1563	1563	99.94	1	1563	6289	4727	0	3049
S100EC	262	12846	Eco15ORF4165P	1197	33	87.88	237	269	7232	7200	0.31	34.2
S110EC	228	14006	M1.EcoMI	1623	1623	100	1	1623	4539	6161	0	3009
S110EC	228	14006	M1.EcoMI	1191	1191	100	1	1191	6154	7344	0	2361
S110EC	15	71943	M.EcoMII	1620	1581	100	40	1620	27656	26076	0	3092
S110EC	29	65157	M.EcoMIII	1638	1638	100	1	1638	12398	14035	0	3247
S110EC	29	65157	M.EcoMIII	1638	15	100	992	1006	17111	17125	6.6	30.2
S110EC	74	28547	M.EcoMIV	765	765	100	1	765	4058	4822	0	1516
S110EC	131	18628	M.EcoMV	1050	1050	100	1	1050	14606	15655	0	2040
S110EC	230	35111	M.EcoNwEVDcm	1419	1419	100	1	1419	30197	28779	0	2813
S110EC	127	8096	M.EcoMVI	891	891	100	1	891	3868	4758	0	1725
S110EC	106	40178	M.EcoNwEDam	837	837	100	1	837	794	1630	0	1659
S110EC	176	42662	M.EcoMVII	3606	25	96	596	620	32172	32148	0.004	42.1
S110EC	176	42662	M.EcoMVII	3606	16	100	201	216	38988	38973	3.7	32.2
S110EC	217	5695	M.EcoMVIII	684	684	100	1	684	1830	2513	0	1356
S110EC	43	87402	Eco29kI	645	16	100	238	253	42163	42178	0.64	32.2
S110EC	251	14198	EcoDEC4CORF2749P	1041	16	100	654	669	270	255	1.1	32.2
S110EC	81	53372	Eco248534P	1053	16	100	941	956	51263	51248	1.1	32.2
S110EC	443	106856	EcoAPECORF2077P	1590	18	100	608	625	48696	48713	0.1	36.2
S110EC	443	106856	EcoAPECORF2077P	1590	17	100	1034	1050	42189	42205	0.41	34.2
S110EC	443	106856	EcoAPECORF2077P	1590	15	100	668	682	22354	22368	6.4	30.2
S110EC	443	106856	EcoAPECORF2077P	1590	15	100	1543	1557	28337	28351	6.4	30.2
S110EC	287	754	EcoDEC13EORF3046P	1191	16	100	920	935	800	815	1.2	32.2
S110EC	217	5695	Eco7A8ORF29P	684	606	98.51	79	684	1908	2513	0	1130

S110EC	164	23175	EcoDEC2CORF2043P	2019	21	95.24	1522	1542	13843	13863	0.52	34.2
S110EC	131	18628	Eco1886ORF14455P	1053	594	82.32	7	600	14612	15205	6e-95	345
S110EC	131	18628	Eco1886ORF14455P	1053	104	81.73	817	920	15422	15525	7e-08	56
S110EC	131	18628	Eco1886ORF14455P	1053	16	100	953	968	5248	5233	1.1	32.2
S110EC	256	5085	Eco1886ORF14565P	900	15	100	272	286	4829	4815	3.6	30.2
S110EC	217	5695	EcoR7ACORFAP	684	684	97.37	1	684	1830	2513	0	1213
S110EC	217	5695	EcoR100ORF1P	648	606	98.51	43	648	1908	2513	0	1130
S110EC	217	5695	Eco605ORFMP	684	684	93.86	1	684	1830	2513	0	1023
S110EC	168	7575	Eco84137ORF201P	1635	27	92.59	811	837	5529	5503	0.027	38.2
S110EC	168	7575	Eco84137ORF201P	1635	20	95	550	569	5769	5750	1.7	32.2
S110EC	168	7575	Eco1520ORF67P	1563	1563	99.94	1	1563	6303	4741	0	3049
S110EC	228	14006	Eco15ORF4165P	1197	33	87.88	237	269	6828	6860	0.31	34.2
S112EC	3	14002	M1.EcoMI	1623	1623	100	1	1623	9540	7918	0	3009
S112EC	3	14002	M1.EcoMI	1191	1191	100	1	1191	7925	6735	0	2361
S112EC	30	116442	M.EcoMII	1620	1581	100	40	1620	70736	72316	0	3092
S112EC	12	116855	M.EcoMIII	1638	1638	100	1	1638	72875	71238	0	3247
S112EC	12	116855	M.EcoMIII	1638	15	100	992	1006	68162	68148	6.6	30.2
S112EC	20	70212	M.EcoMIV	765	765	100	1	765	55159	54395	0	1516
S112EC	44	184732	M.EcoMV	1050	1050	100	1	1050	4095	3046	0	2040
S112EC	44	184732	M.EcoMV	1050	16	100	115	130	104915	104930	1.1	32.2
S112EC	69	20763	M.EcoNwEVDcm	1419	1419	100	1	1419	11988	10570	0	2813
S112EC	22	91686	M.EcoMVI	891	891	100	1	891	11920	11030	0	1725
S112EC	48	65061	M.EcoNwEDam	837	837	100	1	837	25677	26513	0	1659
S112EC	2	450887	M.EcoMVII	3606	25	96	596	620	160126	160150	0.004	42.1
S112EC	2	450887	M.EcoMVII	3606	17	100	3552	3568	438610	438626	0.94	34.2
S112EC	2	450887	M.EcoMVII	3606	16	100	201	216	153310	153325	3.7	32.2
S112EC	2	450887	M.EcoMVII	3606	16	100	815	830	391321	391336	3.7	32.2
S112EC	55	5726	M.EcoMVIII	684	684	100	1	684	1826	2509	0	1356
S112EC	47	70334	Eco29kI	645	16	100	375	390	11483	11498	0.65	32.2
S112EC	2	450887	EcoDEC4CORF2749P	1041	16	100	654	669	118719	118704	1.1	32.2
S112EC	2	450887	EcoDEC4CORF2749P	1041	16	100	643	658	352340	352325	1.1	32.2
S112EC	88	11283	Eco248534P	1053	16	100	262	277	5551	5536	1.1	32.2
S112EC	117	173760	EcoAPECORF2077P	1590	18	100	1494	1511	149535	149552	0.1	36.2
S112EC	117	173760	EcoAPECORF2077P	1590	16	100	827	842	63581	63596	1.6	32.2
S112EC	117	173760	EcoAPECORF2077P	1590	15	100	414	428	5325	5311	6.4	30.2
S112EC	192	148588	EcoDEC13EORF3046P	1191	16	100	192	207	148208	148223	1.2	32.2

S112EC	55	5726	Eco7A8ORF29P	684	606	98.51	79	684	1904	2509	0	1130
S112EC	16	34887	EcoDEC2CORF2043P	2019	21	95.24	1522	1542	17409	17389	0.52	34.2
S112EC	44	184732	Eco1886ORF14455P	1053	594	82.32	7	600	4089	3496	6e-95	345
S112EC	44	184732	Eco1886ORF14455P	1053	104	81.73	817	920	3279	3176	7e-08	56
S112EC	192	148588	Eco1886ORF14565P	900	15	100	74	88	9245	9231	3.6	30.2
S112EC	192	148588	Eco1886ORF14565P	900	15	100	67	81	36872	36886	3.6	30.2
S112EC	55	5726	EcoR7ACORFAP	684	684	97.37	1	684	1826	2509	0	1213
S112EC	55	5726	EcoR100ORF1P	648	606	98.51	43	648	1904	2509	0	1130
S112EC	55	5726	Eco605ORFMP	684	684	93.86	1	684	1826	2509	0	1023
S112EC	24	7570	Eco84137ORF201P	1635	27	92.59	811	837	5524	5498	0.027	38.2
S112EC	24	7570	Eco84137ORF201P	1635	20	95	550	569	5764	5745	1.7	32.2
S112EC	24	7570	Eco1520ORF67P	1563	1563	99.94	1	1563	6298	4736	0	3049
S112EC	3	14002	Eco15ORF4165P	1197	33	87.88	237	269	7251	7219	0.31	34.2
S102EC	90	13980	M1.EcoMI	1623	1623	100	1	1623	9518	7896	0	3009
S102EC	90	13980	M1.EcoMI	1191	1191	100	1	1191	7903	6713	0	2361
S102EC	184	74043	M.EcoMII	1620	1581	100	40	1620	45650	44070	0	3092
S102EC	18	98344	M.EcoMIII	1638	1638	100	1	1638	44034	45671	0	3247
S102EC	18	98344	M.EcoMIII	1638	15	100	992	1006	48747	48761	6.6	30.2
S102EC	256	4867	M.EcoMIV	765	765	100	1	765	3230	2466	0	1516
S102EC	139	6584	M.EcoMV	1050	1050	100	1	1050	2709	3758	0	2040
S102EC	23	137799	M.EcoNwEVDcm	1419	1419	100	1	1419	22370	20952	0	2813
S102EC	30	158985	M.EcoMVI	891	891	100	1	891	147138	148028	0	1725
S102EC	186	65039	M.EcoNwEDam	837	837	100	1	837	25655	26491	0	1659
S102EC	106	455837	M.EcoMVII	3606	25	96	596	620	341084	341108	0.004	42.1
S102EC	106	455837	M.EcoMVII	3606	16	100	3337	3352	83822	83837	3.7	32.2
S102EC	106	455837	M.EcoMVII	3606	16	100	201	216	334268	334283	3.7	32.2
S102EC	156	18073	M.EcoMVIII	684	684	100	1	684	10876	11559	0	1356
S102EC	24	222224	Eco29kI	645	16	100	238	253	176264	176249	0.64	32.2
S102EC	106	455837	EcoDEC4CORF2749P	1041	16	100	654	669	299677	299662	1	32.2
S102EC	125	11188	Eco248534P	1053	16	100	262	277	5529	5514	1.1	32.2
S102EC	43	173624	EcoAPECORF2077P	1590	18	100	1494	1511	149399	149416	0.1	36.2
S102EC	43	173624	EcoAPECORF2077P	1590	16	100	827	842	63445	63460	1.6	32.2
S102EC	43	173624	EcoAPECORF2077P	1590	15	100	414	428	5302	5288	6.4	30.2
S102EC	80	204989	EcoDEC13EORF3046P	1191	16	100	8	23	45310	45325	1.2	32.2
S102EC	80	204989	EcoDEC13EORF3046P	1191	16	100	192	207	204609	204624	1.2	32.2
S102EC	156	18073	Eco7A8ORF29P	684	606	98.51	79	684	10954	11559	0	1130

S102EC	106	455837	EcoDEC2CORF2043P	2019	21	95.24	1522	1542	120334	120314	0.52	34.2
S102EC	106	455837	EcoDEC2CORF2043P	2019	16	100	910	925	98205	98190	2.1	32.2
S102EC	106	455837	EcoDEC2CORF2043P	2019	19	94.74	1813	1831	424265	424283	8.1	30.2
S102EC	106	455837	EcoDEC2CORF2043P	2019	15	100	459	473	447546	447532	8.1	30.2
S102EC	139	6584	Eco1886ORF14455P	1053	594	82.32	7	600	2715	3308	5e-95	345
S102EC	139	6584	Eco1886ORF14455P	1053	104	81.73	817	920	3525	3628	7e-08	56
S102EC	303	229716	Eco1886ORF14565P	900	15	100	289	303	39929	39943	3.6	30.2
S102EC	303	229716	Eco1886ORF14565P	900	15	100	273	287	59115	59101	3.6	30.2
S102EC	156	18073	EcoR7ACORFAP	684	684	97.37	1	684	10876	11559	0	1213
S102EC	156	18073	EcoR100ORF1P	648	606	98.51	43	648	10954	11559	0	1130
S102EC	156	18073	Eco605ORFMP	684	684	93.86	1	684	10876	11559	0	1023
S102EC	105	5704	Eco84137ORF201P	1635	27	92.59	811	837	177	151	0.027	38.2
S102EC	105	5704	Eco84137ORF201P	1635	20	95	550	569	417	398	1.7	32.2
S102EC	105	5704	Eco1520ORF67P	1563	902	99.89	1	902	951	50	0	1739
S102EC	90	13980	Eco15ORF4165P	1197	33	87.88	237	269	7229	7197	0.31	34.2
P189EC	171	12848	M1.EcoMI	1623	1623	100	1	1623	9523	7901	0	3009
P189EC	171	12848	M1.EcoMI	1191	1191	100	1	1191	7908	6718	0	2361
P189EC	28	74048	M.EcoMII	1620	1581	100	40	1620	28448	30028	0	3092
P189EC	526	150329	M.EcoMIII	1638	1638	100	1	1638	72990	71353	0	3247
P189EC	526	150329	M.EcoMIII	1638	15	100	992	1006	68277	68263	6.8	30.2
P189EC	526	150329	M.EcoMIII	1638	15	100	739	753	142783	142769	6.8	30.2
P189EC	328	255340	M.EcoMIV	765	765	100	1	765	190072	189308	0	1516
P189EC	19	71694	M.EcoMV	1050	1050	100	1	1050	4077	3028	0	2040
P189EC	63	95155	M.EcoNwEVDcm	1419	1419	100	1	1419	30203	28785	0	2813
P189EC	2	158989	M.EcoMVI	891	891	100	1	891	147142	148032	0	1725
P189EC	14	190862	M.EcoNwEDam	837	837	100	1	837	39439	38603	0	1659
P189EC	94	347952	M.EcoMVII	3606	25	96	596	620	256482	256506	0.004	42.1
P189EC	94	347952	M.EcoMVII	3606	16	100	201	216	249778	249793	3.8	32.2
P189EC	230	1174	M.EcoMVIII	684	684	100	1	684	723	40	0	1356
P189EC	93	42541	Eco29kI	645	16	100	375	390	1033	1048	0.66	32.2
P189EC	94	347952	EcoDEC4CORF2749P	1041	16	100	654	669	215187	215172	1.1	32.2
P189EC	339	288645	Eco248534P	1053	16	100	941	956	36242	36257	1.1	32.2
P189EC	339	288645	Eco248534P	1053	15	100	66	80	32352	32366	4.3	30.2
P189EC	339	288645	Eco248534P	1053	15	100	972	986	162075	162061	4.3	30.2
P189EC	328	255340	EcoAPECORF2077P	1590	18	100	1260	1277	92996	93013	0.11	36.2
P189EC	328	255340	EcoAPECORF2077P	1590	17	100	99	115	72006	71990	0.42	34.2

P189EC	328	255340	EcoAPECORF2077P	1590	17	100	1	17	97358	97342	0.42	34.2
P189EC	328	255340	EcoAPECORF2077P	1590	15	100	65	79	9889	9903	6.6	30.2
P189EC	328	255340	EcoAPECORF2077P	1590	15	100	1025	1039	47941	47927	6.6	30.2
P189EC	328	255340	EcoAPECORF2077P	1590	15	100	669	683	137832	137818	6.6	30.2
P189EC	63	95155	EcoDEC13EORF3046P	1191	16	100	920	935	40381	40396	1.2	32.2
P189EC	230	1174	Eco7A8ORF29P	684	606	98.51	79	684	645	40	0	1130
P189EC	339	288645	EcoDEC2CORF2043P	2019	21	95.24	1522	1542	220895	220875	0.53	34.2
P189EC	339	288645	EcoDEC2CORF2043P	2019	15	100	1262	1276	270840	270854	8.4	30.2
P189EC	19	71694	Eco1886ORF14455P	1053	594	82.32	7	600	4071	3478	6e-95	345
P189EC	19	71694	Eco1886ORF14455P	1053	104	81.73	817	920	3261	3158	8e-08	56
P189EC	366	25846	Eco1886ORF14565P	900	15	100	50	64	19273	19259	3.7	30.2
P189EC	230	1174	EcoR7ACORFAP	684	684	97.37	1	684	723	40	0	1213
P189EC	230	1174	EcoR100ORF1P	648	606	98.51	43	648	645	40	0	1130
P189EC	230	1174	Eco605ORFMP	684	684	93.86	1	684	723	40	0	1023
P189EC	80	7563	Eco84137ORF201P	1635	27	92.59	811	837	5517	5491	0.028	38.2
P189EC	80	7563	Eco84137ORF201P	1635	20	95	550	569	5757	5738	1.7	32.2
P189EC	80	7563	Eco1520ORF67P	1563	1563	99.94	1	1563	6291	4729	0	3049
P189EC	171	12848	Eco15ORF4165P	1197	33	87.88	237	269	7234	7202	0.32	34.2
S92EC	23	12844	M1.EcoMI	1623	1623	100	1	1623	3376	4998	0	3009
S92EC	23	12844	M1.EcoMI	1191	1191	100	1	1191	4991	6181	0	2361
S92EC	121	116324	M.EcoMII	1620	1581	100	40	1620	70724	72304	0	3092
S92EC	2	308317	M.EcoMIII	1638	1638	100	1	1638	72853	71216	0	3247
S92EC	2	308317	M.EcoMIII	1638	16	100	1336	1351	275903	275918	1.7	32.2
S92EC	2	308317	M.EcoMIII	1638	15	100	992	1006	68140	68126	6.6	30.2
S92EC	2	308317	M.EcoMIII	1638	15	100	739	753	142646	142632	6.6	30.2
S92EC	2	308317	M.EcoMIII	1638	15	100	1509	1523	243080	243094	6.6	30.2
S92EC	82	188747	M.EcoMIV	765	765	100	1	765	123626	122862	0	1516
S92EC	20	21979	M.EcoMV	1050	1050	100	1	1050	3926	2877	0	2040
S92EC	108	38974	M.EcoNwEVDcm	1419	1419	100	1	1419	8826	10244	0	2813
S92EC	50	132143	M.EcoMVI	891	891	100	1	891	120296	121186	0	1725
S92EC	6	190858	M.EcoNwEDam	837	837	100	1	837	39435	38599	0	1659
S92EC	12	116576	M.EcoMVII	3606	25	96	596	620	91521	91497	0.004	42.1
S92EC	12	116576	M.EcoMVII	3606	16	100	201	216	98337	98322	3.7	32.2
S92EC	52	12496	M.EcoMVIII	684	684	100	1	684	10335	11018	0	1356
S92EC	100	92256	Eco29kI	645	16	100	375	390	6329	6344	0.65	32.2
S92EC	100	92256	Eco29kI	645	15	100	494	508	82520	82506	2.6	30.2

S92EC	79	230388	EcoDEC4CORF2749P	1041	16	100	654	669	16301	16316	1.1	32.2
S92EC	113	11187	Eco248534P	1053	16	100	262	277	5528	5513	1.1	32.2
S92EC	322	109181	EcoAPECORF2077P	1590	18	100	608	625	51080	51097	0.1	36.2
S92EC	322	109181	EcoAPECORF2077P	1590	17	100	1034	1050	44573	44589	0.41	34.2
S92EC	322	109181	EcoAPECORF2077P	1590	15	100	668	682	24738	24752	6.4	30.2
S92EC	322	109181	EcoAPECORF2077P	1590	15	100	1543	1557	30721	30735	6.4	30.2
S92EC	100	92256	EcoDEC13EORF3046P	1191	16	100	920	935	92287	92272	1.2	32.2
S92EC	52	12496	Eco7A8ORF29P	684	606	98.51	79	684	10413	11018	0	1130
S92EC	79	230388	EcoDEC2CORF2043P	2019	21	95.24	1522	1542	195646	195666	0.52	34.2
S92EC	20	21979	Eco1886ORF14455P	1053	594	82.32	7	600	3920	3327	6e-95	345
S92EC	20	21979	Eco1886ORF14455P	1053	104	81.73	817	920	3110	3007	7e-08	56
S92EC	94	229707	Eco1886ORF14565P	900	15	100	289	303	39933	39947	3.6	30.2
S92EC	94	229707	Eco1886ORF14565P	900	15	100	273	287	59117	59103	3.6	30.2
S92EC	52	12496	EcoR7ACORFAP	684	684	97.37	1	684	10335	11018	0	1213
S92EC	52	12496	EcoR100ORF1P	648	606	98.51	43	648	10413	11018	0	1130
S92EC	52	12496	Eco605ORFMP	684	684	93.86	1	684	10335	11018	0	1023
S92EC	76	3257	Eco84137ORF201P	1635	27	92.59	811	837	2097	2123	0.027	38.2
S92EC	76	3257	Eco84137ORF201P	1635	20	95	550	569	1857	1876	1.7	32.2
S92EC	76	3257	Eco1520ORF67P	1563	1563	99.94	1	1563	1323	2885	0	3049
S92EC	23	12844	Eco15ORF4165P	1197	33	87.88	237	269	5665	5697	0.31	34.2
S117EC	30	13979	M1.EcoMI	1623	1623	100	1	1623	9517	7895	0	3009
S117EC	30	13979	M1.EcoMI	1191	1191	100	1	1191	7902	6712	0	2361
S117EC	4	233762	M.EcoMII	1620	1581	100	40	1620	188162	189742	0	3092
S117EC	4	233762	M.EcoMII	1620	16	100	748	763	53193	53208	1.6	32.2
S117EC	4	233762	M.EcoMII	1620	15	100	1592	1606	41636	41650	6.5	30.2
S117EC	131	330108	M.EcoMIII	1638	1638	100	1	1638	257306	258943	0	3247
S117EC	131	330108	M.EcoMIII	1638	16	100	1336	1351	54256	54241	1.7	32.2
S117EC	131	330108	M.EcoMIII	1638	15	100	1509	1523	87079	87065	6.5	30.2
S117EC	131	330108	M.EcoMIII	1638	15	100	739	753	187513	187527	6.5	30.2
S117EC	131	330108	M.EcoMIII	1638	15	100	992	1006	262019	262033	6.5	30.2
S117EC	140	290706	M.EcoMIV	765	765	100	1	765	55004	55768	0	1516
S117EC	49	21985	M.EcoMV	1050	1050	100	1	1050	18110	19159	0	2040
S117EC	75	136407	M.EcoNwEVDcm	1419	1419	100	1	1419	127632	126214	0	2813
S117EC	2	34536	M.EcoMVI	891	891	100	1	891	11896	11006	0	1725
S117EC	91	190856	M.EcoNwEDam	837	837	100	1	837	151472	152308	0	1659
S117EC	5	208966	M.EcoMVII	3606	25	96	596	620	94204	94228	0.004	42.1

S117EC	5	208966	M.EcoMVII	3606	16	100	201	216	87388	87403	3.7	32.2
S117EC	74	9798	M.EcoMVIII	684	684	92.54	1	684	2913	2230	0	952
S117EC	277	242594	Eco29kI	645	16	100	238	253	196634	196619	0.64	32.2
S117EC	131	330108	EcoDEC4CORF2749P	1041	16	100	643	658	67431	67416	1	32.2
S117EC	168	185899	Eco248534P	1053	16	100	941	956	45854	45839	1.1	32.2
S117EC	168	185899	Eco248534P	1053	15	100	66	80	49744	49730	4.2	30.2
S117EC	140	290706	EcoAPECORF2077P	1590	18	100	1260	1277	152079	152062	0.1	36.2
S117EC	140	290706	EcoAPECORF2077P	1590	17	100	1	17	147717	147733	0.41	34.2
S117EC	140	290706	EcoAPECORF2077P	1590	17	100	99	115	173069	173085	0.41	34.2
S117EC	140	290706	EcoAPECORF2077P	1590	15	100	669	683	107243	107257	6.3	30.2
S117EC	140	290706	EcoAPECORF2077P	1590	15	100	1025	1039	197134	197148	6.3	30.2
S117EC	140	290706	EcoAPECORF2077P	1590	15	100	65	79	235186	235172	6.3	30.2
S117EC	75	136407	EcoDEC13EORF3046P	1191	16	100	920	935	18	33	1.2	32.2
S117EC	74	9798	Eco7A8ORF29P	684	621	92.91	64	684	2850	2230	0	882
S117EC	83	292365	EcoDEC2CORF2043P	2019	21	95.24	1522	1542	153511	153531	0.52	34.2
S117EC	83	292365	EcoDEC2CORF2043P	2019	16	100	703	718	78247	78232	2	32.2
S117EC	83	292365	EcoDEC2CORF2043P	2019	15	100	1262	1276	103566	103552	8.1	30.2
S117EC	49	21985	Eco1886ORF14455P	1053	594	82.32	7	600	18116	18709	5e-95	345
S117EC	49	21985	Eco1886ORF14455P	1053	104	81.73	817	920	18926	19029	7e-08	56
S117EC	168	185899	Eco1886ORF14565P	900	15	100	176	190	88938	88924	3.6	30.2
S117EC	168	185899	Eco1886ORF14565P	900	15	100	289	303	137724	137738	3.6	30.2
S117EC	168	185899	Eco1886ORF14565P	900	15	100	273	287	156908	156894	3.6	30.2
S117EC	74	9798	EcoR7ACORFAP	684	684	92.69	1	684	2913	2230	0	959
S117EC	74	9798	EcoR100ORF1P	648	621	92.91	28	648	2850	2230	0	882
S117EC	74	9798	Eco605ORFMP	684	684	97.37	1	684	2913	2230	0	1213
S117EC	12	68020	Eco84137ORF201P	1635	22	95.45	558	579	16643	16622	0.11	36.2
S117EC	151	164750	Eco1520ORF67P	1563	18	100	1501	1518	46435	46418	0.1	36.2
S117EC	151	164750	Eco1520ORF67P	1563	15	100	1239	1253	78993	79007	6.2	30.2
S117EC	30	13979	Eco15ORF4165P	1197	33	87.88	237	269	7228	7196	0.3	34.2
MS2493	210	12852	M1.EcoMI	1623	1623	100	1	1623	3384	5006	0	3009
MS2493	210	12852	M1.EcoMI	1191	1191	100	1	1191	4999	6189	0	2361
MS2493	170	74052	M.EcoMII	1620	1581	100	40	1620	45659	44079	0	3092
MS2493	20	113143	M.EcoMIII	1638	1638	100	1	1638	40341	41978	0	3247
MS2493	20	113143	M.EcoMIII	1638	15	100	992	1006	45054	45068	6.5	30.2
MS2493	30	300151	M.EcoMIV	765	765	100	1	765	234883	234119	0	1516
MS2493	22	185105	M.EcoMV	1050	1050	100	1	1050	4081	3032	0	2040

MS2493	22	185105	M.EcoMV	1050	16	100	115	130	104901	104916	1	32.2
MS2493	45	38974	M.EcoNwEVDcm	1419	1419	100	1	1419	8834	10252	0	2813
MS2493	117	158993	M.EcoMVI	891	891	100	1	891	11906	11016	0	1725
MS2493	8	190866	M.EcoNwEDam	837	837	100	1	837	39443	38607	0	1659
MS2493	175	348068	M.EcoMVII	3606	25	96	596	620	91529	91505	0.004	42.1
MS2493	175	348068	M.EcoMVII	3606	16	100	201	216	98345	98330	3.6	32.2
MS2493	62	17658	M.EcoMVIII	684	684	100	1	684	7311	6628	0	1356
MS2493	25	83597	Eco29kI	645	16	100	375	390	11484	11499	0.64	32.2
MS2493	175	348068	EcoDEC4CORF2749P	1041	16	100	654	669	132936	132951	1	32.2
MS2493	31	8502	Eco248534P	1053	16	100	262	277	5537	5522	1.1	32.2
MS2493	117	158993	EcoAPECORF2077P	1590	18	100	1511	1528	98192	98209	0.1	36.2
MS2493	117	158993	EcoAPECORF2077P	1590	17	100	1550	1566	45482	45498	0.4	34.2
MS2493	117	158993	EcoAPECORF2077P	1590	15	100	50	64	9325	9339	6.3	30.2
MS2493	117	158993	EcoAPECORF2077P	1590	19	94.74	1493	1511	71614	71632	6.3	30.2
MS2493	55	1375	EcoDEC13EORF3046P	1191	16	100	920	935	31	16	1.2	32.2
MS2493	62	17658	Eco7A8ORF29P	684	606	98.51	79	684	7233	6628	0	1130
MS2493	175	348068	EcoDEC2CORF2043P	2019	21	95.24	1522	1542	312285	312305	0.51	34.2
MS2493	175	348068	EcoDEC2CORF2043P	2019	19	94.74	1813	1831	8348	8330	8	30.2
MS2493	22	185105	Eco1886ORF14455P	1053	594	82.32	7	600	4075	3482	5e-95	345
MS2493	22	185105	Eco1886ORF14455P	1053	104	81.73	817	920	3265	3162	7e-08	56
MS2493	175	348068	Eco1886ORF14565P	900	15	100	565	579	171481	171495	3.5	30.2
MS2493	62	17658	EcoR7ACORFAP	684	684	97.37	1	684	7311	6628	0	1213
MS2493	62	17658	EcoR100ORF1P	648	606	98.51	43	648	7233	6628	0	1130
MS2493	62	17658	EcoR100ORF1P	648	18	94.44	312	329	3195	3178	10	28.2
MS2493	62	17658	Eco605ORFMP	684	684	93.86	1	684	7311	6628	0	1023
MS2493	47	7556	Eco84137ORF201P	1635	27	92.59	811	837	2105	2131	0.027	38.2
MS2493	47	7556	Eco84137ORF201P	1635	20	95	550	569	1865	1884	1.6	32.2
MS2493	47	7556	Eco1520ORF67P	1563	1563	99.94	1	1563	1331	2893	0	3049
MS2493	210	12852	Eco15ORF4165P	1197	33	87.88	237	269	5673	5705	0.3	34.2
HVM826	34	12317	M1.EcoMI	1623	1623	100	1	1623	3388	5010	0	3009
HVM826	34	12317	M1.EcoMI	1191	1191	100	1	1191	5003	6193	0	2361
HVM826	30	74096	M.EcoMII	1620	1581	100	40	1620	45664	44084	0	3092
HVM826	8	94465	M.EcoMIII	1638	1638	100	1	1638	21663	23300	0	3247
HVM826	8	94465	M.EcoMIII	1638	15	100	992	1006	26376	26390	6.6	30.2
HVM826	93	356555	M.EcoMIV	765	765	100	1	765	291287	290523	0	1516
HVM826	6	83784	M.EcoMV	1050	1050	100	1	1050	3410	2361	0	2040

HVM826	79	137756	M.EcoNwEVDcm	1419	1419	100	1	1419	30201	28783	0	2813
HVM826	221	158997	M.EcoMVI	891	891	100	1	891	11910	11020	0	1725
HVM826	37	55343	M.EcoNwEDam	837	837	100	1	837	15959	16795	0	1659
HVM826	42	393612	M.EcoMVII	3606	25	96	596	620	115141	115117	0.004	42.1
HVM826	42	393612	M.EcoMVII	3606	16	100	201	216	121957	121942	3.7	32.2
HVM826	140	13708	M.EcoMVIII	684	684	100	1	684	10391	11074	0	1356
HVM826	79	137756	Eco29kI	645	16	100	375	390	126338	126323	0.64	32.2
HVM826	79	137756	Eco29kI	645	15	100	494	508	50146	50160	2.5	30.2
HVM826	42	393612	EcoDEC4CORF2749P	1041	16	100	654	669	156548	156563	1.1	32.2
HVM826	49	11200	Eco248534P	1053	16	100	262	277	5722	5737	1.1	32.2
HVM826	221	158997	EcoAPECORF2077P	1590	18	100	1511	1528	98196	98213	0.1	36.2
HVM826	221	158997	EcoAPECORF2077P	1590	17	100	1550	1566	45486	45502	0.41	34.2
HVM826	221	158997	EcoAPECORF2077P	1590	15	100	50	64	9329	9343	6.4	30.2
HVM826	221	158997	EcoAPECORF2077P	1590	19	94.74	1493	1511	71618	71636	6.4	30.2
HVM826	79	137756	EcoDEC13EORF3046P	1191	16	100	920	935	40379	40394	1.2	32.2
HVM826	140	13708	Eco7A8ORF29P	684	606	98.51	79	684	10469	11074	0	1130
HVM826	229	178470	EcoDEC2CORF2043P	2019	21	95.24	1522	1542	25171	25151	0.52	34.2
HVM826	229	178470	EcoDEC2CORF2043P	2019	16	100	703	718	100435	100450	2.1	32.2
HVM826	229	178470	EcoDEC2CORF2043P	2019	15	100	1262	1276	75116	75130	8.1	30.2
HVM826	6	83784	Eco1886ORF14455P	1053	594	82.32	7	600	3404	2811	5e-95	345
HVM826	6	83784	Eco1886ORF14455P	1053	104	81.73	817	920	2594	2491	7e-08	56
HVM826	93	356555	Eco1886ORF14565P	900	15	100	831	845	18749	18735	3.6	30.2
HVM826	140	13708	EcoR7ACORFAP	684	684	97.37	1	684	10391	11074	0	1213
HVM826	140	13708	EcoR100ORF1P	648	606	98.51	43	648	10469	11074	0	1130
HVM826	177	3415	Eco605ORFMP	684	684	96.49	1	684	111	794	0	1166
HVM826	38	7572	Eco84137ORF201P	1635	27	92.59	811	837	2109	2135	0.027	38.2
HVM826	38	7572	Eco84137ORF201P	1635	20	95	550	569	1869	1888	1.7	32.2
HVM826	38	7572	Eco1520ORF67P	1563	1563	99.94	1	1563	1335	2897	0	3049
HVM826	34	12317	Eco15ORF4165P	1197	33	87.88	237	269	5677	5709	0.31	34.2
P53EC	306	12846	M1.EcoMI	1623	1623	100	1	1623	9521	7899	0	3009
P53EC	306	12846	M1.EcoMI	1191	1191	100	1	1191	7906	6716	0	2361
P53EC	299	74046	M.EcoMII	1620	1581	100	40	1620	28446	30026	0	3092
P53EC	21	124262	M.EcoMIII	1638	1638	100	1	1638	51460	53097	0	3247
P53EC	21	124262	M.EcoMIII	1638	15	100	992	1006	56173	56187	6.8	30.2
P53EC	84	300930	M.EcoMIV	765	765	100	1	765	65227	65991	0	1516
P53EC	49	186841	M.EcoMV	1050	1050	100	1	1050	3981	2932	0	2040

P53EC	49	186841	M.EcoMV	1050	16	100	115	130	104801	104816	1.1	32.2
P53EC	110	136418	M.EcoNwEVDcm	1419	1419	100	1	1419	8828	10246	0	2813
P53EC	2	158987	M.EcoMVI	891	891	100	1	891	147140	148030	0	1725
P53EC	54	190860	M.EcoNwEDam	837	837	100	1	837	151476	152312	0	1659
P53EC	7	189648	M.EcoMVII	3606	25	96	596	620	114814	114790	0.004	42.1
P53EC	7	189648	M.EcoMVII	3606	16	100	201	216	121630	121615	3.8	32.2
P53EC	411	13212	M.EcoMVIII	684	684	100	1	684	2928	2245	0	1356
P53EC	110	136418	Eco29kI	645	16	100	375	390	50490	50505	0.66	32.2
P53EC	110	136418	Eco29kI	645	15	100	494	508	126682	126668	2.6	30.2
P53EC	37	3188	EcoDEC4CORF2749P	1041	16	100	84	99	1267	1252	1.1	32.2
P53EC	344	634735	Eco248534P	1053	16	100	941	956	353088	353073	1.1	32.2
P53EC	344	634735	Eco248534P	1053	15	100	972	986	227256	227270	4.3	30.2
P53EC	344	634735	Eco248534P	1053	15	100	66	80	356978	356964	4.3	30.2
P53EC	84	300930	EcoAPECORF2077P	1590	18	100	1260	1277	162303	162286	0.11	36.2
P53EC	84	300930	EcoAPECORF2077P	1590	17	100	1	17	157941	157957	0.42	34.2
P53EC	84	300930	EcoAPECORF2077P	1590	17	100	99	115	183293	183309	0.42	34.2
P53EC	84	300930	EcoAPECORF2077P	1590	15	100	669	683	117467	117481	6.6	30.2
P53EC	84	300930	EcoAPECORF2077P	1590	15	100	1025	1039	207358	207372	6.6	30.2
P53EC	84	300930	EcoAPECORF2077P	1590	15	100	65	79	245410	245396	6.6	30.2
P53EC	339	1381	EcoDEC13EORF3046P	1191	16	100	920	935	1403	1418	1.2	32.2
P53EC	411	13212	Eco7A8ORF29P	684	606	98.51	79	684	2850	2245	0	1130
P53EC	418	181318	EcoDEC2CORF2043P	2019	21	95.24	1522	1542	145535	145555	0.53	34.2
P53EC	49	186841	Eco1886ORF14455P	1053	594	82.32	7	600	3975	3382	6e-95	345
P53EC	49	186841	Eco1886ORF14455P	1053	104	81.73	817	920	3165	3062	8e-08	56
P53EC	449	133297	Eco1886ORF14565P	900	15	100	884	898	61975	61989	3.7	30.2
P53EC	411	13212	EcoR7ACORFAP	684	684	97.37	1	684	2928	2245	0	1213
P53EC	411	13212	EcoR100ORF1P	648	606	98.51	43	648	2850	2245	0	1130
P53EC	411	13212	Eco605ORFMP	684	684	93.86	1	684	2928	2245	0	1023
P53EC	239	7561	Eco84137ORF201P	1635	27	92.59	811	837	5515	5489	0.028	38.2
P53EC	239	7561	Eco84137ORF201P	1635	20	95	550	569	5755	5736	1.7	32.2
P53EC	239	7561	Eco1520ORF67P	1563	1563	99.94	1	1563	6289	4727	0	3049
P53EC	306	12846	Eco15ORF4165P	1197	33	87.88	237	269	7232	7200	0.32	34.2
S135EC	246	14007	M1.EcoMI	1623	1623	100	1	1623	4539	6161	0	3009
S135EC	246	14007	M1.EcoMI	1191	1191	100	1	1191	6154	7344	0	2361
S135EC	58	47706	M.EcoMII	1620	1581	100	40	1620	10213	11793	0	3092
S135EC	367	112866	M.EcoMIII	1638	1638	100	1	1638	39931	41568	0	3247

S135EC	367	112866	M.EcoMIII	1638	15	100	992	1006	44644	44658	6.8	30.2
S135EC	67	53921	M.EcoMIV	765	765	100	1	765	15586	16350	0	1516
S135EC	90	187034	M.EcoMV	1050	1050	100	1	1050	183012	184061	0	2040
S135EC	90	187034	M.EcoMV	1050	16	100	115	130	82192	82177	1.1	32.2
S135EC	36	20366	M.EcoNwEVDcm	1419	1419	100	1	1419	8852	10270	0	2813
S135EC	19	62477	M.EcoMVI	891	891	100	1	891	50630	51520	0	1725
S135EC	110	43074	M.EcoNwEDam	837	837	100	1	837	10766	9930	0	1659
S135EC	55	177369	M.EcoMVII	3606	25	96	596	620	113921	113897	0.004	42.1
S135EC	55	177369	M.EcoMVII	3606	16	100	201	216	120737	120722	3.8	32.2
S135EC	2	5730	M.EcoMVIII	684	684	100	1	684	3977	3294	0	1356
S135EC	16	136513	Eco29kI	645	16	100	238	253	46037	46052	0.66	32.2
S135EC	55	177369	EcoDEC4CORF2749P	1041	16	100	654	669	155328	155343	1.1	32.2
S135EC	95	11286	Eco248534P	1053	16	100	262	277	5554	5539	1.1	32.2
S135EC	248	79380	EcoAPECORF2077P	1590	18	100	608	625	28231	28214	0.11	36.2
S135EC	248	79380	EcoAPECORF2077P	1590	17	100	1034	1050	34738	34722	0.42	34.2
S135EC	248	79380	EcoAPECORF2077P	1590	15	100	1543	1557	48590	48576	6.6	30.2
S135EC	248	79380	EcoAPECORF2077P	1590	15	100	668	682	54573	54559	6.6	30.2
S135EC	218	136	EcoDEC13EORF3046P	1191	16	100	920	935	167	152	1.2	32.2
S135EC	2	5730	Eco7A8ORF29P	684	606	98.51	79	684	3899	3294	0	1130
S135EC	53	161384	EcoDEC2CORF2043P	2019	21	95.24	1522	1542	34228	34208	0.54	34.2
S135EC	90	187034	Eco1886ORF14455P	1053	594	82.32	7	600	183018	183611	6e-95	345
S135EC	90	187034	Eco1886ORF14455P	1053	104	81.73	817	920	183828	183931	8e-08	56
S135EC	315	54856	Eco1886ORF14565P	900	15	100	273	287	8742	8756	3.7	30.2
S135EC	315	54856	Eco1886ORF14565P	900	15	100	289	303	27928	27914	3.7	30.2
S135EC	2	5730	EcoR7ACORFAP	684	684	97.37	1	684	3977	3294	0	1213
S135EC	2	5730	EcoR100ORF1P	648	606	98.51	43	648	3899	3294	0	1130
S135EC	2	5730	Eco605ORFMP	684	684	93.86	1	684	3977	3294	0	1023
S135EC	101	47010	Eco84137ORF201P	1635	22	95.45	558	579	41611	41590	0.11	36.2
S135EC	97	1368	Eco1520ORF67P	1563	22	100	1	22	96	75	4e-04	44.1
S135EC	246	14007	Eco15ORF4165P	1197	33	87.88	237	269	6828	6860	0.32	34.2
S107EC	100	10095	M1.EcoMI	1623	1623	100	1	1623	5633	4011	0	3009
S107EC	100	10095	M1.EcoMI	1191	1191	100	1	1191	4018	2828	0	2361
S107EC	195	83238	M.EcoMII	1620	1581	100	40	1620	59268	60848	0	3092
S107EC	29	100176	M.EcoMIII	1638	1638	100	1	1638	72997	71360	0	3247
S107EC	29	100176	M.EcoMIII	1638	15	100	992	1006	68284	68270	6.5	30.2
S107EC	17	17145	M.EcoMIV	765	765	100	1	765	1959	2723	0	1516

S107EC	44	44462	M.EcoMV	1050	1050	100	1	1050	5769	4720	0	2040
S107EC	76	38980	M.EcoNwEVDcm	1419	1419	100	1	1419	8850	10268	0	2813
S107EC	81	15494	M.EcoMVI	891	891	100	1	891	11922	11032	0	1725
S107EC	55	135370	M.EcoNwEDam	837	837	100	1	837	39459	38623	0	1659
S107EC	88	91284	M.EcoMVII	3606	25	96	596	620	20237	20261	0.004	42.1
S107EC	88	91284	M.EcoMVII	3606	16	100	201	216	13421	13436	3.7	32.2
S107EC	164	5728	M.EcoMVIII	684	684	100	1	684	3975	3292	0	1356
S107EC	153	12276	Eco29kI	645	16	100	375	390	877	862	0.64	32.2
S107EC	64	63413	EcoDEC4CORF2749P	1041	16	100	654	669	42226	42211	1	32.2
S107EC	71	11286	Eco248534P	1053	16	100	262	277	5554	5539	1.1	32.2
S107EC	195	83238	EcoAPECORF2077P	1590	19	100	312	330	26740	26758	0.026	38.2
S107EC	195	83238	EcoAPECORF2077P	1590	15	100	266	280	17408	17422	6.3	30.2
S107EC	195	83238	EcoAPECORF2077P	1590	15	100	265	279	63962	63948	6.3	30.2
S107EC	144	756	EcoDEC13EORF3046P	1191	16	100	920	935	800	815	1.2	32.2
S107EC	164	5728	Eco7A8ORF29P	684	606	98.51	79	684	3897	3292	0	1130
S107EC	13	25406	EcoDEC2CORF2043P	2019	21	95.24	1522	1542	17563	17583	0.52	34.2
S107EC	44	44462	Eco1886ORF14455P	1053	594	82.32	7	600	5763	5170	5e-95	345
S107EC	44	44462	Eco1886ORF14455P	1053	104	81.73	817	920	4953	4850	7e-08	56
S107EC	220	85870	Eco1886ORF14565P	900	15	100	289	303	39964	39978	3.6	30.2
S107EC	220	85870	Eco1886ORF14565P	900	15	100	273	287	59150	59136	3.6	30.2
S107EC	164	5728	EcoR7ACORFAP	684	684	97.37	1	684	3975	3292	0	1213
S107EC	164	5728	EcoR100ORF1P	648	606	98.51	43	648	3897	3292	0	1130
S107EC	164	5728	Eco605ORFMP	684	684	93.86	1	684	3975	3292	0	1023
S107EC	165	2514	Eco84137ORF201P	1635	27	92.59	811	837	2121	2147	0.027	38.2
S107EC	165	2514	Eco84137ORF201P	1635	20	95	550	569	1881	1900	1.7	32.2
S107EC	165	2514	Eco1520ORF67P	1563	1168	99.91	1	1168	1347	2514	0	2266
S107EC	100	10095	Eco15ORF4165P	1197	33	87.88	237	269	3344	3312	0.31	34.2
S108EC	95	6103	M1.EcoMI	1623	1623	100	1	1623	3512	1890	0	3009
S108EC	95	6103	M1.EcoMI	1191	1191	100	1	1191	1897	707	0	2361
S108EC	16	61751	M.EcoMII	1620	1581	100	40	1620	33358	31778	0	3092
S108EC	106	124675	M.EcoMIII	1638	1638	100	1	1638	72996	71359	0	3247
S108EC	106	124675	M.EcoMIII	1638	15	100	992	1006	68283	68269	6.6	30.2
S108EC	156	596510	M.EcoMIV	765	765	100	1	765	235764	235000	0	1516
S108EC	4	85748	M.EcoMV	1050	1050	100	1	1050	5755	4706	0	2040
S108EC	141	38977	M.EcoNwEVDcm	1419	1419	100	1	1419	8836	10254	0	2813
S108EC	18	91663	M.EcoMVI	891	891	100	1	891	79826	80716	0	1725

S108EC	35	190868	M.EcoNwEDam	837	837	100	1	837	39445	38609	0	1659
S108EC	156	596510	M.EcoMVII	3606	25	96	596	620	525463	525487	0.004	42.1
S108EC	156	596510	M.EcoMVII	3606	16	100	3337	3352	83832	83847	3.7	32.2
S108EC	156	596510	M.EcoMVII	3606	16	100	201	216	518647	518662	3.7	32.2
S108EC	97	5714	M.EcoMVIII	684	684	100	1	684	3961	3278	0	1356
S108EC	143	47600	Eco29kI	645	16	100	375	390	11494	11509	0.65	32.2
S108EC	156	596510	EcoDEC4CORF2749P	1041	16	100	654	669	484056	484041	1.1	32.2
S108EC	237	389936	Eco248534P	1053	16	100	941	956	51984	51999	1.1	32.2
S108EC	237	389936	Eco248534P	1053	15	100	66	80	48094	48108	4.2	30.2
S108EC	237	389936	Eco248534P	1053	15	100	972	986	177817	177803	4.2	30.2
S108EC	156	596510	EcoAPECORF2077P	1590	18	100	1260	1277	138688	138705	0.1	36.2
S108EC	156	596510	EcoAPECORF2077P	1590	17	100	99	115	117698	117682	0.41	34.2
S108EC	156	596510	EcoAPECORF2077P	1590	17	100	1	17	143050	143034	0.41	34.2
S108EC	156	596510	EcoAPECORF2077P	1590	17	100	930	946	510456	510472	0.41	34.2
S108EC	156	596510	EcoAPECORF2077P	1590	15	100	65	79	55581	55595	6.4	30.2
S108EC	156	596510	EcoAPECORF2077P	1590	15	100	1025	1039	93633	93619	6.4	30.2
S108EC	156	596510	EcoAPECORF2077P	1590	15	100	669	683	183524	183510	6.4	30.2
S108EC	156	596510	EcoAPECORF2077P	1590	15	100	1401	1415	339865	339851	6.4	30.2
S108EC	156	596510	EcoAPECORF2077P	1590	15	100	1436	1450	424237	424251	6.4	30.2
S108EC	25	43157	EcoDEC13EORF3046P	1191	23	95.65	377	399	31956	31978	0.02	38.2
S108EC	97	5714	Eco7A8ORF29P	684	606	98.51	79	684	3883	3278	0	1130
S108EC	237	389936	EcoDEC2CORF2043P	2019	21	95.24	1522	1542	236637	236617	0.52	34.2
S108EC	237	389936	EcoDEC2CORF2043P	2019	16	100	703	718	311901	311916	2.1	32.2
S108EC	237	389936	EcoDEC2CORF2043P	2019	15	100	1262	1276	286582	286596	8.1	30.2
S108EC	4	85748	Eco1886ORF14455P	1053	594	82.32	7	600	5749	5156	6e-95	345
S108EC	4	85748	Eco1886ORF14455P	1053	104	81.73	817	920	4939	4836	7e-08	56
S108EC	237	389936	Eco1886ORF14565P	900	15	100	176	190	8900	8914	3.6	30.2
S108EC	97	5714	EcoR7ACORFAP	684	684	97.37	1	684	3961	3278	0	1213
S108EC	97	5714	EcoR100ORF1P	648	606	98.51	43	648	3883	3278	0	1130
S108EC	97	5714	Eco605ORFMP	684	684	93.86	1	684	3961	3278	0	1023
S108EC	72	7558	Eco84137ORF201P	1635	27	92.59	811	837	2107	2133	0.027	38.2
S108EC	72	7558	Eco84137ORF201P	1635	20	95	550	569	1867	1886	1.7	32.2
S108EC	72	7558	Eco1520ORF67P	1563	1563	99.94	1	1563	1333	2895	0	3049
S108EC	95	6103	Eco15ORF4165P	1197	33	87.88	237	269	1223	1191	0.31	34.2
P146EC	75	10114	M1.EcoMI	1623	1623	100	1	1623	9539	7917	0	3009
P146EC	75	10114	M1.EcoMI	1191	1191	100	1	1191	7924	6734	0	2361

P146EC	30	341888	M.EcoMII	1620	1581	100	40	1620	45671	44091	0	3092
P146EC	30	341888	M.EcoMII	1620	16	100	748	763	180914	180899	1.7	32.2
P146EC	30	341888	M.EcoMII	1620	15	100	1592	1606	192471	192457	6.6	30.2
P146EC	20	85985	M.EcoMIII	1638	1638	100	1	1638	13183	14820	0	3247
P146EC	20	85985	M.EcoMIII	1638	15	100	992	1006	17896	17910	6.7	30.2
P146EC	16	174488	M.EcoMIV	765	765	100	1	765	103507	102743	0	1516
P146EC	1	94718	M.EcoMV	1050	1050	100	1	1050	91867	92916	0	2040
P146EC	22	24737	M.EcoNwEVDcm	1419	1419	100	1	1419	15962	14544	0	2813
P146EC	5	159005	M.EcoMVI	891	891	100	1	891	11918	11028	0	1725
P146EC	49	190878	M.EcoNwEDam	837	837	100	1	837	39455	38619	0	1659
P146EC	189	317593	M.EcoMVII	3606	25	96	596	620	61056	61032	0.004	42.1
P146EC	189	317593	M.EcoMVII	3606	16	100	201	216	67872	67857	3.8	32.2
P146EC	340	4098	M.EcoMVIII	684	684	100	1	684	1355	2038	0	1356
P146EC	140	9092	Eco29kI	645	16	100	375	390	5981	5966	0.66	32.2
P146EC	189	317593	EcoDEC4CORF2749P	1041	16	100	654	669	102463	102478	1.1	32.2
P146EC	11	11208	Eco248534P	1053	16	100	262	277	5730	5745	1.1	32.2
P146EC	307	56324	EcoAPECORF2077P	1590	19	100	312	330	32643	32625	0.027	38.2
P146EC	307	56324	EcoAPECORF2077P	1590	15	100	1314	1328	20111	20125	6.5	30.2
P146EC	307	56324	EcoAPECORF2077P	1590	15	100	724	738	35693	35707	6.5	30.2
P146EC	338	662	EcoDEC13EORF3046P	1191	16	100	920	935	702	717	1.2	32.2
P146EC	340	4098	Eco7A8ORF29P	684	606	98.51	79	684	1433	2038	0	1130
P146EC	189	317593	EcoDEC2CORF2043P	2019	21	95.24	1522	1542	281810	281830	0.53	34.2
P146EC	307	56324	Eco1886ORF14455P	1053	362	97.79	692	1053	38517	38878	2e-175	613
P146EC	307	56324	Eco1886ORF14565P	900	900	100	1	900	23072	22173	0	1742
P146EC	340	4098	EcoR7ACORFAP	684	684	97.37	1	684	1355	2038	0	1213
P146EC	340	4098	EcoR100ORF1P	648	606	98.51	43	648	1433	2038	0	1130
P146EC	170	1184	Eco605ORFMP	684	684	99.85	1	684	686	3	0	1348
P146EC	67	7579	Eco84137ORF201P	1635	27	92.59	811	837	2117	2143	0.027	38.2
P146EC	67	7579	Eco84137ORF201P	1635	20	95	550	569	1877	1896	1.7	32.2
P146EC	67	7579	Eco1520ORF67P	1563	1563	99.87	1	1563	1343	2904	0	3033
P146EC	75	10114	Eco15ORF4165P	1197	33	87.88	237	269	7250	7218	0.31	34.2
S116EC	97	13122	M1.EcoMI	1623	1623	100	1	1623	9545	7923	0	3009
S116EC	97	13122	M1.EcoMI	1191	1191	100	1	1191	7930	6740	0	2361
S116EC	10	116436	M.EcoMII	1620	1581	100	40	1620	70836	72416	0	3092
S116EC	281	86118	M.EcoMIII	1638	1638	100	1	1638	73012	71375	0	3247
S116EC	281	86118	M.EcoMIII	1638	15	100	992	1006	68299	68285	6.5	30.2

S116EC	50	85325	M.EcoMIV	765	765	100	1	765	65345	66109	0	1516
S116EC	44	57341	M.EcoMV	1050	1050	100	1	1050	53319	54368	0	2040
S116EC	141	38972	M.EcoNwEVDcm	1419	1419	100	1	1419	30197	28779	0	2813
S116EC	13	91673	M.EcoMVI	891	891	100	1	891	11924	11034	0	1725
S116EC	27	191369	M.EcoNwEDam	837	837	100	1	837	39461	38625	0	1659
S116EC	30	117996	M.EcoMVII	3606	25	96	596	620	54850	54874	0.004	42.1
S116EC	30	117996	M.EcoMVII	3606	16	100	201	216	48034	48049	3.6	32.2
S116EC	113	8454	M.EcoMVIII	684	684	100	1	684	6322	7005	0	1356
S116EC	14	215905	Eco29kI	645	16	100	238	253	46037	46052	0.63	32.2
S116EC	14	215905	Eco29kI	645	18	94.44	210	227	17750	17733	9.9	28.2
S116EC	30	117996	EcoDEC4CORF2749P	1041	16	100	654	669	13443	13428	1	32.2
S116EC	1	64161	Eco248534P	1053	16	100	941	956	14723	14708	1	32.2
S116EC	1	64161	Eco248534P	1053	15	100	66	80	18613	18599	4.1	30.2
S116EC	227	90449	EcoAPECORF2077P	1590	18	100	608	625	39115	39098	0.1	36.2
S116EC	227	90449	EcoAPECORF2077P	1590	17	100	1034	1050	45622	45606	0.4	34.2
S116EC	227	90449	EcoAPECORF2077P	1590	15	100	1543	1557	59480	59466	6.3	30.2
S116EC	227	90449	EcoAPECORF2077P	1590	15	100	668	682	65463	65449	6.3	30.2
S116EC	159	656	EcoDEC13EORF3046P	1191	16	100	920	935	31	16	1.2	32.2
S116EC	113	8454	Eco7A8ORF29P	684	606	98.51	79	684	6400	7005	0	1130
S116EC	29	54266	EcoDEC2CORF2043P	2019	21	95.24	1522	1542	973	993	0.51	34.2
S116EC	44	57341	Eco1886ORF14455P	1053	594	82.32	7	600	53325	53918	5e-95	345
S116EC	44	57341	Eco1886ORF14455P	1053	104	81.73	817	920	54135	54238	7e-08	56
S116EC	85	54080	Eco1886ORF14565P	900	15	100	884	898	36945	36931	3.5	30.2
S116EC	113	8454	EcoR7ACORFAP	684	684	97.37	1	684	6322	7005	0	1213
S116EC	113	8454	EcoR100ORF1P	648	606	98.51	43	648	6400	7005	0	1130
S116EC	113	8454	Eco605ORFMP	684	684	93.86	1	684	6322	7005	0	1023
S116EC	52	18171	Eco84137ORF201P	1635	22	95.45	558	579	16671	16650	0.1	36.2
S116EC	212	1916	Eco1520ORF67P	1563	569	100	1	569	644	76	0	1128
S116EC	97	13122	Eco15ORF4165P	1197	33	87.88	237	269	7256	7224	0.3	34.2
P56EC	46	12840	M1.EcoMI	1623	1623	100	1	1623	3372	4994	0	3009
P56EC	46	12840	M1.EcoMI	1191	1191	100	1	1191	4987	6177	0	2361
P56EC	119	74040	M.EcoMII	1620	1581	100	40	1620	28440	30020	0	3092
P56EC	41	307886	M.EcoMIII	1638	1638	100	1	1638	72849	71212	0	3247
P56EC	41	307886	M.EcoMIII	1638	16	100	1336	1351	275899	275914	1.7	32.2
P56EC	41	307886	M.EcoMIII	1638	15	100	992	1006	68136	68122	6.6	30.2
P56EC	41	307886	M.EcoMIII	1638	15	100	739	753	142642	142628	6.6	30.2

P56EC	41	307886	M.EcoMIII	1638	15	100	1509	1523	243076	243090	6.6	30.2
P56EC	175	4877	M.EcoMIV	765	765	100	1	765	1698	2462	0	1516
P56EC	91	102920	M.EcoMV	1050	1050	100	1	1050	11124	10075	0	2040
P56EC	221	33476	M.EcoNwEVDcm	1419	1419	100	1	1419	24701	23283	0	2813
P56EC	44	127337	M.EcoMVI	891	891	100	1	891	11894	11004	0	1725
P56EC	71	190854	M.EcoNwEDam	837	837	100	1	837	39431	38595	0	1659
P56EC	281	335559	M.EcoMVII	3606	25	96	596	620	220797	220821	0.004	42.1
P56EC	281	335559	M.EcoMVII	3606	16	100	201	216	213981	213996	3.7	32.2
P56EC	291	17546	M.EcoMVIII	684	684	100	1	684	7262	6579	0	1356
P56EC	323	41043	Eco29kI	645	16	100	375	390	29611	29596	0.64	32.2
P56EC	281	335559	EcoDEC4CORF2749P	1041	16	100	654	669	179390	179375	1	32.2
P56EC	248	11184	Eco248534P	1053	16	100	262	277	5525	5510	1.1	32.2
P56EC	98	53385	EcoAPECORF2077P	1590	18	100	1494	1511	24272	24255	0.1	36.2
P56EC	97	56322	EcoDEC13EORF3046P	1191	16	100	920	935	56353	56338	1.2	32.2
P56EC	291	17546	Eco7A8ORF29P	684	606	98.51	79	684	7184	6579	0	1130
P56EC	281	335559	EcoDEC2CORF2043P	2019	21	95.24	1522	1542	44	24	0.52	34.2
P56EC	281	335559	EcoDEC2CORF2043P	2019	19	94.74	1813	1831	303978	303996	8.1	30.2
P56EC	281	335559	EcoDEC2CORF2043P	2019	15	100	459	473	327259	327245	8.1	30.2
P56EC	91	102920	Eco1886ORF14455P	1053	594	82.32	7	600	11118	10525	5e-95	345
P56EC	91	102920	Eco1886ORF14455P	1053	104	81.73	817	920	10308	10205	7e-08	56
P56EC	281	335559	Eco1886ORF14565P	900	15	100	565	579	140845	140831	3.6	30.2
P56EC	291	17546	EcoR7ACORFAP	684	684	97.37	1	684	7262	6579	0	1213
P56EC	291	17546	EcoR100ORF1P	648	606	98.51	43	648	7184	6579	0	1130
P56EC	291	17546	Eco605ORFMP	684	684	93.86	1	684	7262	6579	0	1023
P56EC	68	7543	Eco84137ORF201P	1635	27	92.59	811	837	5497	5471	0.027	38.2
P56EC	68	7543	Eco84137ORF201P	1635	20	95	550	569	5737	5718	1.7	32.2
P56EC	68	7543	Eco1520ORF67P	1563	1563	99.94	1	1563	6271	4709	0	3049
P56EC	46	12840	Eco15ORF4165P	1197	33	87.88	237	269	5661	5693	0.31	34.2
S95EC	7	12868	M1.EcoMI	1623	1623	100	1	1623	9543	7921	0	3009
S95EC	7	12868	M1.EcoMI	1191	1191	100	1	1191	7928	6738	0	2361
S95EC	33	116347	M.EcoMII	1620	1581	100	40	1620	70747	72327	0	3092
S95EC	221	231860	M.EcoMIII	1638	1638	100	1	1638	158925	160562	0	3247
S95EC	221	231860	M.EcoMIII	1638	15	100	739	753	89132	89146	6.7	30.2
S95EC	221	231860	M.EcoMIII	1638	15	100	992	1006	163638	163652	6.7	30.2
S95EC	12	191455	M.EcoMIV	765	765	100	1	765	65343	66107	0	1516
S95EC	13	95893	M.EcoMV	1050	1050	100	1	1050	91871	92920	0	2040

S95EC	50	38976	M.EcoNwEVDcm	1419	1419	100	1	1419	8850	10268	0	2813
S95EC	60	41718	M.EcoMVI	891	891	100	1	891	11922	11032	0	1725
S95EC	40	190882	M.EcoNwEDam	837	837	100	1	837	39459	38623	0	1659
S95EC	132	155924	M.EcoMVII	3606	25	96	596	620	10593	10617	0.004	42.1
S95EC	132	155924	M.EcoMVII	3606	16	100	201	216	3777	3792	3.7	32.2
S95EC	93	5728	M.EcoMVIII	684	684	100	1	684	1828	2511	0	1356
S95EC	66	97402	Eco29kI	645	16	100	375	390	11474	11489	0.65	32.2
S95EC	66	97402	Eco29kI	645	15	100	494	508	87666	87652	2.6	30.2
S95EC	131	77648	EcoDEC4CORF2749P	1041	16	100	643	658	46683	46668	1.1	32.2
S95EC	127	523787	Eco248534P	1053	16	100	941	956	202170	202155	1.1	32.2
S95EC	127	523787	Eco248534P	1053	15	100	972	986	76385	76399	4.3	30.2
S95EC	127	523787	Eco248534P	1053	15	100	66	80	206060	206046	4.3	30.2
S95EC	205	109384	EcoAPECORF2077P	1590	18	100	608	625	58235	58218	0.1	36.2
S95EC	205	109384	EcoAPECORF2077P	1590	17	100	1034	1050	64742	64726	0.41	34.2
S95EC	205	109384	EcoAPECORF2077P	1590	15	100	1543	1557	78594	78580	6.5	30.2
S95EC	205	109384	EcoAPECORF2077P	1590	15	100	668	682	84577	84563	6.5	30.2
S95EC	149	658	EcoDEC13EORF3046P	1191	16	100	920	935	31	16	1.2	32.2
S95EC	93	5728	Eco7A8ORF29P	684	606	98.51	79	684	1906	2511	0	1130
S95EC	127	523787	EcoDEC2CORF2043P	2019	21	95.24	1522	1542	17565	17585	0.53	34.2
S95EC	127	523787	EcoDEC2CORF2043P	2019	15	100	914	928	325548	325562	8.2	30.2
S95EC	127	523787	EcoDEC2CORF2043P	2019	15	100	489	503	365472	365486	8.2	30.2
S95EC	127	523787	EcoDEC2CORF2043P	2019	15	100	186	200	521260	521274	8.2	30.2
S95EC	13	95893	Eco1886ORF14455P	1053	594	82.32	7	600	91877	92470	6e-95	345
S95EC	13	95893	Eco1886ORF14455P	1053	104	81.73	817	920	92687	92790	7e-08	56
S95EC	222	116931	Eco1886ORF14565P	900	15	100	67	81	80182	80168	3.6	30.2
S95EC	222	116931	Eco1886ORF14565P	900	15	100	74	88	107782	107796	3.6	30.2
S95EC	93	5728	EcoR7ACORFAP	684	684	97.37	1	684	1828	2511	0	1213
S95EC	93	5728	EcoR100ORF1P	648	606	98.51	43	648	1906	2511	0	1130
S95EC	93	5728	Eco605ORFMP	684	684	93.86	1	684	1828	2511	0	1023
S95EC	61	7572	Eco84137ORF201P	1635	27	92.59	811	837	5526	5500	0.027	38.2
S95EC	61	7572	Eco84137ORF201P	1635	20	95	550	569	5766	5747	1.7	32.2
S95EC	61	7572	Eco1520ORF67P	1563	1563	99.94	1	1563	6300	4738	0	3049
S95EC	7	12868	Eco15ORF4165P	1197	33	87.88	237	269	7254	7222	0.31	34.2
HVR2496	70	10931	M1.EcoMI	1623	1623	100	1	1623	9545	7923	0	3009
HVR2496	70	10931	M1.EcoMI	1191	1191	100	1	1191	7930	6740	0	2361
HVR2496	16	108341	M.EcoMII	1620	1581	100	40	1620	70848	72428	0	3092

HVR2496	219	320180	M.EcoMIII	1638	1638	100	1	1638	72879	71242	0	3247
HVR2496	219	320180	M.EcoMIII	1638	16	100	1336	1351	259843	259858	1.6	32.2
HVR2496	219	320180	M.EcoMIII	1638	15	100	992	1006	68166	68152	6.5	30.2
HVR2496	219	320180	M.EcoMIII	1638	15	100	739	753	142672	142658	6.5	30.2
HVR2496	219	320180	M.EcoMIII	1638	15	100	1509	1523	227020	227034	6.5	30.2
HVR2496	86	72689	M.EcoMIV	765	765	100	1	765	7421	6657	0	1516
HVR2496	66	9488	M.EcoMV	1050	1050	100	1	1050	4099	3050	0	2040
HVR2496	87	39004	M.EcoNwEVDcm	1419	1419	100	1	1419	8852	10270	0	2813
HVR2496	11	127337	M.EcoMVI	891	891	100	1	891	115490	116380	0	1725
HVR2496	1	190884	M.EcoNwEDam	837	837	100	1	837	39461	38625	0	1659
HVR2496	273	223029	M.EcoMVII	3606	25	96	596	620	104704	104728	0.004	42.1
HVR2496	273	223029	M.EcoMVII	3606	16	100	201	216	97997	98012	3.6	32.2
HVR2496	76	14492	M.EcoMVIII	684	684	100	1	684	10592	11275	0	1356
HVR2496	67	23839	Eco29kI	645	16	100	375	390	17517	17502	0.63	32.2
HVR2496	273	223029	EcoDEC4CORF2749P	1041	16	100	654	669	63406	63391	1	32.2
HVR2496	109	11288	Eco248534P	1053	16	100	262	277	5809	5824	1	32.2
HVR2496	41	37004	EcoAPECORF2077P	1590	18	100	1260	1277	947	930	0.1	36.2
HVR2496	41	37004	EcoAPECORF2077P	1590	17	100	99	115	21937	21953	0.4	34.2
HVR2496	164	656	EcoDEC13EORF3046P	1191	16	100	920	935	702	717	1.2	32.2
HVR2496	76	14492	Eco7A8ORF29P	684	606	98.51	79	684	10670	11275	0	1130
HVR2496	49	30245	EcoDEC2CORF2043P	2019	21	95.24	1522	1542	12735	12715	0.51	34.2
HVR2496	66	9488	Eco1886ORF14455P	1053	594	82.32	7	600	4093	3500	5e-95	345
HVR2496	66	9488	Eco1886ORF14455P	1053	104	81.73	817	920	3283	3180	7e-08	56
HVR2496	273	223029	Eco1886ORF14565P	900	15	100	565	579	24861	24847	3.5	30.2
HVR2496	76	14492	EcoR7ACORFAP	684	684	97.37	1	684	10592	11275	0	1213
HVR2496	76	14492	EcoR100ORF1P	648	606	98.51	43	648	10670	11275	0	1130
HVR2496	76	14492	Eco605ORFMP	684	684	93.86	1	684	10592	11275	0	1023
HVR2496	45	10988	Eco84137ORF201P	1635	27	92.59	811	837	5824	5850	0.026	38.2
HVR2496	45	10988	Eco84137ORF201P	1635	20	95	550	569	5584	5603	1.6	32.2
HVR2496	45	10988	Eco1520ORF67P	1563	1563	99.94	1	1563	5050	6612	0	3049
HVR2496	70	10931	Eco15ORF4165P	1197	33	87.88	237	269	7256	7224	0.3	34.2
HVM52	75	129723	M1.EcoMI	1623	18	100	364	381	77411	77428	0.11	36.2
HVM52	269	11441	M1.EcoMI	1191	16	100	1153	1168	10413	10398	1.3	32.2
HVM52	14	80118	M.EcoMII	1620	19	100	1333	1351	73708	73690	0.028	38.2
HVM52	14	80118	M.EcoMII	1620	17	100	273	289	32010	31994	0.43	34.2
HVM52	22	14762	M.EcoMIII	1638	1638	100	1	1638	2058	3695	0	3247

HVM52	88	101224	M.EcoMIV	765	17	100	332	348	31021	31037	0.2	34.2
HVM52	88	101224	M.EcoMIV	765	15	100	345	359	42183	42169	3.2	30.2
HVM52	594	291	M.EcoMV	1050	322	86.34	86	407	324	3	3e-78	289
HVM52	126	9651	M.EcoNwEVDcm	1419	1419	100	1	1419	7404	8822	0	2813
HVM52	13	88066	M.EcoMVI	891	891	100	1	891	76219	77109	0	1725
HVM52	17	161692	M.EcoNwEDam	837	837	100	1	837	132031	132867	0	1659
HVM52	78	11155	M.EcoMVII	3606	25	96	596	620	10687	10711	0.004	42.1
HVM52	78	11155	M.EcoMVII	3606	16	100	201	216	3871	3886	3.8	32.2
HVM52	177	3814	M.EcoMVIII	684	680	94.26	5	684	3896	3217	0	1039
HVM52	235	7134	Eco29kI	645	16	100	375	390	6316	6331	0.67	32.2
HVM52	29	9398	EcoDEC4CORF2749P	1041	16	100	643	658	4796	4811	1.1	32.2
HVM52	143	18352	Eco248534P	1053	16	100	941	956	10896	10881	1.1	32.2
HVM52	143	18352	Eco248534P	1053	15	100	66	80	14786	14772	4.4	30.2
HVM52	540	25330	EcoAPECORF2077P	1590	19	100	312	330	22045	22063	0.027	38.2
HVM52	540	25330	EcoAPECORF2077P	1590	15	100	724	738	18190	18176	6.6	30.2
HVM52	451	747	EcoDEC13EORF3046P	1191	16	100	920	935	30	15	1.3	32.2
HVM52	177	3814	Eco7A8ORF29P	684	621	95.65	64	684	3837	3217	0	1017
HVM52	167	27764	EcoDEC2CORF2043P	2019	21	95.24	1522	1542	17552	17572	0.54	34.2
HVM52	763	15634	Eco1886ORF14455P	1053	726	99.45	328	1053	1	726	0	1366
HVM52	254	6154	Eco1886ORF14565P	900	15	100	831	845	5954	5968	3.7	30.2
HVM52	177	3814	EcoR7ACORFAP	684	680	94.85	5	684	3896	3217	0	1070
HVM52	177	3814	EcoR100ORF1P	648	621	95.65	28	648	3837	3217	0	1017
HVM52	177	3814	Eco605ORFMP	684	680	99.41	5	684	3896	3217	0	1316
HVM52	209	10271	Eco84137ORF201P	1635	27	92.59	811	837	5674	5648	0.028	38.2
HVM52	209	10271	Eco84137ORF201P	1635	20	95	550	569	5914	5895	1.7	32.2
HVM52	209	10271	Eco1520ORF67P	1563	1563	99.94	1	1563	6448	4886	0	3049
HVM52	480	4279	Eco15ORF4165P	1197	16	100	280	295	4227	4242	1.3	32.2
HVM5	102	11693	M1.EcoMI	1623	1623	100	1	1623	2225	3847	0	3009
HVM5	102	11693	M1.EcoMI	1191	1191	100	1	1191	3840	5030	0	2361
HVM5	126	74040	M.EcoMII	1620	1581	100	40	1620	45647	44067	0	3092
HVM5	15	240063	M.EcoMIII	1638	1638	100	1	1638	235084	236721	0	3247
HVM5	15	240063	M.EcoMIII	1638	16	100	1336	1351	32034	32019	1.6	32.2
HVM5	15	240063	M.EcoMIII	1638	15	100	1509	1523	64857	64843	6.4	30.2
HVM5	15	240063	M.EcoMIII	1638	15	100	739	753	165291	165305	6.4	30.2
HVM5	15	240063	M.EcoMIII	1638	15	100	992	1006	239797	239811	6.4	30.2
HVM5	9	538	M.EcoMIV	765	584	99.49	67	650	1	584	0	1134

HVM5	88	26175	M.EcoMV	1050	1050	100	1	1050	22300	23349	0	2040
HVM5	18	137755	M.EcoNwEVDcm	1419	1419	100	1	1419	40359	38941	0	2813
HVM5	64	158981	M.EcoMVI	891	891	100	1	891	147134	148024	0	1725
HVM5	65	190854	M.EcoNwEDam	837	837	100	1	837	39431	38595	0	1659
HVM5	94	299882	M.EcoMVII	3606	25	96	596	620	185120	185144	0.004	42.1
HVM5	94	299882	M.EcoMVII	3606	16	100	201	216	178304	178319	3.6	32.2
HVM5	233	72658	M.EcoMVIII	684	16	100	410	425	59094	59109	0.67	32.2
HVM5	31	130598	Eco29kI	645	16	100	238	253	46007	46022	0.63	32.2
HVM5	31	130598	Eco29kI	645	18	94.44	210	227	17720	17703	9.8	28.2
HVM5	94	299882	EcoDEC4CORF2749P	1041	16	100	654	669	143713	143698	1	32.2
HVM5	378	20082	Eco248534P	1053	16	100	262	277	741	726	1	32.2
HVM5	194	173619	EcoAPECORF2077P	1590	18	100	1494	1511	149394	149411	0.1	36.2
HVM5	194	173619	EcoAPECORF2077P	1590	16	100	827	842	63440	63455	1.6	32.2
HVM5	194	173619	EcoAPECORF2077P	1590	15	100	414	428	5297	5283	6.2	30.2
HVM5	164	145489	EcoDEC13EORF3046P	1191	16	100	920	935	67955	67940	1.2	32.2
HVM5	162	29612	Eco7A8ORF29P	684	20	95	213	232	26972	26991	0.67	32.2
HVM5	167	21971	EcoDEC2CORF2043P	2019	21	95.24	1522	1542	44	24	0.51	34.2
HVM5	88	26175	Eco1886ORF14455P	1053	594	82.32	7	600	22306	22899	5e-95	345
HVM5	88	26175	Eco1886ORF14455P	1053	104	81.73	817	920	23116	23219	7e-08	56
HVM5	233	72658	Eco1886ORF14565P	900	15	100	831	845	7330	7316	3.5	30.2
HVM5	26	109033	EcoR7ACORFAP	684	21	95.24	181	201	23804	23824	0.17	34.2
HVM5	162	29612	EcoR100ORF1P	648	20	95	177	196	26972	26991	0.63	32.2
HVM5	162	29612	EcoR100ORF1P	648	14	100	345	358	3463	3450	9.9	28.2
HVM5	167	21971	Eco605ORFMP	684	16	100	112	127	12442	12457	0.67	32.2
HVM5	158	7532	Eco84137ORF201P	1635	27	92.59	811	837	2093	2119	0.026	38.2
HVM5	158	7532	Eco84137ORF201P	1635	20	95	550	569	1853	1872	1.6	32.2
HVM5	158	7532	Eco1520ORF67P	1563	1563	99.94	1	1563	1319	2881	0	3049
HVM5	102	11693	Eco15ORF4165P	1197	33	87.88	237	269	4514	4546	0.3	34.2
HVM3189	119	11725	M1.EcoMI	1623	1623	100	1	1623	9547	7925	0	3009
HVM3189	119	11725	M1.EcoMI	1191	1191	100	1	1191	7932	6742	0	2361
HVM3189	15	116441	M.EcoMII	1620	1581	100	40	1620	45679	44099	0	3092
HVM3189	1	139152	M.EcoMIII	1638	1638	100	1	1638	66217	67854	0	3247
HVM3189	1	139152	M.EcoMIII	1638	15	100	992	1006	70930	70944	6.5	30.2
HVM3189	16	34680	M.EcoMIV	765	765	100	1	765	31098	30334	0	1516
HVM3189	109	3687	M.EcoMV	1050	714	100	337	1050	1	714	0	1374
HVM3189	33	19727	M.EcoNwEVDcm	1419	1419	100	1	1419	8854	10272	0	2813

HVM3189	77	91683	M.EcoMVI	891	891	100	1	891	79836	80726	0	1725
HVM3189	50	171399	M.EcoNwEDam	837	837	100	1	837	39463	38627	0	1659
HVM3189	36	39013	M.EcoMVII	3606	25	96	596	620	8672	8648	0.004	42.1
HVM3189	36	39013	M.EcoMVII	3606	16	100	201	216	15488	15473	3.6	32.2
HVM3189	117	10598	M.EcoMVIII	684	684	100	1	684	8461	9144	0	1356
HVM3189	29	36163	Eco29kI	645	16	100	375	390	11469	11484	0.64	32.2
HVM3189	18	109708	EcoDEC4CORF2749P	1041	16	100	654	669	11060	11075	1	32.2
HVM3189	101	11288	Eco248534P	1053	16	100	262	277	5811	5826	1.1	32.2
HVM3189	299	109367	EcoAPECORF2077P	1590	18	100	608	625	51228	51245	0.1	36.2
HVM3189	299	109367	EcoAPECORF2077P	1590	17	100	1034	1050	44721	44737	0.4	34.2
HVM3189	299	109367	EcoAPECORF2077P	1590	15	100	668	682	24886	24900	6.3	30.2
HVM3189	299	109367	EcoAPECORF2077P	1590	15	100	1543	1557	30869	30883	6.3	30.2
HVM3189	226	654	EcoDEC13EORF3046P	1191	16	100	920	935	702	717	1.2	32.2
HVM3189	117	10598	Eco7A8ORF29P	684	606	98.51	79	684	8539	9144	0	1130
HVM3189	86	23888	EcoDEC2CORF2043P	2019	21	95.24	1522	1542	17374	17354	0.52	34.2
HVM3189	288	523	Eco1886ORF14455P	1053	389	84.58	7	395	205	593	5e-80	295
HVM3189	354	109125	Eco1886ORF14565P	900	15	100	289	303	39950	39964	3.6	30.2
HVM3189	354	109125	Eco1886ORF14565P	900	15	100	273	287	59136	59122	3.6	30.2
HVM3189	117	10598	EcoR7ACORFAP	684	684	97.37	1	684	8461	9144	0	1213
HVM3189	117	10598	EcoR100ORF1P	648	606	98.51	43	648	8539	9144	0	1130
HVM3189	117	10598	Eco605ORFMP	684	684	93.86	1	684	8461	9144	0	1023
HVM3189	35	158489	Eco84137ORF201P	1635	743	77.12	520	1262	54211	53469	1e-28	125
HVM3189	35	158489	Eco84137ORF201P	1635	152	80.92	1	152	54730	54579	2e-12	71.9
HVM3189	35	158489	Eco84137ORF201P	1635	15	100	495	509	12700	12714	6.5	30.2
HVM3189	35	158489	Eco84137ORF201P	1635	15	100	20	34	155755	155769	6.5	30.2
HVM3189	90	7576	Eco1520ORF67P	1563	1563	99.94	1	1563	1351	2913	0	3049
HVM3189	119	11725	Eco15ORF4165P	1197	33	87.88	237	269	7258	7226	0.3	34.2
P50EC	119	11722	M1.EcoMI	1623	1623	100	1	1623	2254	3876	0	3009
P50EC	119	11722	M1.EcoMI	1191	1191	100	1	1191	3869	5059	0	2361
P50EC	27	44849	M.EcoMII	1620	829	100	792	1620	1	829	0	1643
P50EC	72	51177	M.EcoMIII	1638	1638	100	1	1638	12889	14526	0	3247
P50EC	72	51177	M.EcoMIII	1638	15	100	992	1006	17602	17616	6.7	30.2
P50EC	63	10645	M.EcoMIV	765	765	100	1	765	6083	6847	0	1516
P50EC	277	3636	M.EcoMV	1050	1038	100	1	1038	2675	3712	0	2016
P50EC	81	24293	M.EcoNwEVDcm	1419	1419	100	1	1419	15518	14100	0	2813
P50EC	65	19344	M.EcoMVI	891	891	100	1	891	11924	11034	0	1725

P50EC	11	171394	M.EcoNwEDam	837	837	100	1	837	39461	38625	0	1659
P50EC	252	11890	M.EcoMVII	3606	25	96	596	620	1405	1381	0.004	42.1
P50EC	252	11890	M.EcoMVII	3606	16	100	201	216	8221	8206	3.7	32.2
P50EC	229	5730	M.EcoMVIII	684	684	100	1	684	1830	2513	0	1356
P50EC	19	47991	Eco29kI	645	16	100	375	390	37180	37165	0.65	32.2
P50EC	284	2936	EcoDEC4CORF2749P	1041	16	100	654	669	241	226	1.1	32.2
P50EC	184	18374	Eco248534P	1053	16	100	941	956	8891	8876	1.1	32.2
P50EC	184	18374	Eco248534P	1053	15	100	66	80	12781	12767	4.3	30.2
P50EC	116	16864	EcoAPECORF2077P	1590	18	100	1260	1277	3737	3754	0.1	36.2
P50EC	116	16864	EcoAPECORF2077P	1590	17	100	1	17	8099	8083	0.41	34.2
P50EC	150	1629	EcoDEC13EORF3046P	1191	16	100	920	935	1660	1645	1.2	32.2
P50EC	229	5730	Eco7A8ORF29P	684	606	98.51	79	684	1908	2513	0	1130
P50EC	134	3630	EcoDEC2CORF2043P	2019	21	95.24	1522	1542	373	353	0.53	34.2
P50EC	277	3636	Eco1886ORF14455P	1053	594	82.32	7	600	2681	3274	6e-95	345
P50EC	277	3636	Eco1886ORF14455P	1053	104	81.73	817	920	3491	3594	7e-08	56
P50EC	396	20969	Eco1886ORF14565P	900	15	100	565	579	14307	14293	3.6	30.2
P50EC	229	5730	EcoR7ACORFAP	684	684	97.37	1	684	1830	2513	0	1213
P50EC	229	5730	EcoR100ORF1P	648	606	98.51	43	648	1908	2513	0	1130
P50EC	229	5730	Eco605ORFMP	684	684	93.86	1	684	1830	2513	0	1023
P50EC	208	2991	Eco84137ORF201P	1635	27	92.59	811	837	945	919	0.027	38.2
P50EC	208	2991	Eco84137ORF201P	1635	20	95	550	569	1185	1166	1.7	32.2
P50EC	208	2991	Eco1520ORF67P	1563	1563	99.94	1	1563	1719	157	0	3049
P50EC	119	11722	Eco15ORF4165P	1197	33	87.88	237	269	4543	4575	0.31	34.2
HVR83	60	11705	M1.EcoMI	1623	1623	100	1	1623	2237	3859	0	3009
HVR83	60	11705	M1.EcoMI	1191	1191	100	1	1191	3852	5042	0	2361
HVR83	65	74078	M.EcoMII	1620	1581	100	40	1620	28454	30034	0	3092
HVR83	14	114122	M.EcoMIII	1638	1638	100	1	1638	72863	71226	0	3247
HVR83	14	114122	M.EcoMIII	1638	15	100	992	1006	68150	68136	6.8	30.2
HVR83	58	274909	M.EcoMIV	765	765	100	1	765	65329	66093	0	1516
HVR83	64	95878	M.EcoMV	1050	1050	100	1	1050	4083	3034	0	2040
HVR83	149	38974	M.EcoNwEVDcm	1419	1419	100	1	1419	30199	28781	0	2813
HVR83	179	158995	M.EcoMVI	891	891	100	1	891	11908	11018	0	1725
HVR83	51	190868	M.EcoNwEDam	837	837	100	1	837	39445	38609	0	1659
HVR83	384	101530	M.EcoMVII	3606	25	96	596	620	89501	89525	0.004	42.1
HVR83	384	101530	M.EcoMVII	3606	16	100	201	216	82685	82700	3.8	32.2
HVR83	112	13433	M.EcoMVIII	684	684	100	1	684	2804	2121	0	1356

HVR83	102	97413	Eco29kI	645	16	100	375	390	11485	11500	0.67	32.2
HVR83	102	97413	Eco29kI	645	15	100	494	508	87677	87663	2.7	30.2
HVR83	384	101530	EcoDEC4CORF2749P	1041	16	100	654	669	48094	48079	1.1	32.2
HVR83	81	6455	Eco248534P	1053	16	100	262	277	5539	5524	1.1	32.2
HVR83	179	158995	EcoAPECORF2077P	1590	18	100	1511	1528	98194	98211	0.11	36.2
HVR83	179	158995	EcoAPECORF2077P	1590	17	100	1550	1566	45484	45500	0.43	34.2
HVR83	179	158995	EcoAPECORF2077P	1590	15	100	50	64	9327	9341	6.6	30.2
HVR83	179	158995	EcoAPECORF2077P	1590	19	94.74	1493	1511	71616	71634	6.6	30.2
HVR83	137	672	EcoDEC13EORF3046P	1191	16	100	920	935	31	16	1.3	32.2
HVR83	124	12210	Eco7A8ORF29P	684	621	98.55	64	684	10289	10909	0	1160
HVR83	48	147514	EcoDEC2CORF2043P	2019	21	95.24	1522	1542	112647	112667	0.54	34.2
HVR83	48	147514	EcoDEC2CORF2043P	2019	16	100	703	718	37383	37368	2.1	32.2
HVR83	48	147514	EcoDEC2CORF2043P	2019	15	100	1262	1276	62702	62688	8.5	30.2
HVR83	64	95878	Eco1886ORF14455P	1053	594	82.32	7	600	4077	3484	6e-95	345
HVR83	64	95878	Eco1886ORF14455P	1053	104	81.73	817	920	3267	3164	8e-08	56
HVR83	384	101530	Eco1886ORF14565P	900	15	100	565	579	9549	9535	3.7	30.2
HVR83	124	12210	EcoR7ACORFAP	684	684	100	1	684	10226	10909	0	1356
HVR83	124	12210	EcoR100ORF1P	648	621	98.55	28	648	10289	10909	0	1160
HVR83	124	12210	Eco605ORFMP	684	684	94.44	1	684	10226	10909	0	1055
HVR83	73	3856	Eco84137ORF201P	1635	27	92.59	811	837	1810	1784	0.028	38.2
HVR83	73	3856	Eco84137ORF201P	1635	20	95	550	569	2050	2031	1.7	32.2
HVR83	73	3856	Eco1520ORF67P	1563	1563	99.94	1	1563	2584	1022	0	3049
HVR83	60	11705	Eco15ORF4165P	1197	33	87.88	237	269	4526	4558	0.32	34.2
S1EC	57	32283	M1.EcoMI	1623	1623	100	1	1623	28958	27336	0	3009
S1EC	57	32283	M1.EcoMI	1191	1191	100	1	1191	27343	26153	0	2361
S1EC	41	81248	M.EcoMII	1620	1581	100	40	1620	35647	37227	0	3092
S1EC	5	75352	M.EcoMIII	1638	1638	100	1	1638	71965	73602	0	3247
S1EC	5	75352	M.EcoMIII	1638	15	100	739	753	2172	2186	6.7	30.2
S1EC	170	20056	M.EcoMIV	765	765	100	1	765	8017	8781	0	1516
S1EC	330	2091	M.EcoMV	1050	1050	100	1	1050	1965	916	0	2040
S1EC	130	10153	M.EcoNwEVDcm	1419	1378	100	1	1378	8856	10233	0	2732
S1EC	69	89863	M.EcoMVI	891	891	100	1	891	11928	11038	0	1725
S1EC	54	27850	M.EcoNwEDam	837	837	100	1	837	14968	15804	0	1659
S1EC	50	97163	M.EcoMVII	3606	25	96	596	620	71549	71525	0.004	42.1
S1EC	50	97163	M.EcoMVII	3606	16	100	201	216	78253	78238	3.7	32.2
S1EC	116	11993	M.EcoMVIII	684	684	100	1	684	2206	1523	0	1356

S1EC	56	64635	Eco29kI	645	16	100	238	253	18675	18660	0.65	32.2
S1EC	153	8406	EcoDEC4CORF2749P	1041	16	100	643	658	7722	7737	1.1	32.2
S1EC	115	26699	Eco248534P	1053	16	100	941	956	12812	12827	1.1	32.2
S1EC	115	26699	Eco248534P	1053	15	100	66	80	8922	8936	4.3	30.2
S1EC	34	35111	EcoAPECORF2077P	1590	19	100	312	330	3818	3800	0.027	38.2
S1EC	34	35111	EcoAPECORF2077P	1590	15	100	724	738	7518	7532	6.5	30.2
S1EC	34	35111	EcoAPECORF2077P	1590	19	94.74	1523	1541	33601	33619	6.5	30.2
S1EC	235	22519	EcoDEC13EORF3046P	1191	16	100	8	23	20351	20366	1.2	32.2
S1EC	116	11993	Eco7A8ORF29P	684	606	98.51	79	684	2128	1523	0	1130
S1EC	76	40940	EcoDEC2CORF2043P	2019	21	95.24	1522	1542	17577	17597	0.53	34.2
S1EC	34	35111	Eco1886ORF14455P	1053	1053	100	1	1053	10224	11276	0	2046
S1EC	13	11458	Eco1886ORF14565P	900	900	100	1	900	5707	4808	0	1742
S1EC	116	11993	EcoR7ACORFAP	684	684	97.37	1	684	2206	1523	0	1213
S1EC	116	11993	EcoR100ORF1P	648	606	98.51	43	648	2128	1523	0	1130
S1EC	116	11993	Eco605ORFMP	684	684	93.86	1	684	2206	1523	0	1023
S1EC	99	16926	Eco84137ORF201P	1635	22	95.45	558	579	6625	6604	0.11	36.2
S1EC	243	45792	Eco1520ORF67P	1563	18	100	1532	1549	10487	10504	0.1	36.2
S1EC	57	32283	Eco15ORF4165P	1197	33	87.88	237	269	26669	26637	0.31	34.2
S11EC	306	8960	M1.EcoMI	1623	1623	100	1	1623	3408	5030	0	3009
S11EC	306	8960	M1.EcoMI	1191	1191	100	1	1191	5023	6213	0	2361
S11EC	57	36913	M.EcoMII	1620	996	100	625	1620	1	996	0	1974
S11EC	31	59999	M.EcoMIII	1638	1638	100	1	1638	27267	25630	0	3247
S11EC	31	59999	M.EcoMIII	1638	15	100	992	1006	22554	22540	6.7	30.2
S11EC	64	64402	M.EcoMIV	765	765	100	1	765	47888	48652	0	1516
S11EC	189	49048	M.EcoMV	1050	1050	100	1	1050	45026	46075	0	2040
S11EC	275	38972	M.EcoNwEVDcm	1419	1419	100	1	1419	30197	28779	0	2813
S11EC	89	27897	M.EcoMVI	891	891	100	1	891	16050	16940	0	1725
S11EC	185	11281	M.EcoNwEDam	837	837	100	1	837	10565	9729	0	1659
S11EC	41	52493	M.EcoMVII	3606	25	96	596	620	7738	7762	0.004	42.1
S11EC	41	52493	M.EcoMVII	3606	16	100	201	216	922	937	3.7	32.2
S11EC	438	1978	M.EcoMVIII	684	604	100	81	684	7	610	0	1197
S11EC	270	31824	Eco29kI	645	16	100	375	390	20437	20422	0.66	32.2
S11EC	146	3632	EcoDEC4CORF2749P	1041	16	100	654	669	261	246	1.1	32.2
S11EC	154	7141	Eco248534P	1053	20	95	908	927	1823	1804	1.1	32.2
S11EC	161	35288	EcoAPECORF2077P	1590	19	100	312	330	6233	6251	0.027	38.2
S11EC	161	35288	EcoAPECORF2077P	1590	15	100	724	738	2533	2519	6.5	30.2

S11EC	161	35288	EcoAPECORF2077P	1590	15	100	1314	1328	18765	18751	6.5	30.2
S11EC	400	748	EcoDEC13EORF3046P	1191	16	100	920	935	800	815	1.2	32.2
S11EC	438	1978	Eco7A8ORF29P	684	604	98.51	81	684	7	610	0	1126
S11EC	148	6475	EcoDEC2CORF2043P	2019	21	95.24	1522	1542	98	78	0.53	34.2
S11EC	194	8567	Eco1886ORF14455P	1053	1053	100	1	1053	246	1298	0	2046
S11EC	161	35288	Eco1886ORF14565P	900	900	100	1	900	15804	16703	0	1742
S11EC	438	1978	EcoR7ACORFAP	684	604	98.34	81	684	7	610	0	1118
S11EC	438	1978	EcoR100ORF1P	648	604	98.51	45	648	7	610	0	1126
S11EC	223	2488	Eco605ORFMP	684	684	100	1	684	121	804	0	1356
S11EC	187	7698	Eco84137ORF201P	1635	22	95.45	558	579	5011	5032	0.11	36.2
S11EC	161	35288	Eco1520ORF67P	1563	18	100	1532	1549	31962	31979	0.1	36.2
S11EC	306	8960	Eco15ORF4165P	1197	33	87.88	237	269	5697	5729	0.31	34.2
S93EC	89	30919	M1.EcoMI	1623	1623	100	1	1623	29723	28101	0	3009
S93EC	89	30919	M1.EcoMI	1191	1191	100	1	1191	28108	26918	0	2361
S93EC	75	43528	M.EcoMII	1620	1024	100	597	1620	1	1024	0	2030
S93EC	94	63966	M.EcoMIII	1638	1638	100	1	1638	18152	16515	0	3247
S93EC	94	63966	M.EcoMIII	1638	15	100	992	1006	13439	13425	6.6	30.2
S93EC	125	33591	M.EcoMIV	765	765	100	1	765	24558	23794	0	1516
S93EC	202	6204	M.EcoMV	1050	1050	100	1	1050	4101	3052	0	2040
S93EC	110	7103	M.EcoNwEVDcm	1419	1419	100	1	1419	2319	901	0	2813
S93EC	57	38437	M.EcoMVI	891	891	100	1	891	11926	11036	0	1725
S93EC	105	72230	M.EcoNwEDam	837	837	100	1	837	12921	12085	0	1659
S93EC	126	64224	M.EcoMVII	3606	25	96	596	620	43812	43836	0.004	42.1
S93EC	126	64224	M.EcoMVII	3606	16	100	201	216	36996	37011	3.7	32.2
S93EC	190	3897	M.EcoMVIII	684	684	100	1	684	2145	1462	0	1356
S93EC	47	24119	Eco29kI	645	16	100	375	390	11474	11489	0.65	32.2
S93EC	140	43035	EcoDEC4CORF2749P	1041	16	100	643	658	19830	19845	1.1	32.2
S93EC	108	25554	Eco248534P	1053	16	100	941	956	22163	22148	1.1	32.2
S93EC	113	22896	EcoAPECORF2077P	1590	19	100	312	330	14504	14486	0.026	38.2
S93EC	113	22896	EcoAPECORF2077P	1590	15	100	1314	1328	1972	1986	6.4	30.2
S93EC	113	22896	EcoAPECORF2077P	1590	15	100	724	738	18204	18218	6.4	30.2
S93EC	205	752	EcoDEC13EORF3046P	1191	16	100	920	935	800	815	1.2	32.2
S93EC	190	3897	Eco7A8ORF29P	684	606	98.51	79	684	2067	1462	0	1130
S93EC	106	46431	EcoDEC2CORF2043P	2019	21	95.24	1522	1542	30215	30195	0.52	34.2
S93EC	113	22896	Eco1886ORF14455P	1053	1053	100	1	1053	20910	21962	0	2046
S93EC	113	22896	Eco1886ORF14565P	900	900	100	1	900	4933	4034	0	1742

S93EC	190	3897	EcoR7ACORFAP	684	684	97.37	1	684	2145	1462	0	1213
S93EC	190	3897	EcoR100ORF1P	648	606	98.51	43	648	2067	1462	0	1130
S93EC	190	3897	Eco605ORFMP	684	684	93.86	1	684	2145	1462	0	1023
S93EC	163	13538	Eco84137ORF201P	1635	22	95.45	558	579	3218	3197	0.11	36.2
S93EC	245	53059	Eco1520ORF67P	1563	18	100	1532	1549	2389	2406	0.1	36.2
S93EC	89	30919	Eco15ORF4165P	1197	33	87.88	237	269	27434	27402	0.31	34.2
S113EC	31	12213	M1.EcoMI	1623	1623	100	1	1623	8888	7266	0	3009
S113EC	31	12213	M1.EcoMI	1191	1191	100	1	1191	7273	6083	0	2361
S113EC	6	116445	M.EcoMII	1620	1581	100	40	1620	45676	44096	0	3092
S113EC	30	264341	M.EcoMIII	1638	1638	100	1	1638	72877	71240	0	3247
S113EC	30	264341	M.EcoMIII	1638	15	100	992	1006	68164	68150	6.8	30.2
S113EC	30	264341	M.EcoMIII	1638	15	100	739	753	142670	142656	6.8	30.2
S113EC	30	264341	M.EcoMIII	1638	15	100	1509	1523	243104	243118	6.8	30.2
S113EC	82	37101	M.EcoMIV	765	765	100	1	765	6106	6870	0	1516
S113EC	123	7669	M.EcoMV	1050	1050	100	1	1050	6587	7636	0	2040
S113EC	125	38974	M.EcoNwEVDcm	1419	1419	100	1	1419	8850	10268	0	2813
S113EC	24	91674	M.EcoMVI	891	891	100	1	891	11922	11032	0	1725
S113EC	33	171398	M.EcoNwEDam	837	837	100	1	837	132014	132850	0	1659
S113EC	259	186585	M.EcoMVII	3606	25	96	596	620	185595	185619	0.004	42.1
S113EC	259	186585	M.EcoMVII	3606	16	100	201	216	178779	178794	3.8	32.2
S113EC	88	5728	M.EcoMVIII	684	684	99.85	1	684	1828	2511	0	1348
S113EC	41	63483	Eco29kI	645	16	100	238	253	46035	46050	0.66	32.2
S113EC	259	186585	EcoDEC4CORF2749P	1041	16	100	654	669	144188	144173	1.1	32.2
S113EC	106	23263	Eco248534P	1053	16	100	971	986	18799	18814	1.1	32.2
S113EC	109	173761	EcoAPECORF2077P	1590	18	100	1494	1511	24300	24283	0.11	36.2
S113EC	109	173761	EcoAPECORF2077P	1590	16	100	827	842	110254	110239	1.7	32.2
S113EC	109	173761	EcoAPECORF2077P	1590	15	100	414	428	168510	168524	6.6	30.2
S113EC	135	756	EcoDEC13EORF3046P	1191	16	100	920	935	800	815	1.2	32.2
S113EC	88	5728	Eco7A8ORF29P	684	606	98.35	79	684	1906	2511	0	1122
S113EC	79	16750	EcoDEC2CORF2043P	2019	2019	100	1	2019	5427	7445	0	4002
S113EC	79	16750	EcoDEC2CORF2043P	2019	24	91.67	139	162	5588	5565	2.1	32.2
S113EC	123	7669	Eco1886ORF14455P	1053	594	82.32	7	600	6593	7186	6e-95	345
S113EC	123	7669	Eco1886ORF14455P	1053	104	81.73	817	920	7403	7506	8e-08	56
S113EC	106	23263	Eco1886ORF14565P	900	16	100	519	534	17708	17723	0.93	32.2
S113EC	88	5728	EcoR7ACORFAP	684	684	97.22	1	684	1828	2511	0	1205
S113EC	88	5728	EcoR100ORF1P	648	606	98.35	43	648	1906	2511	0	1122

S113EC	88	5728	Eco605ORFMP	684	684	93.71	1	684	1828	2511	0	1015
S113EC	64	12303	Eco84137ORF201P	1635	22	95.45	558	579	3907	3886	0.11	36.2
S113EC	46	55587	Eco1520ORF67P	1563	18	100	1501	1518	53852	53869	0.1	36.2
S113EC	46	55587	Eco1520ORF67P	1563	15	100	1239	1253	21294	21280	6.4	30.2
S113EC	31	12213	Eco15ORF4165P	1197	33	87.88	237	269	6599	6567	0.32	34.2
S98EC	174	12336	M1.EcoMI	1623	1623	100	1	1623	3400	5022	0	3009
S98EC	174	12336	M1.EcoMI	1191	1191	100	1	1191	5015	6205	0	2361
S98EC	182	72266	M.EcoMII	1620	1581	100	40	1620	45676	44096	0	3092
S98EC	8	73638	M.EcoMIII	1638	1638	100	1	1638	62211	63848	0	3247
S98EC	8	73638	M.EcoMIII	1638	15	100	992	1006	66924	66938	6.7	30.2
S98EC	193	61632	M.EcoMIV	765	765	100	1	765	29712	28948	0	1516
S98EC	257	7669	M.EcoMV	1050	1050	100	1	1050	6587	7636	0	2040
S98EC	93	14722	M.EcoNwEVDcm	1419	1419	100	1	1419	8850	10268	0	2813
S98EC	266	12892	M.EcoMVI	891	891	100	1	891	11922	11032	0	1725
S98EC	172	66276	M.EcoNwEDam	837	837	100	1	837	39459	38623	0	1659
S98EC	45	95688	M.EcoMVII	3606	25	96	596	620	6121	6097	0.004	42.1
S98EC	45	95688	M.EcoMVII	3606	16	100	201	216	12937	12922	3.8	32.2
S98EC	178	5728	M.EcoMVIII	684	684	100	1	684	3975	3292	0	1356
S98EC	88	72526	Eco29kI	645	16	100	375	390	61122	61107	0.66	32.2
S98EC	45	95688	EcoDEC4CORF2749P	1041	16	100	654	669	47528	47543	1.1	32.2
S98EC	95	46942	Eco248534P	1053	16	100	941	956	18070	18055	1.1	32.2
S98EC	95	46942	Eco248534P	1053	15	100	66	80	21960	21946	4.3	30.2
S98EC	474	85489	EcoAPECORF2077P	1590	18	100	608	625	34161	34144	0.11	36.2
S98EC	474	85489	EcoAPECORF2077P	1590	17	100	1034	1050	40668	40652	0.42	34.2
S98EC	474	85489	EcoAPECORF2077P	1590	15	100	1543	1557	54520	54506	6.6	30.2
S98EC	474	85489	EcoAPECORF2077P	1590	15	100	668	682	60503	60489	6.6	30.2
S98EC	232	756	EcoDEC13EORF3046P	1191	16	100	920	935	800	815	1.2	32.2
S98EC	178	5728	Eco7A8ORF29P	684	606	98.51	79	684	3897	3292	0	1130
S98EC	55	41783	EcoDEC2CORF2043P	2019	21	95.24	1522	1542	17586	17606	0.53	34.2
S98EC	257	7669	Eco1886ORF14455P	1053	594	82.32	7	600	6593	7186	6e-95	345
S98EC	257	7669	Eco1886ORF14455P	1053	104	81.73	817	920	7403	7506	8e-08	56
S98EC	172	66276	Eco1886ORF14565P	900	15	100	30	44	6822	6808	3.7	30.2
S98EC	178	5728	EcoR7ACORFAP	684	684	97.37	1	684	3975	3292	0	1213
S98EC	178	5728	EcoR100ORF1P	648	606	98.51	43	648	3897	3292	0	1130
S98EC	178	5728	Eco605ORFMP	684	684	93.86	1	684	3975	3292	0	1023
S98EC	57	7709	Eco84137ORF201P	1635	27	92.59	811	837	2121	2147	0.028	38.2

S98EC	57	7709	Eco84137ORF201P	1635	20	95	550	569	1881	1900	1.7	32.2
S98EC	57	7709	Eco1520ORF67P	1563	1563	100	1	1563	1347	2909	0	3057
S98EC	174	12336	Eco15ORF4165P	1197	33	87.88	237	269	5689	5721	0.31	34.2
S97EC	78	33042	M1.EcoMI	1623	1623	100	1	1623	29717	28095	0	3009
S97EC	78	33042	M1.EcoMI	1191	1191	100	1	1191	28102	26912	0	2361
S97EC	32	116437	M.EcoMII	1620	1581	100	40	1620	70836	72416	0	3092
S97EC	7	181195	M.EcoMIII	1638	1638	100	1	1638	126252	127889	0	3247
S97EC	7	181195	M.EcoMIII	1638	15	100	739	753	56459	56473	6.9	30.2
S97EC	7	181195	M.EcoMIII	1638	15	100	992	1006	130965	130979	6.9	30.2
S97EC	74	70032	M.EcoMIV	765	765	100	1	765	65341	66105	0	1516
S97EC	279	7667	M.EcoMV	1050	1050	100	1	1050	1155	106	0	2040
S97EC	65	14722	M.EcoNwEVDcm	1419	1419	100	1	1419	5947	4529	0	2813
S97EC	33	91643	M.EcoMVI	891	891	100	1	891	11920	11030	0	1725
S97EC	25	171410	M.EcoNwEDam	837	837	100	1	837	39457	38621	0	1659
S97EC	235	289101	M.EcoMVII	3606	25	96	596	620	160157	160181	0.004	42.1
S97EC	235	289101	M.EcoMVII	3606	16	100	201	216	153341	153356	3.9	32.2
S97EC	38	27762	M.EcoMVIII	684	684	100	1	684	5305	4622	0	1356
S97EC	27	97408	Eco29kI	645	16	100	375	390	86000	85985	0.68	32.2
S97EC	27	97408	Eco29kI	645	15	100	494	508	9809	9823	2.7	30.2
S97EC	324	126669	EcoDEC4CORF2749P	1041	16	100	643	658	63049	63034	1.1	32.2
S97EC	276	95128	Eco248534P	1053	16	100	971	986	4992	4977	1.1	32.2
S97EC	276	95128	Eco248534P	1053	15	100	559	573	64923	64937	4.4	30.2
S97EC	68	57451	EcoAPECORF2077P	1590	18	100	608	625	27804	27821	0.11	36.2
S97EC	68	57451	EcoAPECORF2077P	1590	17	100	1034	1050	21297	21313	0.43	34.2
S97EC	68	57451	EcoAPECORF2077P	1590	15	100	668	682	1462	1476	6.7	30.2
S97EC	68	57451	EcoAPECORF2077P	1590	15	100	1543	1557	7445	7459	6.7	30.2
S97EC	169	27025	EcoDEC13EORF3046P	1191	16	100	920	935	42	57	1.3	32.2
S97EC	38	27762	Eco7A8ORF29P	684	606	98.51	79	684	5227	4622	0	1130
S97EC	276	95128	EcoDEC2CORF2043P	2019	2019	100	1	2019	29144	31162	0	4002
S97EC	276	95128	EcoDEC2CORF2043P	2019	24	91.67	139	162	29305	29282	2.2	32.2
S97EC	279	7667	Eco1886ORF14455P	1053	594	82.32	7	600	1149	556	6e-95	345
S97EC	279	7667	Eco1886ORF14455P	1053	104	81.73	817	920	339	236	8e-08	56
S97EC	276	95128	Eco1886ORF14565P	900	16	100	519	534	6083	6068	0.95	32.2
S97EC	38	27762	EcoR7ACORFAP	684	684	97.37	1	684	5305	4622	0	1213
S97EC	38	27762	EcoR100ORF1P	648	606	98.51	43	648	5227	4622	0	1130
S97EC	38	27762	Eco605ORFMP	684	684	93.86	1	684	5305	4622	0	1023

S97EC	235	289101	Eco84137ORF201P	1635	18	100	43	60	178530	178547	0.11	36.2
S97EC	235	289101	Eco84137ORF201P	1635	16	100	1212	1227	179817	179802	1.7	32.2
S97EC	235	289101	Eco84137ORF201P	1635	15	100	1080	1094	276473	276459	6.9	30.2
S97EC	106	170118	Eco1520ORF67P	1563	18	100	1532	1549	6981	6998	0.11	36.2
S97EC	78	33042	Eco15ORF4165P	1197	33	87.88	237	269	27428	27396	0.32	34.2
S122EC	385	6121	M1.EcoMI	1623	1623	100	1	1623	1659	37	0	3009
S122EC	223	4941	M1.EcoMI	1191	1191	100	1	1191	4988	3798	0	2361
S122EC	41	58681	M.EcoMII	1620	1581	100	40	1620	45764	47344	0	3092
S122EC	142	18414	M.EcoMIII	1638	1638	100	1	1638	11258	9621	0	3247
S122EC	142	18414	M.EcoMIII	1638	15	100	992	1006	6545	6531	6.9	30.2
S122EC	20	54083	M.EcoMIV	765	765	100	1	765	15571	16335	0	1516
S122EC	353	1794	M.EcoMV	1050	959	100	92	1050	1874	916	0	1859
S122EC	127	23542	M.EcoNwEVDcm	1419	1419	100	1	1419	14767	13349	0	2813
S122EC	64	51516	M.EcoMVI	891	891	100	1	891	1287	397	0	1725
S122EC	216	61162	M.EcoNwEDam	837	837	100	1	837	21778	22614	0	1659
S122EC	145	33796	M.EcoMVII	3606	25	96	596	620	3396	3372	0.004	42.1
S122EC	145	33796	M.EcoMVII	3606	16	100	201	216	10212	10197	3.8	32.2
S122EC	188	5734	M.EcoMVIII	684	684	100	1	684	1834	2517	0	1356
S122EC	205	5541	Eco29kI	645	16	100	238	253	4500	4515	0.67	32.2
S122EC	109	9256	EcoDEC4CORF2749P	1041	16	100	643	658	7413	7398	1.1	32.2
S122EC	125	38148	Eco248534P	1053	16	100	941	956	24260	24275	1.1	32.2
S122EC	125	38148	Eco248534P	1053	15	100	66	80	20370	20384	4.4	30.2
S122EC	160	32489	EcoAPECORF2077P	1590	18	100	1494	1511	22130	22113	0.11	36.2
S122EC	281	19942	EcoDEC13EORF3046P	1191	16	100	920	935	50	65	1.3	32.2
S122EC	188	5734	Eco7A8ORF29P	684	606	98.51	79	684	1912	2517	0	1130
S122EC	155	48639	EcoDEC2CORF2043P	2019	21	95.24	1522	1542	31157	31137	0.54	34.2
S122EC	353	1794	Eco1886ORF14455P	1053	495	83.43	106	600	1860	1366	9e-91	331
S122EC	353	1794	Eco1886ORF14455P	1053	104	81.73	817	920	1149	1046	8e-08	56
S122EC	297	28679	Eco1886ORF14565P	900	15	100	50	64	6654	6668	3.7	30.2
S122EC	188	5734	EcoR7ACORFAP	684	684	97.37	1	684	1834	2517	0	1213
S122EC	188	5734	EcoR100ORF1P	648	606	98.51	43	648	1912	2517	0	1130
S122EC	188	5734	Eco605ORFMP	684	684	93.86	1	684	1834	2517	0	1023
S122EC	134	7634	Eco84137ORF201P	1635	27	92.59	811	837	5588	5562	0.028	38.2
S122EC	134	7634	Eco84137ORF201P	1635	20	95	550	569	5828	5809	1.7	32.2
S122EC	134	7634	Eco1520ORF67P	1563	1563	100	1	1563	6362	4800	0	3057
S122EC	223	4941	Eco15ORF4165P	1197	33	87.88	237	269	4314	4282	0.32	34.2

MS2481	76	33046	M1.EcoMI	1623	1623	100	1	1623	3402	5024	0	3009
MS2481	76	33046	M1.EcoMI	1191	1191	100	1	1191	5017	6207	0	2361
MS2481	11	88539	M.EcoMII	1620	1581	100	40	1620	65997	67577	0	3092
MS2481	24	285048	M.EcoMIII	1638	1638	100	1	1638	73012	71375	0	3247
MS2481	24	285048	M.EcoMIII	1638	16	100	1336	1351	276062	276077	1.8	32.2
MS2481	24	285048	M.EcoMIII	1638	15	100	992	1006	68299	68285	7	30.2
MS2481	24	285048	M.EcoMIII	1638	15	100	739	753	142805	142791	7	30.2
MS2481	24	285048	M.EcoMIII	1638	15	100	1509	1523	243239	243253	7	30.2
MS2481	65	66256	M.EcoMIV	765	765	100	1	765	41762	42526	0	1516
MS2481	85	7671	M.EcoMV	1050	1050	100	1	1050	1159	110	0	2040
MS2481	98	38973	M.EcoNwEVDcm	1419	1419	100	1	1419	30198	28780	0	2813
MS2481	20	91699	M.EcoMVI	891	891	100	1	891	11924	11034	0	1725
MS2481	40	54381	M.EcoNwEDam	837	837	100	1	837	14997	15833	0	1659
MS2481	56	37940	M.EcoMVII	3606	25	96	596	620	10644	10668	0.004	42.1
MS2481	56	37940	M.EcoMVII	3606	16	100	201	216	3828	3843	3.9	32.2
MS2481	116	5730	M.EcoMVIII	684	684	100	1	684	3977	3294	0	1356
MS2481	61	52292	Eco29kI	645	16	100	238	253	19712	19727	0.68	32.2
MS2481	50	39344	EcoDEC4CORF2749P	1041	16	100	654	669	30836	30851	1.1	32.2
MS2481	86	31543	Eco248534P	1053	16	100	941	956	7368	7353	1.1	32.2
MS2481	86	31543	Eco248534P	1053	15	100	66	80	11258	11244	4.5	30.2
MS2481	112	164144	EcoAPECORF2077P	1590	18	100	1260	1277	138912	138929	0.11	36.2
MS2481	112	164144	EcoAPECORF2077P	1590	17	100	99	115	117922	117906	0.43	34.2
MS2481	112	164144	EcoAPECORF2077P	1590	17	100	1	17	143274	143258	0.43	34.2
MS2481	112	164144	EcoAPECORF2077P	1590	15	100	65	79	55805	55819	6.8	30.2
MS2481	112	164144	EcoAPECORF2077P	1590	15	100	1025	1039	93857	93843	6.8	30.2
MS2481	187	11030	EcoDEC13EORF3046P	1191	16	100	920	935	11061	11046	1.3	32.2
MS2481	116	5730	Eco7A8ORF29P	684	606	98.51	79	684	3899	3294	0	1130
MS2481	64	81884	EcoDEC2CORF2043P	2019	21	95.24	1522	1542	64403	64383	0.55	34.2
MS2481	85	7671	Eco1886ORF14455P	1053	594	82.32	7	600	1153	560	6e-95	345
MS2481	85	7671	Eco1886ORF14455P	1053	104	81.73	817	920	343	240	8e-08	56
MS2481	73	66289	Eco1886ORF14565P	900	16	100	399	414	61843	61858	0.96	32.2
MS2481	73	66289	Eco1886ORF14565P	900	19	94.74	620	638	55038	55020	3.8	30.2
MS2481	116	5730	EcoR7ACORFAP	684	684	97.37	1	684	3977	3294	0	1213
MS2481	116	5730	EcoR100ORF1P	648	606	98.51	43	648	3899	3294	0	1130
MS2481	116	5730	Eco605ORFMP	684	684	93.86	1	684	3977	3294	0	1023
MS2481	189	322	Eco84137ORF201P	1635	27	92.59	811	837	386	360	0.028	38.2

MS2481	188	2110	Eco1520ORF67P	1563	838	100	1	838	838	1	0	1620
MS2481	76	33046	Eco15ORF4165P	1197	33	87.88	237	269	5691	5723	0.32	34.2
S115EC	7	63398	M1.EcoMI	1623	1623	100	1	1623	60073	58451	0	3009
S115EC	7	63398	M1.EcoMI	1191	1191	100	1	1191	58458	57268	0	2361
S115EC	19	81930	M.EcoMII	1620	19	100	1333	1351	53689	53707	0.028	38.2
S115EC	94	92861	M.EcoMIII	1638	24	95.83	32	55	21097	21074	0.007	40.1
S115EC	94	92861	M.EcoMIII	1638	17	100	558	574	35719	35735	0.44	34.2
S115EC	94	92861	M.EcoMIII	1638	16	100	182	197	35102	35087	1.7	32.2
S115EC	94	92861	M.EcoMIII	1638	16	100	1369	1384	78454	78439	1.7	32.2
S115EC	94	92861	M.EcoMIII	1638	15	100	1274	1288	77674	77688	6.9	30.2
S115EC	2	150708	M.EcoMIV	765	765	100	1	765	92028	91264	0	1516
S115EC	138	25405	M.EcoMV	1050	1041	96.06	1	1041	16029	17069	0	1713
S115EC	11	38973	M.EcoNwEVDcm	1419	1419	100	1	1419	30198	28780	0	2813
S115EC	23	159010	M.EcoMVI	891	891	100	1	891	11922	11032	0	1725
S115EC	114	118828	M.EcoNwEDam	837	837	100	1	837	39459	38623	0	1659
S115EC	115	29267	M.EcoMVII	3606	25	96	596	620	911	887	0.004	42.1
S115EC	115	29267	M.EcoMVII	3606	16	100	201	216	7727	7712	3.9	32.2
S115EC	51	15519	M.EcoMVIII	684	684	97.08	1	684	3959	3276	0	1197
S115EC	70	13714	Eco29kI	645	16	100	375	390	11162	11177	0.68	32.2
S115EC	138	25405	EcoDEC4CORF2749P	1041	1041	100	1	1041	12569	13609	0	1974
S115EC	182	9720	Eco248534P	1053	16	100	262	277	4061	4046	1.1	32.2
S115EC	30	86249	EcoAPECORF2077P	1590	28	92.86	909	936	74342	74369	0.007	40.1
S115EC	30	86249	EcoAPECORF2077P	1590	18	100	99	116	73502	73519	0.11	36.2
S115EC	30	86249	EcoAPECORF2077P	1590	15	100	550	564	22703	22689	6.7	30.2
S115EC	30	86249	EcoAPECORF2077P	1590	15	100	71	85	35742	35756	6.7	30.2
S115EC	133	756	EcoDEC13EORF3046P	1191	16	100	920	935	800	815	1.3	32.2
S115EC	51	15519	Eco7A8ORF29P	684	621	96.46	64	684	3896	3276	0	1057
S115EC	121	14022	EcoDEC2CORF2043P	2019	21	95.24	1522	1542	10767	10747	0.55	34.2
S115EC	138	25405	Eco1886ORF14455P	1053	641	81.59	7	647	16035	16675	6e-92	335
S115EC	138	25405	Eco1886ORF14455P	1053	173	82.08	805	977	16833	17005	1e-12	71.9
S115EC	116	91331	Eco1886ORF14565P	900	15	100	565	579	51595	51609	3.8	30.2
S115EC	51	15519	EcoR7ACORFAP	684	684	96.35	1	684	3959	3276	0	1158
S115EC	51	15519	EcoR100ORF1P	648	621	96.46	28	648	3896	3276	0	1057
S115EC	51	15519	Eco605ORFMP	684	684	95.61	1	684	3959	3276	0	1118
S115EC	38	122897	Eco84137ORF201P	1635	18	100	43	60	17534	17551	0.11	36.2
S115EC	38	122897	Eco84137ORF201P	1635	16	100	1212	1227	18821	18806	1.7	32.2

S115EC	38	122897	Eco84137ORF201P	1635	15	100	1080	1094	115477	115463	6.9	30.2
S115EC	276	38256	Eco1520ORF67P	1563	18	100	1501	1518	12871	12854	0.11	36.2
S115EC	7	63398	Eco15ORF4165P	1197	33	87.88	237	269	57784	57752	0.32	34.2
S99EC	7	33018	M1.EcoMI	1623	1623	100	1	1623	3374	4996	0	3009
S99EC	7	33018	M1.EcoMI	1191	1191	100	1	1191	4989	6179	0	2361
S99EC	29	116232	M.EcoMII	1620	1581	100	40	1620	70632	72212	0	3092
S99EC	12	245053	M.EcoMIII	1638	1638	100	1	1638	9804	8167	0	3247
S99EC	12	245053	M.EcoMIII	1638	16	100	1336	1351	212854	212869	1.7	32.2
S99EC	12	245053	M.EcoMIII	1638	15	100	992	1006	5091	5077	6.7	30.2
S99EC	12	245053	M.EcoMIII	1638	15	100	739	753	79597	79583	6.7	30.2
S99EC	12	245053	M.EcoMIII	1638	15	100	1509	1523	180031	180045	6.7	30.2
S99EC	134	536	M.EcoMIV	765	584	99.83	67	650	584	1	0	1150
S99EC	95	186784	M.EcoMV	1050	1050	100	1	1050	182909	183958	0	2040
S99EC	95	186784	M.EcoMV	1050	16	100	115	130	82089	82074	1.1	32.2
S99EC	213	37036	M.EcoNwEVDcm	1419	1419	100	1	1419	8824	10242	0	2813
S99EC	42	158983	M.EcoMVI	891	891	100	1	891	147136	148026	0	1725
S99EC	35	190856	M.EcoNwEDam	837	837	100	1	837	151472	152308	0	1659
S99EC	15	370015	M.EcoMVII	3606	25	96	596	620	114811	114787	0.004	42.1
S99EC	15	370015	M.EcoMVII	3606	16	100	201	216	121627	121612	3.7	32.2
S99EC	34	5702	M.EcoMVIII	684	684	99.85	1	684	1802	2485	0	1348
S99EC	337	22753	Eco29kI	645	16	100	113	128	12083	12098	0.65	32.2
S99EC	15	370015	EcoDEC4CORF2749P	1041	16	100	654	669	156218	156233	1.1	32.2
S99EC	6	285305	Eco248534P	1053	16	100	941	956	51952	51967	1.1	32.2
S99EC	6	285305	Eco248534P	1053	15	100	66	80	48062	48076	4.3	30.2
S99EC	6	285305	Eco248534P	1053	15	100	972	986	177785	177771	4.3	30.2
S99EC	42	158983	EcoAPECORF2077P	1590	18	100	1511	1528	60850	60833	0.1	36.2
S99EC	42	158983	EcoAPECORF2077P	1590	17	100	1550	1566	113560	113544	0.41	34.2
S99EC	42	158983	EcoAPECORF2077P	1590	19	94.74	1493	1511	87428	87410	6.5	30.2
S99EC	42	158983	EcoAPECORF2077P	1590	15	100	50	64	149717	149703	6.5	30.2
S99EC	179	1385	EcoDEC13EORF3046P	1191	16	100	920	935	31	16	1.2	32.2
S99EC	34	5702	Eco7A8ORF29P	684	606	98.35	79	684	1880	2485	0	1122
S99EC	15	370015	EcoDEC2CORF2043P	2019	21	95.24	1522	1542	335271	335291	0.53	34.2
S99EC	15	370015	EcoDEC2CORF2043P	2019	15	100	459	473	8349	8363	8.2	30.2
S99EC	15	370015	EcoDEC2CORF2043P	2019	19	94.74	1813	1831	31630	31612	8.2	30.2
S99EC	95	186784	Eco1886ORF14455P	1053	594	82.32	7	600	182915	183508	6e-95	345
S99EC	95	186784	Eco1886ORF14455P	1053	104	81.73	817	920	183725	183828	7e-08	56

S99EC	337	22753	Eco1886ORF14565P	900	15	100	584	598	8147	8133	3.6	30.2
S99EC	34	5702	EcoR7ACORFAP	684	684	97.22	1	684	1802	2485	0	1205
S99EC	34	5702	EcoR100ORF1P	648	606	98.35	43	648	1880	2485	0	1122
S99EC	34	5702	Eco605ORFMP	684	684	93.71	1	684	1802	2485	0	1015
S99EC	24	31403	Eco84137ORF201P	1635	22	95.45	558	579	16643	16622	0.11	36.2
S99EC	95	186784	Eco1520ORF67P	1563	18	100	1501	1518	46435	46418	0.1	36.2
S99EC	95	186784	Eco1520ORF67P	1563	17	100	157	173	172108	172092	0.41	34.2
S99EC	95	186784	Eco1520ORF67P	1563	15	100	1239	1253	78993	79007	6.4	30.2
S99EC	7	33018	Eco15ORF4165P	1197	33	87.88	237	269	5663	5695	0.31	34.2
S133EC	31	32129	M1.EcoMI	1623	1623	100	1	1623	29718	28096	0	3009
S133EC	31	32129	M1.EcoMI	1191	1191	100	1	1191	28103	26913	0	2361
S133EC	10	340495	M.EcoMII	1620	1581	100	40	1620	294894	296474	0	3092
S133EC	10	340495	M.EcoMII	1620	16	100	748	763	159650	159665	1.7	32.2
S133EC	10	340495	M.EcoMII	1620	15	100	1592	1606	148093	148107	6.7	30.2
S133EC	1	309358	M.EcoMIII	1638	1638	100	1	1638	72875	71238	0	3247
S133EC	1	309358	M.EcoMIII	1638	16	100	1336	1351	275925	275940	1.7	32.2
S133EC	1	309358	M.EcoMIII	1638	15	100	992	1006	68162	68148	6.8	30.2
S133EC	1	309358	M.EcoMIII	1638	15	100	739	753	142668	142654	6.8	30.2
S133EC	1	309358	M.EcoMIII	1638	15	100	1509	1523	243102	243116	6.8	30.2
S133EC	4	301044	M.EcoMIV	765	765	100	1	765	65341	66105	0	1516
S133EC	17	7667	M.EcoMV	1050	1050	100	1	1050	6585	7634	0	2040
S133EC	83	38972	M.EcoNwEVDcm	1419	1419	100	1	1419	8848	10266	0	2813
S133EC	5	91685	M.EcoMVI	891	891	100	1	891	11920	11030	0	1725
S133EC	11	190880	M.EcoNwEDam	837	837	100	1	837	151496	152332	0	1659
S133EC	101	15805	M.EcoMVII	3606	3606	99.97	1	3606	6130	9735	0	6980
S133EC	71	5726	M.EcoMVIII	684	684	100	1	684	1826	2509	0	1356
S133EC	90	3643	Eco29kI	645	645	100	1	645	2156	1512	0	1237
S133EC	63	300354	EcoDEC4CORF2749P	1041	16	100	654	669	186613	186628	1.1	32.2
S133EC	80	397658	Eco248534P	1053	16	100	941	956	59706	59721	1.1	32.2
S133EC	80	397658	Eco248534P	1053	15	100	66	80	55816	55830	4.3	30.2
S133EC	80	397658	Eco248534P	1053	15	100	972	986	185539	185525	4.3	30.2
S133EC	264	109241	EcoAPECORF2077P	1590	18	100	608	625	58212	58195	0.11	36.2
S133EC	264	109241	EcoAPECORF2077P	1590	17	100	1034	1050	64719	64703	0.42	34.2
S133EC	264	109241	EcoAPECORF2077P	1590	15	100	1543	1557	78571	78557	6.6	30.2
S133EC	264	109241	EcoAPECORF2077P	1590	15	100	668	682	84554	84540	6.6	30.2
S133EC	213	162951	EcoDEC13EORF3046P	1191	16	100	8	23	3272	3287	1.2	32.2

S133EC	213	162951	EcoDEC13EORF3046P	1191	16	100	192	207	162571	162586	1.2	32.2
S133EC	71	5726	Eco7A8ORF29P	684	606	98.51	79	684	1904	2509	0	1130
S133EC	123	119367	EcoDEC2CORF2043P	2019	21	95.24	1522	1542	83584	83604	0.54	34.2
S133EC	17	7667	Eco1886ORF14455P	1053	594	82.32	7	600	6591	7184	6e-95	345
S133EC	17	7667	Eco1886ORF14455P	1053	104	81.73	817	920	7401	7504	8e-08	56
S133EC	301	219470	Eco1886ORF14565P	900	15	100	289	303	29685	29699	3.7	30.2
S133EC	301	219470	Eco1886ORF14565P	900	15	100	273	287	48869	48855	3.7	30.2
S133EC	71	5726	EcoR7ACORFAP	684	684	97.37	1	684	1826	2509	0	1213
S133EC	71	5726	EcoR100ORF1P	648	606	98.51	43	648	1904	2509	0	1130
S133EC	71	5726	Eco605ORFMP	684	684	93.86	1	684	1826	2509	0	1023
S133EC	49	7695	Eco84137ORF201P	1635	27	92.59	811	837	5649	5623	0.028	38.2
S133EC	49	7695	Eco84137ORF201P	1635	20	95	550	569	5889	5870	1.7	32.2
S133EC	49	7695	Eco1520ORF67P	1563	1563	100	1	1563	6423	4861	0	3057
S133EC	90	3643	Eco15ORF4165P	1197	20	100	1117	1136	471	452	0.005	40.1
S129EC	60	32135	M1.EcoMI	1623	1623	100	1	1623	29724	28102	0	3009
S129EC	60	32135	M1.EcoMI	1191	1191	100	1	1191	28109	26919	0	2361
S129EC	355	110501	M.EcoMII	1620	1581	100	40	1620	45680	44100	0	3092
S129EC	355	110501	M.EcoMII	1620	17	100	1558	1574	80515	80531	0.44	34.2
S129EC	3	142376	M.EcoMIII	1638	1638	100	1	1638	98849	100486	0	3247
S129EC	3	142376	M.EcoMIII	1638	15	100	739	753	29056	29070	7	30.2
S129EC	3	142376	M.EcoMIII	1638	15	100	992	1006	103562	103576	7	30.2
S129EC	20	152453	M.EcoMIV	765	765	100	1	765	101919	101155	0	1516
S129EC	26	8475	M.EcoMV	1050	1050	100	1	1050	6591	7640	0	2040
S129EC	16	10781	M.EcoNwEVDcm	1419	1419	100	1	1419	2006	588	0	2813
S129EC	37	81069	M.EcoMVI	891	891	100	1	891	79834	80724	0	1725
S129EC	25	84599	M.EcoNwEDam	837	837	100	1	837	39463	38627	0	1659
S129EC	54	30361	M.EcoMVII	3606	3606	99.97	1	3606	24304	20699	0	6980
S129EC	17	23234	M.EcoMVIII	684	684	100	1	684	3979	3296	0	1356
S129EC	86	108088	Eco29kI	645	16	100	238	253	62128	62113	0.68	32.2
S129EC	52	31002	EcoDEC4CORF2749P	1041	16	100	654	669	9840	9825	1.1	32.2
S129EC	337	111183	Eco248534P	1053	16	100	941	956	59667	59682	1.1	32.2
S129EC	337	111183	Eco248534P	1053	15	100	66	80	55777	55791	4.5	30.2
S129EC	42	74784	EcoAPECORF2077P	1590	18	100	1494	1511	24304	24287	0.11	36.2
S129EC	186	752	EcoDEC13EORF3046P	1191	16	100	920	935	800	815	1.3	32.2
S129EC	17	23234	Eco7A8ORF29P	684	606	98.51	79	684	3901	3296	0	1130
S129EC	39	92385	EcoDEC2CORF2043P	2019	2019	97.33	1	2019	29101	31119	0	3574

S129EC	39	92385	EcoDEC2CORF2043P	2019	24	91.67	139	162	29262	29239	2.2	32.2
S129EC	39	92385	EcoDEC2CORF2043P	2019	15	100	1332	1346	51211	51225	8.6	30.2
S129EC	355	110501	Eco1886ORF14455P	1053	1053	99.91	1	1053	85297	86349	0	2038
S129EC	39	92385	Eco1886ORF14565P	900	16	100	519	534	5616	5601	0.96	32.2
S129EC	17	23234	EcoR7ACORFAP	684	684	97.37	1	684	3979	3296	0	1213
S129EC	17	23234	EcoR100ORF1P	648	606	98.51	43	648	3901	3296	0	1130
S129EC	17	23234	Eco605ORFMP	684	684	93.86	1	684	3979	3296	0	1023
S129EC	160	7716	Eco84137ORF201P	1635	27	92.59	811	837	5670	5644	0.029	38.2
S129EC	160	7716	Eco84137ORF201P	1635	20	95	550	569	5910	5891	1.8	32.2
S129EC	160	7716	Eco1520ORF67P	1563	1563	100	1	1563	6444	4882	0	3057
S129EC	60	32135	Eco15ORF4165P	1197	33	87.88	237	269	27435	27403	0.32	34.2
S124EC	28	32133	M1.EcoMI	1623	1623	100	1	1623	29722	28100	0	3009
S124EC	28	32133	M1.EcoMI	1191	1191	100	1	1191	28107	26917	0	2361
S124EC	47	123437	M.EcoMII	1620	1581	100	40	1620	77836	79416	0	3092
S124EC	47	123437	M.EcoMII	1620	17	100	1558	1574	43001	42985	0.43	34.2
S124EC	6	177076	M.EcoMIII	1638	1638	100	1	1638	115440	117077	0	3247
S124EC	6	177076	M.EcoMIII	1638	15	100	739	753	45647	45661	6.8	30.2
S124EC	6	177076	M.EcoMIII	1638	15	100	992	1006	120153	120167	6.8	30.2
S124EC	58	121586	M.EcoMIV	765	765	100	1	765	56318	55554	0	1516
S124EC	121	7671	M.EcoMV	1050	1050	100	1	1050	6589	7638	0	2040
S124EC	36	11113	M.EcoNwEVDcm	1419	1419	100	1	1419	8852	10270	0	2813
S124EC	23	89086	M.EcoMVI	891	891	100	1	891	79787	80677	0	1725
S124EC	12	152355	M.EcoNwEDam	837	837	100	1	837	112971	113807	0	1659
S124EC	142	27204	M.EcoMVII	3606	3606	99.97	1	3606	21147	17542	0	6980
S124EC	76	5730	M.EcoMVIII	684	684	100	1	684	3977	3294	0	1356
S124EC	179	109127	Eco29kI	645	16	100	238	253	46037	46052	0.67	32.2
S124EC	284	193514	EcoDEC4CORF2749P	1041	16	100	654	669	68739	68754	1.1	32.2
S124EC	245	261823	Eco248534P	1053	16	100	941	956	59665	59680	1.1	32.2
S124EC	245	261823	Eco248534P	1053	15	100	66	80	55775	55789	4.4	30.2
S124EC	245	261823	Eco248534P	1053	15	100	972	986	185498	185484	4.4	30.2
S124EC	33	45597	EcoAPECORF2077P	1590	18	100	1260	1277	40834	40817	0.11	36.2
S124EC	33	45597	EcoAPECORF2077P	1590	17	100	1	17	36472	36488	0.42	34.2
S124EC	66	754	EcoDEC13EORF3046P	1191	16	100	920	935	800	815	1.3	32.2
S124EC	76	5730	Eco7A8ORF29P	684	606	98.51	79	684	3899	3294	0	1130
S124EC	24	55269	EcoDEC2CORF2043P	2019	2019	97.33	1	2019	29099	31117	0	3574
S124EC	24	55269	EcoDEC2CORF2043P	2019	24	91.67	139	162	29260	29237	2.1	32.2

S124EC	24	55269	EcoDEC2CORF2043P	2019	15	100	1332	1346	51209	51223	8.4	30.2
S124EC	47	123437	Eco1886ORF14455P	1053	1053	99.91	1	1053	38219	37167	0	2038
S124EC	24	55269	Eco1886ORF14565P	900	16	100	519	534	5614	5599	0.94	32.2
S124EC	76	5730	EcoR7ACORFAP	684	684	97.37	1	684	3977	3294	0	1213
S124EC	76	5730	EcoR100ORF1P	648	606	98.51	43	648	3899	3294	0	1130
S124EC	76	5730	Eco605ORFMP	684	684	93.86	1	684	3977	3294	0	1023
S124EC	54	6549	Eco84137ORF201P	1635	27	92.59	811	837	958	984	0.028	38.2
S124EC	54	6549	Eco84137ORF201P	1635	20	95	550	569	718	737	1.7	32.2
S124EC	54	6549	Eco1520ORF67P	1563	1563	100	1	1563	184	1746	0	3057
S124EC	28	32133	Eco15ORF4165P	1197	33	87.88	237	269	27433	27401	0.32	34.2
IR65	30	32418	M1.EcoMI	1623	1623	100	1	1623	29713	28091	0	3009
IR65	30	32418	M1.EcoMI	1191	1191	100	1	1191	28098	26908	0	2361
IR65	90	74104	M.EcoMII	1620	1581	100	40	1620	28503	30083	0	3092
IR65	228	307991	M.EcoMIII	1638	1638	100	1	1638	235189	236826	0	3247
IR65	228	307991	M.EcoMIII	1638	16	100	1336	1351	32139	32124	1.7	32.2
IR65	228	307991	M.EcoMIII	1638	15	100	1509	1523	64962	64948	6.8	30.2
IR65	228	307991	M.EcoMIII	1638	15	100	739	753	165396	165410	6.8	30.2
IR65	228	307991	M.EcoMIII	1638	15	100	992	1006	239902	239916	6.8	30.2
IR65	24	167803	M.EcoMIV	765	765	100	1	765	65337	66101	0	1516
IR65	286	168720	M.EcoMV	1050	1050	100	1	1050	159325	160374	0	2040
IR65	286	168720	M.EcoMV	1050	16	100	115	130	58505	58490	1.1	32.2
IR65	56	38985	M.EcoNwEVDcm	1419	1419	100	1	1419	8844	10262	0	2813
IR65	13	91680	M.EcoMVI	891	891	100	1	891	11916	11026	0	1725
IR65	17	107803	M.EcoNwEDam	837	837	100	1	837	39453	38617	0	1659
IR65	275	402442	M.EcoMVII	3606	25	96	596	620	256368	256392	0.004	42.1
IR65	275	402442	M.EcoMVII	3606	16	100	201	216	249664	249679	3.8	32.2
IR65	20	10930	M.EcoMVIII	684	684	100	1	684	1822	2505	0	1356
IR65	202	243240	Eco29kI	645	16	100	238	253	46029	46044	0.67	32.2
IR65	275	402442	EcoDEC4CORF2749P	1041	16	100	654	669	215073	215058	1.1	32.2
IR65	84	29806	Eco248534P	1053	1053	100	1	1053	7830	6778	0	2040
IR65	40	82264	EcoAPECORF2077P	1590	19	100	312	330	24426	24444	0.027	38.2
IR65	40	82264	EcoAPECORF2077P	1590	15	100	724	738	20726	20712	6.6	30.2
IR65	233	762	EcoDEC13EORF3046P	1191	16	100	920	935	800	815	1.3	32.2
IR65	20	10930	Eco7A8ORF29P	684	606	98.51	79	684	1900	2505	0	1130
IR65	275	402442	EcoDEC2CORF2043P	2019	21	95.24	1522	1542	35852	35832	0.54	34.2
IR65	275	402442	EcoDEC2CORF2043P	2019	19	94.74	1813	1831	339549	339567	8.5	30.2

IR65	275	402442	EcoDEC2CORF2043P	2019	15	100	459	473	362830	362816	8.5	30.2
IR65	40	82264	Eco1886ORF14455P	1053	1053	99.81	1	1053	18531	17479	0	2030
IR65	286	168720	Eco1886ORF14565P	900	15	100	831	845	60252	60266	3.7	30.2
IR65	20	10930	EcoR7ACORFAP	684	684	97.37	1	684	1822	2505	0	1213
IR65	20	10930	EcoR100ORF1P	648	606	98.51	43	648	1900	2505	0	1130
IR65	20	10930	Eco605ORFMP	684	684	93.86	1	684	1822	2505	0	1023
IR65	26	7634	Eco84137ORF201P	1635	27	92.59	811	837	2115	2141	0.028	38.2
IR65	26	7634	Eco84137ORF201P	1635	20	95	550	569	1875	1894	1.7	32.2
IR65	26	7634	Eco1520ORF67P	1563	1563	100	1	1563	1341	2903	0	3057
IR65	30	32418	Eco15ORF4165P	1197	33	87.88	237	269	27424	27392	0.32	34.2
IR68	23	32428	M1.EcoMI	1623	1623	100	1	1623	2784	4406	0	3009
IR68	23	32428	M1.EcoMI	1191	1191	100	1	1191	4399	5589	0	2361
IR68	37	116488	M.EcoMII	1620	1581	100	40	1620	70887	72467	0	3092
IR68	19	330952	M.EcoMIII	1638	1638	100	1	1638	258150	259787	0	3247
IR68	19	330952	M.EcoMIII	1638	16	100	1336	1351	55100	55085	1.7	32.2
IR68	19	330952	M.EcoMIII	1638	15	100	1509	1523	87923	87909	6.9	30.2
IR68	19	330952	M.EcoMIII	1638	15	100	739	753	188357	188371	6.9	30.2
IR68	19	330952	M.EcoMIII	1638	15	100	992	1006	262863	262877	6.9	30.2
IR68	17	167374	M.EcoMIV	765	765	100	1	765	64908	65672	0	1516
IR68	31	187017	M.EcoMV	1050	1050	100	1	1050	4101	3052	0	2040
IR68	31	187017	M.EcoMV	1050	16	100	115	130	104921	104936	1.1	32.2
IR68	64	38993	M.EcoNwEVDcm	1419	1419	100	1	1419	8854	10272	0	2813
IR68	3	91680	M.EcoMVI	891	891	100	1	891	11926	11036	0	1725
IR68	1	107813	M.EcoNwEDam	837	837	100	1	837	68429	69265	0	1659
IR68	155	364632	M.EcoMVII	3606	25	96	596	620	241166	241190	0.004	42.1
IR68	155	364632	M.EcoMVII	3606	16	100	201	216	234350	234365	3.8	32.2
IR68	68	8090	M.EcoMVIII	684	684	100	1	684	6337	5654	0	1356
IR68	65	62595	Eco29kI	645	16	100	113	128	52435	52450	0.67	32.2
IR68	155	364632	EcoDEC4CORF2749P	1041	16	100	654	669	199759	199744	1.1	32.2
IR68	65	62595	Eco248534P	1053	1053	100	1	1053	43132	42080	0	2040
IR68	65	62595	Eco248534P	1053	16	100	405	420	23524	23509	1.1	32.2
IR68	163	42106	EcoAPECORF2077P	1590	19	100	312	330	27848	27866	0.027	38.2
IR68	163	42106	EcoAPECORF2077P	1590	15	100	724	738	24148	24134	6.7	30.2
IR68	247	150250	EcoDEC13EORF3046P	1191	16	100	8	23	31238	31253	1.3	32.2
IR68	68	8090	Eco7A8ORF29P	684	606	98.51	79	684	6259	5654	0	1130
IR68	183	390161	EcoDEC2CORF2043P	2019	21	95.24	1522	1542	236624	236604	0.54	34.2

IR68	183	390161	EcoDEC2CORF2043P	2019	16	100	703	718	311888	311903	2.1	32.2
IR68	183	390161	EcoDEC2CORF2043P	2019	15	100	1262	1276	286569	286583	8.5	30.2
IR68	163	42106	Eco1886ORF14455P	1053	1053	99.81	1	1053	21953	20901	0	2030
IR68	247	150250	Eco1886ORF14565P	900	15	100	74	88	51574	51560	3.7	30.2
IR68	247	150250	Eco1886ORF14565P	900	15	100	67	81	79192	79206	3.7	30.2
IR68	68	8090	EcoR7ACORFAP	684	684	97.37	1	684	6337	5654	0	1213
IR68	68	8090	EcoR100ORF1P	648	606	98.51	43	648	6259	5654	0	1130
IR68	68	8090	Eco605ORFMP	684	684	93.86	1	684	6337	5654	0	1023
IR68	74	7644	Eco84137ORF201P	1635	27	92.59	811	837	5598	5572	0.028	38.2
IR68	74	7644	Eco84137ORF201P	1635	20	95	550	569	5838	5819	1.7	32.2
IR68	74	7644	Eco1520ORF67P	1563	1563	100	1	1563	6372	4810	0	3057
IR68	23	32428	Eco15ORF4165P	1197	33	87.88	237	269	5073	5105	0.32	34.2
IR49	37	32418	M1.EcoMI	1623	1623	100	1	1623	2774	4396	0	3009
IR49	37	32418	M1.EcoMI	1191	1191	100	1	1191	4389	5579	0	2361
IR49	26	74104	M.EcoMII	1620	1581	100	40	1620	28503	30083	0	3092
IR49	28	307991	M.EcoMIII	1638	1638	100	1	1638	235189	236826	0	3247
IR49	28	307991	M.EcoMIII	1638	16	100	1336	1351	32139	32124	1.7	32.2
IR49	28	307991	M.EcoMIII	1638	15	100	1509	1523	64962	64948	6.9	30.2
IR49	28	307991	M.EcoMIII	1638	15	100	739	753	165396	165410	6.9	30.2
IR49	28	307991	M.EcoMIII	1638	15	100	992	1006	239902	239916	6.9	30.2
IR49	13	167803	M.EcoMIV	765	765	100	1	765	65337	66101	0	1516
IR49	6	186951	M.EcoMV	1050	1050	100	1	1050	4091	3042	0	2040
IR49	6	186951	M.EcoMV	1050	16	100	115	130	104911	104926	1.1	32.2
IR49	43	38996	M.EcoNwEVDcm	1419	1419	100	1	1419	8844	10262	0	2813
IR49	11	158999	M.EcoMVI	891	891	100	1	891	147152	148042	0	1725
IR49	24	107803	M.EcoNwEDam	837	837	100	1	837	68419	69255	0	1659
IR49	10	378650	M.EcoMVII	3606	25	96	596	620	123535	123511	0.004	42.1
IR49	10	378650	M.EcoMVII	3606	16	100	201	216	129845	129830	3.8	32.2
IR49	30	10930	M.EcoMVIII	684	684	100	1	684	1822	2505	0	1356
IR49	241	276400	Eco29kI	645	16	100	238	253	46029	46044	0.67	32.2
IR49	28	307991	EcoDEC4CORF2749P	1041	16	100	643	658	45314	45299	1.1	32.2
IR49	17	28553	Eco248534P	1053	1053	100	1	1053	2031	3083	0	2040
IR49	17	28553	Eco248534P	1053	16	100	405	420	21639	21654	1.1	32.2
IR49	181	112008	EcoAPECORF2077P	1590	19	100	312	330	54170	54188	0.027	38.2
IR49	181	112008	EcoAPECORF2077P	1590	15	100	1200	1214	18276	18290	6.7	30.2
IR49	181	112008	EcoAPECORF2077P	1590	15	100	724	738	50470	50456	6.7	30.2

IR49	181	112008	EcoDEC13EORF3046P	1191	16	100	963	978	44760	44775	1.3	32.2
IR49	181	112008	EcoDEC13EORF3046P	1191	16	100	192	207	111628	111643	1.3	32.2
IR49	30	10930	Eco7A8ORF29P	684	606	98.51	79	684	1900	2505	0	1130
IR49	10	378650	EcoDEC2CORF2043P	2019	21	95.24	1522	1542	343783	343803	0.54	34.2
IR49	10	378650	EcoDEC2CORF2043P	2019	15	100	459	473	17073	17087	8.5	30.2
IR49	10	378650	EcoDEC2CORF2043P	2019	19	94.74	1813	1831	40354	40336	8.5	30.2
IR49	181	112008	Eco1886ORF14455P	1053	1053	99.81	1	1053	48275	47223	0	2030
IR49	75	15300	Eco1886ORF14565P	900	15	100	584	598	7783	7769	3.7	30.2
IR49	30	10930	EcoR7ACORFAP	684	684	97.37	1	684	1822	2505	0	1213
IR49	30	10930	EcoR100ORF1P	648	606	98.51	43	648	1900	2505	0	1130
IR49	30	10930	Eco605ORFMP	684	684	93.86	1	684	1822	2505	0	1023
IR49	73	7634	Eco84137ORF201P	1635	27	92.59	811	837	2115	2141	0.028	38.2
IR49	73	7634	Eco84137ORF201P	1635	20	95	550	569	1875	1894	1.7	32.2
IR49	73	7634	Eco1520ORF67P	1563	1563	100	1	1563	1341	2903	0	3057
IR49	37	32418	Eco15ORF4165P	1197	33	87.88	237	269	5063	5095	0.32	34.2
S77EC	102	259911	M1.EcoMI	1623	16	100	1596	1611	228904	228889	1.7	32.2
S77EC	102	259911	M1.EcoMI	1623	15	100	386	400	23634	23648	6.8	30.2
S77EC	102	259911	M1.EcoMI	1623	15	100	117	131	224731	224717	6.8	30.2
S77EC	151	189945	M1.EcoMI	1191	16	100	799	814	172999	173014	1.3	32.2
S77EC	151	189945	M.EcoMII	1620	19	100	1333	1351	42674	42656	0.028	38.2
S77EC	151	189945	M.EcoMII	1620	17	100	273	289	976	960	0.44	34.2
S77EC	151	189945	M.EcoMII	1620	20	95	1287	1306	124919	124900	1.7	32.2
S77EC	4	286776	M.EcoMIII	1638	1638	100	1	1638	213974	215611	0	3247
S77EC	4	286776	M.EcoMIII	1638	16	100	1336	1351	10924	10909	1.7	32.2
S77EC	4	286776	M.EcoMIII	1638	15	100	1509	1523	43747	43733	6.9	30.2
S77EC	4	286776	M.EcoMIII	1638	15	100	739	753	144181	144195	6.9	30.2
S77EC	4	286776	M.EcoMIII	1638	15	100	992	1006	218687	218701	6.9	30.2
S77EC	102	259911	M.EcoMIV	765	765	100	1	765	235780	235016	0	1516
S77EC	48	21157	M.EcoMV	1050	1050	100	1	1050	4099	3050	0	2040
S77EC	20	38973	M.EcoNwEVDcm	1419	1419	100	1	1419	30198	28780	0	2813
S77EC	14	91696	M.EcoMVI	891	891	100	1	891	11924	11034	0	1725
S77EC	26	163471	M.EcoNwEDam	837	837	100	1	837	39461	38625	0	1659
S77EC	123	204516	M.EcoMVII	3606	25	96	596	620	115155	115131	0.004	42.1
S77EC	123	204516	M.EcoMVII	3606	16	100	201	216	121971	121956	3.9	32.2
S77EC	78	13763	M.EcoMVIII	684	684	99.85	1	684	3977	3294	0	1348
S77EC	174	86008	Eco29kI	645	16	100	358	373	49961	49946	0.68	32.2

S77EC	123	204516	EcoDEC4CORF2749P	1041	16	100	654	669	156562	156577	1.1	32.2
S77EC	164	62593	Eco248534P	1053	1053	100	1	1053	43130	42078	0	2040
S77EC	164	62593	Eco248534P	1053	16	100	405	420	23522	23507	1.1	32.2
S77EC	189	173752	EcoAPECORF2077P	1590	18	100	1494	1511	149527	149544	0.11	36.2
S77EC	189	173752	EcoAPECORF2077P	1590	16	100	827	842	63584	63599	1.7	32.2
S77EC	189	173752	EcoAPECORF2077P	1590	15	100	414	428	5328	5314	6.7	30.2
S77EC	139	754	EcoDEC13EORF3046P	1191	16	100	920	935	31	16	1.3	32.2
S77EC	78	13763	Eco7A8ORF29P	684	606	98.35	79	684	3899	3294	0	1122
S77EC	45	23385	EcoDEC2CORF2043P	2019	2019	99.95	1	2019	20645	18627	0	3994
S77EC	45	23385	EcoDEC2CORF2043P	2019	24	91.67	139	162	20484	20507	2.2	32.2
S77EC	48	21157	Eco1886ORF14455P	1053	594	82.32	7	600	4093	3500 6e-95		345
S77EC	48	21157	Eco1886ORF14455P	1053	104	81.73	817	920	3283	3180 8e-08		56
S77EC	22	21963	Eco1886ORF14565P	900	16	100	519	534	3453	3438	0.95	32.2
S77EC	78	13763	EcoR7ACORFAP	684	684	97.22	1	684	3977	3294	0	1205
S77EC	78	13763	EcoR100ORF1P	648	606	98.35	43	648	3899	3294	0	1122
S77EC	78	13763	Eco605ORFMP	684	684	93.71	1	684	3977	3294	0	1015
S77EC	123	204516	Eco84137ORF201P	1635	18	100	43	60	96782	96765	0.11	36.2
S77EC	123	204516	Eco84137ORF201P	1635	16	100	1212	1227	95495	95510	1.7	32.2
S77EC	94	59248	Eco1520ORF67P	1563	18	100	1501	1518	46519	46502	0.11	36.2
S77EC	102	259911	Eco15ORF4165P	1197	16	100	280	295	39874	39859	1.3	32.2
IR18E	71	30780	M1.EcoMI	1623	1623	100	1	1623	29707	28085	0	3009
IR18E	71	30780	M1.EcoMI	1191	1191	100	1	1191	28092	26902	0	2361
IR18E	52	74098	M.EcoMII	1620	1581	100	40	1620	28497	30077	0	3092
IR18E	20	330930	M.EcoMIII	1638	1638	100	1	1638	72998	71361	0	3247
IR18E	20	330930	M.EcoMIII	1638	16	100	1336	1351	276048	276063	1.7	32.2
IR18E	20	330930	M.EcoMIII	1638	15	100	992	1006	68285	68271	6.8	30.2
IR18E	20	330930	M.EcoMIII	1638	15	100	739	753	142791	142777	6.8	30.2
IR18E	20	330930	M.EcoMIII	1638	15	100	1509	1523	243225	243239	6.8	30.2
IR18E	37	262010	M.EcoMIV	765	765	100	1	765	235974	235210	0	1516
IR18E	293	15037	M.EcoMV	1050	1050	100	1	1050	11015	12064	0	2040
IR18E	76	137758	M.EcoNwEVDcm	1419	1419	100	1	1419	30204	28786	0	2813
IR18E	21	127366	M.EcoMVI	891	891	100	1	891	11910	11020	0	1725
IR18E	23	65386	M.EcoNwEDam	837	837	100	1	837	26002	26838	0	1659
IR18E	173	378927	M.EcoMVII	3606	25	96	596	620	123529	123505	0.004	42.1
IR18E	173	378927	M.EcoMVII	3606	16	100	201	216	130124	130109	3.8	32.2
IR18E	96	9045	M.EcoMVIII	684	684	97.08	1	684	7290	6607	0	1197

IR18E	76	137758	Eco29kI	645	16	100	375	390	126340	126325	0.66	32.2
IR18E	76	137758	Eco29kI	645	15	100	494	508	50149	50163	2.6	30.2
IR18E	173	378927	EcoDEC4CORF2749P	1041	16	100	654	669	164715	164730	1.1	32.2
IR18E	182	533974	Eco248534P	1053	16	100	941	956	281596	281611	1.1	32.2
IR18E	182	533974	Eco248534P	1053	15	100	66	80	277706	277720	4.3	30.2
IR18E	182	533974	Eco248534P	1053	15	100	972	986	407429	407415	4.3	30.2
IR18E	145	109357	EcoAPECORF2077P	1590	18	100	608	625	51218	51235	0.11	36.2
IR18E	145	109357	EcoAPECORF2077P	1590	17	100	1034	1050	44711	44727	0.42	34.2
IR18E	145	109357	EcoAPECORF2077P	1590	15	100	668	682	24870	24884	6.6	30.2
IR18E	145	109357	EcoAPECORF2077P	1590	15	100	1543	1557	30853	30867	6.6	30.2
IR18E	76	137758	EcoDEC13EORF3046P	1191	16	100	920	935	40382	40397	1.2	32.2
IR18E	96	9045	Eco7A8ORF29P	684	621	96.46	64	684	7227	6607	0	1057
IR18E	182	533974	EcoDEC2CORF2043P	2019	21	95.24	1522	1542	466249	466229	0.54	34.2
IR18E	182	533974	EcoDEC2CORF2043P	2019	15	100	186	200	2590	2576	8.4	30.2
IR18E	182	533974	EcoDEC2CORF2043P	2019	15	100	489	503	158378	158364	8.4	30.2
IR18E	182	533974	EcoDEC2CORF2043P	2019	15	100	914	928	198178	198164	8.4	30.2
IR18E	182	533974	EcoDEC2CORF2043P	2019	15	100	1262	1276	516194	516208	8.4	30.2
IR18E	8	65669	Eco1886ORF14455P	1053	1053	99.15	1	1053	19107	18055	0	1974
IR18E	212	190649	Eco1886ORF14565P	900	15	100	884	898	48401	48415	3.7	30.2
IR18E	96	9045	EcoR7ACORFAP	684	684	96.35	1	684	7290	6607	0	1158
IR18E	96	9045	EcoR100ORF1P	648	621	96.46	28	648	7227	6607	0	1057
IR18E	96	9045	Eco605ORFMP	684	684	95.61	1	684	7290	6607	0	1118
IR18E	173	378927	Eco84137ORF201P	1635	18	100	43	60	105156	105139	0.11	36.2
IR18E	173	378927	Eco84137ORF201P	1635	16	100	1212	1227	103869	103884	1.7	32.2
IR18E	173	378927	Eco84137ORF201P	1635	16	100	751	766	374287	374302	1.7	32.2
IR18E	173	378927	Eco84137ORF201P	1635	15	100	1080	1094	7213	7227	6.8	30.2
IR18E	172	55769	Eco1520ORF67P	1563	18	100	1501	1518	35538	35555	0.1	36.2
IR18E	172	55769	Eco1520ORF67P	1563	15	100	1239	1253	2980	2966	6.5	30.2
IR18E	71	30780	Eco15ORF4165P	1197	33	87.88	237	269	27418	27386	0.32	34.2
HVM1299	66	30786	M1.EcoMI	1623	1623	100	1	1623	1142	2764	0	3009
HVM1299	66	30786	M1.EcoMI	1191	1191	99.92	1	1191	2757	3947	0	2353
HVM1299	79	74102	M.EcoMII	1620	1581	100	40	1620	28503	30083	0	3092
HVM1299	6	250077	M.EcoMIII	1638	1638	100	1	1638	232760	234397	0	3247
HVM1299	6	250077	M.EcoMIII	1638	16	100	1336	1351	29710	29695	1.7	32.2
HVM1299	6	250077	M.EcoMIII	1638	15	100	1509	1523	62533	62519	6.8	30.2
HVM1299	6	250077	M.EcoMIII	1638	15	100	739	753	162967	162981	6.8	30.2

HVM1299	6	250077	M.EcoMIII	1638	15	100	992	1006	237473	237487	6.8	30.2
HVM1299	13	191655	M.EcoMIV	765	765	100	1	765	65543	66307	0	1516
HVM1299	127	2907	M.EcoMV	1050	677	100	374	1050	1	677	0	1300
HVM1299	58	30516	M.EcoNwEVDcm	1419	1419	100	1	1419	30202	28784	0	2813
HVM1299	5	159003	M.EcoMVI	891	891	100	1	891	147156	148046	0	1725
HVM1299	7	190876	M.EcoNwEDam	837	837	100	1	837	151492	152328	0	1659
HVM1299	177	171652	M.EcoMVII	3606	25	96	596	620	48186	48210	0.004	42.1
HVM1299	177	171652	M.EcoMVII	3606	16	100	201	216	41370	41385	3.8	32.2
HVM1299	98	5708	M.EcoMVIII	684	684	97.08	1	684	3953	3270	0	1197
HVM1299	76	20636	Eco29kI	645	16	100	375	390	19658	19643	0.67	32.2
HVM1299	9	78186	EcoDEC4CORF2749P	1041	1041	100	1	1041	3975	2935	0	1974
HVM1299	199	41074	Eco248534P	1053	1053	100	1	1053	13418	14470	0	2040
HVM1299	199	41074	Eco248534P	1053	16	100	405	420	33026	33041	1.1	32.2
HVM1299	35	170624	EcoAPECORF2077P	1590	18	100	1494	1511	146399	146416	0.11	36.2
HVM1299	35	170624	EcoAPECORF2077P	1590	16	100	827	842	60445	60460	1.7	32.2
HVM1299	35	170624	EcoAPECORF2077P	1590	15	100	414	428	2189	2175	6.6	30.2
HVM1299	188	11965	EcoDEC13EORF3046P	1191	16	100	920	935	11996	11981	1.3	32.2
HVM1299	98	5708	Eco7A8ORF29P	684	621	96.46	64	684	3890	3270	0	1057
HVM1299	319	131326	EcoDEC2CORF2043P	2019	21	95.24	1522	1542	89	69	0.54	34.2
HVM1299	209	70219	Eco1886ORF14455P	1053	1053	99.15	1	1053	23657	22605	0	1974
HVM1299	197	11983	Eco1886ORF14565P	900	15	100	584	598	8167	8153	3.7	30.2
HVM1299	98	5708	EcoR7ACORFAP	684	684	96.35	1	684	3953	3270	0	1158
HVM1299	98	5708	EcoR100ORF1P	648	621	96.46	28	648	3890	3270	0	1057
HVM1299	98	5708	Eco605ORFMP	684	684	95.61	1	684	3953	3270	0	1118
HVM1299	177	171652	Eco84137ORF201P	1635	18	100	43	60	66559	66576	0.11	36.2
HVM1299	177	171652	Eco84137ORF201P	1635	16	100	1212	1227	67846	67831	1.7	32.2
HVM1299	177	171652	Eco84137ORF201P	1635	15	100	1080	1094	164502	164488	6.8	30.2
HVM1299	313	53567	Eco1520ORF67P	1563	18	100	1501	1518	32520	32537	0.11	36.2
HVM1299	66	30786	Eco15ORF4165P	1197	33	87.88	237	269	3431	3463	0.32	34.2
HVM3017	30	30774	M1.EcoMI	1623	1623	100	1	1623	1130	2752	0	3009
HVM3017	30	30774	M1.EcoMI	1191	1191	99.92	1	1191	2745	3935	0	2353
HVM3017	195	74049	M.EcoMII	1620	1581	100	40	1620	45656	44076	0	3092
HVM3017	361	275306	M.EcoMIII	1638	1638	100	1	1638	257989	259626	0	3247
HVM3017	361	275306	M.EcoMIII	1638	16	100	1336	1351	54939	54924	1.7	32.2
HVM3017	361	275306	M.EcoMIII	1638	15	100	1509	1523	87762	87748	6.8	30.2
HVM3017	361	275306	M.EcoMIII	1638	15	100	739	753	188196	188210	6.8	30.2

HVM3017	361	275306	M.EcoMIII	1638	15	100	992	1006	262702	262716	6.8	30.2
HVM3017	45	301024	M.EcoMIV	765	765	100	1	765	65321	66085	0	1516
HVM3017	53	2895	M.EcoMV	1050	665	100	386	1050	2951	2287	0	1277
HVM3017	64	104599	M.EcoNwEVDcm	1419	1419	100	1	1419	8832	10250	0	2813
HVM3017	38	91634	M.EcoMVI	891	891	100	1	891	79787	80677	0	1725
HVM3017	33	190864	M.EcoNwEDam	837	837	100	1	837	151480	152316	0	1659
HVM3017	187	348064	M.EcoMVII	3606	25	96	596	620	256594	256618	0.004	42.1
HVM3017	187	348064	M.EcoMVII	3606	16	100	201	216	249778	249793	3.8	32.2
HVM3017	68	15587	M.EcoMVIII	684	684	97.08	1	684	11703	12386	0	1197
HVM3017	64	104599	Eco29kI	645	16	100	375	390	50494	50509	0.67	32.2
HVM3017	100	44692	EcoDEC4CORF2749P	1041	1041	100	1	1041	4920	3880	0	1974
HVM3017	18	9669	Eco248534P	1053	1053	100	1	1053	1908	2960	0	2040
HVM3017	57	67266	EcoAPECORF2077P	1590	18	100	1511	1528	60858	60841	0.11	36.2
HVM3017	182	11847	EcoDEC13EORF3046P	1191	16	100	920	935	11878	11863	1.2	32.2
HVM3017	68	15587	Eco7A8ORF29P	684	621	96.46	64	684	11766	12386	0	1057
HVM3017	187	348064	EcoDEC2CORF2043P	2019	21	95.24	1522	1542	35840	35820	0.54	34.2
HVM3017	187	348064	EcoDEC2CORF2043P	2019	19	94.74	1813	1831	339775	339793	8.4	30.2
HVM3017	105	70371	Eco1886ORF14455P	1053	1053	99.15	1	1053	46619	47671	0	1974
HVM3017	249	187618	Eco1886ORF14565P	900	15	100	884	898	142303	142289	3.7	30.2
HVM3017	68	15587	EcoR7ACORFAP	684	684	96.35	1	684	11703	12386	0	1158
HVM3017	68	15587	EcoR100ORF1P	648	621	96.46	28	648	11766	12386	0	1057
HVM3017	68	15587	Eco605ORFMP	684	684	95.61	1	684	11703	12386	0	1118
HVM3017	187	348064	Eco84137ORF201P	1635	18	100	43	60	274967	274984	0.11	36.2
HVM3017	187	348064	Eco84137ORF201P	1635	16	100	751	766	5613	5598	1.7	32.2
HVM3017	187	348064	Eco84137ORF201P	1635	16	100	1212	1227	276254	276239	1.7	32.2
HVM3017	186	81396	Eco1520ORF67P	1563	18	100	1501	1518	44476	44459	0.11	36.2
HVM3017	186	81396	Eco1520ORF67P	1563	15	100	1239	1253	77034	77048	6.5	30.2
HVM3017	30	30774	Eco15ORF4165P	1197	33	87.88	237	269	3419	3451	0.32	34.2
S119EC	19	30788	M1.EcoMI	1623	1623	100	1	1623	29715	28093	0	3009
S119EC	19	30788	M1.EcoMI	1191	1191	99.92	1	1191	28100	26910	0	2353
S119EC	21	116371	M.EcoMII	1620	1581	100	40	1620	70770	72350	0	3092
S119EC	9	283734	M.EcoMIII	1638	1638	100	1	1638	266417	268054	0	3247
S119EC	9	283734	M.EcoMIII	1638	16	100	1336	1351	63431	63416	1.7	32.2
S119EC	9	283734	M.EcoMIII	1638	15	100	1509	1523	96254	96240	6.8	30.2
S119EC	9	283734	M.EcoMIII	1638	15	100	739	753	196625	196639	6.8	30.2
S119EC	9	283734	M.EcoMIII	1638	15	100	992	1006	271130	271144	6.8	30.2

S119EC	29	225250	M.EcoMIV	765	765	100	1	765	65339	66103	0	1516
S119EC	71	112157	M.EcoMV	1050	1050	100	1	1050	108807	109856	0	2040
S119EC	85	38977	M.EcoNwEVDcm	1419	1419	100	1	1419	30202	28784	0	2813
S119EC	8	159005	M.EcoMVI	891	891	100	1	891	147158	148048	0	1725
S119EC	28	171399	M.EcoNwEDam	837	837	100	1	837	132015	132851	0	1659
S119EC	16	369893	M.EcoMVII	3606	25	96	596	620	254869	254893	0.004	42.1
S119EC	16	369893	M.EcoMVII	3606	16	100	201	216	248162	248177	3.8	32.2
S119EC	45	15601	M.EcoMVIII	684	684	97.08	1	684	11717	12400	0	1197
S119EC	237	222492	Eco29kI	645	16	100	238	253	46279	46294	0.67	32.2
S119EC	71	112157	EcoDEC4CORF2749P	1041	1041	100	1	1041	105347	106387	0	1974
S119EC	71	112157	EcoDEC4CORF2749P	1041	19	94.74	326	344	29475	29493	4.3	30.2
S119EC	264	44080	Eco248534P	1053	1053	100	1	1053	13420	14472	0	2040
S119EC	264	44080	Eco248534P	1053	16	100	405	420	33028	33043	1.1	32.2
S119EC	225	173755	EcoAPECORF2077P	1590	18	100	1494	1511	149530	149547	0.11	36.2
S119EC	225	173755	EcoAPECORF2077P	1590	16	100	827	842	63576	63591	1.7	32.2
S119EC	225	173755	EcoAPECORF2077P	1590	15	100	414	428	5320	5306	6.6	30.2
S119EC	117	760	EcoDEC13EORF3046P	1191	16	100	920	935	31	16	1.3	32.2
S119EC	45	15601	Eco7A8ORF29P	684	621	96.46	64	684	11780	12400	0	1057
S119EC	16	369893	EcoDEC2CORF2043P	2019	21	95.24	1522	1542	34224	34204	0.54	34.2
S119EC	16	369893	EcoDEC2CORF2043P	2019	19	94.74	1813	1831	338050	338068	8.4	30.2
S119EC	16	369893	EcoDEC2CORF2043P	2019	15	100	459	473	361331	361317	8.4	30.2
S119EC	71	112157	Eco1886ORF14455P	1053	594	82.32	7	600	108813	109406	6e-95	345
S119EC	71	112157	Eco1886ORF14455P	1053	104	81.73	817	920	109623	109726	8e-08	56
S119EC	71	112157	Eco1886ORF14455P	1053	16	100	883	898	54112	54127	1.1	32.2
S119EC	206	76171	Eco1886ORF14565P	900	15	100	50	64	69598	69584	3.7	30.2
S119EC	45	15601	EcoR7ACORFAP	684	684	96.35	1	684	11717	12400	0	1158
S119EC	45	15601	EcoR100ORF1P	648	621	96.46	28	648	11780	12400	0	1057
S119EC	45	15601	Eco605ORFMP	684	684	95.61	1	684	11717	12400	0	1118
S119EC	206	76171	Eco84137ORF201P	1635	22	95.45	558	579	24656	24635	0.11	36.2
S119EC	74	175589	Eco1520ORF67P	1563	18	100	1501	1518	44490	44473	0.11	36.2
S119EC	74	175589	Eco1520ORF67P	1563	17	100	157	173	170163	170147	0.42	34.2
S119EC	74	175589	Eco1520ORF67P	1563	15	100	1239	1253	77048	77062	6.5	30.2
S119EC	19	30788	Eco15ORF4165P	1197	33	87.88	237	269	27426	27394	0.32	34.2
S118EC	30	30786	M1.EcoMI	1623	1623	100	1	1623	1142	2764	0	3009
S118EC	30	30786	M1.EcoMI	1191	1191	99.92	1	1191	2757	3947	0	2353
S118EC	20	74104	M.EcoMII	1620	1581	100	40	1620	28503	30083	0	3092

S118EC	8	275493	M.EcoMIII	1638	1638	100	1	1638	17386	15749	0	3247
S118EC	8	275493	M.EcoMIII	1638	16	100	1336	1351	220372	220387	1.7	32.2
S118EC	8	275493	M.EcoMIII	1638	15	100	992	1006	12673	12659	6.8	30.2
S118EC	8	275493	M.EcoMIII	1638	15	100	739	753	87178	87164	6.8	30.2
S118EC	8	275493	M.EcoMIII	1638	15	100	1509	1523	187549	187563	6.8	30.2
S118EC	25	301248	M.EcoMIV	765	765	100	1	765	235980	235216	0	1516
S118EC	10	112096	M.EcoMV	1050	1050	100	1	1050	108817	109866	0	2040
S118EC	77	38973	M.EcoNwEVDcm	1419	1419	100	1	1419	8844	10262	0	2813
S118EC	6	159003	M.EcoMVI	891	891	100	1	891	147156	148046	0	1725
S118EC	22	190876	M.EcoNwEDam	837	837	100	1	837	39453	38617	0	1659
S118EC	168	378678	M.EcoMVII	3606	25	96	596	620	123535	123511	0.004	42.1
S118EC	168	378678	M.EcoMVII	3606	16	100	201	216	130351	130336	3.8	32.2
S118EC	44	15599	M.EcoMVIII	684	684	97.08	1	684	3953	3270	0	1197
S118EC	10	112096	Eco29kI	645	16	100	375	390	11487	11502	0.67	32.2
S118EC	10	112096	Eco29kI	645	15	100	494	508	87678	87664	2.6	30.2
S118EC	10	112096	EcoDEC4CORF2749P	1041	1041	100	1	1041	105357	106397	0	1974
S118EC	10	112096	EcoDEC4CORF2749P	1041	19	94.74	326	344	29485	29503	4.3	30.2
S118EC	221	44078	Eco248534P	1053	1053	100	1	1053	30729	29677	0	2040
S118EC	221	44078	Eco248534P	1053	16	100	405	420	11121	11106	1.1	32.2
S118EC	258	109259	EcoAPECORF2077P	1590	18	100	608	625	58224	58207	0.11	36.2
S118EC	258	109259	EcoAPECORF2077P	1590	17	100	1034	1050	64731	64715	0.42	34.2
S118EC	258	109259	EcoAPECORF2077P	1590	15	100	1543	1557	78589	78575	6.6	30.2
S118EC	258	109259	EcoAPECORF2077P	1590	15	100	668	682	84572	84558	6.6	30.2
S118EC	69	13194	EcoDEC13EORF3046P	1191	16	100	920	935	38	53	1.3	32.2
S118EC	44	15599	Eco7A8ORF29P	684	621	96.46	64	684	3890	3270	0	1057
S118EC	168	378678	EcoDEC2CORF2043P	2019	21	95.24	1522	1542	344289	344309	0.54	34.2
S118EC	168	378678	EcoDEC2CORF2043P	2019	15	100	459	473	17073	17087	8.4	30.2
S118EC	168	378678	EcoDEC2CORF2043P	2019	19	94.74	1813	1831	40354	40336	8.4	30.2
S118EC	10	112096	Eco1886ORF14455P	1053	594	82.32	7	600	108823	109416	6e-95	345
S118EC	10	112096	Eco1886ORF14455P	1053	104	81.73	817	920	109633	109736	8e-08	56
S118EC	10	112096	Eco1886ORF14455P	1053	16	100	883	898	54122	54137	1.1	32.2
S118EC	297	226212	Eco1886ORF14565P	900	15	100	289	303	36428	36442	3.7	30.2
S118EC	297	226212	Eco1886ORF14565P	900	15	100	273	287	55612	55598	3.7	30.2
S118EC	44	15599	EcoR7ACORFAP	684	684	96.35	1	684	3953	3270	0	1158
S118EC	44	15599	EcoR100ORF1P	648	621	96.46	28	648	3890	3270	0	1057
S118EC	44	15599	Eco605ORFMP	684	684	95.61	1	684	3953	3270	0	1118

S118EC	168	378678	Eco84137ORF201P	1635	18	100	43	60	105162	105145	0.11	36.2
S118EC	168	378678	Eco84137ORF201P	1635	16	100	1212	1227	103875	103890	1.7	32.2
S118EC	168	378678	Eco84137ORF201P	1635	16	100	751	766	374516	374531	1.7	32.2
S118EC	168	378678	Eco84137ORF201P	1635	15	100	1080	1094	7219	7233	6.8	30.2
S118EC	134	165344	Eco1520ORF67P	1563	18	100	1501	1518	39853	39836	0.11	36.2
S118EC	134	165344	Eco1520ORF67P	1563	15	100	1239	1253	72411	72425	6.5	30.2
S118EC	30	30786	Eco15ORF4165P	1197	33	87.88	237	269	3431	3463	0.32	34.2
S65EC	296	34185	M1.EcoMI	1623	1623	100	1	1623	29723	28101	0	3009
S65EC	296	34185	M1.EcoMI	1191	1191	100	1	1191	28108	26918	0	2361
S65EC	18	45788	M.EcoMII	1620	1581	100	40	1620	37573	35993	0	3092
S65EC	11	247099	M.EcoMIII	1638	1638	100	1	1638	174297	175934	0	3247
S65EC	11	247099	M.EcoMIII	1638	15	100	1509	1523	4070	4056	6.8	30.2
S65EC	11	247099	M.EcoMIII	1638	15	100	739	753	104504	104518	6.8	30.2
S65EC	11	247099	M.EcoMIII	1638	15	100	992	1006	179010	179024	6.8	30.2
S65EC	49	101945	M.EcoMIV	765	765	100	1	765	10026	10790	0	1516
S65EC	106	20651	M.EcoMV	1050	1050	100	1	1050	4101	3052	0	2040
S65EC	160	12558	M.EcoNwEVDcm	1419	1419	100	1	1419	8854	10272	0	2813
S65EC	21	91692	M.EcoMVI	891	891	100	1	891	79845	80735	0	1725
S65EC	36	143474	M.EcoNwEDam	837	837	100	1	837	113582	114418	0	1659
S65EC	35	59896	M.EcoMVII	3606	25	96	596	620	10564	10588	0.004	42.1
S65EC	35	59896	M.EcoMVII	3606	16	100	201	216	3748	3763	3.8	32.2
S65EC	145	9750	M.EcoMVIII	684	684	100	1	684	2218	1535	0	1356
S65EC	235	65108	Eco29kI	645	16	100	113	128	50986	50971	0.67	32.2
S65EC	143	14100	EcoDEC4CORF2749P	1041	16	100	643	658	8330	8345	1.1	32.2
S65EC	235	65108	Eco248534P	1053	1053	100	1	1053	43132	42080	0	2040
S65EC	235	65108	Eco248534P	1053	16	100	405	420	23524	23509	1.1	32.2
S65EC	91	9888	EcoAPECORF2077P	1590	18	100	1260	1277	4822	4839	0.11	36.2
S65EC	91	9888	EcoAPECORF2077P	1590	17	100	1	17	9184	9168	0.42	34.2
S65EC	161	752	EcoDEC13EORF3046P	1191	16	100	920	935	31	16	1.2	32.2
S65EC	145	9750	Eco7A8ORF29P	684	606	98.51	79	684	2140	1535	0	1130
S65EC	192	189913	EcoDEC2CORF2043P	2019	21	95.24	1522	1542	153628	153648	0.54	34.2
S65EC	192	189913	EcoDEC2CORF2043P	2019	16	100	703	718	78364	78349	2.1	32.2
S65EC	192	189913	EcoDEC2CORF2043P	2019	15	100	1262	1276	103683	103669	8.4	30.2
S65EC	106	20651	Eco1886ORF14455P	1053	594	82.32	7	600	4095	3502	6e-95	345
S65EC	106	20651	Eco1886ORF14455P	1053	104	81.73	817	920	3285	3182	8e-08	56
S65EC	371	42715	Eco1886ORF14565P	900	15	100	289	303	15828	15814	3.7	30.2

S65EC	145	9750	EcoR7ACORFAP	684	684	97.37	1	684	2218	1535	0	1213
S65EC	145	9750	EcoR100ORF1P	648	606	98.51	43	648	2140	1535	0	1130
S65EC	145	9750	Eco605ORFMP	684	684	93.86	1	684	2218	1535	0	1023
S65EC	132	7716	Eco84137ORF201P	1635	27	92.59	811	837	5670	5644	0.028	38.2
S65EC	132	7716	Eco84137ORF201P	1635	20	95	550	569	5910	5891	1.7	32.2
S65EC	132	7716	Eco1520ORF67P	1563	1563	99.94	1	1563	6444	4882	0	3049
S65EC	296	34185	Eco15ORF4165P	1197	33	87.88	237	269	27434	27402	0.32	34.2
S103EC	88	32308	M1.EcoMI	1623	1623	100	1	1623	2664	4286	0	3009
S103EC	88	32308	M1.EcoMI	1191	1191	100	1	1191	4279	5469	0	2361
S103EC	79	77352	M.EcoMII	1620	1581	100	40	1620	70837	72417	0	3092
S103EC	13	39922	M.EcoMIII	1638	1638	100	1	1638	32441	34078	0	3247
S103EC	13	39922	M.EcoMIII	1638	15	100	992	1006	37154	37168	6.8	30.2
S103EC	83	41001	M.EcoMIV	765	765	100	1	765	31065	30301	0	1516
S103EC	215	7494	M.EcoMV	1050	1050	100	1	1050	6412	7461	0	2040
S103EC	52	12552	M.EcoNwEVDcm	1419	1419	100	1	1419	8848	10266	0	2813
S103EC	96	41397	M.EcoMVI	891	891	100	1	891	29785	30675	0	1725
S103EC	40	71660	M.EcoNwEDam	837	837	100	1	837	32276	33112	0	1659
S103EC	264	67494	M.EcoMVII	3606	25	96	596	620	36996	37020	0.004	42.1
S103EC	264	67494	M.EcoMVII	3606	16	100	201	216	30181	30196	3.8	32.2
S103EC	3	11505	M.EcoMVIII	684	684	100	1	684	7605	8288	0	1356
S103EC	81	28924	Eco29kI	645	16	100	375	390	17516	17501	0.67	32.2
S103EC	72	79983	EcoDEC4CORF2749P	1041	16	100	643	658	64407	64392	1.1	32.2
S103EC	59	32040	Eco248534P	1053	16	100	941	956	12784	12769	1.1	32.2
S103EC	59	32040	Eco248534P	1053	15	100	66	80	16674	16660	4.3	30.2
S103EC	153	17827	EcoAPECORF2077P	1590	18	100	1260	1277	4078	4095	0.11	36.2
S103EC	153	17827	EcoAPECORF2077P	1590	17	100	1	17	8440	8424	0.42	34.2
S103EC	27	52832	EcoDEC13EORF3046P	1191	23	95.65	377	399	6970	6948	0.02	38.2
S103EC	3	11505	Eco7A8ORF29P	684	606	98.51	79	684	7683	8288	0	1130
S103EC	58	75014	EcoDEC2CORF2043P	2019	21	95.24	1522	1542	69220	69200	0.54	34.2
S103EC	215	7494	Eco1886ORF14455P	1053	594	82.32	7	600	6418	7011	6e-95	345
S103EC	215	7494	Eco1886ORF14455P	1053	104	81.73	817	920	7228	7331	8e-08	56
S103EC	345	104993	Eco1886ORF14565P	900	15	100	74	88	9227	9213	3.7	30.2
S103EC	345	104993	Eco1886ORF14565P	900	15	100	67	81	36854	36868	3.7	30.2
S103EC	3	11505	EcoR7ACORFAP	684	684	97.37	1	684	7605	8288	0	1213
S103EC	3	11505	EcoR100ORF1P	648	606	98.51	43	648	7683	8288	0	1130
S103EC	3	11505	Eco605ORFMP	684	684	93.86	1	684	7605	8288	0	1023

S103EC	28	7581	Eco84137ORF201P	1635	27	92.59	811	837	2119	2145	0.028	38.2
S103EC	28	7581	Eco84137ORF201P	1635	20	95	550	569	1879	1898	1.7	32.2
S103EC	28	7581	Eco1520ORF67P	1563	1563	99.94	1	1563	1345	2907	0	3049
S103EC	88	32308	Eco15ORF4165P	1197	33	87.88	237	269	4953	4985	0.32	34.2
S125EC	268	17580	M1.EcoMI	1623	1623	100	1	1623	4545	6167	0	3009
S125EC	268	17580	M1.EcoMI	1191	1191	100	1	1191	6160	7350	0	2361
S125EC	100	9050	M.EcoMII	1620	875	100	40	914	8258	9132	0	1693
S125EC	76	17848	M.EcoMIII	1638	1638	100	1	1638	5057	3420	0	3247
S125EC	76	17848	M.EcoMIII	1638	15	100	992	1006	344	330	6.9	30.2
S125EC	79	48936	M.EcoMIV	765	765	100	1	765	24259	25023	0	1516
S125EC	372	1744	M.EcoMV	1050	909	100	142	1050	1	909	0	1760
S125EC	56	20363	M.EcoNwEVDcm	1419	1419	100	1	1419	11588	10170	0	2813
S125EC	128	34536	M.EcoMVI	891	891	100	1	891	22689	23579	0	1725
S125EC	44	46024	M.EcoNwEDam	837	837	100	1	837	6640	7476	0	1659
S125EC	52	59375	M.EcoMVII	3606	25	96	596	620	17541	17565	0.004	42.1
S125EC	52	59375	M.EcoMVII	3606	16	100	201	216	10725	10740	3.8	32.2
S125EC	232	8057	M.EcoMVIII	684	606	98.51	79	684	4274	4879	0	1130
S125EC	46	63627	Eco29kI	645	16	100	238	253	17667	17652	0.67	32.2
S125EC	208	5907	EcoDEC4CORF2749P	1041	21	95.24	157	177	5570	5590	0.28	34.2
S125EC	43	6563	Eco248534P	1053	16	100	565	580	3546	3531	1.1	32.2
S125EC	104	58594	EcoAPECORF2077P	1590	18	100	1494	1511	24308	24291	0.11	36.2
S125EC	273	748	EcoDEC13EORF3046P	1191	16	100	920	935	31	16	1.3	32.2
S125EC	232	8057	Eco7A8ORF29P	684	684	100	1	684	4196	4879	0	1356
S125EC	13	91128	EcoDEC2CORF2043P	2019	1095	96.44	907	2001	31631	32725	0	1861
S125EC	13	91128	EcoDEC2CORF2043P	2019	723	99.86	1	723	30479	31201	0	1425
S125EC	13	91128	EcoDEC2CORF2043P	2019	24	91.67	139	162	30640	30617	2.1	32.2
S125EC	372	1744	Eco1886ORF14455P	1053	456	83.77	145	600	4	459	1e-86	317
S125EC	372	1744	Eco1886ORF14455P	1053	104	81.73	817	920	676	779	8e-08	56
S125EC	237	142459	Eco1886ORF14565P	900	15	100	831	845	91295	91281	3.7	30.2
S125EC	232	8057	EcoR7ACORFAP	684	621	98.55	64	684	4259	4879	0	1160
S125EC	232	8057	EcoR100ORF1P	648	648	100	1	648	4232	4879	0	1285
S125EC	232	8057	Eco605ORFMP	684	621	95.17	64	684	4259	4879	0	993
S125EC	52	59375	Eco84137ORF201P	1635	18	100	43	60	35914	35931	0.11	36.2
S125EC	52	59375	Eco84137ORF201P	1635	16	100	1212	1227	37201	37186	1.7	32.2
S125EC	382	122411	Eco1520ORF67P	1563	18	100	1532	1549	39832	39849	0.11	36.2
S125EC	268	17580	Eco15ORF4165P	1197	33	87.88	237	269	6834	6866	0.32	34.2

S96EC	217	33019	M1.EcoMI	1623	1623	100	1	1623	29694	28072	0	3009
S96EC	217	33019	M1.EcoMI	1191	1191	100	1	1191	28079	26889	0	2361
S96EC	30	74046	M.EcoMII	1620	1581	100	40	1620	45653	44073	0	3092
S96EC	19	150664	M.EcoMIII	1638	1638	100	1	1638	72855	71218	0	3247
S96EC	19	150664	M.EcoMIII	1638	15	100	992	1006	68142	68128	6.8	30.2
S96EC	19	150664	M.EcoMIII	1638	15	100	739	753	142648	142634	6.8	30.2
S96EC	38	4737	M.EcoMIV	765	765	100	1	765	1558	2322	0	1516
S96EC	22	451099	M.EcoMV	1050	594	82.49	7	600	23654	23061	2e-97	353
S96EC	22	451099	M.EcoMV	1050	104	80.77	817	920	22844	22741	2e-05	48.1
S96EC	22	451099	M.EcoMV	1050	16	100	622	637	79661	79646	1.1	32.2
S96EC	22	451099	M.EcoMV	1050	15	100	631	645	247018	247004	4.3	30.2
S96EC	26	35978	M.EcoNwEVDcm	1419	1419	100	1	1419	27203	25785	0	2813
S96EC	17	158987	M.EcoMVI	891	891	100	1	891	147140	148030	0	1725
S96EC	46	190860	M.EcoNwEDam	837	837	100	1	837	39437	38601	0	1659
S96EC	34	411038	M.EcoMVII	3606	25	96	596	620	109733	109709	0.004	42.1
S96EC	34	411038	M.EcoMVII	3606	16	100	201	216	116440	116425	3.8	32.2
S96EC	43	23208	M.EcoMVIII	684	684	100	1	684	3953	3270	0	1356
S96EC	154	276382	Eco29kI	645	16	100	238	253	230422	230407	0.66	32.2
S96EC	34	411038	EcoDEC4CORF2749P	1041	16	100	654	669	151031	151046	1.1	32.2
S96EC	22	451099	Eco248534P	1053	16	100	941	956	169437	169422	1.1	32.2
S96EC	22	451099	Eco248534P	1053	15	100	972	986	43604	43618	4.3	30.2
S96EC	22	451099	Eco248534P	1053	15	100	66	80	173327	173313	4.3	30.2
S96EC	103	225651	EcoAPECORF2077P	1590	18	100	1260	1277	138672	138689	0.11	36.2
S96EC	103	225651	EcoAPECORF2077P	1590	17	100	99	115	117682	117666	0.42	34.2
S96EC	103	225651	EcoAPECORF2077P	1590	17	100	1	17	143034	143018	0.42	34.2
S96EC	103	225651	EcoAPECORF2077P	1590	15	100	65	79	55573	55587	6.6	30.2
S96EC	103	225651	EcoAPECORF2077P	1590	15	100	1025	1039	93617	93603	6.6	30.2
S96EC	103	225651	EcoAPECORF2077P	1590	15	100	669	683	183508	183494	6.6	30.2
S96EC	78	1381	EcoDEC13EORF3046P	1191	16	100	920	935	1403	1418	1.2	32.2
S96EC	43	23208	Eco7A8ORF29P	684	606	98.51	79	684	3875	3270	0	1130
S96EC	221	214259	EcoDEC2CORF2043P	2019	21	95.24	1522	1542	153255	153275	0.54	34.2
S96EC	221	214259	EcoDEC2CORF2043P	2019	16	100	703	718	77991	77976	2.1	32.2
S96EC	221	214259	EcoDEC2CORF2043P	2019	15	100	1262	1276	103310	103296	8.4	30.2
S96EC	22	451099	Eco1886ORF14455P	1053	1053	99.34	1	1053	23660	22608	0	1990
S96EC	176	202698	Eco1886ORF14565P	900	15	100	884	898	142307	142293	3.7	30.2
S96EC	43	23208	EcoR7ACORFAP	684	684	97.37	1	684	3953	3270	0	1213

S96EC	43	23208	EcoR100ORF1P	648	606	98.51	43	648	3875	3270	0	1130
S96EC	43	23208	Eco605ORFMP	684	684	93.86	1	684	3953	3270	0	1023
S96EC	110	264	Eco84137ORF201P	1635	27	92.59	811	837	269	243	0.028	38.2
S96EC	77	2051	Eco1520ORF67P	1563	779	100	1	779	1325	2103	0	1503
S96EC	217	33019	Eco15ORF4165P	1197	33	87.88	237	269	27405	27373	0.32	34.2
S130EC	79	33052	M1.EcoMI	1623	1623	100	1	1623	3408	5030	0	3009
S130EC	79	33052	M1.EcoMI	1191	1191	100	1	1191	5023	6213	0	2361
S130EC	113	71609	M.EcoMII	1620	845	100	40	884	70847	71691	0	1633
S130EC	44	46373	M.EcoMIII	1638	1638	100	1	1638	12781	11144	0	3247
S130EC	44	46373	M.EcoMIII	1638	15	100	992	1006	8068	8054	6.8	30.2
S130EC	19	102771	M.EcoMIV	765	765	100	1	765	96758	95994	0	1516
S130EC	223	3846	M.EcoMV	1050	1050	100	1	1050	1798	2847	0	2040
S130EC	97	27025	M.EcoNwEVDcm	1419	1419	100	1	1419	8858	10276	0	2813
S130EC	71	83463	M.EcoMVI	891	891	100	1	891	11930	11040	0	1725
S130EC	121	47815	M.EcoNwEDam	837	837	100	1	837	29272	30108	0	1659
S130EC	54	53829	M.EcoMVII	3606	25	96	596	620	10568	10592	0.004	42.1
S130EC	54	53829	M.EcoMVII	3606	16	100	201	216	3752	3767	3.8	32.2
S130EC	156	5686	M.EcoMVIII	684	684	100	1	684	1836	2519	0	1356
S130EC	43	35255	Eco29kI	645	16	100	238	253	21628	21613	0.67	32.2
S130EC	7	72474	EcoDEC4CORF2749P	1041	16	100	643	658	34389	34374	1.1	32.2
S130EC	26	113608	Eco248534P	1053	16	100	941	956	77438	77423	1.1	32.2
S130EC	26	113608	Eco248534P	1053	15	100	66	80	81328	81314	4.3	30.2
S130EC	175	5162	EcoAPECORF2077P	1590	18	100	1260	1277	397	380	0.11	36.2
S130EC	253	748	EcoDEC13EORF3046P	1191	16	100	920	935	800	815	1.2	32.2
S130EC	156	5686	Eco7A8ORF29P	684	606	98.51	79	684	1914	2519	0	1130
S130EC	98	23711	EcoDEC2CORF2043P	2019	21	95.24	1522	1542	3290	3310	0.54	34.2
S130EC	223	3846	Eco1886ORF14455P	1053	594	82.32	7	600	1804	2397	6e-95	345
S130EC	223	3846	Eco1886ORF14455P	1053	104	81.73	817	920	2614	2717	8e-08	56
S130EC	160	63654	Eco1886ORF14565P	900	15	100	884	898	3482	3468	3.7	30.2
S130EC	156	5686	EcoR7ACORFAP	684	684	97.37	1	684	1836	2519	0	1213
S130EC	156	5686	EcoR100ORF1P	648	606	98.51	43	648	1914	2519	0	1130
S130EC	156	5686	Eco605ORFMP	684	684	93.86	1	684	1836	2519	0	1023
S130EC	186	5071	Eco84137ORF201P	1635	27	92.59	811	837	398	424	0.028	38.2
S130EC	162	5107	Eco1520ORF67P	1563	1186	100	378	1563	5189	4004	0	2309
S130EC	79	33052	Eco15ORF4165P	1197	33	87.88	237	269	5697	5729	0.32	34.2
S109EC	91	32304	M1.EcoMI	1623	1623	100	1	1623	2660	4282	0	3009

S109EC	91	32304	M1.EcoMI	1191	1191	100	1	1191	4275	5465	0	2361
S109EC	105	74324	M.EcoMII	1620	1581	100	40	1620	45890	44310	0	3092
S109EC	5	35905	M.EcoMIII	1638	1638	100	1	1638	7790	9427	0	3247
S109EC	5	35905	M.EcoMIII	1638	15	100	992	1006	12503	12517	6.7	30.2
S109EC	54	58338	M.EcoMIV	765	765	100	1	765	25784	25020	0	1516
S109EC	8	74701	M.EcoMV	1050	1050	100	1	1050	70679	71728	0	2040
S109EC	157	26524	M.EcoNwEVDcm	1419	1419	100	1	1419	1630	3048	0	2813
S109EC	43	81060	M.EcoMVI	891	891	100	1	891	1287	397	0	1725
S109EC	111	49783	M.EcoNwEDam	837	837	100	1	837	45204	46040	0	1659
S109EC	18	105352	M.EcoMVII	3606	25	96	596	620	92359	92383	0.004	42.1
S109EC	18	105352	M.EcoMVII	3606	16	100	201	216	85543	85558	3.8	32.2
S109EC	131	4216	M.EcoMVIII	684	684	100	1	684	1822	2505	0	1356
S109EC	52	3626	Eco29kI	645	645	100	1	645	2176	1532	0	1237
S109EC	92	23408	EcoDEC4CORF2749P	1041	16	100	643	658	17522	17537	1.1	32.2
S109EC	263	246880	Eco248534P	1053	16	100	941	956	85837	85822	1.1	32.2
S109EC	263	246880	Eco248534P	1053	15	100	66	80	89727	89713	4.3	30.2
S109EC	144	4097	EcoAPECORF2077P	1590	18	100	1431	1448	189	206	0.11	36.2
S109EC	144	4097	EcoAPECORF2077P	1590	15	100	808	822	4053	4039	6.5	30.2
S109EC	113	34648	EcoDEC13EORF3046P	1191	23	95.65	377	399	11741	11719	0.02	38.2
S109EC	131	4216	Eco7A8ORF29P	684	606	98.51	79	684	1900	2505	0	1130
S109EC	56	150832	EcoDEC2CORF2043P	2019	21	95.24	1522	1542	52051	52071	0.53	34.2
S109EC	56	150832	EcoDEC2CORF2043P	2019	15	100	1262	1276	2106	2092	8.3	30.2
S109EC	8	74701	Eco1886ORF14455P	1053	594	82.32	7	600	70685	71278	6e-95	345
S109EC	8	74701	Eco1886ORF14455P	1053	104	81.73	817	920	71495	71598	8e-08	56
S109EC	263	246880	Eco1886ORF14565P	900	15	100	176	190	128921	128907	3.7	30.2
S109EC	263	246880	Eco1886ORF14565P	900	15	100	289	303	177697	177711	3.7	30.2
S109EC	263	246880	Eco1886ORF14565P	900	15	100	273	287	196881	196867	3.7	30.2
S109EC	131	4216	EcoR7ACORFAP	684	684	97.37	1	684	1822	2505	0	1213
S109EC	131	4216	EcoR100ORF1P	648	606	98.51	43	648	1900	2505	0	1130
S109EC	286	81883	Eco605ORFMP	684	684	99.56	1	684	79581	80264	0	1332
S109EC	103	18693	Eco84137ORF201P	1635	22	95.45	558	579	2099	2120	0.11	36.2
S109EC	64	98999	Eco1520ORF67P	1563	18	100	1532	1549	87474	87457	0.1	36.2
S109EC	52	3626	Eco15ORF4165P	1197	20	100	1117	1136	491	472	0.005	40.1
S111EC	13	32304	M1.EcoMI	1623	1623	100	1	1623	29713	28091	0	3009
S111EC	13	32304	M1.EcoMI	1191	1191	100	1	1191	28098	26908	0	2361
S111EC	26	54621	M.EcoMII	1620	1581	100	40	1620	9020	10600	0	3092

S111EC	104	261663	M.EcoMIII	1638	1638	100	1	1638	258239	259876	0	3247
S111EC	104	261663	M.EcoMIII	1638	16	100	1336	1351	55189	55174	1.7	32.2
S111EC	104	261663	M.EcoMIII	1638	15	100	1509	1523	88012	87998	6.7	30.2
S111EC	104	261663	M.EcoMIII	1638	15	100	739	753	188446	188460	6.7	30.2
S111EC	75	225220	M.EcoMIV	765	765	100	1	765	65337	66101	0	1516
S111EC	6	102121	M.EcoMV	1050	1050	100	1	1050	98099	99148	0	2040
S111EC	53	11053	M.EcoNwEVDcm	1419	1419	100	1	1419	2278	860	0	2813
S111EC	44	127344	M.EcoMVI	891	891	100	1	891	115497	116387	0	1725
S111EC	17	209761	M.EcoNwEDam	837	837	100	1	837	170377	171213	0	1659
S111EC	259	344293	M.EcoMVII	3606	25	96	596	620	220827	220851	0.004	42.1
S111EC	259	344293	M.EcoMVII	3606	16	100	201	216	214011	214026	3.8	32.2
S111EC	79	5722	M.EcoMVIII	684	684	100	1	684	1822	2505	0	1356
S111EC	72	96404	Eco29kI	645	16	100	375	390	84994	84979	0.66	32.2
S111EC	72	96404	Eco29kI	645	15	100	494	508	8803	8817	2.6	30.2
S111EC	259	344293	EcoDEC4CORF2749P	1041	16	100	654	669	179420	179405	1.1	32.2
S111EC	18	313305	Eco248534P	1053	16	100	941	956	55496	55481	1.1	32.2
S111EC	18	313305	Eco248534P	1053	15	100	66	80	59386	59372	4.3	30.2
S111EC	211	109357	EcoAPECORF2077P	1590	18	100	608	625	51218	51235	0.11	36.2
S111EC	211	109357	EcoAPECORF2077P	1590	17	100	1034	1050	44711	44727	0.42	34.2
S111EC	211	109357	EcoAPECORF2077P	1590	15	100	668	682	24876	24890	6.5	30.2
S111EC	211	109357	EcoAPECORF2077P	1590	15	100	1543	1557	30859	30873	6.5	30.2
S111EC	106	22755	EcoDEC13EORF3046P	1191	23	95.65	377	399	11741	11719	0.02	38.2
S111EC	79	5722	Eco7A8ORF29P	684	606	98.51	79	684	1900	2505	0	1130
S111EC	259	344293	EcoDEC2CORF2043P	2019	21	95.24	1522	1542	71	51	0.53	34.2
S111EC	259	344293	EcoDEC2CORF2043P	2019	19	94.74	1813	1831	304008	304026	8.3	30.2
S111EC	259	344293	EcoDEC2CORF2043P	2019	15	100	459	473	327289	327275	8.3	30.2
S111EC	6	102121	Eco1886ORF14455P	1053	594	82.32	7	600	98105	98698	6e-95	345
S111EC	6	102121	Eco1886ORF14455P	1053	104	81.73	817	920	98915	99018	7e-08	56
S111EC	259	344293	Eco1886ORF14565P	900	15	100	565	579	140875	140861	3.7	30.2
S111EC	79	5722	EcoR7ACORFAP	684	684	97.37	1	684	1822	2505	0	1213
S111EC	79	5722	EcoR100ORF1P	648	606	98.51	43	648	1900	2505	0	1130
S111EC	79	5722	Eco605ORFMP	684	684	93.86	1	684	1822	2505	0	1023
S111EC	17	209761	Eco84137ORF201P	1635	1635	100	1	1635	50205	51839	0	3241
S111EC	17	209761	Eco84137ORF201P	1635	17	100	1054	1070	147653	147669	0.43	34.2
S111EC	17	209761	Eco84137ORF201P	1635	15	100	1102	1116	59734	59748	6.7	30.2
S111EC	17	209761	Eco84137ORF201P	1635	15	100	1330	1344	178085	178071	6.7	30.2

S111EC	17	209761	Eco1520ORF67P	1563	27	92.59	775	801	51015	51041	0.026	38.2
S111EC	17	209761	Eco1520ORF67P	1563	20	95	535	554	50754	50773	1.6	32.2
S111EC	17	209761	Eco1520ORF67P	1563	19	94.74	1242	1260	157112	157094	6.4	30.2
S111EC	13	32304	Eco15ORF4165P	1197	33	87.88	237	269	27424	27392	0.31	34.2
S121EC	37	30686	M1.EcoMI	1623	1623	100	1	1623	4543	6165	0	3009
S121EC	37	30686	M1.EcoMI	1191	1191	100	1	1191	6158	7348	0	2361
S121EC	12	114874	M.EcoMII	1620	1581	100	40	1620	70735	72315	0	3092
S121EC	47	74286	M.EcoMIII	1638	1638	100	1	1638	1364	3001	0	3247
S121EC	47	74286	M.EcoMIII	1638	15	100	992	1006	6077	6091	6.7	30.2
S121EC	87	96340	M.EcoMIV	765	765	100	1	765	31072	30308	0	1516
S121EC	331	3570	M.EcoMV	1050	1050	100	1	1050	1965	916	0	2040
S121EC	129	38973	M.EcoNwEVDcm	1419	1419	100	1	1419	30198	28780	0	2813
S121EC	74	70915	M.EcoMVI	891	891	100	1	891	11928	11038	0	1725
S121EC	91	41526	M.EcoNwEDam	837	837	100	1	837	18614	17778	0	1659
S121EC	16	155858	M.EcoMVII	3606	25	96	596	620	135466	135490	0.004	42.1
S121EC	16	155858	M.EcoMVII	3606	16	100	201	216	128650	128665	3.8	32.2
S121EC	125	5686	M.EcoMVIII	684	684	100	1	684	1834	2517	0	1356
S121EC	18	97414	Eco29kI	645	16	100	375	390	11488	11503	0.66	32.2
S121EC	18	97414	Eco29kI	645	15	100	494	508	87678	87664	2.6	30.2
S121EC	25	112173	EcoDEC4CORF2749P	1041	16	100	643	658	38138	38153	1.1	32.2
S121EC	27	29495	Eco248534P	1053	16	100	941	956	8771	8756	1.1	32.2
S121EC	27	29495	Eco248534P	1053	15	100	66	80	12661	12647	4.3	30.2
S121EC	148	173767	EcoAPECORF2077P	1590	18	100	1494	1511	24306	24289	0.11	36.2
S121EC	148	173767	EcoAPECORF2077P	1590	16	100	827	842	110260	110245	1.6	32.2
S121EC	148	173767	EcoAPECORF2077P	1590	15	100	414	428	168516	168530	6.5	30.2
S121EC	209	17609	EcoDEC13EORF3046P	1191	16	100	920	935	17640	17625	1.2	32.2
S121EC	125	5686	Eco7A8ORF29P	684	606	98.51	79	684	1912	2517	0	1130
S121EC	9	42652	EcoDEC2CORF2043P	2019	21	95.24	1522	1542	20410	20390	0.53	34.2
S121EC	331	3570	Eco1886ORF14455P	1053	594	82.32	7	600	1959	1366	6e-95	345
S121EC	331	3570	Eco1886ORF14455P	1053	104	81.73	817	920	1149	1046	7e-08	56
S121EC	118	24792	Eco1886ORF14565P	900	15	100	272	286	4066	4052	3.7	30.2
S121EC	125	5686	EcoR7ACORFAP	684	684	97.37	1	684	1834	2517	0	1213
S121EC	125	5686	EcoR100ORF1P	648	606	98.51	43	648	1912	2517	0	1130
S121EC	125	5686	Eco605ORFMP	684	684	93.86	1	684	1834	2517	0	1023
S121EC	126	27844	Eco84137ORF201P	1635	1635	100	1	1635	10227	11861	0	3241
S121EC	126	27844	Eco84137ORF201P	1635	15	100	1102	1116	19755	19769	6.7	30.2

S121EC	126	27844	Eco1520ORF67P	1563	27	92.59	775	801	11037	11063	0.026	38.2
S121EC	126	27844	Eco1520ORF67P	1563	20	95	535	554	10776	10795	1.6	32.2
S121EC	37	30686	Eco15ORF4165P	1197	33	87.88	237	269	6832	6864	0.31	34.2
S15EC	321	24833	M1.EcoMI	1623	1623	100	1	1623	592	2214	0	3009
S15EC	321	24833	M1.EcoMI	1191	1191	100	1	1191	2207	3397	0	2361
S15EC	373	5752	M.EcoMII	1620	806	100	815	1620	1	806	0	1598
S15EC	112	44873	M.EcoMIII	1638	1638	100	1	1638	513	2150	0	3247
S15EC	112	44873	M.EcoMIII	1638	15	100	992	1006	5226	5240	6.7	30.2
S15EC	62	34920	M.EcoMIV	765	765	100	1	765	15597	16361	0	1516
S15EC	640	1521	M.EcoMV	1050	679	100	372	1050	8	686	0	1304
S15EC	98	7809	M.EcoNwEVDcm	1419	1419	100	1	1419	5540	6958	0	2813
S15EC	2	21565	M.EcoMVI	891	891	100	1	891	11930	11040	0	1725
S15EC	576	1413	M.EcoNwEDam	837	713	100	1	713	783	1495	0	1413
S15EC	158	30775	M.EcoMVII	3606	25	96	596	620	23624	23600	0.004	42.1
S15EC	158	30775	M.EcoMVII	3606	16	100	201	216	30440	30425	3.8	32.2
S15EC	67	5682	M.EcoMVIII	684	684	100	1	684	1836	2519	0	1356
S15EC	408	15500	Eco29kI	645	16	100	375	390	4385	4370	0.66	32.2
S15EC	702	36225	EcoDEC4CORF2749P	1041	20	95	157	176	2557	2576	1.1	32.2
S15EC	702	36225	EcoDEC4CORF2749P	1041	15	100	90	104	9325	9311	4.2	30.2
S15EC	374	17600	Eco248534P	1053	16	100	941	956	1407	1422	1.1	32.2
S15EC	231	34660	EcoAPECORF2077P	1590	18	100	1511	1528	5648	5665	0.11	36.2
S15EC	555	748	EcoDEC13EORF3046P	1191	16	100	920	935	800	815	1.2	32.2
S15EC	67	5682	Eco7A8ORF29P	684	606	98.51	79	684	1914	2519	0	1130
S15EC	104	17648	EcoDEC2CORF2043P	2019	21	95.24	1522	1542	14201	14221	0.53	34.2
S15EC	691	2499	Eco1886ORF14455P	1053	416	84.62	7	422	2147	2562	1e-86	317
S15EC	379	5439	Eco1886ORF14565P	900	15	100	272	286	4001	3987	3.7	30.2
S15EC	67	5682	EcoR7ACORFAP	684	684	97.37	1	684	1836	2519	0	1213
S15EC	67	5682	EcoR100ORF1P	648	606	98.51	43	648	1914	2519	0	1130
S15EC	67	5682	Eco605ORFMP	684	684	93.86	1	684	1836	2519	0	1023
S15EC	631	18307	Eco84137ORF201P	1635	22	95.45	558	579	17412	17391	0.11	36.2
S15EC	343	11053	Eco1520ORF67P	1563	18	100	1501	1518	10660	10677	0.1	36.2
S15EC	321	24833	Eco15ORF4165P	1197	33	87.88	237	269	2881	2913	0.31	34.2
S131EC	42	33207	M1.EcoMI	1623	1623	100	1	1623	3563	5185	0	3009
S131EC	42	33207	M1.EcoMI	1191	1191	100	1	1191	5178	6368	0	2361
S131EC	86	116439	M.EcoMII	1620	1581	100	40	1620	45676	44096	0	3092
S131EC	30	257193	M.EcoMIII	1638	1638	100	1	1638	184391	186028	0	3247

S131EC	30	257193	M.EcoMIII	1638	15	100	1509	1523	14164	14150	6.7	30.2
S131EC	30	257193	M.EcoMIII	1638	15	100	739	753	114598	114612	6.7	30.2
S131EC	30	257193	M.EcoMIII	1638	15	100	992	1006	189104	189118	6.7	30.2
S131EC	20	177118	M.EcoMIV	765	765	100	1	765	126159	125395	0	1516
S131EC	71	22080	M.EcoMV	1050	1050	100	1	1050	18058	19107	0	2040
S131EC	127	35568	M.EcoNwEVDcm	1419	1419	100	1	1419	8850	10268	0	2813
S131EC	34	34525	M.EcoMVI	891	891	100	1	891	22678	23568	0	1725
S131EC	66	75915	M.EcoNwEDam	837	837	100	1	837	39459	38623	0	1659
S131EC	201	437542	M.EcoMVII	3606	25	96	596	620	322464	322488	0.004	42.1
S131EC	201	437542	M.EcoMVII	3606	16	100	3337	3352	65198	65213	3.8	32.2
S131EC	201	437542	M.EcoMVII	3606	16	100	201	216	315648	315663	3.8	32.2
S131EC	203	794	M.EcoMVIII	684	16	100	410	425	841	826	0.7	32.2
S131EC	80	70381	Eco29kI	645	16	100	375	390	58973	58958	0.66	32.2
S131EC	201	437542	EcoDEC4CORF2749P	1041	16	100	654	669	281057	281042	1.1	32.2
S131EC	2	74105	Eco248534P	1053	16	100	941	956	52002	52017	1.1	32.2
S131EC	2	74105	Eco248534P	1053	15	100	66	80	48112	48126	4.3	30.2
S131EC	72	57779	EcoAPECORF2077P	1590	18	100	1511	1528	51373	51356	0.11	36.2
S131EC	154	756	EcoDEC13EORF3046P	1191	16	100	920	935	800	815	1.2	32.2
S131EC	57	23140	Eco7A8ORF29P	684	16	100	265	280	9137	9152	0.7	32.2
S131EC	201	437542	EcoDEC2CORF2043P	2019	21	95.24	1522	1542	101710	101690	0.53	34.2
S131EC	201	437542	EcoDEC2CORF2043P	2019	16	100	910	925	79581	79566	2.1	32.2
S131EC	201	437542	EcoDEC2CORF2043P	2019	19	94.74	1813	1831	405645	405663	8.3	30.2
S131EC	201	437542	EcoDEC2CORF2043P	2019	15	100	459	473	428926	428912	8.3	30.2
S131EC	71	22080	Eco1886ORF14455P	1053	594	82.32	7	600	18064	18657	6e-95	345
S131EC	71	22080	Eco1886ORF14455P	1053	104	81.73	817	920	18874	18977	8e-08	56
S131EC	201	437542	Eco1886ORF14565P	900	15	100	565	579	242512	242498	3.7	30.2
S131EC	1	102594	EcoR7ACORFAP	684	21	95.24	181	201	17177	17197	0.18	34.2
S131EC	57	23140	EcoR100ORF1P	648	16	100	229	244	9137	9152	0.66	32.2
S131EC	57	23140	Eco605ORFMP	684	16	100	265	280	9137	9152	0.7	32.2
S131EC	201	437542	Eco84137ORF201P	1635	18	100	43	60	340837	340854	0.11	36.2
S131EC	201	437542	Eco84137ORF201P	1635	16	100	1212	1227	342124	342109	1.7	32.2
S131EC	237	191118	Eco1520ORF67P	1563	18	100	1532	1549	151369	151352	0.1	36.2
S131EC	42	33207	Eco15ORF4165P	1197	33	87.88	237	269	5852	5884	0.31	34.2
S123EC	233	33050	M1.EcoMI	1623	1623	100	1	1623	29725	28103	0	3009
S123EC	233	33050	M1.EcoMI	1191	1191	100	1	1191	28110	26920	0	2361
S123EC	59	65269	M.EcoMII	1620	1581	100	40	1620	44381	45961	0	3092

S123EC	156	23941	M.EcoMIII	1638	1638	100	1	1638	20495	22132	0	3247
S123EC	108	18265	M.EcoMIV	765	765	100	1	765	11283	12047	0	1516
S123EC	309	2105	M.EcoMV	1050	1050	100	1	1050	1965	916	0	2040
S123EC	1	16009	M.EcoNwEVDcm	1419	1419	100	1	1419	1779	361	0	2813
S123EC	219	15710	M.EcoMVI	891	891	100	1	891	3863	4753	0	1725
S123EC	51	33786	M.EcoNwEDam	837	837	100	1	837	14965	15801	0	1659
S123EC	46	13457	M.EcoMVII	3606	25	96	596	620	7252	7228	0.004	42.1
S123EC	249	13725	M.EcoMVIII	684	684	100	1	684	3938	3255	0	1356
S123EC	279	12928	Eco29kI	645	16	100	238	253	1375	1360	0.67	32.2
S123EC	501	1463	EcoDEC4CORF2749P	1041	25	92	108	132	950	926	0.28	34.2
S123EC	2	34844	Eco248534P	1053	16	100	941	956	8830	8815	1.1	32.2
S123EC	2	34844	Eco248534P	1053	15	100	66	80	12720	12706	4.4	30.2
S123EC	252	17730	EcoAPECORF2077P	1590	18	100	1260	1277	4860	4877	0.11	36.2
S123EC	252	17730	EcoAPECORF2077P	1590	17	100	1	17	9222	9206	0.43	34.2
S123EC	476	15883	EcoDEC13EORF3046P	1191	16	100	8	23	14501	14516	1.3	32.2
S123EC	249	13725	Eco7A8ORF29P	684	606	98.51	79	684	3860	3255	0	1130
S123EC	57	3131	EcoDEC2CORF2043P	2019	41	92.68	1723	1763	2052	2092	4e-08	58
S123EC	57	3131	EcoDEC2CORF2043P	2019	15	100	1374	1388	1697	1711	8.4	30.2
S123EC	309	2105	Eco1886ORF14455P	1053	594	82.32	7	600	1959	1366	6e-95	345
S123EC	309	2105	Eco1886ORF14455P	1053	104	81.73	817	920	1149	1046	8e-08	56
S123EC	285	10661	Eco1886ORF14565P	900	15	100	272	286	6021	6035	3.7	30.2
S123EC	249	13725	EcoR7ACORFAP	684	684	97.37	1	684	3938	3255	0	1213
S123EC	249	13725	EcoR100ORF1P	648	606	98.51	43	648	3860	3255	0	1130
S123EC	249	13725	Eco605ORFMP	684	684	93.86	1	684	3938	3255	0	1023
S123EC	132	26220	Eco84137ORF201P	1635	1635	100	1	1635	11320	12954	0	3241
S123EC	132	26220	Eco84137ORF201P	1635	15	100	1102	1116	20848	20862	6.8	30.2
S123EC	132	26220	Eco1520ORF67P	1563	27	92.59	775	801	12130	12156	0.027	38.2
S123EC	132	26220	Eco1520ORF67P	1563	20	95	535	554	11869	11888	1.7	32.2
S123EC	233	33050	Eco15ORF4165P	1197	33	87.88	237	269	27436	27404	0.32	34.2
S126EC	18	34185	M1.EcoMI	1623	1623	100	1	1623	29723	28101	0	3009
S126EC	18	34185	M1.EcoMI	1191	1191	100	1	1191	28108	26918	0	2361
S126EC	65	114196	M.EcoMII	1620	1581	100	40	1620	45680	44100	0	3092
S126EC	6	247758	M.EcoMIII	1638	1638	100	1	1638	73001	71364	0	3247
S126EC	6	247758	M.EcoMIII	1638	15	100	992	1006	68288	68274	6.6	30.2
S126EC	6	247758	M.EcoMIII	1638	15	100	739	753	142794	142780	6.6	30.2
S126EC	6	247758	M.EcoMIII	1638	15	100	1509	1523	243226	243240	6.6	30.2

S126EC	2	89587	M.EcoMIV	765	765	100	1	765	24758	23994	0	1516
S126EC	200	4057	M.EcoMV	1050	1050	100	1	1050	1963	914	0	2040
S126EC	102	36519	M.EcoNwEVDcm	1419	1419	100	1	1419	6400	7818	0	2813
S126EC	61	89470	M.EcoMVI	891	891	100	1	891	79797	80687	0	1725
S126EC	27	112009	M.EcoNwEDam	837	837	100	1	837	39463	38627	0	1659
S126EC	10	245464	M.EcoMVII	3606	25	96	596	620	135423	135447	0.004	42.1
S126EC	10	245464	M.EcoMVII	3606	16	100	201	216	128607	128622	3.7	32.2
S126EC	135	5732	M.EcoMVIII	684	684	100	1	684	3979	3296	0	1356
S126EC	17	240068	Eco29kI	645	16	100	238	253	196664	196649	0.65	32.2
S126EC	32	28147	EcoDEC4CORF2749P	1041	16	100	643	658	15211	15196	1.1	32.2
S126EC	15	126880	Eco248534P	1053	16	100	941	956	36227	36242	1.1	32.2
S126EC	15	126880	Eco248534P	1053	15	100	66	80	32337	32351	4.3	30.2
S126EC	70	66929	EcoAPECORF2077P	1590	18	100	1494	1511	42704	42721	0.1	36.2
S126EC	108	752	EcoDEC13EORF3046P	1191	16	100	920	935	31	16	1.2	32.2
S126EC	135	5732	Eco7A8ORF29P	684	606	98.51	79	684	3901	3296	0	1130
S126EC	79	54017	EcoDEC2CORF2043P	2019	21	95.24	1522	1542	37341	37361	0.53	34.2
S126EC	200	4057	Eco1886ORF14455P	1053	594	82.32	7	600	1957	1364	6e-95	345
S126EC	200	4057	Eco1886ORF14455P	1053	104	81.73	817	920	1147	1044	7e-08	56
S126EC	156	72716	Eco1886ORF14565P	900	15	100	289	303	39972	39986	3.6	30.2
S126EC	156	72716	Eco1886ORF14565P	900	15	100	273	287	59158	59144	3.6	30.2
S126EC	135	5732	EcoR7ACORFAP	684	684	97.37	1	684	3979	3296	0	1213
S126EC	135	5732	EcoR100ORF1P	648	606	98.51	43	648	3901	3296	0	1130
S126EC	135	5732	Eco605ORFMP	684	684	93.86	1	684	3979	3296	0	1023
S126EC	43	48810	Eco84137ORF201P	1635	1635	100	1	1635	47623	45989	0	3241
S126EC	43	48810	Eco84137ORF201P	1635	15	100	1102	1116	38095	38081	6.6	30.2
S126EC	43	48810	Eco1520ORF67P	1563	27	92.59	775	801	46813	46787	0.026	38.2
S126EC	43	48810	Eco1520ORF67P	1563	20	95	535	554	47074	47055	1.6	32.2
S126EC	18	34185	Eco15ORF4165P	1197	33	87.88	237	269	27434	27402	0.31	34.2
S132EC	81	7960	M1.EcoMI	1623	1623	100	1	1623	4635	3013	0	3009
S132EC	81	7960	M1.EcoMI	1191	1191	100	1	1191	3020	1830	0	2361
S132EC	84	115147	M.EcoMII	1620	1581	100	40	1620	44364	42784	0	3092
S132EC	22	288082	M.EcoMIII	1638	1638	100	1	1638	215160	216797	0	3247
S132EC	22	288082	M.EcoMIII	1638	16	100	1336	1351	9063	9048	1.7	32.2
S132EC	22	288082	M.EcoMIII	1638	15	100	1509	1523	44935	44921	6.7	30.2
S132EC	22	288082	M.EcoMIII	1638	15	100	739	753	145367	145381	6.7	30.2
S132EC	22	288082	M.EcoMIII	1638	15	100	992	1006	219873	219887	6.7	30.2

S132EC	76	37692	M.EcoMIV	765	765	100	1	765	26051	26815	0	1516
S132EC	133	7671	M.EcoMV	1050	1050	100	1	1050	6589	7638	0	2040
S132EC	66	21147	M.EcoNwEVDcm	1419	1419	100	1	1419	12372	10954	0	2813
S132EC	1	43805	M.EcoMVI	891	891	100	1	891	2475	1585	0	1725
S132EC	69	84576	M.EcoNwEDam	837	837	100	1	837	45192	46028	0	1659
S132EC	10	263233	M.EcoMVII	3606	25	96	596	620	73143	73119	0.004	42.1
S132EC	10	263233	M.EcoMVII	3606	16	100	201	216	79850	79835	3.7	32.2
S132EC	50	5730	M.EcoMVIII	684	684	100	1	684	1830	2513	0	1356
S132EC	71	64576	Eco29kI	645	16	100	238	253	46037	46052	0.65	32.2
S132EC	22	288082	EcoDEC4CORF2749P	1041	16	100	643	658	25287	25272	1.1	32.2
S132EC	12	102240	Eco248534P	1053	16	100	941	956	50331	50316	1.1	32.2
S132EC	12	102240	Eco248534P	1053	15	100	66	80	54221	54207	4.3	30.2
S132EC	97	173763	EcoAPECORF2077P	1590	18	100	1494	1511	149538	149555	0.1	36.2
S132EC	97	173763	EcoAPECORF2077P	1590	16	100	827	842	63584	63599	1.6	32.2
S132EC	97	173763	EcoAPECORF2077P	1590	15	100	414	428	5328	5314	6.5	30.2
S132EC	99	27150	EcoDEC13EORF3046P	1191	16	100	920	935	46	61	1.2	32.2
S132EC	50	5730	Eco7A8ORF29P	684	606	98.51	79	684	1908	2513	0	1130
S132EC	46	66269	EcoDEC2CORF2043P	2019	21	95.24	1522	1542	35860	35840	0.53	34.2
S132EC	133	7671	Eco1886ORF14455P	1053	594	82.32	7	600	6595	7188	6e-95	345
S132EC	133	7671	Eco1886ORF14455P	1053	104	81.73	817	920	7405	7508	7e-08	56
S132EC	134	131586	Eco1886ORF14565P	900	15	100	884	898	60468	60482	3.6	30.2
S132EC	50	5730	EcoR7ACORFAP	684	684	97.37	1	684	1830	2513	0	1213
S132EC	50	5730	EcoR100ORF1P	648	606	98.51	43	648	1908	2513	0	1130
S132EC	50	5730	Eco605ORFMP	684	684	93.86	1	684	1830	2513	0	1023
S132EC	11	85193	Eco84137ORF201P	1635	1635	100	1	1635	75051	73417	0	3241
S132EC	11	85193	Eco84137ORF201P	1635	15	100	1102	1116	65523	65509	6.6	30.2
S132EC	11	85193	Eco1520ORF67P	1563	27	92.59	775	801	74241	74215	0.026	38.2
S132EC	11	85193	Eco1520ORF67P	1563	20	95	535	554	74502	74483	1.6	32.2
S132EC	81	7960	Eco15ORF4165P	1197	33	87.88	237	269	2346	2314	0.31	34.2
S53EC	45	33008	M1.EcoMI	1623	1623	100	1	1623	3364	4986	0	3009
S53EC	45	33008	M1.EcoMI	1191	1191	100	1	1191	4979	6169	0	2361
S53EC	15	116447	M.EcoMII	1620	1581	100	40	1620	45680	44100	0	3092
S53EC	77	79617	M.EcoMIII	1638	1638	100	1	1638	72881	71244	0	3247
S53EC	77	79617	M.EcoMIII	1638	15	100	992	1006	68168	68154	6.7	30.2
S53EC	12	58124	M.EcoMIV	765	765	100	1	765	39414	38650	0	1516
S53EC	188	1540	M.EcoMV	1050	705	100	346	1050	1	705	0	1356

S53EC	5	18058	M.EcoNwEVDcm	1419	1419	100	1	1419	9283	7865	0	2813
S53EC	56	17935	M.EcoMVI	891	891	100	1	891	11926	11036	0	1725
S53EC	147	19628	M.EcoNwEDam	837	837	100	1	837	18883	18047	0	1659
S53EC	153	30353	M.EcoMVII	3606	3606	100	1	3606	6136	9741	0	6988
S53EC	161	5682	M.EcoMVIII	684	684	100	1	684	1832	2515	0	1356
S53EC	135	17380	Eco29kI	645	16	100	375	390	11477	11492	0.66	32.2
S53EC	177	136586	EcoDEC4CORF2749P	1041	16	100	643	658	59959	59974	1.1	32.2
S53EC	20	47855	Eco248534P	1053	16	100	941	956	1368	1353	1.1	32.2
S53EC	20	47855	Eco248534P	1053	15	100	66	80	5258	5244	4.3	30.2
S53EC	64	110594	EcoAPECORF2077P	1590	18	100	1260	1277	57740	57723	0.11	36.2
S53EC	64	110594	EcoAPECORF2077P	1590	17	100	1	17	53378	53394	0.42	34.2
S53EC	64	110594	EcoAPECORF2077P	1590	17	100	99	115	78730	78746	0.42	34.2
S53EC	64	110594	EcoAPECORF2077P	1590	15	100	669	683	12904	12918	6.5	30.2
S53EC	64	110594	EcoAPECORF2077P	1590	15	100	1025	1039	102795	102809	6.5	30.2
S53EC	260	10368	EcoDEC13EORF3046P	1191	16	100	920	935	48	63	1.2	32.2
S53EC	161	5682	Eco7A8ORF29P	684	606	98.51	79	684	1910	2515	0	1130
S53EC	97	12678	EcoDEC2CORF2043P	2019	21	95.24	1522	1542	59	79	0.53	34.2
S53EC	169	6728	Eco1886ORF14455P	1053	410	84.63	7	416	6397	6806	2e-85	313
S53EC	329	72710	Eco1886ORF14565P	900	15	100	289	303	33442	33456	3.7	30.2
S53EC	329	72710	Eco1886ORF14565P	900	15	100	273	287	52628	52614	3.7	30.2
S53EC	161	5682	EcoR7ACORFAP	684	684	97.37	1	684	1832	2515	0	1213
S53EC	161	5682	EcoR100ORF1P	648	606	98.51	43	648	1910	2515	0	1130
S53EC	161	5682	Eco605ORFMP	684	684	93.86	1	684	1832	2515	0	1023
S53EC	156	73132	Eco84137ORF201P	1635	22	95.45	558	579	21617	21596	0.11	36.2
S53EC	239	3494	Eco1520ORF67P	1563	18	100	1501	1518	3145	3162	0.1	36.2
S53EC	45	33008	Eco15ORF4165P	1197	33	87.88	237	269	5653	5685	0.31	34.2
S39EC	123	33334	M1.EcoMI	1623	1623	100	1	1623	30049	28427	0	3009
S39EC	123	33334	M1.EcoMI	1191	1191	100	1	1191	28434	27244	0	2361
S39EC	90	41790	M.EcoMII	1620	1005	100	616	1620	41868	40864	0	1992
S39EC	138	13050	M.EcoMIII	1638	1638	100	1	1638	9685	11322	0	3247
S39EC	86	63715	M.EcoMIV	765	765	100	1	765	48532	49296	0	1516
S39EC	368	6801	M.EcoMV	1050	591	96.95	1	591	6289	6879	0	1029
S39EC	186	23625	M.EcoNwEVDcm	1419	1419	100	1	1419	8854	10272	0	2813
S39EC	119	29309	M.EcoMVI	891	891	100	1	891	19635	20525	0	1725
S39EC	106	84573	M.EcoNwEDam	837	837	100	1	837	45189	46025	0	1659
S39EC	328	1822	M.EcoMVII	3606	1900	100	1016	2915	1	1900	0	3606

S39EC	287	5732	M.EcoMVIII	684	684	100	1	684	3979	3296	0	1356
S39EC	124	61753	Eco29kI	645	16	100	238	253	40171	40186	0.67	32.2
S39EC	368	6801	EcoDEC4CORF2749P	1041	1041	100	1	1041	2829	3869	0	1974
S39EC	28	53236	Eco248534P	1053	16	100	941	956	1350	1335	1.1	32.2
S39EC	28	53236	Eco248534P	1053	15	100	66	80	5240	5226	4.4	30.2
S39EC	323	2629	EcoAPECORF2077P	1590	18	100	1431	1448	1056	1039	0.11	36.2
S39EC	520	12733	EcoDEC13EORF3046P	1191	16	100	920	935	12764	12749	1.3	32.2
S39EC	287	5732	Eco7A8ORF29P	684	606	98.51	79	684	3901	3296	0	1130
S39EC	103	38803	EcoDEC2CORF2043P	2019	21	95.24	1522	1542	35237	35257	0.54	34.2
S39EC	368	6801	Eco1886ORF14455P	1053	585	82.05	7	591	6295	6879	1e-89	327
S39EC	505	36404	Eco1886ORF14565P	900	15	100	289	303	21316	21330	3.7	30.2
S39EC	287	5732	EcoR7ACORFAP	684	684	97.37	1	684	3979	3296	0	1213
S39EC	287	5732	EcoR100ORF1P	648	606	98.51	43	648	3901	3296	0	1130
S39EC	353	35209	Eco605ORFMP	684	684	100	1	684	31400	32083	0	1356
S39EC	88	17524	Eco84137ORF201P	1635	18	100	43	60	8642	8659	0.11	36.2
S39EC	88	17524	Eco84137ORF201P	1635	16	100	1212	1227	9929	9914	1.7	32.2
S39EC	350	71029	Eco1520ORF67P	1563	18	100	1501	1518	24587	24604	0.11	36.2
S39EC	123	33334	Eco15ORF4165P	1197	33	87.88	237	269	27760	27728	0.32	34.2
HVM834	635	33004	M1.EcoMI	1623	1623	100	1	1623	29719	28097	0	3009
HVM834	635	33004	M1.EcoMI	1191	1191	100	1	1191	28104	26914	0	2361
HVM834	43	88570	M.EcoMII	1620	1581	100	40	1620	42970	44550	0	3092
HVM834	54	106462	M.EcoMIII	1638	1638	100	1	1638	72877	71240	0	3247
HVM834	54	106462	M.EcoMIII	1638	15	100	992	1006	68164	68150	7	30.2
HVM834	42	30049	M.EcoMIV	765	765	100	1	765	9984	10748	0	1516
HVM834	276	2223	M.EcoMV	1050	1050	100	1	1050	1141	2190	0	2040
HVM834	118	19242	M.EcoNwEVDcm	1419	1419	100	1	1419	8850	10268	0	2813
HVM834	21	91639	M.EcoMVI	891	891	100	1	891	11922	11032	0	1725
HVM834	30	105658	M.EcoNwEDam	837	837	100	1	837	39459	38623	0	1659
HVM834	297	17249	M.EcoMVII	3606	3606	100	1	3606	11192	7587	0	6988
HVM834	257	782	M.EcoMVIII	684	684	100	1	684	743	60	0	1356
HVM834	357	104718	Eco29kI	645	16	100	238	253	46327	46342	0.69	32.2
HVM834	119	39323	EcoDEC4CORF2749P	1041	16	100	654	669	30876	30891	1.1	32.2
HVM834	56	163072	Eco248534P	1053	16	100	941	956	111182	111167	1.1	32.2
HVM834	56	163072	Eco248534P	1053	15	100	66	80	115072	115058	4.5	30.2
HVM834	43	88570	EcoAPECORF2077P	1590	19	100	312	330	10125	10143	0.028	38.2
HVM834	43	88570	EcoAPECORF2077P	1590	15	100	724	738	6920	6906	6.8	30.2

HVM834	43	88570	EcoAPECORF2077P	1590	15	100	265	279	47664	47650	6.8	30.2
HVM834	516	756	EcoDEC13EORF3046P	1191	16	100	920	935	31	16	1.3	32.2
HVM834	257	782	Eco7A8ORF29P	684	606	98.51	79	684	665	60	0	1130
HVM834	130	17550	EcoDEC2CORF2043P	2019	21	95.24	1522	1542	17555	17575	0.56	34.2
HVM834	43	88570	Eco1886ORF14455P	1053	1053	99.81	1	1053	4890	3838	0	2030
HVM834	441	83674	Eco1886ORF14565P	900	15	100	884	898	22339	22325	3.8	30.2
HVM834	257	782	EcoR7ACORFAP	684	684	97.37	1	684	743	60	0	1213
HVM834	257	782	EcoR100ORF1P	648	606	98.51	43	648	665	60	0	1130
HVM834	257	782	Eco605ORFMP	684	684	93.86	1	684	743	60	0	1023
HVM834	86	38313	Eco84137ORF201P	1635	18	100	43	60	28965	28982	0.11	36.2
HVM834	86	38313	Eco84137ORF201P	1635	16	100	1212	1227	30252	30237	1.8	32.2
HVM834	266	44470	Eco1520ORF67P	1563	18	100	1501	1518	19452	19469	0.11	36.2
HVM834	635	33004	Eco15ORF4165P	1197	33	87.88	237	269	27430	27398	0.33	34.2
HVM1997	245	32990	M1.EcoMI	1623	1623	100	1	1623	3346	4968	0	3009
HVM1997	245	32990	M1.EcoMI	1191	1191	100	1	1191	4961	6151	0	2361
HVM1997	126	74055	M.EcoMII	1620	1581	100	40	1620	45662	44082	0	3092
HVM1997	11	310764	M.EcoMIII	1638	1638	100	1	1638	52832	51195	0	3247
HVM1997	11	310764	M.EcoMIII	1638	16	100	1336	1351	255882	255897	1.7	32.2
HVM1997	11	310764	M.EcoMIII	1638	15	100	992	1006	48119	48105	6.7	30.2
HVM1997	11	310764	M.EcoMIII	1638	15	100	739	753	122625	122611	6.7	30.2
HVM1997	11	310764	M.EcoMIII	1638	15	100	1509	1523	223059	223073	6.7	30.2
HVM1997	100	4748	M.EcoMIV	765	765	100	1	765	1569	2333	0	1516
HVM1997	231	4119	M.EcoMV	1050	1050	100	1	1050	1143	94	0	2040
HVM1997	60	137797	M.EcoNwEVDcm	1419	1419	100	1	1419	22380	20962	0	2813
HVM1997	21	158995	M.EcoMVI	891	891	100	1	891	11908	11018	0	1725
HVM1997	29	190868	M.EcoNwEDam	837	837	100	1	837	39445	38609	0	1659
HVM1997	164	17233	M.EcoMVII	3606	3606	100	1	3606	6118	9723	0	6988
HVM1997	192	5714	M.EcoMVIII	684	684	100	1	684	1814	2497	0	1356
HVM1997	60	137797	Eco29kI	645	16	100	375	390	118515	118500	0.66	32.2
HVM1997	60	137797	Eco29kI	645	15	100	494	508	42325	42339	2.6	30.2
HVM1997	165	347956	EcoDEC4CORF2749P	1041	16	100	654	669	215189	215174	1.1	32.2
HVM1997	15	269240	Eco248534P	1053	16	100	941	956	51984	51999	1.1	32.2
HVM1997	15	269240	Eco248534P	1053	15	100	66	80	48094	48108	4.3	30.2
HVM1997	15	269240	Eco248534P	1053	15	100	972	986	177817	177803	4.3	30.2
HVM1997	31	172872	EcoAPECORF2077P	1590	18	100	1494	1511	24285	24268	0.11	36.2
HVM1997	31	172872	EcoAPECORF2077P	1590	16	100	827	842	110239	110224	1.6	32.2

HVM1997	31	172872	EcoAPECORF2077P	1590	15	100	414	428	168382	168396	6.5	30.2
HVM1997	60	137797	EcoDEC13EORF3046P	1191	16	100	920	935	32558	32573	1.2	32.2
HVM1997	192	5714	Eco7A8ORF29P	684	606	98.51	79	684	1892	2497	0	1130
HVM1997	165	347956	EcoDEC2CORF2043P	2019	21	95.24	1522	1542	35844	35824	0.53	34.2
HVM1997	165	347956	EcoDEC2CORF2043P	2019	19	94.74	1813	1831	339667	339685	8.3	30.2
HVM1997	231	4119	Eco1886ORF14455P	1053	594	82.32	7	600	1137	544	6e-95	345
HVM1997	231	4119	Eco1886ORF14455P	1053	104	81.73	817	920	327	224	7e-08	56
HVM1997	364	91279	Eco1886ORF14565P	900	15	100	289	303	39933	39947	3.7	30.2
HVM1997	364	91279	Eco1886ORF14565P	900	15	100	273	287	59117	59103	3.7	30.2
HVM1997	192	5714	EcoR7ACORFAP	684	684	97.37	1	684	1814	2497	0	1213
HVM1997	192	5714	EcoR100ORF1P	648	606	98.51	43	648	1892	2497	0	1130
HVM1997	192	5714	Eco605ORFMP	684	684	93.86	1	684	1814	2497	0	1023
HVM1997	165	347956	Eco84137ORF201P	1635	18	100	43	60	274859	274876	0.11	36.2
HVM1997	165	347956	Eco84137ORF201P	1635	16	100	751	766	5617	5602	1.7	32.2
HVM1997	165	347956	Eco84137ORF201P	1635	16	100	1212	1227	276146	276131	1.7	32.2
HVM1997	41	145729	Eco1520ORF67P	1563	18	100	1501	1518	124880	124897	0.1	36.2
HVM1997	41	145729	Eco1520ORF67P	1563	15	100	1239	1253	92322	92308	6.4	30.2
HVM1997	245	32990	Eco15ORF4165P	1197	33	87.88	237	269	5635	5667	0.31	34.2
HVM1619	41	33008	M1.EcoMI	1623	1623	100	1	1623	29723	28101	0	3009
HVM1619	41	33008	M1.EcoMI	1191	1191	100	1	1191	28108	26918	0	2361
HVM1619	78	116492	M.EcoMII	1620	1581	100	40	1620	70891	72471	0	3092
HVM1619	68	67197	M.EcoMIII	1638	1638	100	1	1638	54382	52745	0	3247
HVM1619	68	67197	M.EcoMIII	1638	15	100	992	1006	49669	49655	6.8	30.2
HVM1619	118	15688	M.EcoMIV	765	765	100	1	765	7592	6828	0	1516
HVM1619	517	936	M.EcoMV	1050	903	100	148	1050	1014	112	0	1748
HVM1619	268	10041	M.EcoNwEVDcm	1419	1266	100	1	1266	1266	1	0	2510
HVM1619	87	91642	M.EcoMVI	891	891	100	1	891	11926	11036	0	1725
HVM1619	103	16849	M.EcoNwEDam	837	837	100	1	837	15013	15849	0	1659
HVM1619	329	17254	M.EcoMVII	3606	3606	100	1	3606	6136	9741	0	6988
HVM1619	73	5690	M.EcoMVIII	684	684	100	1	684	3937	3254	0	1356
HVM1619	365	4860	Eco29kI	645	16	100	375	390	1775	1760	0.67	32.2
HVM1619	293	12857	EcoDEC4CORF2749P	1041	16	100	654	669	6304	6319	1.1	32.2
HVM1619	189	20096	Eco248534P	1053	16	100	941	956	10867	10852	1.1	32.2
HVM1619	189	20096	Eco248534P	1053	15	100	66	80	14757	14743	4.3	30.2
HVM1619	298	6469	EcoAPECORF2077P	1590	18	100	1511	1528	37	20	0.11	36.2
HVM1619	447	752	EcoDEC13EORF3046P	1191	16	100	920	935	31	16	1.2	32.2

HVM1619	73	5690	Eco7A8ORF29P	684	606	98.51	79	684	3859	3254	0	1130
HVM1619	177	20682	EcoDEC2CORF2043P	2019	21	95.24	1522	1542	19769	19749	0.54	34.2
HVM1619	517	936	Eco1886ORF14455P	1053	453	83.66	148	600	1014	562	8e-85	311
HVM1619	517	936	Eco1886ORF14455P	1053	104	81.73	817	920	345	242	8e-08	56
HVM1619	232	11509	Eco1886ORF14565P	900	15	100	565	579	4095	4109	3.7	30.2
HVM1619	73	5690	EcoR7ACORFAP	684	684	97.37	1	684	3937	3254	0	1213
HVM1619	73	5690	EcoR100ORF1P	648	606	98.51	43	648	3859	3254	0	1130
HVM1619	73	5690	Eco605ORFMP	684	684	93.86	1	684	3937	3254	0	1023
HVM1619	164	2973	Eco84137ORF201P	1635	22	95.45	558	579	1194	1173	0.11	36.2
HVM1619	534	1628	Eco1520ORF67P	1563	18	100	1501	1518	1264	1281	0.1	36.2
HVM1619	41	33008	Eco15ORF4165P	1197	33	87.88	237	269	27434	27402	0.32	34.2
S43EC	33	33010	M1.EcoMI	1623	1623	100	1	1623	29725	28103	0	3009
S43EC	33	33010	M1.EcoMI	1191	1191	100	1	1191	28110	26920	0	2361
S43EC	25	61132	M.EcoMII	1620	1581	100	40	1620	15531	17111	0	3092
S43EC	30	45896	M.EcoMIII	1638	1638	100	1	1638	33762	32125	0	3247
S43EC	30	45896	M.EcoMIII	1638	15	100	992	1006	29049	29035	6.6	30.2
S43EC	34	30944	M.EcoMIV	765	765	100	1	765	10027	10791	0	1516
S43EC	99	4575	M.EcoMV	1050	1050	100	1	1050	2691	3740	0	2040
S43EC	100	38983	M.EcoNwEVDcm	1419	1419	100	1	1419	30208	28790	0	2813
S43EC	37	88068	M.EcoMVI	891	891	100	1	891	11928	11038	0	1725
S43EC	23	171393	M.EcoNwEDam	837	837	100	1	837	39465	38629	0	1659
S43EC	60	27171	M.EcoMVII	3606	3606	100	1	3606	6138	9743	0	6988
S43EC	63	7597	M.EcoMVIII	684	684	89.91	1	684	7465	6782	0	809
S43EC	213	104293	Eco29kI	645	16	100	238	253	58333	58318	0.65	32.2
S43EC	98	11646	EcoDEC4CORF2749P	1041	16	100	84	99	8322	8307	1.1	32.2
S43EC	66	48251	Eco248534P	1053	16	100	941	956	27406	27421	1.1	32.2
S43EC	66	48251	Eco248534P	1053	15	100	66	80	23516	23530	4.2	30.2
S43EC	166	109363	EcoAPECORF2077P	1590	18	100	608	625	58220	58203	0.1	36.2
S43EC	166	109363	EcoAPECORF2077P	1590	17	100	1034	1050	64727	64711	0.41	34.2
S43EC	166	109363	EcoAPECORF2077P	1590	15	100	1543	1557	78573	78559	6.4	30.2
S43EC	166	109363	EcoAPECORF2077P	1590	15	100	668	682	84556	84542	6.4	30.2
S43EC	122	3923	EcoDEC13EORF3046P	1191	16	100	920	935	50	65	1.2	32.2
S43EC	63	7597	Eco7A8ORF29P	684	621	88.89	64	684	7402	6782	0	684
S43EC	27	96512	EcoDEC2CORF2043P	2019	21	95.24	1522	1542	80637	80657	0.52	34.2
S43EC	99	4575	Eco1886ORF14455P	1053	594	82.32	7	600	2697	3290	6e-95	345
S43EC	99	4575	Eco1886ORF14455P	1053	104	81.73	817	920	3507	3610	7e-08	56

S43EC	86	13278	Eco1886ORF14565P	900	15	100	74	88	4111	4125	3.6	30.2
S43EC	63	7597	EcoR7ACORFAP	684	684	89.47	1	684	7465	6782	0	785
S43EC	63	7597	EcoR100ORF1P	648	621	88.89	28	648	7402	6782	0	684
S43EC	63	7597	Eco605ORFMP	684	684	89.62	1	684	7465	6782	0	793
S43EC	21	171761	Eco84137ORF201P	1635	18	100	43	60	13301	13284	0.11	36.2
S43EC	21	171761	Eco84137ORF201P	1635	16	100	1212	1227	12014	12029	1.7	32.2
S43EC	53	118799	Eco1520ORF67P	1563	18	100	1501	1518	7573	7556	0.1	36.2
S43EC	53	118799	Eco1520ORF67P	1563	15	100	1239	1253	40131	40145	6.3	30.2
S43EC	33	33010	Eco15ORF4165P	1197	33	87.88	237	269	27436	27404	0.31	34.2
S127EC	214	6590	M1.EcoMI	1623	1623	100	1	1623	3305	1683	0	3009
S127EC	214	6590	M1.EcoMI	1191	1191	100	1	1191	1690	500	0	2361
S127EC	1	74053	M.EcoMII	1620	1581	100	40	1620	28452	30032	0	3092
S127EC	14	330794	M.EcoMIII	1638	1638	100	1	1638	257992	259629	0	3247
S127EC	14	330794	M.EcoMIII	1638	16	100	1336	1351	54941	54926	1.7	32.2
S127EC	14	330794	M.EcoMIII	1638	15	100	1509	1523	87764	87750	6.6	30.2
S127EC	14	330794	M.EcoMIII	1638	15	100	739	753	188198	188212	6.6	30.2
S127EC	14	330794	M.EcoMIII	1638	15	100	992	1006	262705	262719	6.6	30.2
S127EC	34	301241	M.EcoMIV	765	765	100	1	765	235970	235206	0	1516
S127EC	6	186941	M.EcoMV	1050	1050	100	1	1050	4081	3032	0	2040
S127EC	6	186941	M.EcoMV	1050	16	100	115	130	104901	104916	1.1	32.2
S127EC	86	64896	M.EcoNwEVDcm	1419	1419	100	1	1419	56121	54703	0	2813
S127EC	18	91643	M.EcoMVI	891	891	100	1	891	11906	11016	0	1725
S127EC	32	190866	M.EcoNwEDam	837	837	100	1	837	39443	38607	0	1659
S127EC	10	378677	M.EcoMVII	3606	25	96	596	620	255211	255235	0.004	42.1
S127EC	10	378677	M.EcoMVII	3606	16	100	201	216	248395	248410	3.7	32.2
S127EC	49	8996	M.EcoMVIII	684	684	100	1	684	1812	2495	0	1356
S127EC	86	64896	Eco29kI	645	16	100	375	390	14459	14444	0.64	32.2
S127EC	14	330794	EcoDEC4CORF2749P	1041	16	100	643	658	68116	68101	1	32.2
S127EC	11	282256	Eco248534P	1053	16	100	941	956	51982	51997	1.1	32.2
S127EC	11	282256	Eco248534P	1053	15	100	66	80	48092	48106	4.2	30.2
S127EC	11	282256	Eco248534P	1053	15	100	972	986	177815	177801	4.2	30.2
S127EC	145	173629	EcoAPECORF2077P	1590	18	100	1494	1511	24283	24266	0.1	36.2
S127EC	145	173629	EcoAPECORF2077P	1590	16	100	827	842	110237	110222	1.6	32.2
S127EC	145	173629	EcoAPECORF2077P	1590	15	100	414	428	168380	168394	6.4	30.2
S127EC	148	68168	EcoDEC13EORF3046P	1191	16	100	920	935	28	43	1.2	32.2
S127EC	49	8996	Eco7A8ORF29P	684	606	98.51	79	684	1890	2495	0	1130

S127EC	11	282256	EcoDEC2CORF2043P	2019	21	95.24	1522	1542	236635	236615	0.52	34.2
S127EC	6	186941	Eco1886ORF14455P	1053	594	82.32	7	600	4075	3482	5e-95	345
S127EC	6	186941	Eco1886ORF14455P	1053	104	81.73	817	920	3265	3162	7e-08	56
S127EC	148	68168	Eco1886ORF14565P	900	15	100	50	64	61595	61581	3.6	30.2
S127EC	49	8996	EcoR7ACORFAP	684	684	97.37	1	684	1812	2495	0	1213
S127EC	49	8996	EcoR100ORF1P	648	606	98.51	43	648	1890	2495	0	1130
S127EC	49	8996	Eco605ORFMP	684	684	93.86	1	684	1812	2495	0	1023
S127EC	148	68168	Eco84137ORF201P	1635	22	95.45	558	579	16653	16632	0.11	36.2
S127EC	9	191066	Eco1520ORF67P	1563	18	100	1532	1549	39735	39752	0.1	36.2
S127EC	214	6590	Eco15ORF4165P	1197	33	87.88	237	269	1016	984	0.31	34.2
S47EC	73	21704	M1.EcoMI	1623	1623	100	1	1623	18419	16797	0	3009
S47EC	73	21704	M1.EcoMI	1191	1191	100	1	1191	16804	15614	0	2361
S47EC	24	51837	M.EcoMII	1620	1581	100	40	1620	13034	11454	0	3092
S47EC	23	44679	M.EcoMIII	1638	1638	100	1	1638	12716	14353	0	3247
S47EC	23	44679	M.EcoMIII	1638	15	100	992	1006	17429	17443	6.7	30.2
S47EC	77	84077	M.EcoMIV	765	765	100	1	765	22246	23010	0	1516
S47EC	30	1821	M.EcoMV	1050	986	100	65	1050	1	986	0	1913
S47EC	323	3798	M.EcoNwEVDcm	1419	1419	100	1	1419	1548	2966	0	2813
S47EC	66	38435	M.EcoMVI	891	891	100	1	891	26588	27478	0	1725
S47EC	27	43305	M.EcoNwEDam	837	837	100	1	837	11668	12504	0	1659
S47EC	43	42266	M.EcoMVII	3606	3606	100	1	3606	6136	9741	0	6988
S47EC	72	5685	M.EcoMVIII	684	684	100	1	684	1832	2515	0	1356
S47EC	197	17676	Eco29kI	645	16	100	375	390	7675	7690	0.66	32.2
S47EC	25	34546	EcoDEC4CORF2749P	1041	16	100	654	669	10435	10450	1.1	32.2
S47EC	100	47962	Eco248534P	1053	16	100	941	956	26618	26603	1.1	32.2
S47EC	100	47962	Eco248534P	1053	15	100	66	80	30508	30494	4.3	30.2
S47EC	137	16737	EcoAPECORF2077P	1590	18	100	608	625	1682	1699	0.11	36.2
S47EC	284	752	EcoDEC13EORF3046P	1191	16	100	920	935	31	16	1.2	32.2
S47EC	72	5685	Eco7A8ORF29P	684	606	98.51	79	684	1910	2515	0	1130
S47EC	230	3064	EcoDEC2CORF2043P	2019	21	95.24	1522	1542	3038	3058	0.53	34.2
S47EC	30	1821	Eco1886ORF14455P	1053	515	83.3	86	600	22	536	3e-93	339
S47EC	30	1821	Eco1886ORF14455P	1053	104	81.73	817	920	753	856	7e-08	56
S47EC	272	7736	Eco1886ORF14565P	900	15	100	30	44	989	1003	3.7	30.2
S47EC	72	5685	EcoR7ACORFAP	684	684	97.37	1	684	1832	2515	0	1213
S47EC	72	5685	EcoR100ORF1P	648	606	98.51	43	648	1910	2515	0	1130
S47EC	72	5685	Eco605ORFMP	684	684	93.86	1	684	1832	2515	0	1023

S47EC	248	6541	Eco84137ORF201P	1635	22	95.45	558	579	1164	1143	0.11	36.2
S47EC	431	32233	Eco1520ORF67P	1563	18	100	1501	1518	11087	11104	0.1	36.2
S47EC	73	21704	Eco15ORF4165P	1197	33	87.88	237	269	16130	16098	0.31	34.2
S30EC	106	9880	M1.EcoMI	1623	1623	100	1	1623	6595	4973	0	3009
S30EC	106	9880	M1.EcoMI	1191	1191	100	1	1191	4980	3790	0	2361
S30EC	39	62177	M.EcoMII	1620	1581	100	40	1620	5797	4217	0	3092
S30EC	63	52226	M.EcoMIII	1638	1638	100	1	1638	7835	9472	0	3247
S30EC	63	52226	M.EcoMIII	1638	15	100	992	1006	12548	12562	6.7	30.2
S30EC	32	53370	M.EcoMIV	765	765	100	1	765	8223	8987	0	1516
S30EC	253	8477	M.EcoMV	1050	1050	100	1	1050	1965	916	0	2040
S30EC	48	11100	M.EcoNwEVDcm	1419	1419	100	1	1419	2325	907	0	2813
S30EC	127	31343	M.EcoMVI	891	891	100	1	891	19496	20386	0	1725
S30EC	143	43244	M.EcoNwEDam	837	837	100	1	837	32764	33600	0	1659
S30EC	114	41961	M.EcoMVII	3606	3606	100	1	3606	35904	32299	0	6988
S30EC	120	5686	M.EcoMVIII	684	684	100	1	684	3933	3250	0	1356
S30EC	75	35248	Eco29kI	645	16	100	375	390	3164	3179	0.66	32.2
S30EC	141	13843	EcoDEC4CORF2749P	1041	16	100	654	669	9815	9800	1.1	32.2
S30EC	8	88990	Eco248534P	1053	16	100	941	956	25236	25251	1.1	32.2
S30EC	8	88990	Eco248534P	1053	15	100	66	80	21346	21360	4.3	30.2
S30EC	123	69076	EcoAPECORF2077P	1590	18	100	1511	1528	34701	34718	0.11	36.2
S30EC	123	69076	EcoAPECORF2077P	1590	19	94.74	1493	1511	8123	8141	6.5	30.2
S30EC	312	17573	EcoDEC13EORF3046P	1191	16	100	920	935	17604	17589	1.2	32.2
S30EC	120	5686	Eco7A8ORF29P	684	606	98.51	79	684	3855	3250	0	1130
S30EC	131	47212	EcoDEC2CORF2043P	2019	21	95.24	1522	1542	17593	17613	0.53	34.2
S30EC	253	8477	Eco1886ORF14455P	1053	594	82.32	7	600	1959	1366	6e-95	345
S30EC	253	8477	Eco1886ORF14455P	1053	104	81.73	817	920	1149	1046	7e-08	56
S30EC	257	5197	Eco1886ORF14565P	900	15	100	273	287	4134	4120	3.6	30.2
S30EC	120	5686	EcoR7ACORFAP	684	684	97.37	1	684	3933	3250	0	1213
S30EC	120	5686	EcoR100ORF1P	648	606	98.51	43	648	3855	3250	0	1130
S30EC	120	5686	Eco605ORFMP	684	684	93.86	1	684	3933	3250	0	1023
S30EC	167	8724	Eco84137ORF201P	1635	22	95.45	558	579	8410	8389	0.11	36.2
S30EC	421	52579	Eco1520ORF67P	1563	18	100	1532	1549	2356	2373	0.1	36.2
S30EC	106	9880	Eco15ORF4165P	1197	33	87.88	237	269	4306	4274	0.31	34.2
S101EC	267	11704	M1.EcoMI	1623	1623	100	1	1623	3366	4988	0	3009
S101EC	267	11704	M1.EcoMI	1191	1191	100	1	1191	4981	6171	0	2361
S101EC	367	21779	M.EcoMII	1620	843	100	40	882	843	1	0	1629

S101EC	289	11940	M.EcoMIII	1638	1638	100	1	1638	3759	2122	0	3247
S101EC	191	28705	M.EcoMIV	765	765	100	1	765	18745	17981	0	1516
S101EC	258	1796	M.EcoMV	1050	946	100	105	1050	16	961	0	1834
S101EC	106	20313	M.EcoNwEVDcm	1419	1419	100	1	1419	8856	10274	0	2813
S101EC	58	41631	M.EcoMVI	891	891	100	1	891	11928	11038	0	1725
S101EC	165	63973	M.EcoNwEDam	837	837	100	1	837	52878	53714	0	1659
S101EC	333	22353	M.EcoMVII	3606	3606	100	1	3606	6138	9743	0	6988
S101EC	99	5690	M.EcoMVIII	684	684	100	1	684	1834	2517	0	1356
S101EC	247	35410	Eco29kI	645	16	100	375	390	23999	23984	0.66	32.2
S101EC	143	29591	EcoDEC4CORF2749P	1041	16	100	654	669	21154	21169	1.1	32.2
S101EC	149	17262	Eco248534P	1053	16	100	941	956	3323	3338	1.1	32.2
S101EC	254	10268	EcoAPECORF2077P	1590	18	100	1511	1528	6476	6493	0.1	36.2
S101EC	411	750	EcoDEC13EORF3046P	1191	16	100	920	935	800	815	1.2	32.2
S101EC	99	5690	Eco7A8ORF29P	684	606	98.51	79	684	1912	2517	0	1130
S101EC	164	49128	EcoDEC2CORF2043P	2019	21	95.24	1522	1542	35864	35844	0.53	34.2
S101EC	258	1796	Eco1886ORF14455P	1053	495	83.43	106	600	17	511	8e-91	331
S101EC	258	1796	Eco1886ORF14455P	1053	104	81.73	817	920	728	831	7e-08	56
S101EC	218	10284	Eco1886ORF14565P	900	15	100	30	44	6828	6814	3.6	30.2
S101EC	99	5690	EcoR7ACORFAP	684	684	97.37	1	684	1834	2517	0	1213
S101EC	99	5690	EcoR100ORF1P	648	606	98.51	43	648	1912	2517	0	1130
S101EC	99	5690	Eco605ORFMP	684	684	93.86	1	684	1834	2517	0	1023
S101EC	283	7361	Eco84137ORF201P	1635	18	100	43	60	2076	2059	0.11	36.2
S101EC	283	7361	Eco84137ORF201P	1635	16	100	1212	1227	789	804	1.7	32.2
S101EC	274	34442	Eco1520ORF67P	1563	18	100	1501	1518	10251	10234	0.1	36.2
S101EC	267	11704	Eco15ORF4165P	1197	33	87.88	237	269	5655	5687	0.31	34.2
S134EC	69	32313	M1.EcoMI	1623	1623	100	1	1623	2669	4291	0	3009
S134EC	69	32313	M1.EcoMI	1191	1191	100	1	1191	4284	5474	0	2361
S134EC	155	11458	M.EcoMII	1620	1581	100	40	1620	6554	4974	0	3092
S134EC	268	12765	M.EcoMIII	1638	1638	100	1	1638	9702	11339	0	3247
S134EC	111	32264	M.EcoMIV	765	765	100	1	765	24756	23992	0	1516
S134EC	184	7657	M.EcoMV	1050	1050	100	1	1050	1965	916	0	2040
S134EC	287	20339	M.EcoNwEVDcm	1419	1419	100	1	1419	11564	10146	0	2813
S134EC	277	33316	M.EcoMVI	891	891	100	1	891	3519	2629	0	1725
S134EC	30	19566	M.EcoNwEDam	837	837	100	1	837	15045	14209	0	1659
S134EC	150	27276	M.EcoMVII	3606	3428	100	179	3606	27356	23929	0	6635
S134EC	207	5734	M.EcoMVIII	684	684	100	1	684	3981	3298	0	1356

S134EC	169	28508	Eco29kI	645	16	100	238	253	8427	8412	0.67	32.2
S134EC	114	11939	EcoDEC4CORF2749P	1041	16	100	654	669	8508	8493	1.1	32.2
S134EC	163	24613	Eco248534P	1053	16	100	971	986	2145	2130	1.1	32.2
S134EC	162	35651	EcoAPECORF2077P	1590	18	100	1511	1528	6497	6514	0.11	36.2
S134EC	471	750	EcoDEC13EORF3046P	1191	16	100	920	935	31	16	1.3	32.2
S134EC	207	5734	Eco7A8ORF29P	684	606	98.51	79	684	3903	3298	0	1130
S134EC	211	43659	EcoDEC2CORF2043P	2019	2019	97.23	1	2019	40916	38898	0	3558
S134EC	211	43659	EcoDEC2CORF2043P	2019	24	91.67	139	162	40755	40778	2.1	32.2
S134EC	184	7657	Eco1886ORF14455P	1053	594	82.32	7	600	1959	1366	6e-95	345
S134EC	184	7657	Eco1886ORF14455P	1053	104	81.73	817	920	1149	1046	8e-08	56
S134EC	163	24613	Eco1886ORF14565P	900	16	100	519	534	3218	3203	0.94	32.2
S134EC	207	5734	EcoR7ACORFAP	684	684	97.37	1	684	3981	3298	0	1213
S134EC	207	5734	EcoR100ORF1P	648	606	98.51	43	648	3903	3298	0	1130
S134EC	207	5734	Eco605ORFMP	684	684	93.86	1	684	3981	3298	0	1023
S134EC	352	11379	Eco84137ORF201P	1635	18	100	43	60	8374	8391	0.11	36.2
S134EC	352	11379	Eco84137ORF201P	1635	16	100	1212	1227	9661	9646	1.7	32.2
S134EC	331	34320	Eco1520ORF67P	1563	18	100	1501	1518	8941	8924	0.11	36.2
S134EC	69	32313	Eco15ORF4165P	1197	33	87.88	237	269	4958	4990	0.32	34.2
B36EC	315	19736	M1.EcoMI	1623	1623	100	1	1623	16451	14829	0	3009
B36EC	315	19736	M1.EcoMI	1191	1191	100	1	1191	14836	13646	0	2361
B36EC	34	20545	M.EcoMII	1620	1581	100	40	1620	7043	8623	0	3092
B36EC	507	4203	M.EcoMIII	1638	1202	100	1	1202	3072	4273	0	2383
B36EC	980	5315	M.EcoMIV	765	765	100	1	765	3680	2916	0	1516
B36EC	932	4151	M.EcoMV	1050	1050	100	1	1050	2267	3316	0	2040
B36EC	657	4435	M.EcoNwEVDcm	1419	1419	100	1	1419	2025	607	0	2813
B36EC	55	14433	M.EcoMVI	891	891	100	1	891	5486	6376	0	1725
B36EC	199	11488	M.EcoNwEDam	837	837	100	1	837	1828	2664	0	1659
B36EC	239	8961	M.EcoMVII	3606	2904	100	1	2904	2904	1	0	5596
B36EC	684	5685	M.EcoMVIII	684	684	100	1	684	3932	3249	0	1356
B36EC	683	3685	Eco29kI	645	16	100	375	390	104	89	0.66	32.2
B36EC	398	9616	EcoDEC4CORF2749P	1041	16	100	654	669	9446	9431	1.1	32.2
B36EC	2	16736	Eco248534P	1053	16	100	941	956	4918	4903	1.1	32.2
B36EC	2	16736	Eco248534P	1053	15	100	66	80	8808	8794	4.3	30.2
B36EC	382	23883	EcoAPECORF2077P	1590	18	100	1260	1277	439	456	0.11	36.2
B36EC	382	23883	EcoAPECORF2077P	1590	17	100	1	17	4801	4785	0.42	34.2
B36EC	685	750	EcoDEC13EORF3046P	1191	16	100	920	935	31	16	1.2	32.2

B36EC	684	5685	Eco7A8ORF29P	684	606	98.51	79	684	3854	3249	0	1130
B36EC	389	3328	EcoDEC2CORF2043P	2019	21	95.24	1522	1542	69	49	0.53	34.2
B36EC	932	4151	Eco1886ORF14455P	1053	594	82.32	7	600	2273	2866	6e-95	345
B36EC	932	4151	Eco1886ORF14455P	1053	104	81.73	817	920	3083	3186	7e-08	56
B36EC	1025	165	Eco1886ORF14565P	900	15	100	30	44	140	154	3.7	30.2
B36EC	684	5685	EcoR7ACORFAP	684	684	97.37	1	684	3932	3249	0	1213
B36EC	684	5685	EcoR100ORF1P	648	606	98.51	43	648	3854	3249	0	1130
B36EC	684	5685	Eco605ORFMP	684	684	93.86	1	684	3932	3249	0	1023
B36EC	359	3302	Eco84137ORF201P	1635	22	95.45	558	579	2177	2156	0.11	36.2
B36EC	729	1204	Eco1520ORF67P	1563	18	100	1501	1518	451	434	0.1	36.2
B36EC	315	19736	Eco15ORF4165P	1197	33	87.88	237	269	14162	14130	0.31	34.2
S10EC	69	33006	M1.EcoMI	1623	1623	100	1	1623	3362	4984	0	3009
S10EC	69	33006	M1.EcoMI	1191	1191	100	1	1191	4977	6167	0	2361
S10EC	32	104890	M.EcoMII	1620	1581	100	40	1620	59289	60869	0	3092
S10EC	153	330735	M.EcoMIII	1638	1638	100	1	1638	72879	71242	0	3247
S10EC	153	330735	M.EcoMIII	1638	16	100	1336	1351	275929	275944	1.7	32.2
S10EC	153	330735	M.EcoMIII	1638	15	100	992	1006	68166	68152	6.6	30.2
S10EC	153	330735	M.EcoMIII	1638	15	100	739	753	142672	142658	6.6	30.2
S10EC	153	330735	M.EcoMIII	1638	15	100	1509	1523	243106	243120	6.6	30.2
S10EC	50	18240	M.EcoMIV	765	765	100	1	765	6985	6221	0	1516
S10EC	188	344565	M.EcoMV	1050	18	100	976	993	131929	131912	0.068	36.2
S10EC	188	344565	M.EcoMV	1050	16	100	781	796	198872	198887	1.1	32.2
S10EC	61	38976	M.EcoNwEVDcm	1419	1419	100	1	1419	30201	28783	0	2813
S10EC	53	24281	M.EcoMVI	891	891	100	1	891	12434	13324	0	1725
S10EC	56	121359	M.EcoNwEDam	837	837	100	1	837	102552	103388	0	1659
S10EC	59	15043	M.EcoMVII	3606	3606	100	1	3606	8986	5381	0	6988
S10EC	49	13716	M.EcoMVIII	684	684	100	1	684	3930	3247	0	1356
S10EC	81	28028	Eco29kI	645	16	100	375	390	11479	11494	0.65	32.2
S10EC	188	344565	EcoDEC4CORF2749P	1041	16	100	654	669	165220	165235	1.1	32.2
S10EC	17	212657	Eco248534P	1053	16	100	941	956	160714	160699	1.1	32.2
S10EC	17	212657	Eco248534P	1053	15	100	972	986	34881	34895	4.2	30.2
S10EC	17	212657	Eco248534P	1053	15	100	66	80	164604	164590	4.2	30.2
S10EC	130	173001	EcoAPECORF2077P	1590	18	100	1494	1511	148777	148794	0.1	36.2
S10EC	130	173001	EcoAPECORF2077P	1590	16	100	827	842	62823	62838	1.6	32.2
S10EC	130	173001	EcoAPECORF2077P	1590	15	100	414	428	4567	4553	6.4	30.2
S10EC	149	6412	EcoDEC13EORF3046P	1191	16	100	920	935	46	61	1.2	32.2

S10EC	49	13716	Eco7A8ORF29P	684	606	98.51	79	684	3852	3247	0	1130
S10EC	188	344565	EcoDEC2CORF2043P	2019	21	95.24	1522	1542	344566	344586	0.52	34.2
S10EC	188	344565	EcoDEC2CORF2043P	2019	15	100	459	473	17351	17365	8.1	30.2
S10EC	188	344565	EcoDEC2CORF2043P	2019	19	94.74	1813	1831	40632	40614	8.1	30.2
S10EC	128	122843	Eco1886ORF14455P	1053	16	100	958	973	26331	26346	1.1	32.2
S10EC	188	344565	Eco1886ORF14565P	900	15	100	565	579	203765	203779	3.6	30.2
S10EC	49	13716	EcoR7ACORFAP	684	684	97.37	1	684	3930	3247	0	1213
S10EC	49	13716	EcoR100ORF1P	648	606	98.51	43	648	3852	3247	0	1130
S10EC	49	13716	Eco605ORFMP	684	684	93.86	1	684	3930	3247	0	1023
S10EC	188	344565	Eco84137ORF201P	1635	18	100	43	60	105440	105423	0.11	36.2
S10EC	188	344565	Eco84137ORF201P	1635	16	100	1212	1227	104153	104168	1.7	32.2
S10EC	188	344565	Eco84137ORF201P	1635	15	100	1080	1094	7497	7511	6.6	30.2
S10EC	174	195534	Eco1520ORF67P	1563	18	100	1501	1518	45075	45058	0.1	36.2
S10EC	174	195534	Eco1520ORF67P	1563	15	100	1239	1253	77633	77647	6.3	30.2
S10EC	69	33006	Eco15ORF4165P	1197	33	87.88	237	269	5651	5683	0.31	34.2
S12EC	75	33008	M1.EcoMI	1623	1623	100	1	1623	29723	28101	0	3009
S12EC	75	33008	M1.EcoMI	1191	1191	100	1	1191	28108	26918	0	2361
S12EC	15	116440	M.EcoMII	1620	1581	100	40	1620	70839	72419	0	3092
S12EC	1	383106	M.EcoMIII	1638	1638	100	1	1638	72881	71244	0	3247
S12EC	1	383106	M.EcoMIII	1638	16	100	1336	1351	275931	275946	1.7	32.2
S12EC	1	383106	M.EcoMIII	1638	15	100	992	1006	68168	68154	6.6	30.2
S12EC	1	383106	M.EcoMIII	1638	15	100	739	753	142674	142660	6.6	30.2
S12EC	1	383106	M.EcoMIII	1638	15	100	1509	1523	243108	243122	6.6	30.2
S12EC	71	15125	M.EcoMIV	765	765	100	1	765	6987	6223	0	1516
S12EC	32	35629	M.EcoMV	1050	18	100	628	645	9069	9052	0.068	36.2
S12EC	32	35629	M.EcoMV	1050	15	100	576	590	372	358	4.2	30.2
S12EC	45	38971	M.EcoNwEVDcm	1419	1419	100	1	1419	30196	28778	0	2813
S12EC	29	91638	M.EcoMVI	891	891	100	1	891	79791	80681	0	1725
S12EC	42	191371	M.EcoNwEDam	837	837	100	1	837	39463	38627	0	1659
S12EC	79	15045	M.EcoMVII	3606	3606	100	1	3606	8988	5383	0	6988
S12EC	120	5693	M.EcoMVIII	684	684	100	1	684	1832	2515	0	1356
S12EC	35	97404	Eco29kI	645	16	100	375	390	86006	85991	0.65	32.2
S12EC	35	97404	Eco29kI	645	15	100	494	508	9815	9829	2.6	30.2
S12EC	4	361321	EcoDEC4CORF2749P	1041	16	100	654	669	82579	82594	1.1	32.2
S12EC	34	115733	Eco248534P	1053	16	100	941	956	52020	52035	1.1	32.2
S12EC	34	115733	Eco248534P	1053	15	100	66	80	48130	48144	4.2	30.2

S12EC	32	35629	EcoAPECORF2077P	1590	18	100	1511	1528	29233	29216	0.1	36.2
S12EC	91	752	EcoDEC13EORF3046P	1191	16	100	920	935	31	16	1.2	32.2
S12EC	120	5693	Eco7A8ORF29P	684	606	98.51	79	684	1910	2515	0	1130
S12EC	26	102016	EcoDEC2CORF2043P	2019	21	95.24	1522	1542	84550	84530	0.52	34.2
S12EC	251	175827	Eco1886ORF14455P	1053	16	100	124	139	3765	3780	1.1	32.2
S12EC	251	175827	Eco1886ORF14455P	1053	16	100	803	818	81188	81203	1.1	32.2
S12EC	234	82185	Eco1886ORF14565P	900	15	100	273	287	23139	23153	3.6	30.2
S12EC	234	82185	Eco1886ORF14565P	900	15	100	289	303	42325	42311	3.6	30.2
S12EC	120	5693	EcoR7ACORFAP	684	684	97.37	1	684	1832	2515	0	1213
S12EC	120	5693	EcoR100ORF1P	648	606	98.51	43	648	1910	2515	0	1130
S12EC	120	5693	Eco605ORFMP	684	684	93.86	1	684	1832	2515	0	1023
S12EC	47	91914	Eco84137ORF201P	1635	22	95.45	558	579	40399	40378	0.11	36.2
S12EC	190	195536	Eco1520ORF67P	1563	18	100	1501	1518	150538	150555	0.1	36.2
S12EC	190	195536	Eco1520ORF67P	1563	15	100	1239	1253	117980	117966	6.3	30.2
S12EC	75	33008	Eco15ORF4165P	1197	33	87.88	237	269	27434	27402	0.31	34.2
