# Supplementary material for: Lineage-Specific Methyltransferases Define the Methylome of the Globally Disseminated Escherichia coli ST131 Clone
Source: mBio. 2015 Nov 17;6(6):e01602-15. doi: 10.1128/mBio.01602-15 (PMC4659465; doi:10.1128/mBio.01602-15)
Supplement: Table S4 — Primers used in this study. [file mbo005152543st4.pdf]

Table S4:Primers used in this study

Primer No.	oligo name	Sequence	Notes
**qRT-PCR**			
4688	4688-EC958_0008-F	TGGGGGACTAATCTTTCACG	qPCR to detect EC958_0008: 198bp
4689	4689-EC958_0008-R	AGCACCGCCGAATAATTTAG	
4686	4686-EC958_0009-F	ACTACAATCGTTCCCGGATG	qPCR to detect EC958_0009: 164bp
4687	4687-EC958_0009-R	CAGCTCCGGAAAATAAATCG	
4694	4694-EC958_0078-F	GGCAAGCTGACTTCCTTGAG	qPCR to detect EC958_0078: 168bp
4695	4695-EC958_0078-R	TCAGGGTTGGGTTACGTTTC	
4680	4680-EC958_0425-F	GTGCGCAACGTAAGCTGTTA	qPCR to detect EC958_0425: 179bp
4681	4681-EC958_0425-R	ACGTTAGGGATCGAATGCAC	
4692	4692-EC958_1101-F	GGGTGGTAAAGCATCACCTG	qPCR to detect EC958_1101: 190bp
4693	4693-EC958_1101-R	GAGGTACCGAAGCTGTGCTC	
5445	5445-EC958_1545-F	CACTGAACCGGCAGTATGTG	qPCR to detect EC958_1545: 130 bp
5446	5446-EC958_1545-R	TGTTTCCCGGGATAGAACTG	
4684	4684-EC958_2226-F	CGAATGGAACAAACATGCTG	qPCR to detect EC958_2226: 123 bp
4685	4685-EC958_2226-R	TCATCACTCACGCCTTCTTG	
4678	4678-EC958_3663-F	CCGACCCACCGTATAACATC	qPCR to detect EC958_3663: 127 bp
4679	4679-EC958_3663-R	CTGCCCTGCTTTTTCAGAAC	
4690	4690-EC958_3778-F	GCGCCGAGGTTTACTATCAG	qPCR to detect EC958_3778: 145 bp
4691	4691-EC958_3778-R	AACGGCACGTTAAACTCACC	
4696	4696-EC958_4083-F	TTCCGGTAATCAGGACGAAG	qPCR to detect EC958_4038: 146 bp
4697	4697-EC958_4083-R	TAGAGTCGGTACGGGTCAGG	
4698	4698-EC958_4974-F	TGTCCTTGGTAACTGCATCG	qPCR to detect EC958_A0009: 162 bp
4699	4699-EC958_4974-R	ATTTCATTACAGGCCGGTTG	
4774	4774-gapA-F	CGTTAAAGGCGCTAACTTCG	qPCR to detect gapA: 138 bp
4775	4775-gapA-R	ACGGTGGTCATCAGACCTTC	
**Deletion by lambda-red recombination**			
3746	3746-Cm.3a	tcctccttagttcctattcc	

3747	3747-Cm.4a	gtcttgagcgattgtgtagg	to amplify Cm cassette from pDK3
5256	5256_EC958_0078-0077-Fwsc	CCAGCACGAAAAAGCGATCC	Primers to KO EC958_0078-0077 in EC958
5257	5257_EC958_0078-0077-Fwup	AAATCAGCACAATGAACCCG	
5258	5258_EC958_0078-0077-Rvup	ggaataggaactaaggagga TGGTGCTTTGGCCATGATG	
5259	5259_EC958_0078-0077-Fwdn	cctacacaatcgctcaagac ACAGAAGCCGCATAACGCA	
5260	5260_EC958_0078-0077-Rvdn	TCATATAGGAAGGCGGGAG	
5261	5261_EC958_0078-0077-Rvsc	GATATAGAGAATGCCGTCAGG	
5262	5262_EC958_0425-0424-Fwsc	GCGCTGGAAGAGGTGATCT	Primers to KO EC958_0425-0424 in EC958
5263	5263_EC958_0425-0424-Fwup	GCCGACGAGCTGAAAGAGAC	
5264	5264_EC958_0425-0424-Rvup	ggaataggaactaaggagga TTCCAGTTCTACCACGTTCG	
5265	5265_EC958_0425-0424-Fwdn	cctacacaatcgctcaagac GTCAAATAATCCCCGGCGTG	
5266	5266_EC958_0425-0424-Rvdn	CAGCGTGGGTTGCAATAGC	
5267	5267_EC958_0425-0424-Rvsc	ATAAAGTGCTCTGGTGTGGC	
5430	5430-EC958_4083-Fwsc	AACGTCAACCATCAGTTAG	Primers to KO EC958_4083 in EC958
5431	5431-EC958_4083-Fwup	AAAGCAAGAAGCACAACTCC	
5432	5432-EC958_4083-Rvup	ggaataggaactaaggagga ATCGCGGAAGTCGTTACGG	
5295	5295_EC958_4082-4083-Fwdn	cctacacaatcgctcaagac GCCCCAGAGGTGATCTAAAC	
5296	5296_EC958_4082-4083-Rvdn	TTGCGTCGCTGAATATGCTC	
5297	5297_EC958_4082-4083-Rvsc	TTCTTATCCAGCGAGGCTTC	

"sc" screening primers used to amplify and Sanger sequence target mutant genes from putative isogenic mut
"up" primers amplifying 500 bp upstream fragment
"dn" primers amplifying 500 bp downstream fragment

ants
