# Supplementary material for: Lineage-Specific Methyltransferases Define the Methylome of the Globally Disseminated Escherichia coli ST131 Clone
Source: mBio. 2015 Nov 17;6(6):e01602-15. doi: 10.1128/mBio.01602-15 (PMC4659465; doi:10.1128/mBio.01602-15)
Supplement: Table S5 — Assignment of novel methylation motifs to specific MTase genes. [file mbo005152543st5.pdf]

Table S5: Assignment of novel methylation motifs to specific MTase genes

motif	WT			RM.EcoMI KO			RM.EcoMII KO			RM.EcoMIII KO		
	nDetected	nGenome	fraction	nDetected	nGenome	fraction	nDetected	nGenome	fraction	nDetected	nGenome	fraction
GATC	40820	40908	0.997	40535	40908	0.990882	40431	40908	0.98883397	40380	40908	0.987093
CANCATC	6528	6560	0.995	6475	6560	0.987	6445	6560	0.982	6425	6560	0.979
RTACNNNNGTG	703	706	0.995	704	705	0.998	NA	NA	NA	699	705	0.991
CACNNNNGTAY	703	706	0.995	703	705	0.997	NA	NA	NA	702	705	0.995
AACNNNNCTTT	846	847	0.998	841	847	0.992	841	847	0.992	NA	NA	NA
AAAGNNNNGTT	844	847	0.996	839	847	0.99	842	847	0.994	NA	NA	NA
GAGACC	378	378	1	NA	NA	NA	374	378	0.989	373	378	0.3986

RM.EcoMVII KO		
nDetected	nGenome	fraction
39900	40908	0.975
NA	NA	NA
693	705	0.982
700	705	0.992
823	847	0.971
827	847	0.976
365	378	0.965
